# Photocatalytic enantioselective α-aminoalkylation of acyclic imine derivatives by a chiral copper catalyst

**DOI:** 10.1038/s41467-019-11688-7

**Published:** 2019-08-23

**Authors:** Bowen Han, Yanjun Li, Ying Yu, Lei Gong

**Affiliations:** Key Laboratory of Chemical Biology of Fujian Province, iChEM, College of Chemistry and Chemical Engineering, Xiamen University, Xiamen, 361005 Fujian, China

**Keywords:** Asymmetric catalysis, Synthetic chemistry methodology, Photocatalysis

## Abstract

Copper-based asymmetric photocatalysis has great potential in the development of green synthetic approaches to chiral molecules. However, there are several formidable challenges associated with such a conception. These include the relatively weak visible light absorption, short excited-state lifetimes, incompatibility of different catalytic cycles, and the difficulty of the stereocontrol. We report here an effective strategy by means of single-electron-transfer (SET) initiated formation of radicals and photoactive intermediates to address the long-standing problems. Through elaborate selection of well-matched reaction partners, the chiral bisoxazoline copper catalyst is engaged in the SET process, photoredox catalysis, Lewis acid activation and asymmetric induction. Accordingly, a highly enantioselective photocatalytic α-aminoalkylation of acyclic imine derivatives has been accessed. This strategy sheds light on how to make use of diverse functions of a single transition metal catalyst in one reaction, and offers an economic and simplified approach to construction of highly valuable chiral vicinal diamines.

## Abstract (web summary)

Copper-based asymmetric photocatalysis has great synthetic potential, however it has been rarely exploited due to challenges inherent to such systems. Here, a chiral bisoxazoline copper catalyst is involved in a SET process, photoredox catalysis, Lewis acid activation and asymmetric induction to construct chiral vicinal diamines.

## Introduction

Copper catalysis has significant advantages including non-toxicity, low cost, easy handling and diverse functions, all of which make it attractive for green and sustainable chemical synthesis^1–3^. Copper can adopt oxidation states of 0, +1, +2, and +3, allowing for one-electron or two-electron transfer processes^4^. Consequently, various transformations through radical or organometallic pathways can be initiated with a copper catalyst. For instance, copper(II) is a useful one-electron oxidant which has been involved in a number of oxidative-coupling reactions^5^. Some copper(I) complexes have been determined to be photoactive species with highly negative Cu^I^*→Cu^II^ oxidation potentials, and are used in unique visible-light photoredox catalysis^6,7^. In addition, copper can provide strong Lewis acid activation towards substrates through coordination. The combination of copper derivatives with various chiral ligands had led to the discovery of a large number of chiral Lewis acid catalysts for asymmetric nucleophilic addition, rearrangement, cycloaddition, and other transformations^8,9^. These features characterize copper catalysis as an attractive tool in synthetic chemistry.

Recently, the unique features and reactivities of copper complexes in visible light photocatalysis have been well investigated by the group of Reiser and the others^10–17^. The diverse roles and possible inner-sphere catalytic manners enable copper-based catalysts attractive candidates for developing novel photochemical transformations^18,19^. In this context, utilizing one chiral copper complex as a multifunctional catalyst by employing its redox character, potential photochemical properties and asymmetric induction ability would be ideal and highly valuable for the development of economic and unique synthetic approaches to chiral molecules^20^. There are however several formidable challenges associated with such a conception. These include (i) the relatively weaker visible-light absorption and shorter excited-state lifetimes compared to those of conventional heavy metal photosentisizers^6,7^, (ii) the incompatibility of different catalytic cycles^21–25^, (iii) the difficulty of controlling the stereochemistry of the transformations of highly active intermediates such as radicals^26–33^, (iv) the possible diminishing of enantioselectivity raised by fast ligand exchange under photochemical conditions^34–36^. Recently, Fu et al. reported a copper(I)-catalyzed enantioselective C–N cross-coupling reaction driven by visible light, which opens new possibilities for asymmetric photocatalysis. In this reaction, the chiral copper(I) complex serves both as a pre-catalyst for the photoredox process and an asymmetric catalyst in the reductive elimination step^37^. Inspired by this impressive advance and one proof-of-principle study by us^38^, we questioned if incorporation of other functions of the metal, such as one-electron oxidative ability of copper(II) to initiate the radical process, generating in situ more efficient photoactive species would lead to the discovery of novel and economic copper-catalyzed asymmetric photochemical synthesis.

**Fig. 1:**
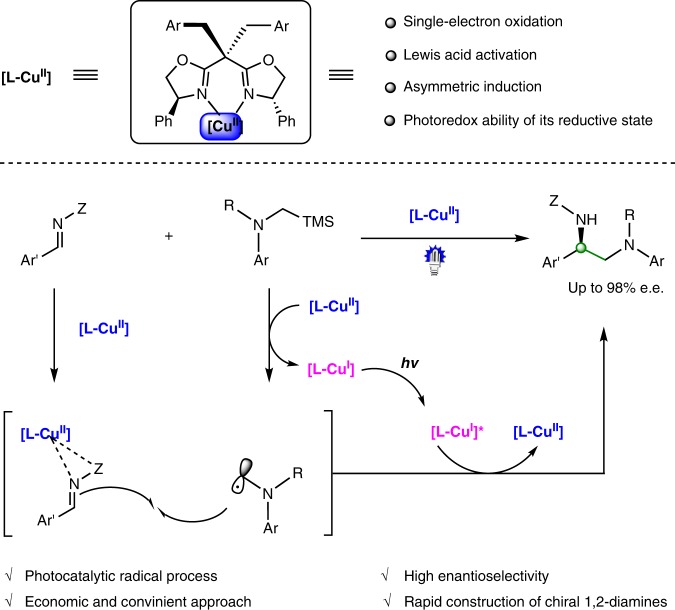
Overview of this work, photocatalytic enantioselective α-aminoalkylation of acyclic imine derivatives by a chiral copper complex

Herein, we report a highly enantioselective photocatalytic α-aminoalkylation of acyclic imine derivatives by a chiral bisoxazoline copper catalyst. This catalyst has been found to play diverse roles in the photochemical reaction, including initiation of the single electron transfer to generate an α-aminoalkyl radical and visible-light active copper(I) species, Lewis acid activation towards the imine substrate, asymmetric induction in the radical addition process, and anticipation of the photoredox process through copper(I) intermediates to close the catalytic cycles (Fig. 1). This method treats with difficult synthetic problems, i.e., enantioselective radical addition to acyclic imines to prepare optically active vicinal diamines^39,40^. and demonstrates how to make use of diverse functions of a single transition metal catalyst in one reaction.

## Results

### Mechanistic hypothesis

**Fig. 2:**
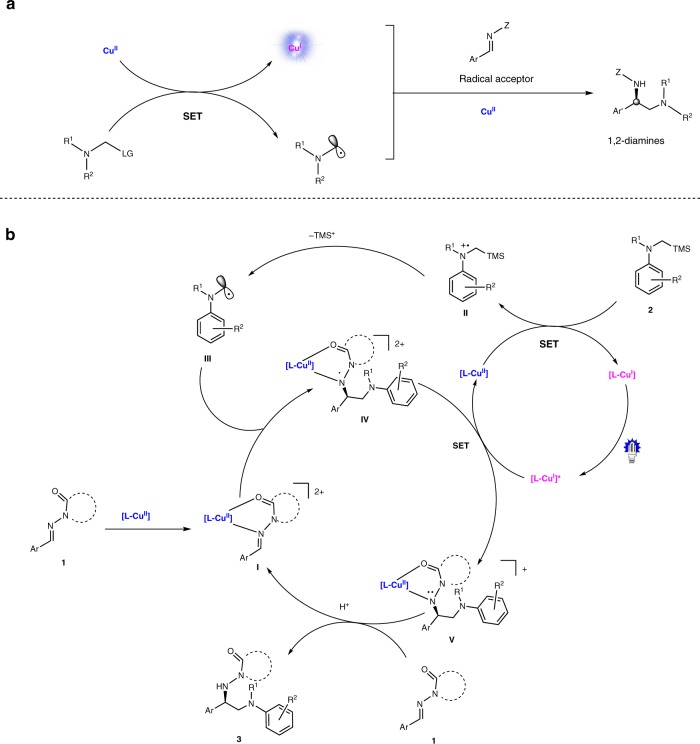
Initial design and mechanistic hypothesis. **a** Our strategy involves single-electron oxidation of electron-rich substrates to initiate the radical process and produce a photoredox active Cu^I^ species. **b** Mechanistic hypothesis, the chiral bisoxazoline copper(II) complex serves as a multifunctional catalyst

At the outset of this study, we envisioned that the catalyst was capable of initiating radical formation through single-electron transfer between the substrate and copper(II) (*E*_red_(Cu^II^/Cu^I^) = ~+0.8 V)^38^, and at the same time generating the photoactive copper(I) species. It is known that the tendency of copper(II) to participate in one-electron redox reactions is often manifested with electron-rich substrates^2^. Accordingly, we carefully examined the oxidation abilities of various electron-rich compounds and identified α-silylamines (*E*_ox_ = ~+0.6 V) as possible radical precursors^41^. The well-matched redox ability of the catalyst and substrate would allow for efficient single electron transfer process, generating the corresponding radical at an adequate concentration (Fig. 2a). *N*-acylhydrazones were considered as the radical acceptors, enabling efficient Lewis acid activation and asymmetric induction through bidentate chelation with the metal^42–44^. Significantly, the in situ-generated copper(I) species could be photoactive and have strong reductive ability at its excited state for the closure of catalytic cycles^18^.

A detailed hypothetical mechanism is illustrated in Fig. 2b. The *N*-acyclhydrazone substrate undergoes fast ligand exchange with the chiral copper catalyst **[L-Cu**^**II**^**]** to generate the intermediate complex (**I**). Meanwhile, the single electron transfer (SET) between α-silylamine and **[L-Cu**^**II**^**]** leads to the formation of the radical cation (**II**) and a copper(I) species **[L-Cu**^**I**^**]**. Dissociation of the TMS cation from **II** affords the α-aminoalkyl radical (**III**). The nucleophilic radical **III** proceeds with radical addition to the C = N double bond of complex **I** under stereocontrol and transformation to copper(II)-stabilized *N* radical species (**IV**)^45^. Reduction of **IV** by the excited copper(I) species**[L-Cu**^**I**^]* affords the monocationic complex (**V**), and this is followed by protonation and ligand exchange to release the chiral diamine product (**3**) and regenerated intermediate **I**.

### Investigation of reaction conditions

We began our study on the photocatalytic enantioselective α-aminoalkylation reaction with *N*-acylhydrazones (**1a–c**) and α-silylamine (**2a**) as model substrates, and the in situ-generated chiral bisoxazoline copper complex as the catalyst (Table 1). Under irradiation with a 24 W blue LEDs lamp at 25 °C in the presence of the premixed copper salt Cu(OTf)_2_ (10 mol%) and the chiral ligand (**L1**) **(**11 mol%) in THF, the reaction of **1a** and **2a** proceeded smoothly and provide the desired product (**3a**) in 70% yield and with 38% e.e. (Table 1, entry 1). Both the blue light (entries 2–5) and copper(II) catalyst (entries 6–9) are essential for the reaction to proceed, confirming that a photochemical process enabled by the chiral copper catalyst is involved. For example, stirring the mixture in the dark at room temperature (entry 2) or at 60 °C (entry 3), replacing the light source by a 24 W red LEDs (entry 4) or a 12 W UV lamp (entry 5), removing the copper catalyst (entry 6), replacing it with a Cu(I) catalyst or other transition metal complexes (entries 7–9), all were reactions that failed. Next, imine substrates bearing a different auxiliary *N*-acyl group were tested (entries 10, 11) and it was found that the reaction of the oxazolidin-2-one-derived substrate (**1b**) provided better enantioselectivity (56% e.e., entry 10). Ligand screening experiments revealed that the chiral bisoxazoline ligand (**L7**) gave the best results in terms of the yield (77%) and the enantioselectivity (80% e.e.) (entry 17). The enantioselectivity could be further improved to 93% e.e. by reducing the reaction temperature to −40 °C (entry 20).

**Table 1:** Optimization of the reaction conditions


Entry^a^	Metal salt	Subs.	Ligand	*T* (°C)	Light source	*t* (h)	Product	Yield (%)^b^	e.e. (%)^c^
1	Cu(OTf)_2_	**1a**	**L1**	25	Blue LEDs	16	**3a**	70	38
2	Cu(OTf)_2_	**1a**	**L1**	25	None	16	**3a**	0	n.a.
3	Cu(OTf)_2_	**1a**	**L1**	60	None	16	**3a**	0	n.a.
4	Cu(OTf)_2_	**1a**	**L1**	25	Red LEDs	16	**3a**	0	n.a.
5	Cu(OTf)_2_	**1a**	**L1**	25	UV lamps	16	**3a**	0	n.a.
6	None	**1a**	None	25	Blue LEDs	16	**3a**	0	n.a.
7	CuOTf	**1a**	**L1**	25	Blue LEDs	16	**3a**	0	n.a.
8	Ni(OTf)_2_	**1a**	**L1**	25	Blue LEDs	16	**3a**	0	n.a.
9	Fe(ClO_4_)_3_	**1a**	**L1**	25	Blue LEDs	16	**3a**	0	n.a.
10	Cu(OTf)_2_	**1b**	**L1**	25	Blue LEDs	16	**3b**	74	56
11	Cu(OTf)_2_	**1c**	**L1**	25	Blue LEDs	16	**3c**	44	17
12	Cu(OTf)_2_	**1b**	**L2**	25	Blue LEDs	16	**3b**	54	11
13	Cu(OTf)_2_	**1b**	**L3**	25	Blue LEDs	16	**3b**	64	5
14	Cu(OTf)_2_	**1b**	**L4**	25	Blue LEDs	16	**3b**	69	68
15	Cu(OTf)_2_	**1b**	**L5**	25	Blue LEDs	16	**3b**	74	71
16	Cu(OTf)_2_	**1b**	**L6**	25	Blue LEDs	16	**3b**	79	30
17	Cu(OTf)_2_	**1b**	**L7**	25	Blue LEDs	16	**3b**	77	80
18	Cu(OTf)_2_	**1b**	**L8**	25	Blue LEDs	16	**3b**	69	79
19	Cu(OTf)_2_	**1b**	**L7**	0	Blue LEDs	24	**3b**	79	86
20	Cu(OTf)_2_	**1b**	**L7**	−40	Blue LEDs	40	**3b**	77	93

n.a.  not applicable

^a^Reaction conditions: **1a–1c** (0.20 mmol), **2a** (0.60 mmol), metal salt (10 mol%), ligand (11 mol%), THF (2.0 mL), indicated temperature, indicated light source, under argon, see more details for the screening of solvent in Supplementary Table 1

^b^Isolated yield

^c^e.e. value determined by chiral HPLC

### Reaction scope

**Fig. 3:**
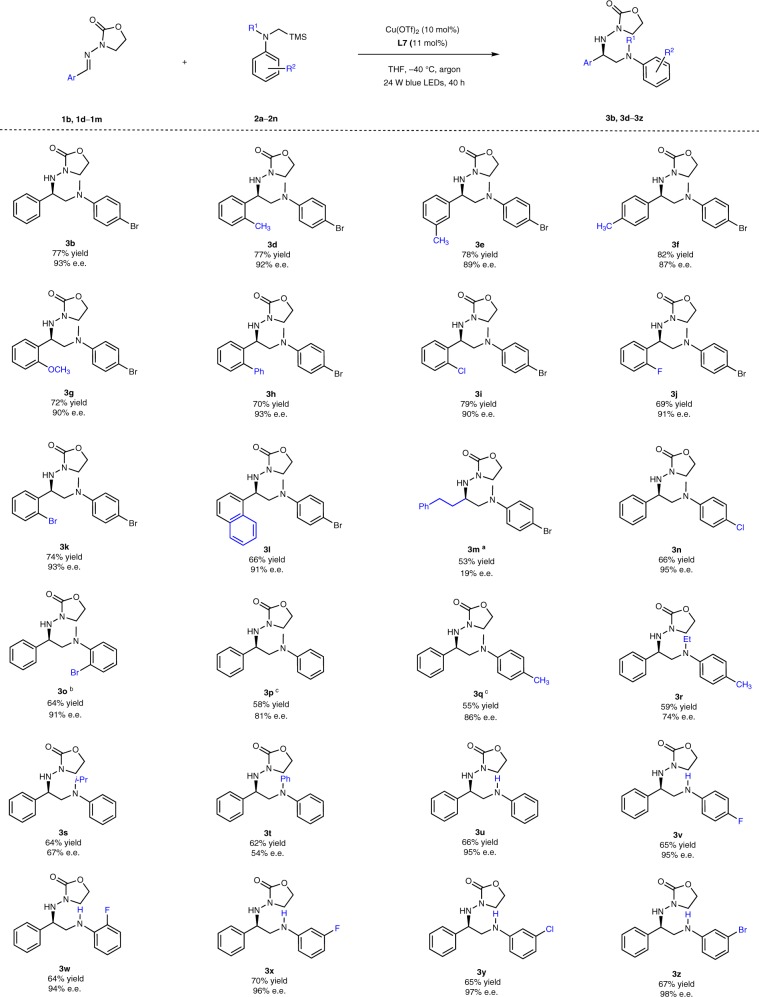
Reaction scope. ^a^Reaction performed at 25 °C. ^b^Reaction performed with 20 mol% Cu(OTf)_2_ and 22 mol% ligand **L8**. ^c^ Reaction performed with 20 mol% Cu(OTf)_2_ and 22 mol% ligand **L7**

With the optimal conditions in hand (Table 1, entry 20), we investigated the scope of the photocatalytic enantioselective α-aminoalkylation reaction (Fig. 3). With respect to the acyclic imine derivatives, we observed good to excellent yields (66–81%) and enantioselectivities (87–93% e.e.) with a range of *N*-acylhydrazones bearing different electron-donating (products **3d–3g**) or electron-withdrawing substituents (products **3h–3k**) on the phenyl ring, and a naphthyl moiety (product **3l**). As a trend, slightly lower e.e. values were obtained for electron-rich aryl *N*-acylhydrazones. It is worth to mention that alkyl-substituted aldimine (product **3m**) was not reactive enough under the standard reaction conditions. The desired product **3m** was obtained in 73% yield and with significantly lower enantioselectivity of 19% at a higher temperature of 25 °C within 40 h.

Regarding the α-aminoalkyl radical precursors, tertiary α-silylamines (products **3n–3t**) were all tolerated in the reaction and gave 54–95% e.e. Typically, electron-deficient α-silylamines were more beneficial to both the reaction rate and enantioselectivity, which might be due to a contribution from radical stabilization^25^. The α-silylamines bearing sterically more demanding substituents on the *N*-α-position (products **3r–3t**) gave somewhat reduced enantioselectivities. Moreover, secondary α-silylamines (products **3u–3z**) were well compatible with the reaction, and provide even better enantioselectivity (94–98% e.e.).

### Probing the radical pathways

**Fig. 4:**
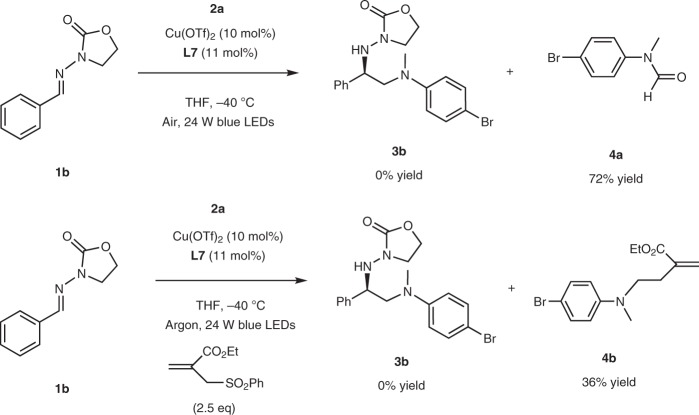
Probing of the α-aminoalkyl radicals. Photocatalytic reaction interfered by air or a radical acceptor

Several control experiments were conducted to probe the formation of the α-aminoalkyl radical. The copper-catalyzed photochemical reaction **1b** + **2a**→**3b** was completely inhibited under an air atmosphere, instead affording an amide side-product (**4a**) in 72% yield. While the reaction interfered with a radical acceptor, use of ethyl 2-((phenylsulfonyl)methyl)acrylate, led to the formation of **4b** (36%). These results suggest strongly the participation of α-aminoalkyl radicals in the photocatalytic reactions (Fig. 4).

The quantum yield (Φ) of the photocatalytic reaction **1b** + **2a**→**3b** was estimated (more details are available in Supplementary Fig. 2). The value of Φ = 0.16 suggests that the radical chain propagation pathway could not be predominant in the photochemical reactions. This result is also consistent with our mechanistic hypothesis.

### Investigations on the multiple functions of the copper catalyst

**Fig. 5:**
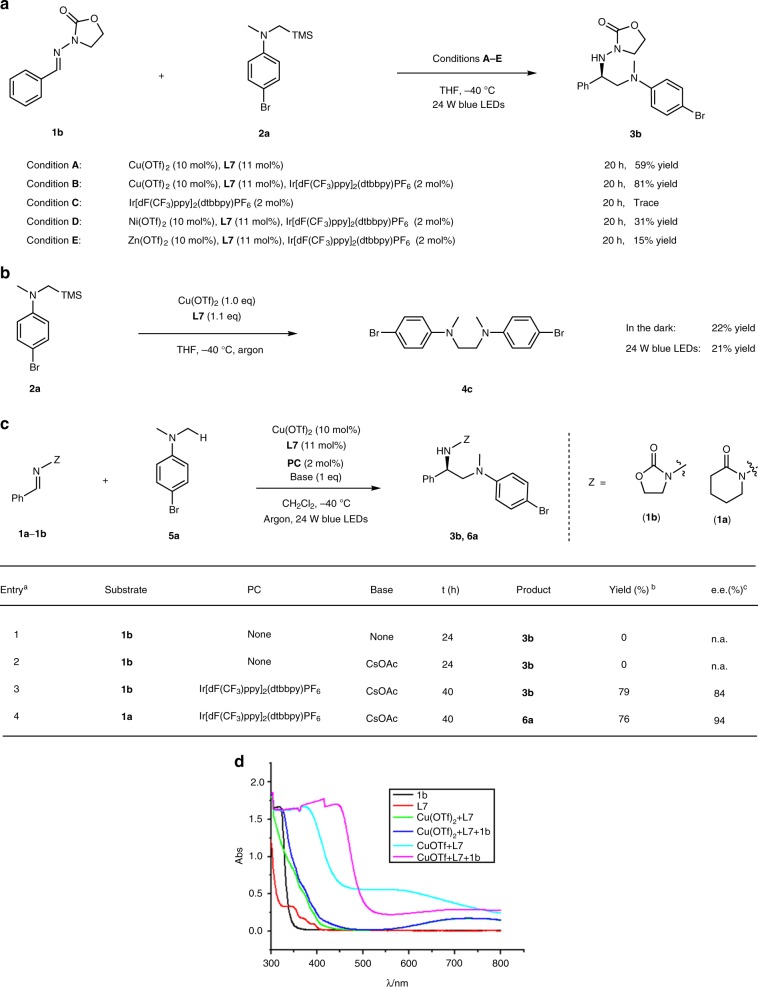
Probing the functions of the chiral copper catalyst. **a** Evidence for Lewis acid activation. **b** Evidence for single electron oxidation. **c** Evidence for powerful stereocontrol. ^a^Reaction conditions: **1b** or **1a** (0.20 mmol), **5a** (0.60 mmol), Cu(OTf)_2_ (10 mol%), **L7** (11 mol%), CH_2_Cl_2_ (2.0 mL), −40 °C, 24 W blue LEDs, under argon. ^b^Isolated yield. ^c^e.e. value determined by chiral HPLC. **d** Evidence for the potential photoactivity of copper(I) species

The roles of the chiral copper catalyst in the photochemical reactions were investigated in detail. First, the reaction **1b** + **2a**→**3b** can be dramatically accelerated by addition of other photosensitizers, such as Ir[dF(CF_3_)ppy]_2_(dtbbpy)PF_6_ (2 mol%). An increased yield of 81% is obtained within 20 h (Fig. 5a). Removal of the copper catalyst but keeping the Ir-based photosensitizer in the system led to the failure of the reaction. Replacing the copper catalyst by other Lewis acids, such as nickel or zinc complexes in the presence of Ir[dF(CF_3_)ppy]_2_(dtbbpy)PF_6_ still afforded the same product (**3b**) with isolated yield of 31% or 15%, respectively. These results suggested that the copper complex most likely performed an essential Lewis acid activation for the transformation.

Single electron oxidation of the α-silylamine substrates by the copper(II) catalyst to generate α-aminoalkyl radicals was confirmed by the stoichiometric reactions between **2a** and **[L7-Cu**^**II**^**]** (Fig. 5b). The reactions in the absence of light or under irradiation by a 24 W blue LED lamp afforded the self-coupling product **4a** in a similar yield, revealing that the single electron transfer process was thermodynamically favored and did not require acceleration by visble light.

To investigate the stereochemistry in the radical transformation process, an established photocatalytic method involving the direct α-C–H activation in the α-aminoalkyl radical generation was introduced to the system (Fig. 5c)^39,40^. In an initial trial, the reaction of oxazolidone-derived substrate (**1b**) and tertiary amine (**5a**) failed to afford the desired product in the absence or presence of an inorganic base (entries 1, 2). Apparently, single electron oxidation of the aromatic amine by copper(II) was unable to generate an adequate concentration of the α-alkylamino radical. The merger of the chiral copper catalyst and Ir[dF(CF_3_)ppy]_2_(dtbbpy)PF_6_ led to the formation of **3b** in 79% yield with 84% e.e. (entry 3). A significant increase in enantioselectivity was achieved when the coordination auxiliary (**Z**) was switched from the oxazolidonyl to a piperidin-2-only moiety (product **6a**, 96% e.e.) (entry 4). A possible interpretation is that the additional base (CsOAc) might have interfered with the chiral Lewis activation through coordination, thus requiring fine-tuning of the auxiliary to achieve the excellent enantioselectivity. All these results provided an indirect indication that the chiral bisoxazoline copper(II) catalyst has a high level of chiral recognition towards the α-alkylamino radical regardless of the formation pathways. Based on these observations, we developed an alternative synthesis of chiral 1,2-diamines through the direct functionalization of C(sp^3^)–H bonds of more readily available tertiary amines. The synergistic combination of chiral copper catalysis and iridium-based photoredox catalysis tolerates a broad scope of aromatic tertiary amines (products **6a–6h**, 64–82% yields and 78–96% e.e.) (see more details in Supplementary Method 3.4).

UV–Vis spectra of the reaction components were recorded to evaluate their potential as photoactive species (Fig. 5d). The individual substrate (**1b**) and chiral ligand (**L7**) show no obvious absorption in the visible light region. The in situ-generated catalyst **[L7-Cu**^**II**^**]** and potential intermediate **[L7-Cu**^**II**^**-1b]** have weak absorption in the range of 400–450 nm, but their reductive states (**[L7-Cu**^**I**^**]** and **[L7-Cu**^**I**^**-1b]**), which potentially exist in the catalytic system, exhibit significantly enhanced absorption. In combination with the control experiments (entries 2, 3 of Table 1 and conditions **B** of Fig. 5a) and the spectral data, it could be concluded that the copper(I) species generated in situ serves as a photosensitizer in the reaction. This conjecture is in agreement with the previously reported mechanism for copper(I)-catalyzed photochemical reactions^6,7^.

### Synthetic utility of the reactions

**Fig. 6:**
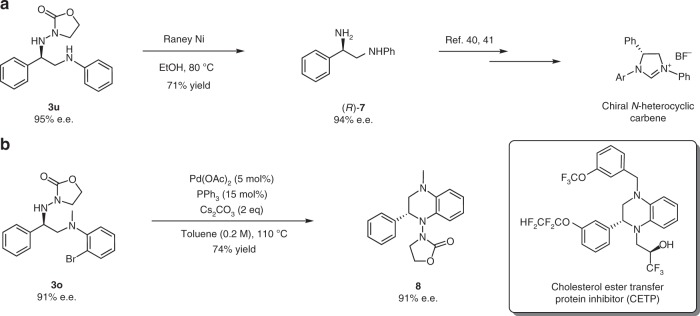
Synthetic utility of the reactions. **a** Transformation of product **3u** to chiral 1,2-diamine **7**. **b** Transformation of product **3o** to dihydroquinoxazline **8**

To expand the synthetic utility of this reactions, we investigated conditions for removal of the oxazolidinyl auxiliary (Fig. 6a). Typical conditions such as oxidative cleavage by SmI_2_ or reductive cleavage by Pd-catalyzed hydrogenation proved to be unselective, affording complex mixtures. The auxiliary could be cleanly removed, however, by the reaction with Raney nickel. For example, treatment of **3u** (95% e.e.) with excess Raney nickel in ethanol at 80 °C for just 1 h led to the formation of the chiral 1,2-diamine ((*R*)-**7**) in 71% yield and with 94% e.e. (Fig. 6a). Accordingly, the absolute configuration of **3u** was assigned as *R*, which is consistent with the proposed reaction mechanism. Chiral vicinal diamines have been widely used as chiral ligands, auxiliaries, and organocatalysts in asymmetric transformations^46^, and can be further converted to other highly valuable molecules, such as *N*-heterocyclic carbenes^47,48^. In addition, a Pd-catalyzed intramolecular cross-coupling reaction of the halogen-substituted product **3o** (91% e.e.) was developed, affording a bioactive dihydroquinoxazoline (**8**) in 74% yield and with 91% e.e. (Fig. 6b). The compounds with the similar core scaffold have been identified as cholesterol ester transfer protein inhibitors^49^.

## Discussion

We have developed an effective strategy for copper-based asymmetric photocatalysis through elaborate utilization of the single-electron oxidation ability of copper(II) to initiate the radical process and generate the photoactive intermediates, concurrently control the stereochemistry of the radical transformaiton. Based on this system, a highly efficient and economic process for photo-induced enantioselective α-aminoalkylation of acyclic imine derivatives has been achieved. The methodology provides possibilities for the use of chiral copper complexes as multifunctional asymmetric photocatalysts and offers a rapid and convenient approach to construction of highly valuable chiral vicinal diamines. Further investigations on detailed reaction mechanisms and applications are in progress in this laboratory.

## Methods

### Preparation of [L7-Cu^II^] and [L8-Cu^II^] complexes

A solution of Cu(OTf)_2_ (36.2 mg, 0.100 mmol) and chiral bisoxaoline ligand **L7** (57.0 mg, 0.110 mmol) or **L8** (82.0 mg, 0.110 mmol) in THF (5.0 mL) was stirred at 40 °C for 1 h, and was used freshly for the catalytic reactions.

### General procedure for the photocataytic reactions

A dried 10 mL Schlenk tube was charged with **1b**, **1d–1m** (0.20 mmol), **2a**–**2n** (0.60 mmol), and a chiral copper catalyst **[L7-Cu**^**II**^**] or [L8-Cu**^**II**^**]** (1.0 mL taken from a 20 mM solution in THF), and THF (1.0 mL). The mixture was degassed with three freeze–pump–thaw cycles. The Schlenk tube was positioned ~5 cm away from a 24 W blue LEDs lamp. After being stirred at −40 °C for the indicated time, the reaction mixture was evaporated to dryness. The residue was purified by flash chromatography on silica gel (eluted with PE/EtOAc = 3:2) to afford the non-racemic product **3b**, and **3d–3z**.

## Supplementary information

Supplementary Information

## Data Availability

The authors declare that the data supporting the findings of this study are available within this article and its Supplementary Information file. For the experimental procedures, and NMR and HPLC analysis of the compounds in this article, see Supplementary Methods and Tables in Supplementary Information.
