# Supplementary material for: Photocatalytic enantioselective α-aminoalkylation of acyclic imine derivatives by a chiral copper catalyst
Source: Nat Commun. 2019 Aug 23;10:3804. doi: 10.1038/s41467-019-11688-7 (PMC6707287; doi:10.1038/s41467-019-11688-7)
Supplement: Supplementary file 1 — Supplementary Information [file 41467_2019_11688_MOESM1_ESM.pdf]

Photocatalytic enantioselective α-aminoalkylation of acyclic imine derivatives enabled by a chiral copper catalyst

Han et al.

Supplementary Methods

1. General Information

Synthesis of the substrates were carried out under an atmosphere of argon with magnetic stirring unless stated otherwise. Visible-light-induced catalytic reactions were performed in 10 or 50 mL Schlenk tubes at the indicated temperature under an atmosphere of argon and under irradiation with a 24 W blue LEDs lamp (λ_{max} = 455 nm; commercial supplier: Hong Chang Lighting Co. Ltd., website: http://hongchang-led.taobao.com). Solvents were distilled under argon from dichloromethane (CH_2Cl_2) or tetrahydrofuran (THF). Acyclic imines **1a**[1], **1b**, **1d**, **1f**, **1i**, **1k**, **1l**, **1m**[2], **1g**[3], α-silylimines **2a**, **2b**, **2d**, **2e**, **2g**–**2i**[4], **2j**[5], **2f**, **2l**, **2m**[6] and bromide **S1**[7], **S3**[8] were synthesized according to the published procedures. All others reagents were purchased from commercial suppliers (TCI, Aldrich, Alfa, Adamas-beta® and J&K) and used without further purification. Flash column chromatography was performed with silica gel (300−400 mesh, pH = 6.7–7.0). ^{1}H NMR and ^{13}C NMR spectra were recorded on a Bruker AM (500 MHz) or Bruker AM (600 MHz) spectrometer at ambient temperature. NMR standards were used as follows: $CDCl_3$ = 7.26 ppm (^{1}H NMR), 77.16 ppm (^{13}C NMR). IR spectra were recorded on a Nicolet Avatar 330 FT-IR spectrophotometer. Chiral HPLC chromatograms were obtained from an Agilent 1260 Series HPLC system. High-resolution mass spectra were recorded on a Bruker En Apex Ultra 7.0 T FT-MS instrument using ESI technique. Optical rotations were measured on Anton Paar MCP 500 polarimeter at concentrations of 1.0 g per 100 mL. UV/Vis absorption spectra were recorded on a Shimadzu UV-2550 in a 10.0 mm quartz cuvette. Enantiomeric excess of the products were determined by HPLC analysis on chiral stationary phases.

2. Synthesis of the Ligands, Substrates and Racemic Reference Products

2.1 Synthesis of the Chiral Ligands

Chiral BOX ligands **L1−L3** were purchased from Aldrich or J&K and used directly without further purification. **L4−L6** was synthesized by a published procedure.[9,10]

L7 was prepared by a modified method.[11] Accordingly, to a solution of **S2** (720 mg, 2.35 mmol) in anhydrous THF (10 mL) was added NaH (60 % dispersion in mineral oil, 564 mg, 14.1 mmol) at room temperature under argon atmosphere. After stirred for 30 min, benzyl bromide **S1** (1.00 g, 4.70 mmol) in 10 mL anhydrous THF was then added dropwise to the mixture. The reaction was stirred at room temperature for 24 h, then quenched with H_2O (20 mL) and extracted with CH_2Cl_2. The combined organic layer was dried over anhydrous Na_2SO_4 and concentrated under reduced pressure. The residue was subjected to silica gel chromatography (eluted with PE:EtOAc = 10:1, PE = petroleum ether) to afford product **L7** as a white solid (980 mg, 1.71 mmol, yield: 73%).

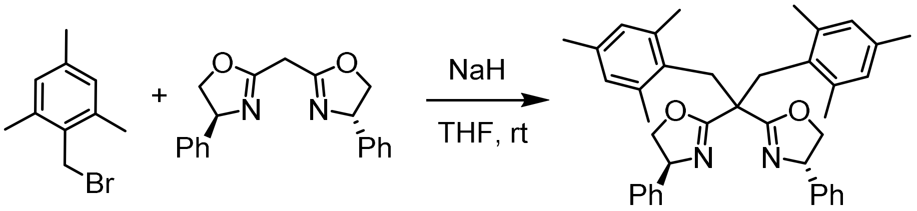

S1 **S2** **L7**

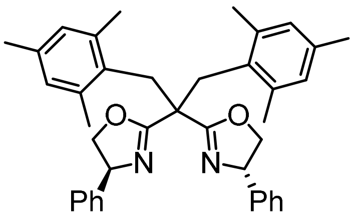

L7

^{1}H NMR (500 MHz, $CDCl_3$) δ 7.31 – 7.26 (m, 4H), 7.25 – 7.21 (m, 2H), 7.10 (d, J = 7.0 Hz, 4H), 6.81 (s, 4H), 4.71 (t, J = 9.3 Hz, 2H), 4.22 (d, J = 14.5 Hz, 2H), 3.84 (d, J = 14.5 Hz, 2H), 3.74 (dd, J = 9.9, 8.2 Hz, 2H), 3.50 (t, J = 8.3 Hz, 2H), 2.38 (s, 12H), 2.24 (s, 6H).

^{13}C NMR (126 MHz, $CDCl_3$) δ 168.9, 141.9, 139.0, 135.8, 133.8, 128.53, 128.47, 127.5, 127.2, 74.7, 69.6, 49.2, 41.8, 22.0, 21.0.

IR (film): ν (cm^{-1}) 3447, 3031, 2963, 2918, 1648, 1611, 1456, 1266, 1190, 1010, 935, 849, 760, 701, 615.

HRMS (ESI, m/z) calcd for $C_{39}H_{42}N_2O_2Na$ $(M+Na)^+$: 593.3138, found:593.3146.

L8 was prepared by a modified method.[11] Accordingly, to a solution of **S2** (306 mg, 1.00 mmol) in anhydrous THF (5 mL) was added NaH (60% dispersion in mineral oil, 240 mg, 6.00 mmol) at room temperature under argon atmosphere. After stirred for 30 min, benzyl bromide **S3** (600 mg, 2.00 mmol) in 5 mL anhydrous THF was then added dropwise to the mixture. The reaction was stirred at room temperature for 24 h, then quenched with H_2O (10 mL) and extracted with CH_2Cl_2. The combined organic layer was dried over anhydrous Na_2SO_4 and concentrated under reduced pressure. The residue was subjected to silica gel chromatography (eluted with PE:EtOAc = 4:1) to afford product **L8** as a white solid (570 mg, 0.77 mmol, yield: 77%).

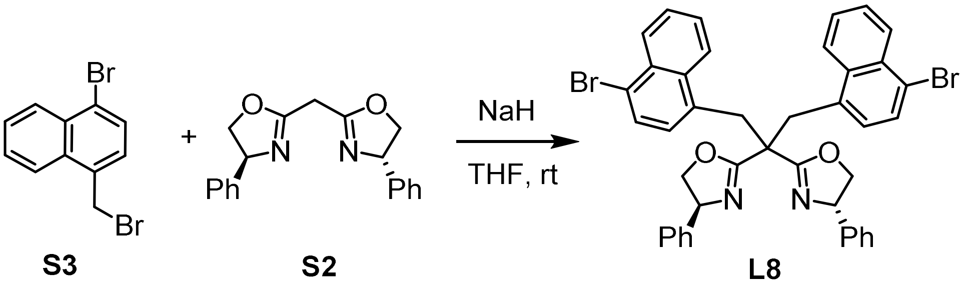

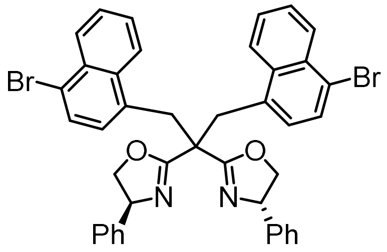

L8

^{1}H NMR (500 MHz, $CDCl_3$) δ 8.31 (dd, J = 34.4, 8.4 Hz, 4H), 7.70 – 7.57 (m, 4H), 7.57 – 7.47 (m, 4H), 7.37 – 7.26 (m, 6H), 7.05 (d, J = 7.2 Hz, 4H), 4.89 (t, J = 9.3 Hz, 2H), 4.23 – 4.03 (m, 4H), 3.96 (t, J = 9.3 Hz, 2H), 3.75 (t, J = 8.3 Hz, 2H).

^{13}C NMR (126 MHz, $CDCl_3$) δ 167.4, 141.7, 134.5, 133.5, 132.1, 129.5, 129.2, 128.7, 128.0, 127.7, 126.9, 126.8, 126.5, 124.4, 122.3, 74.8, 69.6, 48.7, 37.1.

IR (film): ν (cm^{-1}) 3448, 2963, 1735, 1685, 1654, 1560, 1382, 1190, 1010, 756, 701.

HRMS (ESI, m/z) calcd for $C_{41}H_{33}Br_2N_2O_2$ $(M+H)^+$: 745.883, found:745.0891.

2.2 Synthesis of the Substrates

2.2.1 Preparation of Acyclic Imines

Acyclic imines **1b**, **1d**, **1f**, **1g**, **1i**, **1k**, **1l**, **1m** were prepared by published procedures. **1e**, **1h**, **1j** were prepared by a two-step synthesis according to a modified method.[2]

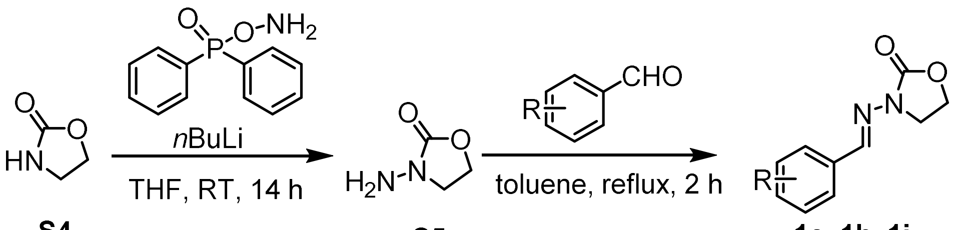

General procedure. To a solution of 2-oxazolidone (**S4**, 4.0 mmol) in THF at -78 °C was added dropwise *n*-BuLi (4.4 mmol, 2.4 M in hexanes). The reaction mixture was allowed to stir for 1 h at -78 °C, then *o*-(2,4-diphenylphosphinyl)-hydroxylamine (4.8 mmol) was added. The mixture was stirred at room temperature for 14 h. The crude product hydrazone (**S5**) was used in the subsequent step without further purification.

To a solution of hydrazone (**S5**) in toluene (0.50 M), aldehyde (2.0 eq), magnesium sulfate (4 eq) and TsOH•H_2O (0.020 eq) were added. The mixture was stirred under reflux for 2 h, then concentrated and purified by flash chromatography (eluted with EtOAc) to afford the corresponding acyclic imines (**1e**, **1h**, **1j**).

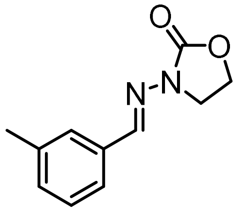

1e

According to the general procedure, 2-oxazolidone (348 mg, 4.0 mmol) was converted to corresponding acyclic imines **1e** (465 mg, 2.28 mmol, yield in total: 57%) as a white solid.

^{1}H NMR (500 MHz, $CDCl_3$) δ 7.69 (s, 1H), 7.63 (s, 1H), 7.49 (d, *J* = 7.6 Hz, 1H), 7.28 (t, *J* = 7.7 Hz, 1H), 7.21 (d, *J* = 7.6 Hz, 1H), 4.65 – 4.40 (m, 2H), 4.00 – 3.87 (m, 2H), 2.37 (s, 3H).

^{13}C NMR (126 MHz, $CDCl_3$) δ 154.5, 144.8, 138.7, 133.8, 131.3, 128.7, 127.8, 125.2, 61.4, 42.7, 21.4.

IR (film): ν (cm^{-1}) 3361, 2921, 2851, 1773, 1406, 1230, 1086, 1038, 746.

HRMS (ESI, m/z) calcd for $C_{11}H_{12}N_2O_2Na$ $(M+Na)^+$: 227.0791, found: 227.0791.

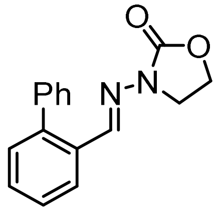

1h

According to the general procedure, 2-oxazolidone (348 mg, 4.0 mmol) was converted to corresponding acyclic imines **1h** (588 mg, 2.21 mmol, yield in total: 55%) as a white solid.

^{1}H NMR (500 MHz, $CDCl_3$) δ 8.26 – 8.14 (m, 1H), 7.63 (s, 1H), 7.48 – 7.36 (m, 5H), 7.36 – 7.30 (m, 3H), 4.50 – 4.41 (m, 2H), 3.75 – 3.63 (m, 2H).

^{13}C NMR (126 MHz, $CDCl_3$) δ 154.4, 143.8, 142.5, 139.7, 131.3, 130.2, 130.1, 129.9, 128.5, 127.9, 127.8, 126.8, 61.3, 42.4.

IR (film): ν (cm^{-1}) 3361, 2920, 2850, 1762, 1477, 1406, 1230, 1086, 1038, 751, 696.

HRMS (ESI, m/z) calcd for $C_{16}H_{14}N_2O_2Na$ $(M+Na)^+$: 289.0947, found: 289.0947.

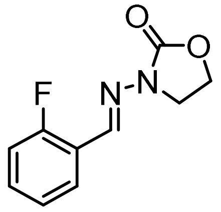

1j

According to the general procedure, 2-oxazolidone (348 mg, 4.0 mmol) was converted to corresponding acyclic imines **1j** (447 mg, 2.15 mmol, yield in total: 57%) as a white solid.

^{1}H NMR (500 MHz, $CDCl_3$) δ 8.17 – 8.02 (m, 1H), 7.89 (s, 1H), 7.36 (td, *J* = 7.3, 1.4 Hz, 1H), 7.18 (t, *J* = 7.6 Hz, 1H), 7.11 – 6.99 (m, 1H), 4.66 – 4.49 (m, 2H), 4.09 – 3.84 (m, 2H).

^{13}C NMR (126 MHz, $CDCl_3$) δ 160.5, 154.3 (d, *J* = 3.5 Hz), 137.1 (d, *J* = 5.6 Hz), 131.9 (d, *J* = 8.6 Hz), 127.2 (d, *J* = 2.5 Hz), 124.7 (d, *J* = 3.5 Hz), 121.7 (d, *J* = 9.6 Hz), 115.7 (d, *J* = 21.0 Hz), 61.5, 42.5.

IR (film): ν (cm^{-1}) 3468, 2921, 2852, 1773, 1654, 1401, 1225, 1090, 773, 746.

HRMS (ESI, m/z) calcd for $C_{10}H_9N_2O_2FNa$ $(M+Na)^+$: 231.0540, found: 231.0527.

2.2.2 Preparation of α-Silylimines

α-Silylimines **2a**, **2b**, **2d**–**2j**, **2l**, **2m** were prepared by published procedures. **2c**, **2k** and **2n** were prepared by a two-step synthesis according to a modified method.[4]

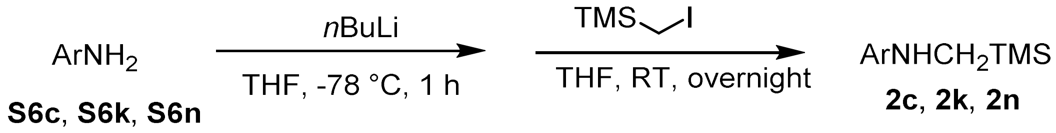

General procedure. A typical experimental procedure for the synthesis of α-silylimines **2c**, **2k** and **2n** is described below. To a solution of aniline (**S6c**, **S6k**, **S6n**, 5.0 mmol) in THF (20 mL) at -78 °C was added dropwise *n*-BuLi (4.4 mmol, 2.4 M in hexanes). The resulting mixture was stirred at room temperature for 1 h. (Iodomethyl)trimethylsilane (5.0 mmol) were added at -78 °C, then the resulting mixture was stirred at room temperature overnight. H_2O (25 mL) was added, and the resulting mixture was extracted with Et_2O (25 mL x 3). The combined organic layer was washed with brine and dried over anhydrous $MgSO_4$. After

concentration in vacuo, the residue was purified by column chromatography (eluted with pure hexane) to give α-silylimines **2c**, **2k** and **2n**.

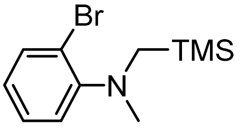

2c

According to the general procedure, 2-bromo-*N*-methylbenzenamine (**S6c**, 930 mg, 5.00 mmol) was converted to corresponding α-silylimines **2c** (870 mg, 3.20 mmol, yield: 64%) as a pale yellow oil.

^{1}H NMR (500 MHz, $CDCl_3$) δ 7.52 (dd, *J* = 7.9, 1.5 Hz, 1H), 7.25 – 7.20 (m, 1H), 7.15 (dd, *J* = 8.0, 1.7 Hz, 1H), 6.86 (ddd, *J* = 7.9, 7.2, 1.7 Hz, 1H), 2.73 (s, 3H), 2.58 (s, 2H), 0.04 (s, 9H).

^{13}C NMR (126 MHz, $CDCl_3$) δ 156.9, 139.9, 129.1, 125.4, 122.2, 99.5, 48.8, 47.4, -1.1.

IR (film): ν (cm^{-1}) 3421, 2955, 2896, 2801, 1613, 1510, 1439, 1315, 1251, 1220, 861, 820, 784, 712, 510.

HRMS (ESI, m/z) calcd for $C_{11}H_{19}BrNSi$ $(M+H)^+$: 272.0465, found: 272.0472.

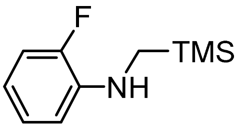

2k

According to the general procedure, 2-fluoroaniline (**S6k**, 556 mg, 5.00 mmol) was converted to corresponding α-silylimines **2k** (650 mg, 3.30 mmol, yield: 66%) as a yellow oil.

^{1}H NMR (500 MHz, $CDCl_3$) δ 7.10 (t, *J* = 7.7 Hz, 1H), 7.03 (ddd, *J* = 12.0, 8.0, 1.3 Hz, 1H), 6.90 – 6.81 (m, 1H), 6.72 – 6.63 (m, 1H), 3.81 (s, 1H), 2.58 (s, 2H), 0.24 (s, 9H).

^{13}C NMR (126 MHz, $CDCl_3$) δ 150.8, 139.1 (d, *J* = 11.2 Hz), 124.7 (d, *J* = 3.4 Hz), 116.1 (d, *J* = 7.0 Hz), 114.1 (d, *J* = 18.3 Hz), 111.9 (d, *J* = 3.5 Hz), 33.0, -2.6.

IR (film): ν (cm^{-1}) 3421, 2955, 2896, 2801, 1613, 1510, 1439, 1315, 1251, 1220, 861, 820, 784, 712, 510.

HRMS (ESI, m/z) calcd for $C_{10}H_{17}FNSi$ $(M+H)^+$: 198.1109, found: 198.1094.

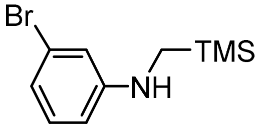

2n

According to the general procedure, 3-bromoaniline (**S6n**, 1.20 g, 5.00 mmol) was converted to corresponding α-silylimines **2n** (1.00 g, 3.89 mmol, yield: 78%) as a yellow oil.

^{1}H NMR (500 MHz, $CDCl_3$) δ 7.03 (dd, *J* = 11.0 Hz, 1H), 6.90 – 6.70 (m, 2H), 6.66 – 6.48 (m, 1H), 3.57 (s, 1H), 2.49 (s, 2H), 0.17 (s, 9H).

^{13}C NMR (126 MHz, $CDCl_3$) δ 151.9, 130.4, 123.4, 119.7, 115.0, 111.5, 33.5, -2.6.

IR (film): ν (cm^{-1}) 3422, 2954, 2894, 2803, 1596, 1497, 1480, 1320, 1250, 985, 861, 760, 681.

HRMS (ESI, m/z) calcd for $C_{10}H_{17}BrNSi$ $(M+H)^+$: 258.0308, found: 258.0313.

2.3 Synthesis of the Racemic Products as References

2.3.1 Preparation of 20 mM Solution of rac-[L7-Cu^{II}] in THF

A solution of $Cu(OTf)_2$ (36.2 mg, 0.10 mmol) and racemic ligand rac-**L7** (62.7 mg, 0.110 mmol) in THF (5.0 mL) was stirred at 40 °C for 1 h, which was used freshly for the catalytic reactions.

2.3.2 Preparation of 20 mM Solution of rac-[L7-Cu^{II}] in CH_2Cl_2

A solution of $Cu(OTf)_2$ (36.2 mg, 0.10 mmol) and racemic ligand rac-**L7** (62.7 mg, 0.110 mmol) in CH_2Cl_2 (5.0 mL) was stirred at 40 °C for 1 h, which was used freshly for the catalytic reactions.

2.3.3 Synthesis of Racemic Reference Compounds rac-3b, 3d–3z

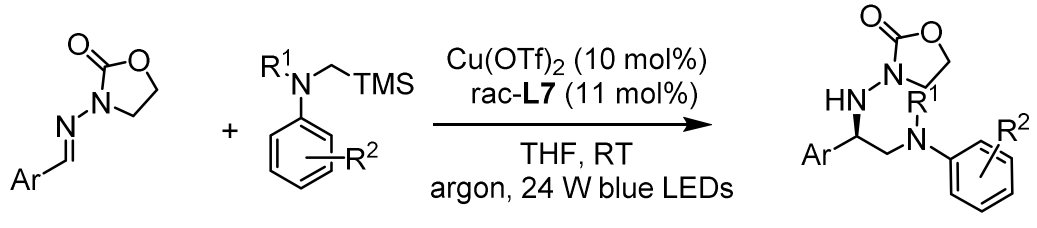

1b, 1d–1m **2a–2n** **3b, 3d–3z**

General procedure. A dried 10 mL Schlenk tube was charged with **1b**, **1d–1m** (0.20 mmol), **2a–2n** (0.60 mmol), racemic catalyst rac-**[L7-Cu^{II}]** (1.0 mL taken from the 20 mM solution in THF), and THF (1.0 mL). The mixture was degassed via three freeze-pump-thaw cycles. The Schlenk tube was positioned approximately 5 cm away from a 24 W blue LEDs lamp. After being stirred at 25 °C (monitored by TLC analysis), the reaction mixture was concentrated and then purified by flash chromatography on silica gel (eluted with PE:EtOAc = 2:1) to afford racemic product rac-**3b**, **3d–3z** as HPLC reference for the determination of enantiomeric excess.

2.3.4 Synthesis of Racemic Reference Compounds *rac*-6a–6h

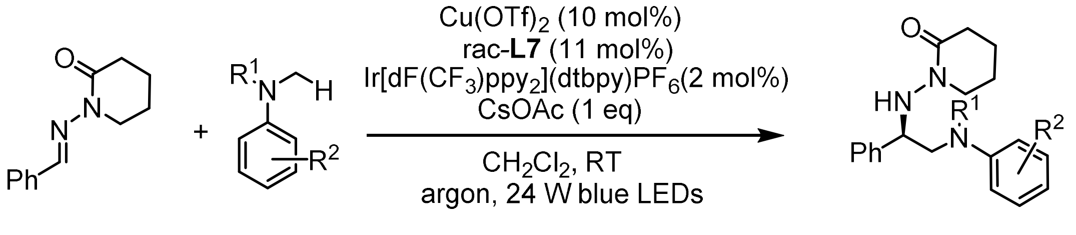

1a **5a–5h** **6a–6h**

General procedure. A dried 10 mL Schlenk tube was charged with **1a** (0.20 mmol), **5a–h** (0.60 mmol), Ir[dF(CF_3)ppy$_2$](dtbpy)PF_6 (0.0040 mmol), racemic catalyst rac-**[L7-Cu^{II}]** (1.0 mL taken from the 20 mM solution in CH_2Cl_2), and CH_2Cl_2 (1.0 mL). The mixture was degassed via three freeze-pump-thaw cycles. The Schlenk tube was positioned approximately 5 cm away from a 24 W blue LEDs lamp. After being stirred at 25 °C (monitored by TLC analysis), the reaction mixture was concentrated and then purified by flash chromatography on silica gel (eluted with PE:EtOAc = 2:1) to afford racemic product rac-**6a–6h** as HPLC reference for the determination of enantiomeric excess.

3. Copper-Catalyzed Asymmetric Photoredox Reactions

3.1 Optimization for Photocatalytic Enantioselective α-Aminoalkylation of Acyclic Imine Derivatives

3.1.1 Preparation of 20 mM Solutions of Non-Racemic Metal Catalysts in THF

A solution of metal salt (0.020 mmol) and chiral ligands **L1–L8** (0.022 mmol) in THF (1.0 mL) was stirred at 40 °C for 1 h, which was used freshly for the catalytic reactions.

3.1.2 General Procedure

A dried 10 mL Schlenk tube was charged with acyclic imine **1b** (0.20 mmol), α-silylimines **2a** (0.60 mmol), metal catalyst (0.020 mol, 1.0 mL taken from the 20 mM solution in THF), and THF (1.0 mL). The mixture was degassed via three freeze-pump-thaw cycles. The Schlenk tube was positioned approximately 5 cm away from the indicated light source. After being stirred at the indicated temperature for the indicated time, the reaction mixture was concentrated to dryness. The e.e. value was determined by chiral HPLC chromatography using a Daicel Chiralpak AD-H column or others.

Supplementary Table 1. Optimization of Photocatalytic Enantioselective α-aminoalkylation of Acyclic Imine Derivatives by a Chiral Copper Complex

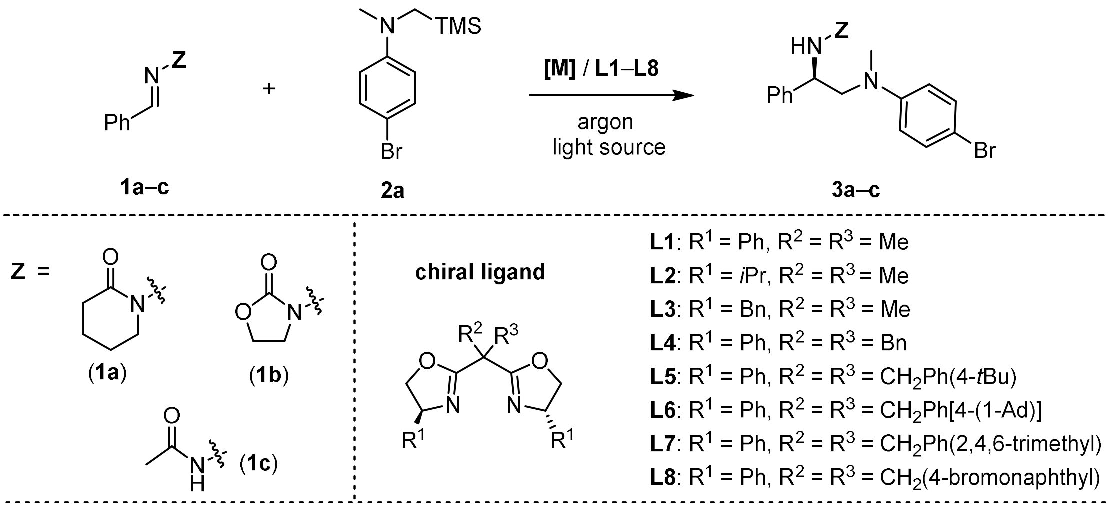

entry[a]	metal salt	subs.	ligand	solvent	T (°C)	light source	t (h)	product	yield (%)[b]	e.e. (%)[c]
1	$Cu(OTf)_2$	**1a**	**L1**	THF	25	blue LEDs	16	**3a**	70	38
2	$Cu(OTf)_2$	**1a**	**L1**	MeOH	25	blue LEDs	16	**3a**	52	13
3	$Cu(OTf)_2$	**1a**	**L1**	MeCN	25	blue LEDs	16	**3a**	0	n.a.
4	$Cu(OTf)_2$	**1a**	**L1**	$CHCl_3$	25	blue LEDs	16	**3a**	0	n.a.
5	$Cu(OTf)_2$	**1a**	**L1**	THF	25	none	16	**3a**	0	n.a.
6	$Cu(OTf)_2$	**1a**	**L1**	THF	60	none	16	**3a**	0	n.a.
7	$Cu(OTf)_2$	**1a**	**L1**	THF	25	red LEDs	16	**3a**	0	n.a.
8	$Cu(OTf)_2$	**1a**	**L1**	THF	25	UV lamp	16	**3a**	0	n.a.
9	none	**1a**	none	THF	25	blue LEDs	16	**3a**	0	n.a.
10	CuOTf	**1a**	**L1**	THF	25	blue LEDs	16	**3a**	0	n.a.
11	$Ni(OTf)_2$	**1a**	**L1**	THF	25	blue LEDs	16	**3a**	0	n.a.
12	$Fe(ClO_4)_3$	**1a**	**L1**	THF	25	blue LEDs	16	**3a**	0	n.a.

13	$Cu(OTf)_2$	**1b**	**L1**	THF	25	blue LEDs	16	**3b**	74	56
14	$Cu(OTf)_2$	**1c**	**L1**	THF	25	blue LEDs	16	**3c**	44	17
15	$Cu(OTf)_2$	**1b**	**L2**	THF	25	blue LEDs	16	**3b**	54	11
16	$Cu(OTf)_2$	**1b**	**L3**	THF	25	blue LEDs	16	**3b**	64	5
17	$Cu(OTf)_2$	**1b**	**L4**	THF	25	blue LEDs	16	**3b**	69	68
18	$Cu(OTf)_2$	**1b**	**L5**	THF	25	blue LEDs	16	**3b**	74	71
19	$Cu(OTf)_2$	**1b**	**L6**	THF	25	blue LEDs	16	**3b**	79	30
20	$Cu(OTf)_2$	**1b**	**L7**	THF	25	blue LEDs	16	**3b**	77	80
21	$Cu(OTf)_2$	**1b**	**L8**	THF	25	blue LEDs	16	**3b**	69	79
22	$Cu(OTf)_2$	**1b**	**L7**	THF	0	blue LEDs	24	**3b**	79	86
23	$Cu(OTf)_2$	**1b**	**L7**	THF	-40	blue LEDs	40	**3b**	77	93

[a] Reaction conditions: **1a–c** (0.20 mmol), **2a** (0.60 mmol), metal salt (10 mol%), ligand (11 mol%), solvent (2.0 mL), indicated temperature, indicated light source, under argon. [b] Isolated yield. [c] E.e. value determined by chiral HPLC. n.a. = not applicable.

3.2 Substrate Scope for Photocatalytic Enantioselective α-Aminoalkylation of Acyclic Imine Derivatives

3.2.1 Preparation of 20 mM Solution of Non-Racemic Catalyst [L7-Cu^{II}] and [L8-Cu^{II}] in THF

A solution of $Cu(OTf)_2$ (36.2 mg, 0.100 mmol) and non-racemic ligand **L7** (62.7 mg, 0.110 mmol) or **L8** (82.0 mg, 0.110 mmol) in THF (5.0 mL) was stirred at 40 °C for 1 h, which was used freshly for the catalytic reactions.

3.2.2 General Procedure

A dried 10 mL Schlenk tube was charged with **1b**, **1d**–**m** (0.20 mmol), **2a**–**2n** (0.60 mmol), chiral copper catalyst **[L7-Cu^{II}]** or **[L8-Cu^{II}]** (1.0 mL taken from the 20 mM solution in

THF), and THF (1.0 mL). The mixture was degassed via three freeze-pump-thaw cycles. The Schlenk tube was positioned approximately 5 cm away from a 24 W blue LEDs lamp. After being stirred at -40 °C for the indicated time, the reaction mixture was concentrated to dryness. The residue was purified by flash chromatography on silica gel (eluted with PE/EtOAc = 2:1) to afford non-racemic product **3b**, **3d–3z**.

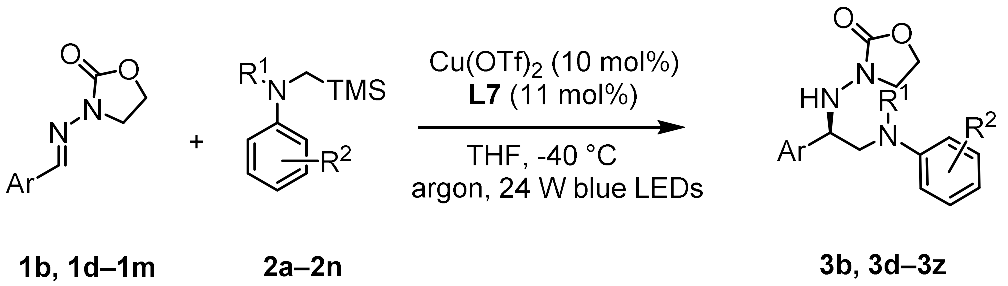

3.2.3 Experimental Details and Characterization Data

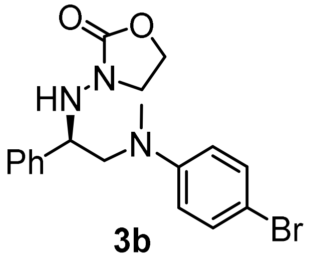

3b

A dried 10 mL Schlenk tube was charged with **1b** (38.0 mg, 0.20 mmol), **2a** (163.4 mg, 0.60 mmol), chiral copper catalyst **[L7-Cu^{II}]** (1.0 mL taken from the 20 mM solution in THF), and THF (1.0 mL). The mixture was degassed via three freeze-pump-thaw cycles. The Schlenk tube was positioned approximately 5 cm away from a 24 W blue LEDs lamp. After being stirred at -40 °C for 40 h, the reaction mixture was concentrated and then purified by flash chromatography on silica gel (eluted with PE:EtOAc = 2:1) to afford product **3b** as a white solid (59.7 mg, 0.153 mmol, yield: 77%). Enantiomeric excess was established by HPLC analysis using a Chiralpak IC column, e.e. = 93% (HPLC: IC, 220 nm, *n*-hexane/isopropanol = 90:10, flow rate: 1 mL/min, 30 °C, t_r(minor) = 33.8 min, t_r(major) = 36.0 min.) $[\alpha]_D^{23}$ = +33.5° (*c* = 1.0, CH_2Cl_2).

^{1}H NMR (500 MHz, $CDCl_3$) δ 7.44 (dt, *J* = 3.4, 2.0 Hz, 2H), 7.42 – 7.26 (m, 5H), 6.75 – 6.65 (m, 2H), 4.75 (s, 1H), 4.59 (dd, *J* = 9.4, 5.0 Hz, 1H), 4.14 (td, *J* = 8.9, 4.9 Hz, 1H), 4.01 (q, *J*

= 8.7 Hz, 1H), 3.62 (dd, J = 14.6, 9.5 Hz, 1H), 3.46 (dd, J = 17.0, 8.8 Hz, 1H), 3.23 – 3.11 (m, 2H), 2.91 (s, 3H).

^{13}C NMR (126 MHz, $CDCl_3$) δ 158.8, 149.2, 139.9, 132.0, 128.8, 128.3, 128.0, 115.2, 109.7, 62.0, 61.5, 58.8, 47.9, 38.5.

IR (film): ν (cm^{-1}) 3483, 3275, 3031, 2922, 1751, 1677, 1593, 1496, 1375, 1244, 1093, 1030, 808, 761, 703.

HRMS (ESI, m/z) calcd for $C_{18}H_{21}BrN_3O_2$ $(M+H)^+$: 390.0812, found: 390.0818.

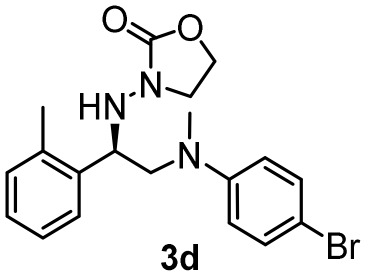

3d

A dried 10 mL Schlenk tube was charged with **1d** (40.8 mg, 0.20 mmol), **2a** (163.4 mg, 0.60 mmol), chiral copper catalyst **[L7-Cu^{II}]** (1.0 mL taken from the 20 mM solution in THF), and THF (1.0 mL). The mixture was degassed via three freeze-pump-thaw cycles. The Schlenk tube was positioned approximately 5 cm away from a 24 W blue LEDs lamp. After being stirred at -40 °C for 40 h, the reaction mixture was concentrated and then purified by flash chromatography on silica gel (eluted with PE:EtOAc = 2:1) to afford product **3d** as a white solid (62.1 mg, 0.154 mmol, yield: 77%). Enantiomeric excess was established by HPLC analysis using a Chiralpak AD-H column, e.e. = 92% (HPLC: AD-H, 220 nm, *n*-hexane/isopropanol = 90:10, flow rate: 1 mL/min, 30 °C, t_r(minor) = 13.3 min, t_r(major) = 22.7 min.) $[\alpha]_D^{23}$ = +36.2° (c = 1.0, CH_2Cl_2).

^{1}H NMR (500 MHz, $CDCl_3$) δ 7.67 (d, J = 7.6 Hz, 1H), 7.33 – 7.27 (m, 2H), 7.24 (d, J = 1.1 Hz, 1H), 7.19 (ddd, J = 20.8, 10.6, 4.3 Hz, 2H), 6.74 – 6.65 (m, 2H), 4.95 (dd, J = 9.2, 5.0 Hz, 1H), 4.73 (s, 1H), 4.16 (td, J = 8.9, 5.0 Hz, 1H), 4.03 (q, J = 8.6 Hz, 1H), 3.59 (dd, J = 14.6, 9.3 Hz, 1H), 3.47 (dd, J = 17.1, 8.6 Hz, 1H), 3.24 (td, J = 8.4, 5.1 Hz, 1H), 3.16 (dd, J = 14.6, 5.1 Hz, 1H), 2.91 (s, 3H), 2.32 (s, 3H).

^{13}C NMR (126 MHz, $CDCl_3$) δ 159.1, 149.4, 138.1, 136.9, 132.1, 130.9, 127.8, 127.1, 126.5, 115.3, 109.8, 61.7, 58.1, 48.2, 38.4, 29.9, 19.6.

IR (film): ν (cm^{-1}) 3482, 3276, 2963, 2922, 1751, 1677, 1592, 1496, 1401, 1260, 1092, 807, 761, 690.

HRMS (ESI, m/z) calcd for $C_{19}H_{22}BrN_3O_2Na$ $(M+Na)^+$: 426.0788, found: 426.0789.

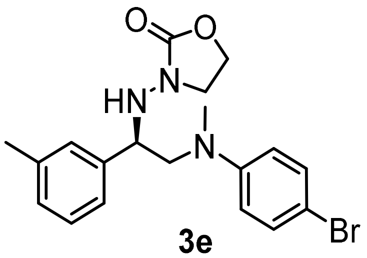

3e

A dried 10 mL Schlenk tube was charged with **1e** (40.8 mg, 0.20 mmol), **2a** (163.4 mg, 0.60 mmol), chiral copper catalyst **[L7-Cu^{II}]** (1.0 mL taken from the 20 mM solution in THF), and THF (1.0 mL). The mixture was degassed via three freeze-pump-thaw cycles. The Schlenk tube was positioned approximately 5 cm away from a 24 W blue LEDs lamp. After being stirred at -40 °C for 40 h, the reaction mixture was concentrated and then purified by flash chromatography on silica gel (eluted with PE:EtOAc = 2:1) to afford product **3e** as a white solid (63.1 mg, 0.157 mmol, yield: 78%). Enantiomeric excess was established by HPLC analysis using a Chiralpak AD-H column, e.e. = 89% (HPLC: AD-H, 220 nm, *n*-hexane/isopropanol = 90:10, flow rate: 1 mL/min, 30 °C, t_r(minor) = 12.3 min, t_r(major) = 15.1 min.) $[\alpha]_D^{23}$ = +29.9° (*c* = 1.0, CH_2Cl_2).

^{1}H NMR (500 MHz, $CDCl_3$) δ 7.35 – 7.28 (m, 2H), 7.25 (dd, *J* = 4.2, 1.3 Hz, 3H), 7.17 – 7.09 (m, 1H), 6.75 – 6.67 (m, 2H), 4.73 (d, *J* = 1.4 Hz, 1H), 4.54 (ddd, *J* = 9.5, 4.9, 1.5 Hz, 1H), 4.14 (td, *J* = 8.9, 4.9 Hz, 1H), 4.02 (q, *J* = 8.7 Hz, 1H), 3.61 (dd, *J* = 14.6, 9.6 Hz, 1H), 3.46 (dd, *J* = 17.1, 8.8 Hz, 1H), 3.23 – 3.11 (m, 2H), 2.91 (s, 3H), 2.37 (s, 3H).

^{13}C NMR (126 MHz, $CDCl_3$) δ 158.8, 149.3, 139.9, 138.4, 132.0, 129.0, 128.7, 128.6, 125.0, 115.2, 109.6, 61.9, 61.5, 58.8, 47.9, 38.4, 21.5.

IR (film): ν (cm^{-1}) 3482, 3276, 2962, 1751, 1670, 1590, 1497, 1448, 1411, 1259, 1097, 807, 705.

HRMS (ESI, m/z) calcd for $C_{19}H_{23}BrN_3O_2$ $(M+H)^+$: 404.0968, found: 404.0974.

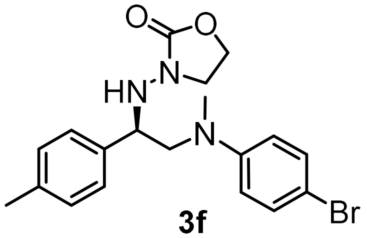

3f

A dried 10 mL Schlenk tube was charged with **1f** (40.8 mg, 0.20 mmol), **2a** (163.4 mg, 0.60 mmol), chiral copper catalyst **[L7-Cu^{II}]** (1.0 mL taken from the 20 mM solution in THF), and THF (1.0 mL). The mixture was degassed via three freeze-pump-thaw cycles. The Schlenk tube was positioned approximately 5 cm away from a 24 W blue LEDs lamp. After being stirred at -40 °C for 40 h, the reaction mixture was concentrated and then purified by flash chromatography on silica gel (eluted with PE:EtOAc = 2:1) to afford product **3f** as a white solid (65.9 mg, 0.164 mmol, yield: 82%). Enantiomeric excess was established by HPLC analysis using a Chiralpak IC column, e.e. = 87% (HPLC: IC, 220 nm, *n*-hexane/isopropanol = 90:10, flow rate: 1 mL/min, 30 °C, t_r(minor) = 39.2 min, t_r(major) = 41.8 min.)) $[\alpha]_D^{23}$ = +34.7° (*c* = 1.0, CH_2Cl_2).

^{1}H NMR (500 MHz, $CDCl_3$) δ 7.40 – 7.27 (m, 4H), 7.17 (d, *J* = 7.8 Hz, 2H), 6.74 – 6.65 (m, 2H), 4.72 (s, 1H), 4.54 (dd, *J* = 8.9, 5.1 Hz, 1H), 4.14 (td, *J* = 8.9, 4.9 Hz, 1H), 4.01 (q, *J* = 8.7 Hz, 1H), 3.60 (dd, *J* = 14.6, 9.5 Hz, 1H), 3.45 (dd, *J* = 17.1, 8.7 Hz, 1H), 3.16 (ddd, *J* = 13.2, 6.9, 3.0 Hz, 2H), 2.90 (s, 3H), 2.36 (s, 3H).

^{13}C NMR (126 MHz, $CDCl_3$) δ 158.8, 149.2, 138.0, 136.8, 132.0, 129.5, 127.9, 115.2, 109.6, 61.6, 61.5, 58.8, 47.9, 38.5, 21.3.

IR (film): ν (cm^{-1}) 3448, 2962, 2923, 2854, 1751, 1654, 1592, 1497, 1401, 1260, 1092, 1027, 803, 739, 701.

HRMS (ESI, m/z) calcd for $C_{19}H_{22}BrN_3O_2Na$ $(M+Na)^+$: 404.0968, found: 404.0969.

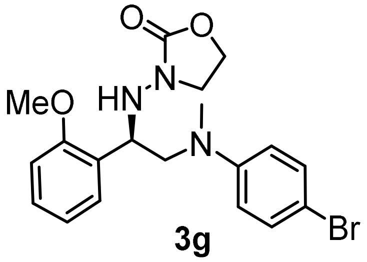

3g

A dried 10 mL Schlenk tube was charged with **1g** (44.0 mg, 0.20 mmol), **2a** (163.4 mg, 0.60 mmol), chiral copper catalyst **[L7-Cu^{II}]** (1.0 mL taken from the 20 mM solution in THF), and THF (1.0 mL). The mixture was degassed via three freeze-pump-thaw cycles. The Schlenk tube was positioned approximately 5 cm away from a 24 W blue LEDs lamp. After being stirred at -40 °C for 40 h, the reaction mixture was concentrated and then purified by flash chromatography on silica gel (eluted with PE:EtOAc = 2:1) to afford product **3g** as a white solid (60.2 mg, 0.144 mmol, yield: 72%). Enantiomeric excess was established by HPLC analysis using a Chiralpak AD-H column, e.e. = 90% (HPLC: AD-H, 220 nm, *n*-hexane/isopropanol = 90:10, flow rate: 1 mL/min, 30 °C, t_r(minor) = 20.6 min, t_r(major) = 23.9 min.) $[\alpha]_D^{23}$ = +18.6° (*c* = 1.0, CH_2Cl_2).

^{1}H NMR (500 MHz, $CDCl_3$) δ 7.58 (dd, *J* = 7.5, 1.5 Hz, 1H), 7.32 – 7.28 (m, 2H), 7.25 (d, *J* = 1.8 Hz, 1H), 6.98 (td, *J* = 7.5, 0.8 Hz, 1H), 6.89 (d, *J* = 7.7 Hz, 1H), 6.74 – 6.67 (m, 2H), 4.97 – 4.87 (m, 1H), 4.76 (d, *J* = 2.9 Hz, 1H), 4.18 – 4.02 (m, 2H), 3.85 (s, 3H), 3.49 (dt, *J* = 9.3, 8.3 Hz, 2H), 3.41 – 3.30 (m, 2H), 2.96 (s, 3H).

^{13}C NMR (126 MHz, $CDCl_3$) δ 158.7, 157.6, 149.0, 131.8, 128.8, 128.1, 127.9, 120.8, 114.8, 110.7, 109.0, 61.3, 56.9, 56.6, 55.5, 47.5, 38.8.

IR (film): ν (cm^{-1}) 3482, 3278, 3049, 2962, 2852, 1751, 1591, 1491, 1400, 1258, 1095, 1029, 808, 759.

HRMS (ESI, m/z) calcd for $C_{19}H_{22}BrN_3O_3Na$ $(M+Na)^+$: 442.0737, found: 442.0730.

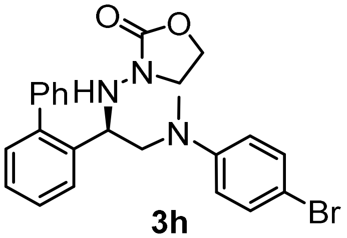

3h

A dried 10 mL Schlenk tube was charged with **1h** (53.3 mg, 0.20 mmol), **2a** (163.4 mg, 0.60 mmol), chiral copper catalyst **[L7-Cu^{II}]** (1.0 mL taken from the 20 mM solution in THF), and THF (1.0 mL). The mixture was degassed via three freeze-pump-thaw cycles. The Schlenk tube was positioned approximately 5 cm away from a 24 W blue LEDs lamp. After being stirred at -40 °C for 40 h, the reaction mixture was concentrated and then purified by flash chromatography on silica gel (eluted with PE:EtOAc = 2:1) to afford product **3h** as a white solid (64.8 mg, 0.139 mmol, yield: 70%). Enantiomeric excess was established by HPLC analysis using a Chiralpak AD-H column, e.e. = 93% (HPLC: AD-H, 220 nm, *n*-hexane/isopropanol = 90:10, flow rate: 1 mL/min, 30 °C, t_r(minor) = 12.6 min, t_r(major) = 20.3 min.) $[\alpha]_D^{23}$ = +48.0° (*c* = 1.0, CH_2Cl_2).

^{1}H NMR (500 MHz, $CDCl_3$) δ 7.85 (dd, J = 7.8, 1.1 Hz, 1H), 7.47 – 7.31 (m, 5H), 7.25 – 7.12 (m, 5H), 6.46 – 6.31 (m, 2H), 4.85 – 4.75 (m, 1H), 4.59 (d, J = 1.6 Hz, 1H), 4.22 – 4.03 (m, 2H), 3.49 – 3.26 (m, 3H), 3.11 (dd, J = 14.7, 4.7 Hz, 1H), 2.62 (s, 3H).

^{13}C NMR (126 MHz, $CDCl_3$) δ 158.7, 148.9, 143.1, 140.6, 137.3, 131.8, 130.2, 129.3, 128.5, 127.9, 127.5, 127.4, 127.1, 114.8, 109.4, 61.4, 58.3, 57.6, 47.8, 38.5.

IR (film): ν (cm^{-1}) 3448, 2962, 2917, 2849, 1751, 1591, 1497, 1400, 1262, 1090, 1031, 864, 805, 742, 703.

HRMS (ESI, m/z) calcd for $C_{24}H_{24}BrN_3O_2Na$ $(M+Na)^+$: 488.0944, found: 488.0952.

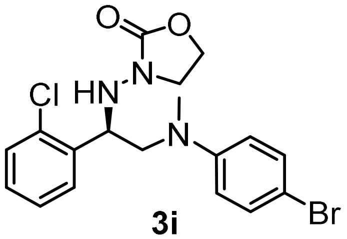

3i

A dried 10 mL Schlenk tube was charged with **1i** (44.9 mg, 0.20 mmol), **2a** (163.4 mg, 0.60 mmol), chiral copper catalyst **[L7-Cu^{II}]** (1.0 mL taken from the 20 mM solution in THF), and THF (1.0 mL). The mixture was degassed via three freeze-pump-thaw cycles. The Schlenk tube was positioned approximately 5 cm away from a 24 W blue LEDs lamp. After being stirred at -40 °C for 40 h, the reaction mixture was concentrated and then purified by flash chromatography on silica gel (eluted with PE:EtOAc = 2:1) to afford product **3i** as a white solid (66.7 mg, 0.158 mmol, yield: 79%). Enantiomeric excess was established by HPLC analysis using a Chiralpak AD-H column, e.e. = 90% (HPLC: AD-H, 220 nm, *n*-hexane/isopropanol = 90:10, flow rate: 1 mL/min, 30 °C, t_r(minor) = 16.8 min, t_r(major) = 27.3 min.) $[\alpha]_D^{23}$ = +55.3° (*c* = 1.0, CH_2Cl_2).

^{1}H NMR (500 MHz, $CDCl_3$) δ 7.88 – 7.76 (m, 1H), 7.40 – 7.28 (m, 4H), 7.27 – 7.22 (m, 1H), 6.80 – 6.69 (m, 2H), 5.12 (dd, *J* = 9.9, 3.1 Hz, 1H), 4.75 (d, *J* = 1.5 Hz, 1H), 4.20 – 4.02 (m, 2H), 3.48 (ddd, *J* = 24.7, 15.7, 9.4 Hz, 2H), 3.30 (ddd, *J* = 13.0, 6.5, 3.4 Hz, 2H), 3.02 (s, 3H).

^{13}C NMR (126 MHz, $CDCl_3$) δ 158.8, 149.2, 137.2, 134.0, 131.9, 129.9, 129.1, 128.6, 127.3, 115.3, 109.7, 61.4, 58.5, 57.0, 47.4, 38.2.

IR (film): ν (cm^{-1}) 3483, 3065, 2963, 2919, 2851, 1752, 1592, 1498, 1375, 1261, 1196, 1095, 1033, 807, 757.

HRMS (ESI, m/z) calcd for $C_{18}H_{19}BrClN_3O_2Na$ $(M+Na)^+$: 446.0241, found: 446.0238.

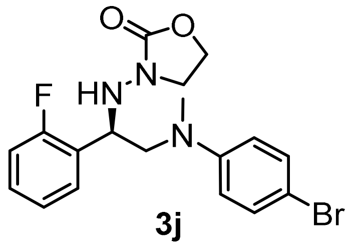

3j

A dried 10 mL Schlenk tube was charged with **1j** (41.6 mg, 0.20 mmol), **2a** (163.4 mg, 0.60 mmol), chiral copper catalyst **[L7-Cu^{II}]** (1.0 mL taken from the 20 mM solution in THF), and THF (1.0 mL). The mixture was degassed via three freeze-pump-thaw cycles. The Schlenk tube was positioned approximately 5 cm away from a 24 W blue LEDs lamp. After being stirred at -40 °C for 40 h, the reaction mixture was concentrated and then purified by flash chromatography on silica gel (eluted with PE:EtOAc = 2:1) to afford product **3j** as a white solid (56.4 mg, 0.139 mmol, yield: 69%). Enantiomeric excess was established by HPLC analysis using a Chiralpak IC column, e.e. = 91% (HPLC: IC, 220 nm, *n*-hexane/isopropanol = 90:10, flow rate: 1 mL/min, 30 °C, t_r(minor) = 36.2 min, t_r(major) = 38.4 min.) $[\alpha]_D^{23}$ = +64.4° (*c* = 1.0, CH_2Cl_2).

^{1}H NMR (500 MHz, $CDCl_3$) δ 7.64 (td, *J* = 7.5, 1.8 Hz, 1H), 7.35 – 7.27 (m, 3H), 7.18 (td, *J* = 7.5, 1.1 Hz, 1H), 7.11 – 7.03 (m, 1H), 6.77 – 6.65 (m, 2H), 4.92 (ddd, *J* = 9.7, 4.7, 2.2 Hz, 1H), 4.72 (d, *J* = 2.1 Hz, 1H), 4.16 (td, *J* = 8.8, 5.0 Hz, 1H), 4.07 (q, *J* = 8.6 Hz, 1H), 3.55 (ddd, *J* = 25.6, 15.8, 9.2 Hz, 2H), 3.36 – 3.23 (m, 2H), 2.97 (s, 3H).

^{13}C NMR (126 MHz, $CDCl_3$) δ 160.4, 158.8, 149.0, 132.0, 129.6 (d, *J* = 8.3 Hz), 128.9 (d, *J* = 4.3 Hz), 126.8 (d, *J* = 12.6 Hz), 124.5 (d, *J* = 3.4 Hz), 115.8 (d, *J* = 22.0 Hz), 115.1, 109.7, 61.4, 57.2, 55.8, 47.6, 38.5.

IR (film): ν (cm^{-1}) 3448, 2963, 2923, 2854, 1752, 1590, 1497, 1453, 1261, 1093, 1027, 865, 803, 762.

HRMS (ESI, m/z) calcd for $C_{18}H_{19}FBrN_3O_2Na$ $(M+Na)^+$: 430.0537, found: 430.0541.

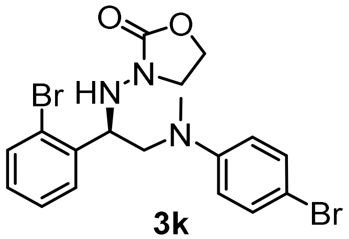

3k

A dried 10 mL Schlenk tube was charged with **1k** (53.8 mg, 0.20 mmol), **2a** (163.4 mg, 0.60 mmol), chiral copper catalyst **[L7-Cu^{II}]** (1.0 mL taken from the 20 mM solution in THF), and THF (1.0 mL). The mixture was degassed via three freeze-pump-thaw cycles. The Schlenk tube was positioned approximately 5 cm away from a 24 W blue LEDs lamp. After being stirred at -40 °C for 40 h, the reaction mixture was concentrated and then purified by flash chromatography on silica gel (eluted with PE:EtOAc = 2:1) to afford product **3k** as a white solid (69.4 mg, 0.148 mmol, yield: 74%). Enantiomeric excess was established by HPLC analysis using a Chiralpak AD-H column, e.e. = 93% (HPLC: AD-H, 220 nm, *n*-hexane/isopropanol = 90:10, flow rate: 1 mL/min, 30 °C, t_r(minor) = 17.6 min, t_r(major) = 29.3 min.) $[\alpha]_D^{23}$ = +27.1° (*c* = 1.0, CH_2Cl_2).

^{1}H NMR (500 MHz, $CDCl_3$) δ 7.81 (d, *J* = 7.5 Hz, 1H), 7.56 (dd, *J* = 8.0, 1.2 Hz, 1H), 7.42 – 7.35 (m, 1H), 7.34 – 7.28 (m, 2H), 7.22 – 7.14 (m, 1H), 6.79 – 6.70 (m, 2H), 5.09 (dd, *J* = 9.7, 3.4 Hz, 1H), 4.76 (d, *J* = 1.3 Hz, 1H), 4.20 – 4.01 (m, 2H), 3.52 (dd, *J* = 16.9, 8.9 Hz, 1H), 3.42 (dd, *J* = 14.4, 10.2 Hz, 1H), 3.28 (ddd, *J* = 6.4, 5.9, 3.1 Hz, 2H), 3.03 (s, 3H).

^{13}C NMR (126 MHz, $CDCl_3$) δ 158.7, 149.2, 138.7, 133.1, 131.9, 129.4, 128.9, 127.9, 124.3, 115.3, 109.7, 61.3, 60.9, 57.0, 47.4, 38.1.

IR (film): ν (cm^{-1}) 3422, 2962, 2924, 2853, 1752, 1707, 1591, 1497, 1400, 1366, 1261, 1165, 1096, 1025, 807, 738, 699.

HRMS (ESI, m/z) calcd for $C_{18}H_{20}Br_2N_3O_2$ $(M+H)^+$:469.9896, found:469.9900.

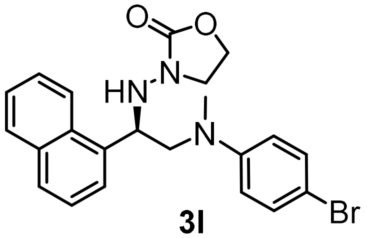

3l

A dried 10 mL Schlenk tube was charged with **1l** (48.1 mg, 0.20 mmol), **2a** (163.4 mg, 0.60 mmol), chiral copper catalyst **[L7-Cu^{II}]** (1.0 mL taken from the 20 mM solution in THF), and THF (1.0 mL). The mixture was degassed via three freeze-pump-thaw cycles. The Schlenk tube was positioned approximately 5 cm away from a 24 W blue LEDs lamp. After being stirred at -40 °C for 40 h, the reaction mixture was concentrated and then purified by flash chromatography on silica gel (eluted with PE:EtOAc = 2:1) to afford product **3l** as a white solid (58.2 mg, 0.133 mmol, yield: 66%). Enantiomeric excess was established by HPLC analysis using a Chiralpak OD-H column, e.e. = 91% (HPLC: OD-H, 220 nm, *n*-hexane/isopropanol = 90:10, flow rate: 1 mL/min, 30 °C, t_r(minor) = 19.6 min, t_r(major) = 24.0 min.) $[\alpha]_D^{23}$ = +8.3° (*c* = 1.0, CH_2Cl_2).

^{1}H NMR (500 MHz, $CDCl_3$) δ 8.48 – 7.68 (m, 4H), 7.65 – 7.40 (m, 3H), 7.39 – 7.28 (m, 2H), 6.73 (d, *J* = 8.8 Hz, 2H), 5.56 (s, 1H), 4.93 (s, 1H), 4.10 (td, *J* = 8.8, 4.7 Hz, 1H), 3.91 (q, *J* = 8.7 Hz, 1H), 3.45 (dd, *J* = 111.3, 72.1 Hz, 4H), 2.98 (s, 3H).

^{13}C NMR (126 MHz, $CDCl_3$) δ 159.0, 149.2, 134.0, 132.0, 131.9, 129.1, 128.4, 126.5, 125.9, 125.5, 124.5, 122.3, 122.1, 115.2, 109.7, 61.5, 58.3, 47.9, 38.4, 29.8.

IR (film): ν (cm^{-1}) 3483, 3275, 3049, 2963, 2922, 1751, 1591, 1497, 1397, 1261, 1092, 1029, 862, 803, 736, 702.

HRMS (ESI, m/z) calcd for $C_{22}H_{23}BrN_3O_2$ $(M+H)^+$: 440.0968, found: 440.0979.

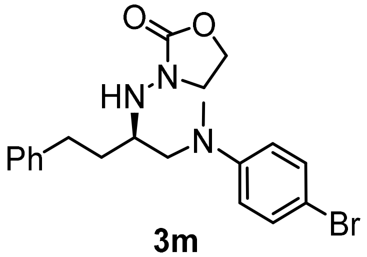

3m

A dried 10 mL Schlenk tube was charged with **1m** (43.7 mg, 0.20 mmol), **2a** (163.4 mg, 0.60 mmol), chiral copper catalyst **[L7-Cu^{II}]** (1.0 mL taken from the 20 mM solution in THF), and THF (1.0 mL). The mixture was degassed via three freeze-pump-thaw cycles. The Schlenk tube was positioned approximately 5 cm away from a 24 W blue LEDs lamp. After being stirred at 25 °C for 40 h, the reaction mixture was concentrated and then purified by flash chromatography on silica gel (eluted with PE:EtOAc = 2:1) to afford product **3m** as a white solid (44.0 mg, 0.106 mmol, yield: 53%). Enantiomeric excess was established by HPLC analysis using a Chiralpak IC column, e.e. = 19% (HPLC: IC, 220 nm, *n*-hexane/isopropanol = 90:10, flow rate: 1 mL/min, 30 °C, t_r(minor) = 49.7 min, t_r(major) = 53.3 min.) $[\alpha]_D^{23}$ = +11.8° (*c* = 1.0, CH_2Cl_2).

^{1}H NMR (500 MHz, $CDCl_3$) δ 7.33 – 7.26 (m, 4H), 7.24 – 7.14 (m, 3H), 6.68 – 6.58 (m, 2H), 4.33 (d, *J* = 2.3 Hz, 1H), 4.25 (t, *J* = 7.9 Hz, 2H), 3.60 – 3.48 (m, 2H), 3.48 – 3.33 (m, 2H), 3.22 (dd, *J* = 13.6, 4.6 Hz, 1H), 2.91 (s, 3H), 2.84 – 2.65 (m, 2H), 1.92 – 1.74 (m, 2H).

^{13}C NMR (126 MHz, $CDCl_3$) δ 159.3, 149.3, 141.8, 132.0, 128.7, 128.4, 126.2, 115.0, 109.4, 61.4, 56.8, 56.5, 48.6, 39.0, 33.2, 31.8.

IR (film): ν (cm^{-1}) 3371, 3027, 2915, 2853, 1752, 1602, 1507, 1406, 1321, 1258, 1096, 1030, 748, 704.

HRMS (ESI, m/z) calcd for $C_{20}H_{24}BrN_3O_2Na$ $(M+Na)^+$: 440.0944, found: 440.0956.

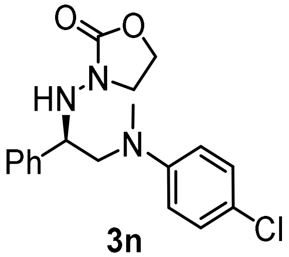

3n

A dried 10 mL Schlenk tube was charged with **1b** (38.0 mg, 0.20 mmol), **2b** (136.7 mg, 0.60 mmol), chiral copper catalyst **[L7-Cu^{II}]** (1.0 mL taken from the 20 mM solution in THF), and THF (1.0 mL). The mixture was degassed via three freeze-pump-thaw cycles. The Schlenk tube was positioned approximately 5 cm away from a 24 W blue LEDs lamp. After being stirred at -40 °C for 40 h, the reaction mixture was concentrated and then purified by flash chromatography on silica gel (eluted with PE:EtOAc = 2:1) to afford product **3n** as a white solid (45.6 mg, 0.132 mmol, yield: 66%). Enantiomeric excess was established by HPLC analysis using a Chiralpak IC column, e.e. = 95% (HPLC: IC, 220 nm, *n*-hexane/isopropanol = 95:5, flow rate: 1 mL/min, 30 °C, t_r(minor) = 31.1 min, t_r(major) = 33.2 min.) $[\alpha]_D^{23}$ = +82.5° (*c* = 1.0, CH_2Cl_2).

^{1}H NMR (500 MHz, $CDCl_3$) δ 7.45 (dt, *J* = 3.3, 2.0 Hz, 2H), 7.41 – 7.28 (m, 3H), 7.21 – 7.14 (m, 2H), 6.79 – 6.72 (m, 2H), 4.78 (d, *J* = 1.4 Hz, 1H), 4.59 (ddd, *J* = 9.4, 5.0, 1.4 Hz, 1H), 4.14 (td, *J* = 8.9, 4.9 Hz, 1H), 4.00 (q, *J* = 8.7 Hz, 1H), 3.62 (dd, *J* = 14.6, 9.5 Hz, 1H), 3.46 (dd, *J* = 17.0, 8.9 Hz, 1H), 3.24 – 3.10 (m, 2H), 2.91 (s, 3H).

^{13}C NMR (126 MHz, $CDCl_3$) δ 158.8, 148.9, 140.0, 129.1, 128.7, 128.3, 128.0, 122.5, 114.8, 61.9, 61.4, 58.9, 47.9, 38.5.

IR (film): ν (cm^{-1}) 3483, 3275, 3030, 2923, 1751, 1678, 1596, 1496, 1405, 1260, 1092, 1027, 974, 943, 806, 756, 700.

HRMS (ESI, m/z) calcd for $C_{18}H_{21}ClN_3O_2$ $(M+H)^+$: 346.1317, found: 346.1321.

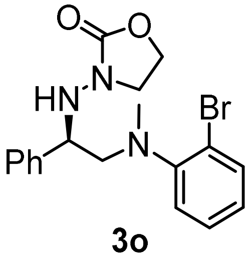

3o

A dried 10 mL Schlenk tube was charged with **1b** (38.0 mg, 0.20 mmol), **2c** (163.4 mg, 0.60 mmol), chiral copper catalyst **[L8-Cu^{II}]** (2.0 mL taken from the 20 mM solution in THF). The mixture was degassed via three freeze-pump-thaw cycles. The Schlenk tube was positioned approximately 5 cm away from a 24 W blue LEDs lamp. After being stirred at -40 °C for 40 h, the reaction mixture was concentrated and then purified by flash chromatography on silica gel (eluted with PE:EtOAc = 2:1) to afford product **3o** as a white solid (49.9 mg, 0.128 mmol, yield: 64%). Enantiomeric excess was established by HPLC analysis using a Chiralpak IC column, e.e. = 91% (HPLC: IC, 220 nm, *n*-hexane/isopropanol = 90:10, flow rate: 1 mL/min, 30 °C, t_r(minor) = 36.5 min, t_r(major) = 49.0 min.) $[\alpha]_D^{23}$ = +99.8° (*c* = 1.0, CH_2Cl_2).

^1H NMR (500 MHz, $CDCl_3$) δ 7.59 (dd, *J* = 7.9, 1.1 Hz, 1H), 7.46 (d, *J* = 7.2 Hz, 2H), 7.36 (t, *J* = 7.3 Hz, 2H), 7.33 – 7.24 (m, 2H), 7.13 (dd, *J* = 7.9, 1.0 Hz, 1H), 7.00 – 6.93 (m, 1H), 5.56 (s, 1H), 4.50 (dd, *J* = 11.2, 3.2 Hz, 1H), 4.13 (td, *J* = 8.8, 5.1 Hz, 1H), 4.00 (q, *J* = 8.7 Hz, 1H), 3.48 (q, *J* = 8.6 Hz, 1H), 3.23 – 3.04 (m, 3H), 2.93 (s, 3H).

^{13}C NMR (126 MHz, $CDCl_3$) δ 158.5, 151.3, 140.5, 133.8, 128.6, 128.4, 128.0, 127.9, 125.5, 123.2, 121.7, 61.3, 61.0, 60.5, 47.5, 42.2.

IR (film): ν (cm^{-1}) 3482, 3263, 3060, 2962, 2801, 1751, 1586, 1474, 1401, 1260, 1091, 1026, 941, 861, 799, 754, 702.

HRMS (ESI, m/z) calcd for $C_{18}H_{20}BrN_3O_2Na$ $(M+Na)^+$: 412.0631, found: 412.0633.

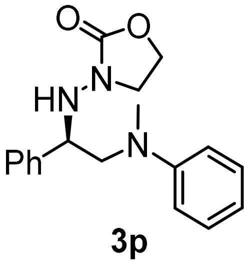

3p

A dried 10 mL Schlenk tube was charged with **1b** (38.0 mg, 0.20 mmol), **2d** (116.0 mg, 0.60 mmol) and chiral copper catalyst **[L7-Cu^{II}]** (2.0 mL taken from the 20 mM solution in THF). The mixture was degassed via three freeze-pump-thaw cycles. The Schlenk tube was positioned approximately 5 cm away from a 24 W blue LEDs lamp. After being stirred at -40 °C for 40 h, the reaction mixture was concentrated and then purified by flash chromatography on silica gel (eluted with PE:EtOAc = 2:1) to afford product **3p** as a white solid (36.2 mg, 0.116 mmol, yield: 58%). Enantiomeric excess was established by HPLC analysis using a Chiralpak AD-H column, e.e. = 81% (HPLC: AD-H, 220 nm, *n*-hexane/isopropanol = 90:10, flow rate: 1 mL/min, 30 °C, t_r(minor) = 12.6 min, t_r(major) = 15.2 min.) $[\alpha]_D^{23}$ = +27.9° (*c* = 1.0, CH_2Cl_2).

^{1}H NMR (500 MHz, $CDCl_3$) δ 7.47 (dt, *J* = 3.2, 2.0 Hz, 2H), 7.41 – 7.34 (m, 2H), 7.34 – 7.29 (m, 1H), 7.28 – 7.26 (m, 1H), 7.25 – 7.23 (m, 1H), 6.86 (dt, *J* = 3.4, 1.8 Hz, 2H), 6.80 – 6.73 (m, 1H), 4.85 (s, 1H), 4.62 (dd, *J* = 9.7, 4.9 Hz, 1H), 4.13 (td, *J* = 8.9, 4.9 Hz, 1H), 4.01 (q, *J* = 8.7 Hz, 1H), 3.66 (dd, *J* = 14.5, 9.8 Hz, 1H), 3.47 (dd, *J* = 17.0, 8.8 Hz, 1H), 3.17 (ddd, *J* = 13.2, 11.4, 4.9 Hz, 2H), 2.95 (s, 3H).

^{13}C NMR (126 MHz, $CDCl_3$) δ 158.7, 150.5, 140.3, 129.3, 128.8, 128.2, 128.1, 117.8, 113.9, 62.0, 61.5, 59.0, 47.9, 38.4.

IR (film): ν (cm^{-1}) 3448, 2963, 2922, 1763, 1671, 1598, 1406, 1261, 1209, 1092, 1035, 800, 754, 696.

HRMS (ESI, m/z) calcd for $C_{18}H_{22}N_3O_2$ $(M+H)^+$: 312.1707, found: 312.1706.

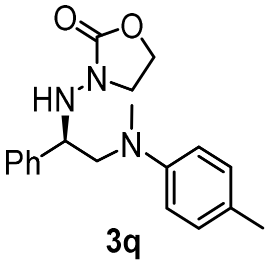

3q

A dried 10 mL Schlenk tube was charged with **1b** (38.0 mg, 0.20 mmol), **2e** (124.4 mg, 0.60 mmol) and chiral copper catalyst **[L7-Cu^{II}]** (2.0 mL taken from the 20 mM solution in THF). The mixture was degassed via three freeze-pump-thaw cycles. The Schlenk tube was positioned approximately 5 cm away from a 24 W blue LEDs lamp. After being stirred at -40 °C for 40 h, the reaction mixture was concentrated and then purified by flash chromatography on silica gel (eluted with PE:EtOAc = 2:1) to afford product **3q** as a white solid (36.0 mg, 0.111 mmol, yield: 55%). Enantiomeric excess was established by HPLC analysis using a Chiralpak AD-H column, e.e. = 86% (HPLC: AD-H, 220 nm, *n*-hexane/isopropanol = 90:10, flow rate: 1 mL/min, 30 °C, t_r(minor) = 14.4 min, t_r(major) = 22.4 min.) $[\alpha]_D^{23}$ = +76.2° (*c* = 1.0, CH_2Cl_2).

^{1}H NMR (500 MHz, $CDCl_3$) δ 7.47 (d, *J* = 7.2 Hz, 2H), 7.37 (t, *J* = 7.3 Hz, 2H), 7.31 (t, *J* = 7.2 Hz, 1H), 7.06 (d, *J* = 8.3 Hz, 2H), 6.80 (d, *J* = 8.5 Hz, 2H), 4.92 (s, 1H), 4.60 (dd, *J* = 9.9, 4.5 Hz, 1H), 4.13 (td, *J* = 8.8, 4.9 Hz, 1H), 4.00 (q, *J* = 8.7 Hz, 1H), 3.59 (dd, *J* = 14.3, 10.0 Hz, 1H), 3.47 (q, *J* = 8.7 Hz, 1H), 3.20 – 3.06 (m, 2H), 2.93 (s, 3H), 2.26 (s, 3H).

^{13}C NMR (126 MHz, $CDCl_3$) δ 158.7, 148.6, 140.4, 129.8, 128.7, 128.1, 128.0, 127.4, 114.5, 61.9, 61.4, 59.6, 47.9, 38.6, 20.4.

IR (film): ν (cm^{-1}) 3483, 3277, 2963, 1752, 1618, 1522, 1400, 1261, 1093, 1026, 800, 701.

HRMS (ESI, m/z) calcd for $C_{19}H_{24}N_3O_2$ $(M+H)^+$:326.1863, found:326.1866.

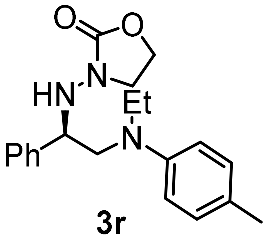

3r

A dried 10 mL Schlenk tube was charged with **1b** (38.0 mg, 0.20 mmol), **2f** (132.9 mg, 0.60 mmol), chiral copper catalyst **[L7-Cu^{II}]** (1.0 mL taken from the 20 mM solution in THF), and THF (1.0 mL). The mixture was degassed via three freeze-pump-thaw cycles. The Schlenk tube was positioned approximately 5 cm away from a 24 W blue LEDs lamp. After being stirred at -40 °C for 40 h, the reaction mixture was concentrated and then purified by flash chromatography on silica gel (eluted with PE:EtOAc = 2:1) to afford product **3r** as a white solid (40.0 mg, 0.118 mmol, yield: 59%). Enantiomeric excess was established by HPLC analysis using a Chiralpak AD-H column, e.e. = 74% (HPLC: AD-H, 220 nm, *n*-hexane/isopropanol = 95:5, flow rate: 1 mL/min, 30 °C, t_r(minor) = 11.4 min, t_r(major) = 14.1 min.) $[\alpha]_D^{23}$ = +14.9° (*c* = 1.0, CH_2Cl_2).

^{1}H NMR (500 MHz, $CDCl_3$) δ 7.51 – 7.44 (m, 2H), 7.40 – 7.34 (m, 2H), 7.33 – 7.28 (m, 1H), 7.06 (d, *J* = 8.2 Hz, 2H), 6.82 (d, *J* = 8.6 Hz, 2H), 4.93 (s, 1H), 4.54 (dd, *J* = 9.8, 4.3 Hz, 1H), 4.11 (td, *J* = 8.9, 4.9 Hz, 1H), 3.99 (q, *J* = 8.7 Hz, 1H), 3.52 – 3.41 (m, 2H), 3.38 (qd, *J* = 7.2, 3.1 Hz, 2H), 3.27 (dd, *J* = 14.5, 4.6 Hz, 1H), 3.12 (td, *J* = 8.4, 4.9 Hz, 1H), 2.26 (s, 3H), 1.09 (t, *J* = 7.0 Hz, 3H).

^{13}C NMR (126 MHz, $CDCl_3$) δ 158.6, 146.8, 140.5, 129.9, 128.7, 128.1, 128.0, 127.4, 115.4, 61.9, 61.4, 56.8, 47.8, 45.5, 20.4, 11.3.

IR (film): ν (cm^{-1}) 3359, 2919, 2850, 1752, 1617, 1519, 1401, 1242, 1088, 1028, 803, 760, 703.

HRMS (ESI, m/z) calcd for $C_{20}H_{25}N_3O_2Na$ $(M+Na)^+$: 362.1839, found: 362.1843.

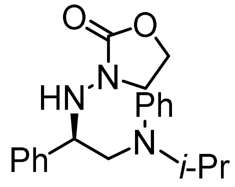

3s

A dried 10 mL Schlenk tube was charged with **1b** (38.0 mg, 0.20 mmol), **2g** (132.9 mg, 0.60 mmol), chiral copper catalyst **[L7-Cu^{II}]** (1.0 mL taken from the 20 mM solution in THF), and THF (1.0 mL). The mixture was degassed via three freeze-pump-thaw cycles. The Schlenk tube was positioned approximately 5 cm away from a 24 W blue LEDs lamp. After being stirred at -40 °C for 40 h, the reaction mixture was concentrated and then purified by flash chromatography on silica gel (eluted with PE:EtOAc = 2:1) to afford product **3s** as a white solid (43.4 mg, 0.128 mmol, yield: 64%). Enantiomeric excess was established by HPLC analysis using a Chiralpak AD-H column, e.e. = 67% (HPLC: AD-H, 220 nm, *n*-hexane/isopropanol = 90:10, flow rate: 1 mL/min, 30 °C, t_r(minor) = 15.3 min, t_r(major) = 13.1 min.) $[\alpha]_D^{23}$ = +22.3° (*c* = 1.0, CH_2Cl_2).

^{1}H NMR (500 MHz, $CDCl_3$) δ 7.50 – 7.43 (m, 2H), 7.42 – 7.33 (m, 2H), 7.33 – 7.26 (m, 3H), 7.08 (d, *J* = 7.8 Hz, 2H), 6.91 (t, *J* = 7.3 Hz, 1H), 5.02 (s, 1H), 4.33 (dd, *J* = 10.1, 4.3 Hz, 1H), 4.08 (td, *J* = 8.9, 4.8 Hz, 1H), 3.96 (q, *J* = 8.7 Hz, 1H), 3.85 (dt, *J* = 13.3, 6.6 Hz, 1H), 3.41 (q, *J* = 8.8 Hz, 1H), 3.31 (dd, *J* = 14.4, 4.3 Hz, 1H), 3.19 (dd, *J* = 14.4, 10.1 Hz, 1H), 3.03 (td, *J* = 8.4, 4.8 Hz, 1H), 1.22 (d, *J* = 6.6 Hz, 3H), 1.05 (d, *J* = 6.6 Hz, 3H).

^{13}C NMR (126 MHz, $CDCl_3$) δ 158.4, 149.3, 141.0, 129.0, 128.6, 128.0, 127.9, 120.3, 119.7, 62.2, 61.2, 52.7, 51.3, 47.5, 20.3, 19.5.

IR (film): ν (cm^{-1}) 3366, 2963, 2920, 1752, 1597, 1496, 1400, 1261, 1089, 1030, 802, 702.

HRMS (ESI, m/z) calcd for $C_{20}H_{25}N_3O_2Na$ $(M+Na)^+$: 362.1839, found: 362.1838.

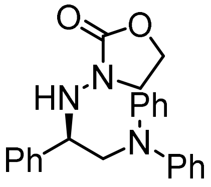

3t

A dried 10 mL Schlenk tube was charged with **1b** (38.0 mg, 0.20 mmol), **2h** (153.3 mg, 0.60 mmol), chiral copper catalyst **[L7-Cu^{II}]** (1.0 mL taken from the 20 mM solution in THF), and THF (1.0 mL). The mixture was degassed via three freeze-pump-thaw cycles. The Schlenk tube was positioned approximately 5 cm away from a 24 W blue LEDs lamp. After being stirred at -40 °C for 40 h, the reaction mixture was concentrated and then purified by flash chromatography on silica gel (eluted with PE:EtOAc = 2:1) to afford product **3t** as a white solid (46.3 mg, 0.124 mmol, yield: 62%). Enantiomeric excess was established by HPLC analysis using a Chiralpak AD-H column, e.e. = 54% (HPLC: AD-H, 220 nm, *n*-hexane/isopropanol = 95:5, flow rate: 1 mL/min, 30 °C, t_r(minor) = 27.0 min, t_r(major) = 21.9 min.) $[\alpha]_D^{23}$ = +25.1° (*c* = 1.0, CH_2Cl_2).

^{1}H NMR (500 MHz, $CDCl_3$) δ 7.43 (dd, *J* = 8.1, 1.4 Hz, 2H), 7.39 – 7.24 (m, 7H), 7.10 – 7.03 (m, 4H), 7.03 – 6.96 (m, 2H), 4.92 (d, *J* = 1.7 Hz, 1H), 4.53 (ddd, *J* = 9.4, 4.3, 1.7 Hz, 1H), 4.16 – 3.89 (m, 3H), 3.80 (dd, *J* = 14.9, 4.4 Hz, 1H), 3.43 (q, *J* = 8.9 Hz, 1H), 3.06 (td, *J* = 8.4, 4.7 Hz, 1H).

^{13}C NMR (126 MHz, $CDCl_3$) δ 158.7, 148.3, 139.9, 129.6, 128.7, 128.2, 128.1, 122.2, 121.7, 62.9, 61.4, 58.0, 47.8.

IR (film): ν (cm^{-1}) 3367, 2962, 2919, 2849, 1752, 1654, 1588, 1494, 1420, 1260, 1091, 1029, 802, 751, 700.

HRMS (ESI, m/z) calcd for $C_{23}H_{23}N_3O_2Na$ $(M+Na)^+$: 396.1682, found: 396.1680.

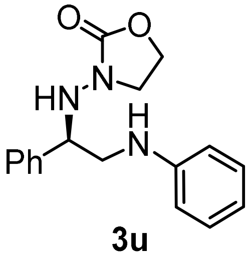

3u

A dried 10 mL Schlenk tube was charged with **1b** (38.0 mg, 0.20 mmol), **2i** (107.6 mg, 0.60 mmol), chiral copper catalyst **[L7-Cu^{II}]** (1.0 mL taken from the 20 mM solution in THF), and THF (1.0 mL). The mixture was degassed via three freeze-pump-thaw cycles. The Schlenk tube was positioned approximately 5 cm away from a 24 W blue LEDs lamp. After being stirred at -40 °C for 40 h, the reaction mixture was concentrated and then purified by flash chromatography on silica gel (eluted with PE:EtOAc = 2:1) to afford product **3u** as a white solid (39.0 mg, 0.131 mmol, yield: 66%). Enantiomeric excess was established by HPLC analysis using a Chiralpak OD-H column, e.e. = 95% (HPLC: OD-H, 220 nm, *n*-hexane/isopropanol = 90:10, flow rate: 1 mL/min, 30 °C, t_r(minor) = 28.5 min, t_r(major) = 17.6 min.) $[\alpha]_D^{23}$ = +27.6° (*c* = 1.0, CH_2Cl_2).

^{1}H NMR (500 MHz, $CDCl_3$) δ 7.58 – 7.50 (m, 2H), 7.42 – 7.32 (m, 3H), 7.26 – 7.21 (m, 2H), 6.76 (t, *J* = 7.3 Hz, 1H), 6.49 (d, *J* = 7.8 Hz, 2H), 5.05 (dd, *J* = 9.3, 6.9 Hz, 1H), 4.75 (d, *J* = 3.0 Hz, 1H), 4.66 (d, *J* = 3.0 Hz, 1H), 4.22 (td, *J* = 9.0, 7.2 Hz, 1H), 4.15 (td, *J* = 9.0, 6.5 Hz, 1H), 3.80 (dd, *J* = 8.6, 7.0 Hz, 1H), 3.62 (ddd, *J* = 9.1, 8.1, 6.5 Hz, 1H), 3.40 – 3.28 (m, 2H).

^{13}C NMR (126 MHz, $CDCl_3$) δ 158.9, 147.8, 139.7, 129.2, 128.6, 128.0, 127.6, 117.6, 113.0, 62.7, 61.3, 48.3, 47.4.

IR (film): ν (cm^{-1}) 3374, 2963, 2922, 1753, 1602, 1508, 1406, 1207, 1096, 1028, 799, 753, 694.

HRMS (ESI, m/z) calcd for $C_{17}H_{19}N_3O_2Na$ $(M+Na)^+$: 320.1369, found: 320.1374.

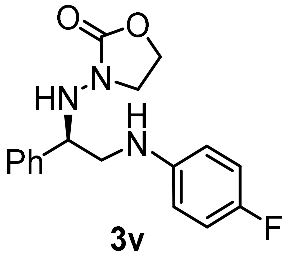

3v

A dried 10 mL Schlenk tube was charged with **1b** (38.0 mg, 0.20 mmol), **2j** (118.4 mg, 0.60 mmol), chiral copper catalyst **[L7-Cu^{II}]** (1.0 mL taken from the 20 mM solution in THF), and THF (1.0 mL). The mixture was degassed via three freeze-pump-thaw cycles. The Schlenk tube was positioned approximately 5 cm away from a 24 W blue LEDs lamp. After being stirred at -40 °C for 40 h, the reaction mixture was concentrated and then purified by flash chromatography on silica gel (eluted with PE:EtOAc = 2:1) to afford product **3v** as a white solid (41.1 mg, 0.130 mmol, yield: 65%). Enantiomeric excess was established by HPLC analysis using a Chiralpak IC column, e.e. = 95% (HPLC: IC, 220 nm, *n*-hexane/isopropanol = 80:20, flow rate: 1 mL/min, 30 °C, t_r(minor) = 34.5 min, t_r(major) = 40.4 min.) $[\alpha]_D^{23}$ = +9.3° (*c* = 1.0, CH_2Cl_2).

^{1}H NMR (500 MHz, $CDCl_3$) δ 7.53 (dt, *J* = 3.8, 2.1 Hz, 2H), 7.46 – 7.29 (m, 3H), 7.07 – 6.86 (m, 2H), 6.51 – 6.29 (m, 2H), 5.05 (dd, *J* = 9.3, 6.9 Hz, 1H), 4.73 (d, *J* = 2.9 Hz, 1H), 4.60 (d, *J* = 2.9 Hz, 1H), 4.26 – 4.10 (m, 2H), 3.76 (dd, *J* = 8.4, 6.9 Hz, 1H), 3.62 (ddd, *J* = 9.1, 8.0, 6.5 Hz, 1H), 3.40 – 3.27 (m, 2H).

^{13}C NMR (126 MHz, $CDCl_3$) δ 159.2, 155.2, 144.3, 139.8, 128.8, 128.4, 127.8, 115.8 (d, *J* = 22.3 Hz), 114.2 (d, *J* = 7.3 Hz), 63.2, 61.6, 49.2, 47.9.

IR (film): ν (cm^{-1}) 3448, 2963, 2921, 2851, 1752, 1654, 1518, 1410, 1261, 1225, 1096, 1031, 799, 702

HRMS (ESI, m/z) calcd for $C_{17}H_{18}N_3O_2Na$ $(M+Na)^+$: 338.1275, found: 338.1279.

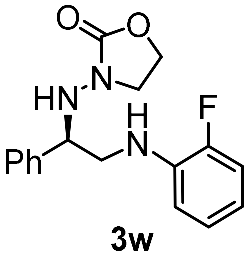

3w

A dried 10 mL Schlenk tube was charged with **1b** (38.0 mg, 0.20 mmol), **2k** (118.4 mg, 0.60 mmol), chiral copper catalyst **[L7-Cu^{II}]** (1.0 mL taken from the 20 mM solution in THF), and THF (1.0 mL). The mixture was degassed via three freeze-pump-thaw cycles. The Schlenk tube was positioned approximately 5 cm away from a 24 W blue LEDs lamp. After being stirred at -40 °C for 40 h, the reaction mixture was concentrated and then purified by flash chromatography on silica gel (eluted with PE:EtOAc = 2:1) to afford product **3w** as a white solid (40.5 mg, 0.129 mmol, yield: 64%). Enantiomeric excess was established by HPLC analysis using a Chiralpak AD-H column, e.e. = 94% (HPLC: AD-H, 220 nm, *n*-hexane/isopropanol = 90:10, flow rate: 1 mL/min, 30 °C, t_r(minor) = 28.4 min, t_r(major) = 24.2 min.) $[\alpha]_D^{23}$ = +45.0° (*c* = +1.0, CH_2Cl_2).

^{1}H NMR (500 MHz, $CDCl_3$) δ 7.61 – 7.49 (m, 2H), 7.46 – 7.28 (m, 3H), 7.07 – 6.93 (m, 2H), 6.80 – 6.71 (m, 1H), 6.62 (dd, *J* = 12.5, 4.6 Hz, 1H), 4.95 (dd, *J* = 9.3, 6.7 Hz, 1H), 4.90 (dd, *J* = 3.9, 3.1 Hz, 1H), 4.70 – 4.64 (m, 1H), 4.28 – 4.12 (m, 2H), 3.85 – 3.77 (m, 1H), 3.67 – 3.58 (m, 1H), 3.49 – 3.38 (m, 2H).

^{13}C NMR (126 MHz, $CDCl_3$) δ 159.1, 150.8, 139.5, 136.4 (d, *J* = 11.5 Hz), 128.8, 128.4, 127.9, 124.7 (d, *J* = 3.4 Hz), 117.3 (d, *J* = 7.0 Hz), 114.7 (d, *J* = 18.5 Hz), 112.5 (d, *J* = 3.1 Hz), 62.9, 61.6, 48.1, 48.0.

IR (film): ν (cm^{-1}) 3448, 2962, 2925, 2854, 1752, 1654, 1612, 1518, 1411, 1261, 1101,814, 703.

HRMS (ESI, m/z) calcd for $C_{17}H_{18}FN_3O_2Na$ $(M+Na)^+$: 338.1275, found: 338.1279.

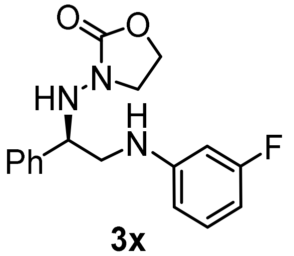

3x

A dried 10 mL Schlenk tube was charged with **1b** (38.0 mg, 0.20 mmol), **2l** (118.4 mg, 0.60 mmol), chiral copper catalyst **[L7-Cu^{II}]** (1.0 mL taken from the 20 mM solution in THF), and THF (1.0 mL). The mixture was degassed via three freeze-pump-thaw cycles. The Schlenk tube was positioned approximately 5 cm away from a 24 W blue LEDs lamp. After being stirred at -40 °C for 40 h, the reaction mixture was concentrated and then purified by flash chromatography on silica gel (eluted with PE:EtOAc = 2:1) to afford product **3x** as a white solid (44.0 mg, 0.140 mmol, yield: 70%). Enantiomeric excess was established by HPLC analysis using a Chiralpak AD-H column, e.e. = 96% (HPLC: AD-H, 220 nm, *n*-hexane/isopropanol = 90:10, flow rate: 1 mL/min, 30 °C, t_r(minor) = 35.3 min, t_r(major) = 26.8 min.) $[\alpha]_D^{23}$ = +41.4° (*c* = 1.0, CH_2Cl_2).

^{1}H NMR (500 MHz, $CDCl_3$) δ 7.44 (dd, *J* = 8.1, 1.4 Hz, 2H), 7.41 – 7.30 (m, 3H), 7.08 (td, *J* = 8.2, 6.8 Hz, 1H), 6.44 – 6.29 (m, 3H), 4.79 (d, *J* = 1.5 Hz, 1H), 4.40 (ddd, *J* = 8.4, 4.7, 1.5 Hz, 2H), 4.19 (td, *J* = 8.8, 6.2 Hz, 1H), 4.10 (dt, *J* = 16.2, 8.1 Hz, 1H), 3.43 (dd, *J* = 16.5, 7.9 Hz, 2H), 3.29 (ddd, *J* = 14.5, 8.2, 5.4 Hz, 2H).

^{13}C NMR (126 MHz, $CDCl_3$) δ 165.2, 159.3, 149.8 (d, *J* = 10.6 Hz), 139.6, 130.5 (d, *J* = 10.2 Hz), 128.9, 128.4, 127.9, 109.2, 104.4 (d, J = 21.5 Hz), 100.0 (d, *J* = 8.6 Hz), 63.0, 61.6, 48.2, 48.0.

IR (film): ν (cm^{-1}) 3404, 2963, 2924, 2582, 1751, 1619, 1497, 1407, 1261, 1151, 1094, 1028, 801, 761, 703.

HRMS (ESI, m/z) calcd for $C_{17}H_{19}FN_3O_2$ $(M+H)^+$: 316.1456, found: 316.1456.

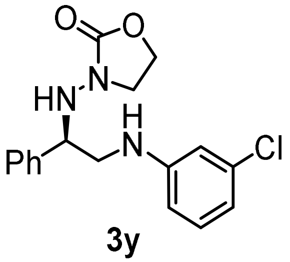

3y

A dried 10 mL Schlenk tube was charged with **1b** (38.0 mg, 0.20 mmol), **2m** (128.3 mg, 0.60 mmol), chiral copper catalyst **[L7-Cu^{II}]** (1.0 mL taken from the 20 mM solution in THF), and THF (1.0 mL). The mixture was degassed via three freeze-pump-thaw cycles. The Schlenk tube was positioned approximately 5 cm away from a 24 W blue LEDs lamp. After being stirred at -40 °C for 40 h, the reaction mixture was concentrated and then purified by flash chromatography on silica gel (eluted with PE:EtOAc = 2:1) to afford product **3y** as a white solid (42.8 mg, 0.129 mmol, yield: 65%). Enantiomeric excess was established by HPLC analysis using a Chiralpak AD-H column, e.e. = 97% (HPLC: AD-H, 220 nm, *n*-hexane/isopropanol = 90:10, flow rate: 1 mL/min, 30 °C, t_r(minor) = 34.9 min, t_r(major) = 28.4 min.) $[\alpha]_D^{23}$ = +5.5° (*c* = 1.0, CH_2Cl_2).

^{1}H NMR (500 MHz, $CDCl_3$) δ 7.52 (dt, *J* = 8.3, 2.2 Hz, 2H), 7.44 – 7.31 (m, 3H), 7.14 (t, *J* = 8.1 Hz, 1H), 6.72 (ddd, *J* = 7.9, 1.9, 0.7 Hz, 1H), 6.46 (t, *J* = 2.1 Hz, 1H), 6.35 (ddd, *J* = 8.3, 2.4, 0.7 Hz, 1H), 5.07 (dd, *J* = 9.4, 6.9 Hz, 1H), 4.75 (d, *J* = 3.0 Hz, 1H), 4.63 (d, *J* = 3.0 Hz, 1H), 4.26 – 4.09 (m, 2H), 3.77 (dd, *J* = 8.7, 7.0 Hz, 1H), 3.62 (ddd, *J* = 9.1, 8.0, 6.5 Hz, 1H), 3.39 – 3.26 (m, 2H).

^{13}C NMR (126 MHz, $CDCl_3$) δ 159.1, 149.1, 139.5, 134.9, 130.2, 128.7, 128.3, 127.7, 117.5, 112.6, 111.5, 62.8, 61.5, 48.0, 47.7.

IR (film): ν (cm^{-1}) 3448, 2962, 2924, 2854, 1773, 1663, 1597, 1490, 1466, 1261, 1097, 1031, 800, 759.

HRMS (ESI, m/z) calcd for $C_{17}H_{19}ClN_3O_2$ $(M+H)^+$: 332.1160, found: 332.1151.

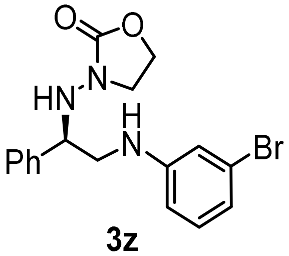

3z

A dried 10 mL Schlenk tube was charged with **1b** (38.0 mg, 0.20 mmol), **2n** (154.9 mg, 0.60 mmol), chiral copper catalyst **[L7-Cu^{II}]** (1.0 mL taken from the 20 mM solution in THF), and THF (1.0 mL). The mixture was degassed via three freeze-pump-thaw cycles. The Schlenk tube was positioned approximately 5 cm away from a 24 W blue LEDs lamp. After being stirred at -40 °C for 40 h, the reaction mixture was concentrated and then purified by flash chromatography on silica gel (eluted with PE:EtOAc = 2:1) to afford product **3z** as a white solid (50.33 mg, 0.134 mmol, yield: 67%). Enantiomeric excess was established by HPLC analysis using a Chiralpak AD-H column, e.e. = 98% (HPLC: AD-H, 220 nm, *n*-hexane/isopropanol = 90:10, flow rate: 1 mL/min, 30 °C, t_r(minor) = 36.7 min, t_r(major) = 30.6 min.) $[\alpha]_D^{23}$ = +23.0° (*c* = 1.0, CH_2Cl_2).

^{1}H NMR (500 MHz, $CDCl_3$) δ 7.52 (dt, *J* = 4.0, 2.2 Hz, 2H), 7.44 – 7.30 (m, 3H), 7.08 (t, *J* = 8.1 Hz, 1H), 6.91 – 6.83 (m, 1H), 6.62 (t, *J* = 2.1 Hz, 1H), 6.42 – 6.35 (m, 1H), 5.07 (dd, *J* = 9.4, 6.9 Hz, 1H), 4.74 (d, *J* = 3.0 Hz, 1H), 4.63 (d, *J* = 3.0 Hz, 1H), 4.27 – 4.08 (m, 2H), 3.77 (dd, *J* = 8.7, 7.0 Hz, 1H), 3.62 (ddd, *J* = 9.1, 8.0, 6.5 Hz, 1H), 3.39 – 3.26 (m, 2H).

^{13}C NMR (126 MHz, $CDCl_3$) δ 159.3, 149.3, 139.5, 130.7, 128.9, 128.4, 127.8, 123.4, 120.7, 115.7, 112.0, 63.0, 61.6, 48.0, 48.0.

IR (film): ν (cm^{-1}) 3482, 3315, 2923, 2853, 1648, 1595, 1459, 1407, 1262, 1094, 1032, 764, 702.

HRMS (ESI, m/z) calcd for $C_{17}H_{18}BrN_3O_2Na$ $(M+Na)^+$: 398.0475, found: 398.0489.

3.3 Optimization of the Enantioselective α-Aminoalkylation of Acyclic Imines Starting from Tertiary Amines by Cooperative Catalysis

General Procedure: A dried 10 mL Schlenk tube was charged with acyclic imines **1a** (0.20 mmol), tertiary amine **5a** (0.60 mmol), copper catalyst (0.020 mol, 1.0 mL taken from the 20 mM solution in CH_2Cl_2), indicated **PC**, indicated base and CH_2Cl_2 (1.0 mL). The mixture was degassed via three freeze-pump-thaw cycles. The Schlenk tube was positioned approximately 5 cm away from a 24 W LEDs lamp. After being stirred at the -40 °C for the indicated time, the reaction mixture was concentrated to dryness. The e.e. value was determined by chiral HPLC chromatography using a Daicel Chiralpak AD-H column or others.

Supplementary Table 2. Optimization of the enantioselective α-aminoalkylation of acyclic imines starting from tertiary amines by cooperative catalysis

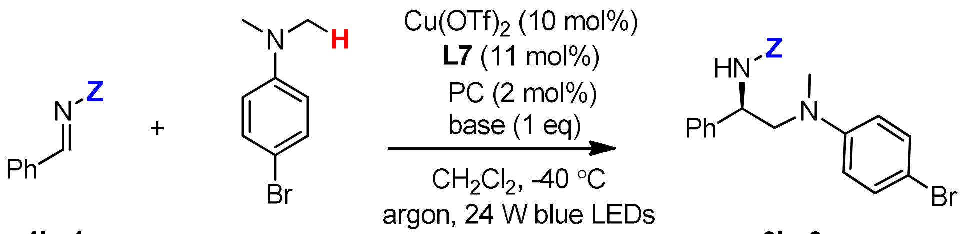

1b, **1a** **5a** **3b**, **6a**

entry[a]	substrate	PC	base	t (h)	product	yield (%)[b]	e.e. (%)[c]
1	**1b**	none	none	24	**3b**	0	n.a.
2	**1b**	none	CsOAc	24	**3b**	0	n.a.
3	**1b**	$Ir[dF(CF_3)ppy]_2(dtbbpy)PF_6$	CsOAc	40	**3b**	79	84
4	**1b**	$Ru(bpy)_3PF_6$	CsOAc	40	**3b**	58	85
5	**1a**	$Ir[dF(CF_3)ppy]_2(dtbbpy)PF_6$	CsOAc	40	**6a**	76	94

[a] Reaction conditions: **1b** or **1a** (0.20 mmol), **5a** (0.60 mmol), $Cu(OTf)_2$ (10 mol%), ligand **L7** (11 mol%), indicated photocatalyst (**PC**, 2 mol%), indicated base (1 eq), CH_2Cl_2 (2 mL), -40 °C, 24 W blue LEDs, under argon. [b] Isolated yield. [c] E.e. value determined by chiral HPLC. n.d. = not determined, n.a. = not applicable.

3.4 Substrate Scope for Asymmetric α-Aminoalkylation of Acyclic Imines Starting from Tertiary Amines

3.4.1 Preparation of 20 mM Solution of Non-Racemic Copper Catalyst [L7-Cu^{II}] in CH_2Cl_2

A solution of $Cu(OTf)_2$ (36.2 mg, 0.100 mmol) and non-racemic BOX ligand **L7** (57.0 mg, 0.110 mmol) in CH_2Cl_2 (5.0 mL) was stirred at 40 °C for 1 h, which was used freshly for the catalytic reactions.

3.4.2 General Procedure and Reaction Scope

A dried 10 mL Schlenk tube was charged with **1a** (0.20 mmol), **5a**–**5h** (0.60 mmol), chiral copper catalyst **[L7-Cu^{II}]** (1.0 mL taken from the 20 mM solution in CH_2Cl_2), $Ir[dF(CF_3)ppy]_2(dtbbpy)PF_6$ (0.0040 mmol), CsOAc (0.20 mmol), and CH_2Cl_2 (1.0 mL). The mixture was degassed via three freeze-pump-thaw cycles. The Schlenk tube was positioned approximately 5 cm away from a 24 W blue LEDs lamp. After being stirred at -40 °C for the indicated time, the reaction mixture was concentrated to dryness. The residue was purified by flash chromatography on silica gel (eluted with PE:EtOAc = 2:1) to afford non-racemic products **6a**–**6h**.

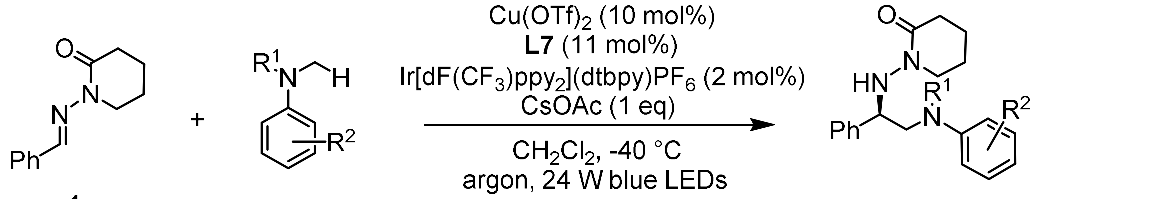

1a **5a–5h** **6a–6h**

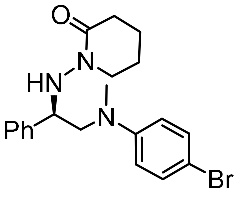

6a
40 h, 76% yield
94% e.e.

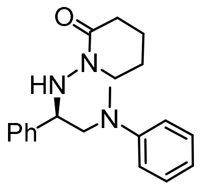

6b
40 h, 64% yield
96% e.e.

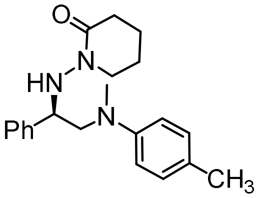

6c
40 h, 71% yield
96% e.e.

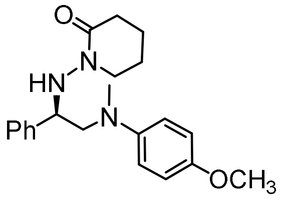

6d
40 h, 74% yield
94% e.e.

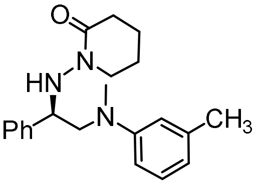

6e
40 h, 77% yield
96% e.e.

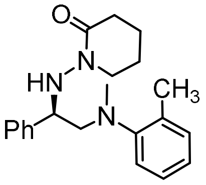

6f
40 h, 75% yield
95% e.e.

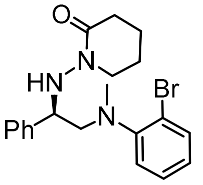

6g
40 h, 82% yield
93% e.e.

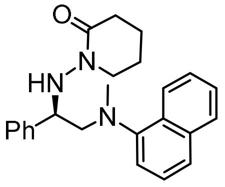

6h
24 h, 69% yield
78% e.e.

3.4.3 Experimental Details and Characterization Data

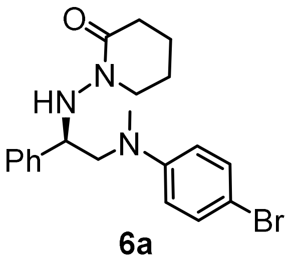

6a

A dried 10 mL Schlenk tube was charged with **1a** (40.5 mg, 0.20 mmol), **5a** (120.0 mg, 0.60 mmol), chiral copper catalyst **[L7-Cu^{II}]** (1.0 mL taken from the 20 mM solution in CH_2Cl_2), Ir[dF(CF_3)ppy]$_2$(dtbbpy)PF_6 (4.5 mg, 0.004 mmol), CsOAc (38.4 mg, 0.20 mmol), and CH_2Cl_2 (1.0 mL). The mixture was degassed via three freeze-pump-thaw cycles. The Schlenk tube was positioned approximately 5 cm away from a 24 W blue LEDs lamp. After being stirred at -40 °C for 40 h, the reaction mixture was concentrated and then purified by flash chromatography on silica gel (eluted with PE:EtOAc = 2:1) to afford product **6a** as a white

solid (60.8 mg, 0.152 mmol, yield: 76%). Enantiomeric excess was established by HPLC analysis using a Chiralpak OD-H column, e.e. = 94% (HPLC: OD-H, 220 nm, *n*-hexane/isopropanol = 97:3, flow rate: 1 mL/min, 30 °C, t_r(minor) = 24.2 min, t_r(major) = 19.9 min). $[\alpha]_D^{23}$ = +9.1° (*c* = 1.0, CH_2Cl_2).

^{1}H NMR (500 MHz, $CDCl_3$) δ 7.40 (d, *J* = 6.9 Hz, 2H), 7.37 – 7.27 (m, 5H), 6.68 (d, *J* = 9.0 Hz, 2H), 4.39 (dd, *J* = 8.6, 5.8 Hz, 1H), 3.72 (dd, *J* = 14.7, 8.7 Hz, 1H), 3.20 (dd, *J* = 14.5, 5.7 Hz, 2H), 2.91 – 2.75 (m, 4H), 2.42 – 2.26 (m, 2H), 1.62 – 1.44 (m, 4H).

^{13}C NMR (126 MHz, $CDCl_3$) δ 169.6, 149.0, 140.7, 131.9, 128.6, 128.3, 128.1, 114.7, 109.0, 61.8, 58.3, 51.9, 38.7, 32.4, 23.6, 21.1.

IR (film): ν (cm^{-1}) 3447, 2961, 2924, 2854, 1768, 1640, 1521, 1497, 1456, 1410, 1262, 1092, 1027, 804, 702.

HRMS (ESI, m/z) calcd for $C_{20}H_{24}BrN_3ONa$ $(M+Na)^+$: 424.0995, found: 424.0994.

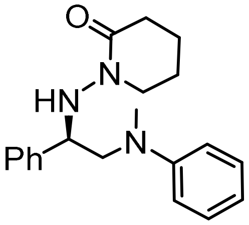

6b

A dried 10 mL Schlenk tube was charged with **1a** (40.5 mg, 0.20 mmol), **5b** (72.7 mg, 0.60 mmol), chiral copper catalyst **[L7-Cu^{II}]** (1.0 mL taken from the 20 mM solution in CH_2Cl_2), Ir[dF(CF_3)ppy]$_2$(dtbbpy)PF_6 (4.5 mg, 0.004 mmol), CsOAc (38.4 mg, 0.20 mmol), and CH_2Cl_2 (1.0 mL). The mixture was degassed via three freeze-pump-thaw cycles. The Schlenk tube was positioned approximately 5 cm away from a 24 W blue LEDs lamp. After being stirred at -40 °C for 40 h, the reaction mixture was concentrated and then purified by flash chromatography on silica gel (eluted with PE:EtOAc = 2:1) to afford product **6b** as a white solid (41.4 mg, 0.128 mmol, yield: 64%). Enantiomeric excess was established by HPLC analysis using a Chiralpak AD-H column, e.e. = 96% (HPLC: AD-H, 220 nm, *n*-hexane/isopropanol = 95:5, flow rate: 1 mL/min, 30 °C, t_r(minor) = 15.1 min, t_r(major) = 20.1 min). $[\alpha]_D^{23}$ = +16.2° (*c* = 1.0, CH_2Cl_2).

^{1}H NMR (500 MHz, $CDCl_3$) δ 7.46 (dt, *J* = 3.5, 2.0 Hz, 2H), 7.41 – 7.30 (m, 3H), 7.29 – 7.24 (m, 2H), 6.87 (d, *J* = 8.0 Hz, 2H), 6.76 (t, *J* = 7.3 Hz, 1H), 5.92 (s, 1H), 4.50 – 4.42 (m, 1H),

3.80 (dd, J = 14.6, 8.9 Hz, 1H), 3.24 (dt, J = 11.5, 4.5 Hz, 2H), 2.92 (s, 3H), 2.88 (dd, J = 11.7, 5.7 Hz, 1H), 2.44 – 2.29 (m, 2H), 1.68 – 1.46 (m, 4H).

^{13}C NMR (126 MHz, $CDCl_3$) δ 169.5, 150.2, 141.0, 129.3, 128.5, 128.4, 128.0, 117.1, 113.3, 61.9, 58.6, 51.9, 38.5, 32.5, 23.6, 21.1.

IR (film): ν (cm^{-1}) 3448, 3264, 2961, 2925, 2855, 1640, 1599, 1506, 1454, 1410, 1305, 1261, 1092, 1028, 801, 702.

HRMS (ESI, m/z) calcd for $C_{20}H_{25}N_3ONa$ $(M+Na)^+$: 346.1890, found: 346.1884.

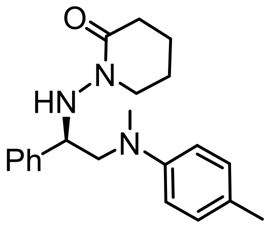

6c

A dried 10 mL Schlenk tube was charged with **1a** (40.5 mg, 0.20 mmol), **5c** (81.1 mg, 0.60 mmol), chiral copper catalyst **[L7-Cu^{II}]** (1.0 mL taken from the 20 mM solution in CH_2Cl_2), Ir[dF(CF_3)ppy]$_2$(dtbbpy)PF_6 (4.5 mg, 0.004 mmol), CsOAc (38.4 mg, 0.20 mmol), and CH_2Cl_2 (1.0 mL). The mixture was degassed via three freeze-pump-thaw cycles. The Schlenk tube was positioned approximately 5 cm away from a 24 W blue LEDs lamp. After being stirred at -40 °C for 40 h, the reaction mixture was concentrated and then purified by flash chromatography on silica gel (eluted with PE:EtOAc = 2:1) to afford product **6c** as a white solid (48.1 mg, 0.143 mmol, yield: 71%). Enantiomeric excess was established by HPLC analysis using a Chiralpak AD-H column, e.e. = 96% (HPLC: AD-H, 220 nm, *n*-hexane/isopropanol = 95:5, flow rate: 1 mL/min, 30 °C, t_r(minor) = 15.8 min, t_r(major) = 21.9 min). $[\alpha]_D^{23}$ = +58.8° (c = 1.0, CH_2Cl_2).

^{1}H NMR (500 MHz, $CDCl_3$) δ 7.43 (dt, J = 3.5, 2.0 Hz, 2H), 7.39 – 7.27 (m, 3H), 7.05 (d, J = 8.2 Hz, 2H), 6.83 – 6.71 (m, 2H), 5.94 (s, 1H), 4.43 (dd, J = 8.7, 5.5 Hz, 1H), 3.71 (dd, J = 14.4, 9.1 Hz, 1H), 3.27 – 3.11 (m, 2H), 2.89 (s, 3H), 2.88 – 2.81 (m, 1H), 2.43 – 2.28 (m, 2H), 2.26 (s, 3H), 1.67 – 1.43 (m, 4H).

^{13}C NMR (126 MHz, $CDCl_3$) δ 169.4, 148.3, 141.1, 129.8, 128.5, 128.4, 127.9, 126.5, 113.8, 61.8, 59.0, 52.0, 38.7, 32.5, 23.6, 21.1, 20.4.

IR (film): ν (cm^{-1}) 3448, 3263, 2925, 1735, 1640, 1456, 1305, 1245, 1122, 944, 803, 760, 703

HRMS (ESI, m/z) calcd for $C_{21}H_{27}N_3ONa$ $(M+Na)^+$: 360.2046, found: 360.2037.

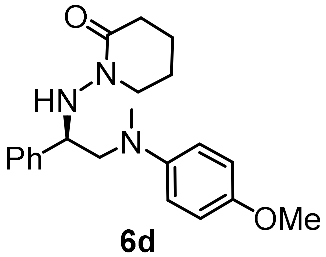

6d

A dried 10 mL Schlenk tube was charged with **1a** (40.5 mg, 0.20 mmol), **5d** (90.7 mg, 0.30 mmol), chiral copper catalyst **[L7-Cu^{II}]** (1.0 mL taken from the 20 mM solution in CH_2Cl_2), Ir[dF(CF_3)ppy]$_2$(dtbbpy)PF_6 (4.5 mg, 0.004 mmol), CsOAc (38.4 mg, 0.20 mmol), and CH_2Cl_2 (1.0 mL). The mixture was degassed via three freeze-pump-thaw cycles. The Schlenk tube was positioned approximately 5 cm away from a 24 W blue LEDs lamp. After being stirred at -40 °C for 40 h, the reaction mixture was concentrated and then purified by flash chromatography on silica gel (eluted with PE:EtOAc = 2:1) to afford product **6d** as a white solid (52.2 mg, 0.148 mmol, yield: 74%). Enantiomeric excess was established by HPLC analysis using a Chiralpak AD-H column, e.e. = 94% (HPLC: AD-H, 220 nm, *n*-hexane/isopropanol = 90:10, flow rate: 1 mL/min, 30 °C, t_r(minor) = 11.0 min, t_r(major) = 13.3 min). $[\alpha]_D^{23}$ = +13.3° (*c* = 1.0, CH_2Cl_2).

^{1}H NMR (500 MHz, $CDCl_3$) δ 7.42 (dt, *J* = 3.6, 2.0 Hz, 2H), 7.38 – 7.27 (m, 3H), 6.97 – 6.68 (m, 4H), 6.03 (s, 1H), 4.41 (dd, *J* = 9.5, 5.0 Hz, 1H), 3.76 (s, 3H), 3.59 (dd, *J* = 14.2, 9.5 Hz, 1H), 3.20 (ddd, *J* = 12.0, 7.4, 4.7 Hz, 1H), 3.10 (dd, *J* = 14.2, 5.0 Hz, 1H), 2.88 (s, 3H), 2.81 (dd, *J* = 11.8, 5.7 Hz, 1H), 2.35 (td, *J* = 6.2, 4.2 Hz, 2H), 1.58 – 1.41 (m, 4H).

^{13}C NMR (126 MHz, $CDCl_3$) δ 169.5, 152.4, 145.5, 141.1, 128.5, 128.4, 127.9, 116.0, 114.8, 61.6, 60.2, 55.8, 52.0, 39.3, 32.5, 23.6, 21.1.

IR (film): ν (cm^{-1}) 3448, 2959, 2918, 2849, 1637, 1460, 1264, 1096, 1027, 804, 741.

HRMS (ESI, m/z) calcd for $C_{21}H_{27}N_3O_2Na$ $(M+Na)^+$: 376.1995, found: 376.1994.

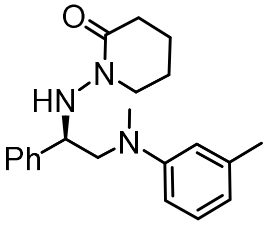

6e

A dried 10 mL Schlenk tube was charged with **1a** (40.5 mg, 0.20 mmol), **5e** (81.1 mg, 0.60 mmol), chiral copper catalyst **[L7-Cu^{II}]** (1.0 mL taken from the 20 mM solution in CH_2Cl_2), Ir[dF(CF_3)ppy]$_2$(dtbbpy)PF_6 (4.5 mg, 0.004 mmol), CsOAc (38.4 mg, 0.20 mmol), and CH_2Cl_2 (1.0 mL). The mixture was degassed via three freeze-pump-thaw cycles. The Schlenk tube was positioned approximately 5 cm away from a 24 W blue LEDs lamp. After being stirred at -40 °C for 40 h, the reaction mixture was concentrated and then purified by flash chromatography on silica gel (eluted with PE:EtOAc = 2:1) to afford product **6e** as a white solid (52.0 mg, 0.154 mmol, yield: 77%). Enantiomeric excess was established by HPLC analysis using a Chiralpak IC column, e.e. = 96% (HPLC: IC, 220 nm, *n*-hexane/isopropanol = 90:10, flow rate: 1 mL/min, 30 °C, t_r(minor) = 19.2 min, t_r(major) = 15.4 min). $[\alpha]_D^{23}$ = +28.7° (*c* = 1.0, CH_2Cl_2).

^{1}H NMR (500 MHz, $CDCl_3$) δ 7.39 (dd, *J* = 8.0, 1.3 Hz, 2H), 7.36 – 7.26 (m, 3H), 7.18 (d, *J* = 7.4 Hz, 1H), 7.15 – 7.08 (m, 1H), 6.99 (ddd, *J* = 12.1, 8.4, 4.5 Hz, 2H), 6.55 (s, 1H), 4.34 (dd, *J* = 11.0, 3.6 Hz, 1H), 3.24 – 3.08 (m, 2H), 2.96 (dd, *J* = 12.7, 3.7 Hz, 1H), 2.84 (s, 3H), 2.71 (dt, *J* = 11.9, 5.8 Hz, 1H), 2.42 (s, 3H), 2.39 (t, *J* = 6.6 Hz, 2H), 1.56 (dtdd, *J* = 46.1, 16.4, 7.7, 4.1 Hz, 4H).

^{13}C NMR (126 MHz, $CDCl_3$) δ 169.7, 150.3, 140.9, 138.8, 129.1, 128.5, 128.4, 128.0, 118.1, 114.0, 110.5, 61.8, 58.5, 51.9, 38.5, 32.3, 23.5, 22.0, 21.0.

IR (film): ν (cm^{-1}) 3448, 3266, 2960, 2855, 1637, 1602, 1497, 1458, 1411, 1305, 1260, 1095, 801, 702.

HRMS (ESI, m/z) calcd for $C_{21}H_{27}N_3ONa$ $(M+Na)^+$: 360.2046, found: 360.2039.

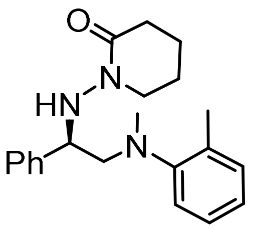

6f

A dried 10 mL Schlenk tube was charged with **1a** (40.5 mg, 0.20 mmol), **5f** (81.1 mg, 0.60 mmol), chiral copper catalyst **[L7-Cu^{II}]** (1.0 mL taken from the 20 mM solution in CH_2Cl_2), Ir[dF(CF_3)ppy]$_2$(dtbbpy)PF_6 (4.5 mg, 0.004 mmol), CsOAc (38.4 mg, 0.20 mmol), and CH_2Cl_2 (1.0 mL). The mixture was degassed via three freeze-pump-thaw cycles. The Schlenk tube was positioned approximately 5 cm away from a 24 W blue LEDs lamp. After being stirred at -40 °C for 40 h, the reaction mixture was concentrated and then purified by flash chromatography on silica gel (eluted with PE:EtOAc = 2:1) to afford product **6f** as a white solid (50.5 mg, 0.150 mmol, yield: 75%). Enantiomeric excess was established by HPLC analysis using a Chiralpak AD-H column, e.e. = 95% (HPLC: AD-H, 220 nm, *n*-hexane/isopropanol = 97:3, flow rate: 1 mL/min, 30 °C, t_r(minor) = 20.8 min, t_r(major) = 18.6 min). $[\alpha]_D^{23}$ = +78.6° (*c* = 1.0, CH_2Cl_2).

^{1}H NMR (500 MHz, $CDCl_3$) δ 7.39 (dd, *J* = 8.0, 1.3 Hz, 2H), 7.36 – 7.26 (m, 3H), 7.18 (d, *J* = 7.4 Hz, 1H), 7.15 – 7.08 (m, 1H), 6.99 (ddd, *J* = 12.1, 8.4, 4.5 Hz, 2H), 6.55 (s, 1H), 4.34 (dd, *J* = 11.0, 3.6 Hz, 1H), 3.24 – 3.08 (m, 2H), 2.96 (dd, *J* = 12.7, 3.7 Hz, 1H), 2.84 (s, 3H), 2.71 (dt, *J* = 11.9, 5.8 Hz, 1H), 2.42 (s, 3H), 2.39 (t, *J* = 6.6 Hz, 2H), 1.56 (dtdd, *J* = 46.1, 16.4, 7.7, 4.1 Hz, 4H).

^{13}C NMR (126 MHz, $CDCl_3$) δ 169.5, 153.0, 141.5, 133.9, 131.1, 128.5, 128.4, 127.9, 126.6, 123.6, 120.7, 61.6, 61.2, 52.2, 42.4, 32.6, 23.6, 21.1, 18.5.

IR (film): ν (cm^{-1}) 3448, 2923, 2851, 1648, 1491, 1459, 1263, 1095, 1026, 801, 737, 703.

HRMS (ESI, m/z) calcd for $C_{21}H_{27}N_3ONa(M+Na)^+$: 360.2046, found: 360.2041.

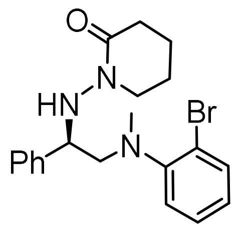

6g

A dried 10 mL Schlenk tube was charged with **1a** (40.5 mg, 0.20 mmol), **5g** (120.0 mg, 0.60 mmol), chiral copper catalyst **[L7-Cu^{II}]** (1.0 mL taken from the 20 mM solution in CH_2Cl_2), Ir[dF(CF_3)ppy]$_2$(dtbbpy)PF_6 (4.5 mg, 0.004 mmol), CsOAc (38.4 mg, 0.20 mmol), and CH_2Cl_2 (1.0 mL). The mixture was degassed via three freeze-pump-thaw cycles. The Schlenk tube was positioned approximately 5 cm away from a 24 W blue LEDs lamp. After being stirred at -40 °C for 40 h, the reaction mixture was concentrated and then purified by flash chromatography on silica gel (eluted with PE:EtOAc = 2:1) to afford product **6g** as a white solid (65.7 mg, 0.164 mmol, yield: 82%). Enantiomeric excess was established by HPLC analysis using a Chiralpak AD-H column, e.e. = 93% (HPLC: AD-H, 220 nm, *n*-hexane/isopropanol = 97:3, flow rate: 1 mL/min, 30 °C, t_r(minor) = 20.8 min, t_r(major) = 18.7 min). $[\alpha]_D^{23}$ = +57.4° (*c* = 1.0, CH_2Cl_2).

^{1}H NMR (500 MHz, $CDCl_3$) δ 7.57 (dd, *J* = 7.9, 1.5 Hz, 1H), 7.40 (dd, *J* = 8.0, 1.4 Hz, 2H), 7.35 – 7.27 (m, 3H), 7.23 (td, *J* = 8.0, 1.5 Hz, 1H), 7.10 (dd, *J* = 8.0, 1.5 Hz, 1H), 6.92 (td, *J* = 7.9, 1.5 Hz, 1H), 6.46 (s, 1H), 4.45 (dd, *J* = 11.0, 3.0 Hz, 1H), 3.30 (dd, *J* = 12.4, 11.3 Hz, 1H), 3.17 (ddd, *J* = 11.9, 7.7, 4.4 Hz, 1H), 3.02 (dd, *J* = 12.7, 3.7 Hz, 1H), 2.96 (s, 3H), 2.74 – 2.63 (m, 1H), 2.37 (t, *J* = 6.5 Hz, 2H), 1.62 – 1.35 (m, 4H).

^{13}C NMR (126 MHz, $CDCl_3$) δ 169.7, 152.0, 141.0, 133.8, 128.6, 128.4, 128.2, 128.0, 125.0, 123.1, 121.5, 61.0, 60.5, 52.3, 42.1, 32.7, 23.7, 21.1.

IR (film): ν (cm^{-1}) 3448, 3261, 2918, 2849, 1641, 1474, 1326, 1264, 1115, 1027, 943, 737, 702.

HRMS (ESI, m/z) calcd for $C_{20}H_{24}BrN_3ONa$ $(M+Na)^+$: 424.0995, found: 424.0988.

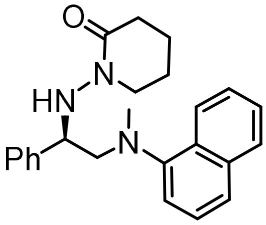

6h

A dried 10 mL Schlenk tube was charged with **1a** (40.5 mg, 0.20 mmol), **5h** (102.7 mg, 0.60 mmol), chiral copper catalyst **[L7-Cu^{II}]** (1.0 mL taken from the 20 mM solution in CH_2Cl_2), Ir[dF(CF_3)ppy]$_2$(dtbbpy)PF_6 (4.5 mg, 0.004 mmol), CsOAc (38.4 mg, 0.20 mmol), and CH_2Cl_2 (1.0 mL). The mixture was degassed via three freeze-pump-thaw cycles. The Schlenk tube was positioned approximately 5 cm away from a 24 W blue LEDs lamp. After being stirred at 25 °C for 24 h, the reaction mixture was concentrated and then purified by flash chromatography on silica gel (eluted with PE:EtOAc = 2:1) to afford product **6h** as a white solid (51.6 mg, 0.138 mmol, yield: 69%). Enantiomeric excess was established by HPLC analysis using a Chiralpak AD-H column, e.e. = 78% (HPLC: AD-H, 220 nm, *n*-hexane/isopropanol = 97:3, flow rate: 1 mL/min, 30 °C, t_r(minor) = 24.2 min, t_r(major) = 19.9 min). $[\alpha]_D^{23}$ = +13.4° (*c* = 1.0, CH_2Cl_2).

^{1}H NMR (500 MHz, $CDCl_3$) δ 8.50 (d, *J* = 8.5 Hz, 1H), 7.81 (d, *J* = 8.0 Hz, 1H), 7.58 – 7.50 (m, 2H), 7.49 – 7.40 (m, 3H), 7.38 – 7.29 (m, 4H), 7.09 (dd, *J* = 7.4, 0.8 Hz, 1H), 6.66 (s, 1H), 4.51 (dd, *J* = 11.1, 3.5 Hz, 1H), 3.51 – 3.42 (m, 1H), 3.17 (ddd, *J* = 12.3, 7.7, 4.7 Hz, 1H), 3.13 – 2.98 (m, 4H), 2.74 (dt, *J* = 12.0, 5.9 Hz, 1H), 2.43 (t, *J* = 6.6 Hz, 2H), 1.68 – 1.60 (m, 2H), 1.53 – 1.40 (m, 2H).

^{13}C NMR (126 MHz, $CDCl_3$) δ 169.7, 151.4, 141.5, 134.9, 129.7, 128.5, 128.4, 128.3, 128.0, 127.1, 125.9, 125.8, 124.4, 123.8, 116.2, 62.1, 61.3, 52.3, 43.4, 32.7, 23.6, 21.1.

IR (film): ν (cm^{-1}) 3448, 2962, 1735, 1637, 1577, 1458, 1396, 1261, 1096, 1018, 801, 701

HRMS (ESI, m/z) calcd for $C_{24}H_{27}N_3ONa$ $(M+Na)^+$: 396.2046, found: 396.2041.

4. Synthetic Tansformation and Absolute Configuration Assignment of the Products

4.1 Transformation of 3q to Chiral 1,2-Diamine 7

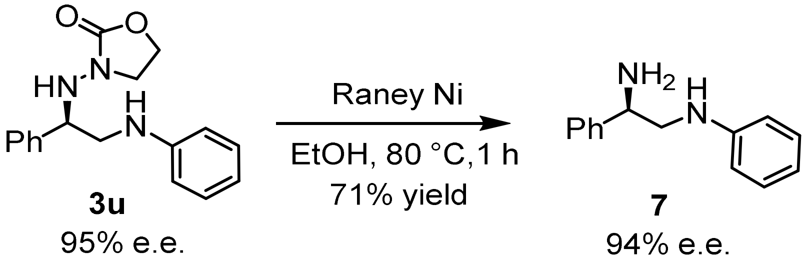

To a Schlenk tube equipped with a magnetic stir bar was added **3u** (52.9 mg, 0.178 mmol, product of the copper-catalyzed asymmetric photoredox reaction, 95% ee), Raney Ni (~ 300 mg, freshly prepared and washed with EtOH) and EtOH (3 mL). The reaction mixture was stirred at 80 °C for 1 h. The reaction mixture was passed through a short pad of celite, and then purified with flash chromatography on silica gel (eluted with EtOAc/PE = 1:1) to afford **7** (26.8 mg, 0.127 mmol, yield: 71%) as a pale yellow oil. Enantiomeric excess was established by HPLC analysis using a Chiralpak IC column, e.e. = 94% (HPLC: IC, 220 nm, *n*-hexane/isopropanol = 90:10, flow rate 1 mL/min, 30 °C, t_r(minor) =30.3 min, t_r(major) = 23.7 min). $[\alpha]_D^{23}$ = +8.6° (c = 1.0, CH_2Cl_2).

^{1}H NMR (500 MHz, $CDCl_3$) δ 7.41 – 7.33 (m, 4H), 7.30 (td, J = 8.6, 4.3 Hz, 1H), 7.18 (t, J = 7.9 Hz, 2H), 6.72 (t, J = 7.3 Hz, 1H), 6.65 (d, J = 7.8 Hz, 2H), 4.19 (dd, J = 8.2, 5.1 Hz, 1H), 4.06 (s, 1H), 3.37 (dd, J = 12.7, 5.0 Hz, 1H), 3.23 (dd, J = 12.7, 8.3 Hz, 1H), 1.65 (s, 2H).

Other analytic data of **7** are consistent with the literature.[12]

4.2 Transformation of 3n to Dihydroquinoxazline 8

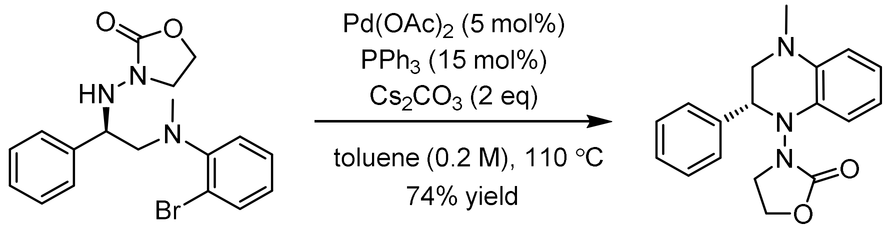

3o
91% e.e.

8
91% e.e.
Dihydroquinoxaline

To a solution of **3o** (78.1 mg, 0.20 mmol) in dry toluene (1.0 mL) was added Cs_2CO_3 (130.3 mg, 0.40 mmol), PPh_3 (7. 9 mg, 0.030 mmol), and $Pd(OAc)_2$ (2.2 mg, 0.010 mmol). The reaction mixture was stirred at 110 °C under Ar for 21 h. Then the resulting mixture was cooled to room temperature, and concentrated under reduced pressure. The residue was purified by flash chromatography on silica gel (eluted with EtOAc/PE = 1:3) to afford the product as a white solid (45.7 mg, 0.148 mmol, yield: 74%). Enantiomeric excess was established by HPLC analysis using a Chiralpak OD-H column, e.e. = 91% (HPLC: OD-H, 220 nm, *n*-hexane/isopropanol = 90:10, flow rate 1 mL/min, 30 °C, t_r(minor) =52.1 min, t_r(major) = 39.1 min). $[\alpha]_D^{23}$ = +49.9° (*c* = 1.0, CH_2Cl_2).

^{1}H NMR (600 MHz, $CDCl_3$) δ 7.82 – 7.29 (m, 5H), 6.99 – 6.55 (m, 4H), 4.84 (d, J = 322.9 Hz, 1H), 4.30 (s, 1H), 4.02 (d, J = 39.0 Hz, 1H), 3.86 – 3.14 (m, 4H), 2.92 (d, J = 66.5 Hz, 3H).

IR (film): ν (cm^{-1}) 3448, 2963, 2922, 1752, 1654, 1598, 1507, 1411, 1262, 1149, 1096, 1027, 799, 751.

HRMS (ESI, m/z) calcd for $C_{18}H_{19}N_3O_2Na$ $(M+Na)^+$: 332.1369, found: 332.1376.

4.3 Absolute Configuration Assignment of the Products

The absolute configuration of product **7** (94% ee, synthesized from asymmetric photoredeox product **3u**) was assigned as *R* according to the literature.[12] Accordingly, asymmetric photoredeox products **3b**, **3d**−**3z** and **6a**−**6h** were assigned as *R* in analogy.

5. Mechanistic Investigations

5.1 UV/Vis-Absorption Spectra of the Reaction Components

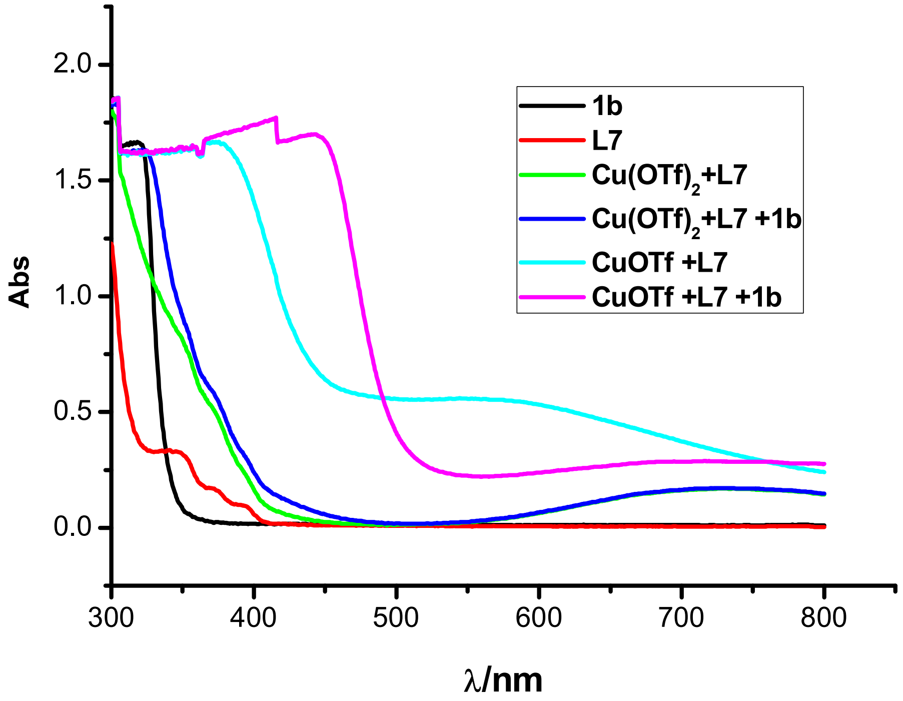

Supplementary Figure 1. UV-Vis spectra of the individual substrates **1b**, chiral ligand **L7**, copper salts $Cu(OTf)_2$, CuOTf, copper complexes **[L7-Cu^{I}]** (generated in situ by stirring a 1:1 mixture of CuOTf and chiral ligand **L7**), **[L7-Cu^{I}-1b]** (generated in situ by stirring a 1:1:1 mixture of CuOTf, chiral ligand **L7** and substrate **1b**), **[L7-Cu^{II}]** (generated in situ by stirring a 1:1 mixture of $Cu(OTf)_2$ and chiral ligand **L7**) and **[L7-Cu^{II}-1b]** (generated in situ by stirring a 1:1:1 mixture of $Cu(OTf)_2$, chiral ligand **L7** and substrate **1b**). All the samples were prepared as a 0.010 M solution and used freshly for the measurement.

Preparation of the samples for UV-Vis spectra measurement. (All the samples were used freshly for UV-Vis spectra measurement.)

1b in THF (0.010 M): 1b (1.90 mg, 0.010 mmol) was dissolved in distilled THF (1 mL).

L7 in THF (0.010 M): BOX ligand **L7** (5.70 mg, 0.010 mmol) was dissolved in distilled THF (1 mL).

[L7-Cu^{I}] in THF (0.010 M): A solution of CuOTf (2.12 mg, 0.010 mmol) and BOX ligand **L7** (5.70 mg, 0.010 mmol) in distilled THF (1 mL) was stirred at 40 °C for 1 h.

[L7-Cu^{I}-1b] in THF (0.010 M): A solution of CuOTf (2.12 mg, 0.010 mmol) and BOX ligand **L7** (5.70 mg, 0.010 mmol) in distilled THF (1 mL) was stirred at 40 °C for 1 h, then **1b** (1.90 mg, 0.010 mmol) was added. The resulting mixure was heated at 40 °C for additional 1 h.

[L7-Cu^{II}] in THF (0.010 M): A solution of $Cu(OTf)_2$ (3.62 mg, 0.010 mmol) and BOX ligand **L7** (5.70 mg, 0.010 mmol) in distilled THF (1 mL) was stirred at 40 °C for 1 h.

[L7-Cu^{II}-1b] in THF (0.010 M): A solution of $Cu(OTf)_2$ (3.62 mg, 0.010 mmol) and BOX ligand **L7** (5.70 mg, 0.010 mmol) in distilled THF (1 mL) was stirred at 40 °C for 1 h, then **1b** (1.90 mg, 0.010 mmol) was added. The resulting mixure was heated at 40 °C for additional 1 h.

Remarks: All of the individual chiral ligand **L7**, substrates **1b** had no obvious absorption in the visible light region. Chiral copper complex **[L7-Cu^{II}]**, and potential intermediate **[L7-Cu^{II}-1b]** exhibited weak absorption enhancement in the range of 400–550 nm. However, chiral copper complex **[L7-Cu^{I}]**, and potential intermediate **[L7-Cu^{I}-1b]** exhibited significant absorption enhancement in the range of 400–550 nm.

5.2 Quantum Yield Measurement

Photon flux of the 24 W blue LEDs was determined as 1.61×10^{-8} einstein/s by standard ferrioxalate actinometry.[6]

Model reaction:

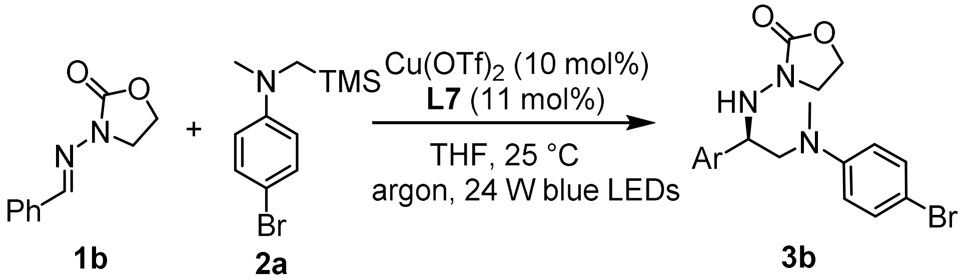

Preparation of the model reaction solution: **1b** (19.0 mg, 0.10 mmol), **2a** (81.7 mg, 0.30 mmol), chiral copper catalyst **[L7-Cu^{II}]** (0.5 mL taken from the 20 mM solution in THF) were sequentially added to a 0.5 mL volumetric flask and filled to the mark with THF.

1 mL of the model reaction solution were added to a Schlenk tube (diameter = 12 mm). The Schlenk tube were placed 5 cm away from the light source. The mixture was degassed via three freeze-pump-thaw cycles and irradiated with the 24 W blue LEDs Lamp. This procedure was repeated 4 times, quenching the reaction after different time intervals: 1 h, 2 h, 3 h, and 4 h for the model reaction.

$$\Phi(\lambda) = \frac{dx/dt}{q^{0}_{n,p}[1-10^{-A(\lambda)}]} \qquad \text{(Eq. 1)}$$

The moles of product **3b** formed for the model reaction were determined by ^{1}H NMR. The moles of product per unit of time are related to the number of photons absorbed. The photons absorbed are correlated to the number of incident photons by the use of Eq. 1. According to this, if we plot the moles of product (x) versus the moles of incident photons ($q^0_{n,p}\cdot dt$), the slope is equal to: $\mathbf{\Phi\cdot(1\text{-}10^{-A(400\ nm)})}$, where Φ is the quantum yield to be determined and A(400 nm) is the absorption of the reaction under study.

A(400 nm) was measured using a Shimadzu UV-2550 UV-Vis spectrophotometer in 10 mm path quartz. An absorbance of 1.69 was determined for the model reaction mixture.

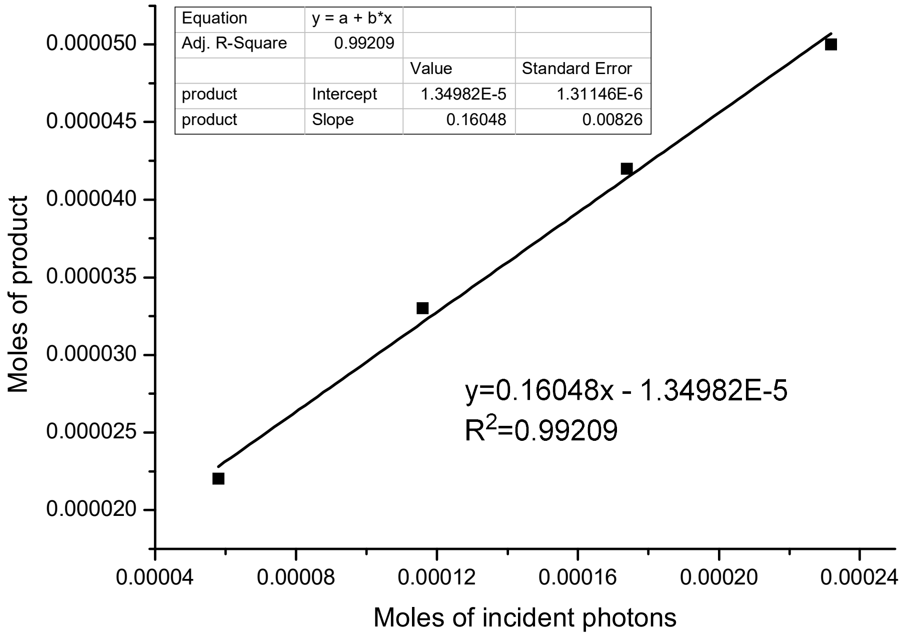

Supplementary Figure 2. The moles of product (x) versus the moles of incident photons ($q^0_{n,p}\cdot dt$).

Conclusion: The quantum yield (Φ) of the photochemical reaction of **1b** with **2a** catalyzed chiral copper catalyst **L7-Cu^II** was calculated to be **0.164**.

5.3 Control Experiments

5.3.1 Photocatalytic Reaction Interfered with Air

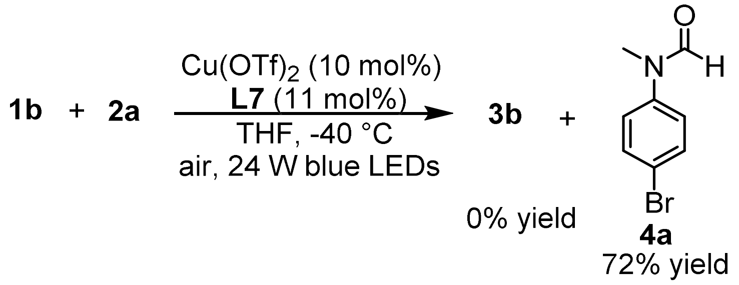

A dried 10 mL round bottom flask was charged with **1b** (38.0 mg, 0.20 mmol), **2a** (163.4 mg, 0.60 mmol), chiral copper catalyst **[L7-Cu^II]** (1.0 mL taken from the 20 mM solution in

THF), and THF (1 mL). The round bottom flask was positioned approximately 5 cm away from a 24 W blue LEDs lamp. The reaction was stirred at -40 °C for 12 h under an atmosphere of air (air balloon). The reaction mixture was concentrated and then purified by flash chromatography on silica gel (eluted with PE:EtOAc = 2:1) to afford product **4a** as a white solid (92.0 mg, 0.432 mmol, yield: 72% based on **2a**).

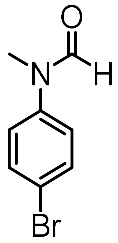

4a

^{1}H NMR (500 MHz, $CDCl_3$) δ 8.45 (s, 1H), 7.53 (d, J = 8.7 Hz, 2H), 7.05 (d, J = 8.7 Hz, 2H), 3.30 (s, 3H).

Other analytic data of **4a** are consistent with the literature.[13]

5.3.2 Catalytic Reaction Interfered with a Competitive Radical Acceptor

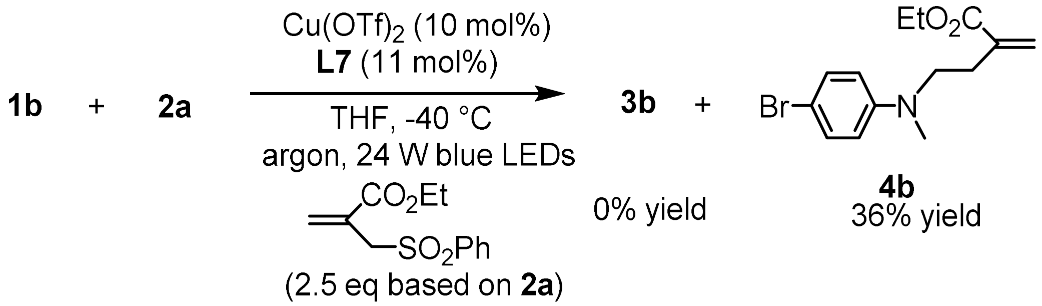

A dried 10 mL Schlenk tube was charged with **1b** (19.0 mg, 0.10 mmol), **2a** (81.7 mg, 0.30 mmol), chiral copper catalyst **[L7-Cu^{II}]** (1.0 mL taken from the 20 mM solution in THF), ethyl 2-((phenylsulfonyl)methyl)acrylate (190.5 mg, 0.75 mmol) and THF (1.0 mL). The mixture was degassed via three freeze-pump-thaw cycles. The Schlenk tube was positioned approximately 5 cm away from a 24 W blue LEDs lamp. After being stirred at -40 °C for 24 h, the reaction mixture was concentrated and then purified by flash chromatography on silica gel (eluted with PE:EtOAc = 5:1) to afford product **4b** as a white solid (33.5 mg, 0.108 mmol, yield: 36% based on **2a**).

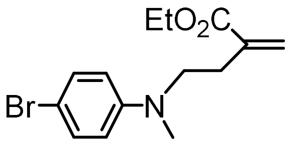

4b

^{1}H NMR (500 MHz, $CDCl_3$) δ 7.29 (d, *J* = 9.0 Hz, 2H), 6.60 (d, *J* = 8.8 Hz, 2H), 6.20 (d, *J* = 1.0 Hz, 1H), 5.58 (s, 1H), 4.23 (q, *J* = 7.1 Hz, 2H), 3.59 – 3.35 (m, 2H), 2.92 (s, 3H), 2.64 – 2.43 (m, 2H), 1.33 (t, *J* = 7.1 Hz, 3H).

^{13}C NMR (126 MHz, $CDCl_3$) δ 167.0, 147.9, 138.3, 132.0, 127.0, 113.8, 108.1, 61.0, 52.4, 38.5, 29.6, 14.4.

IR (film): ν (cm^{-1}) 3413, 2963, 2905, 1717, 1629, 1593, 1500, 1412, 1262, 1104, 1022, 807, 684.

HRMS (ESI, m/z) calcd for $C_{14}H_{18}BrNO_2Na$ $(M+Na)^+$: 334.0413, found: 334.0417.

5.3.3 Evidence for Lewis acid activation.

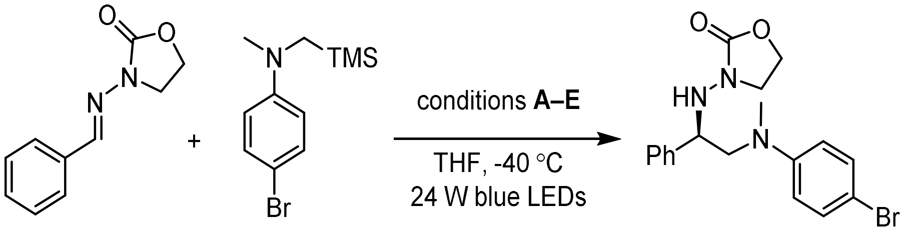

Condition A: A dried 10 mL Schlenk tube was charged with **1b** (38.0 mg, 0.20 mmol), **2a** (163.4 mg, 0.60 mmol), chiral copper catalyst **[L7-Cu^{II}]** (1.0 mL taken from the 20 mM solution in THF), and THF (1.0 mL). The mixture was degassed via three freeze-pump-thaw cycles. The Schlenk tube was positioned approximately 5 cm away from a 24 W blue LEDs lamp. After being stirred at -40 °C for 20 h, the reaction mixture was concentrated and then purified by flash chromatography on silica gel (eluted with PE:EtOAc = 2:1) to afford product **3b** as a white solid (45.9 mg, 0.118 mmol, yield: 59%).

Condition B: A dried 10 mL Schlenk tube was charged with **1b** (38.0 mg, 0.20 mmol), **2a** (163.4 mg, 0.60 mmol), chiral copper catalyst **[L7-Cu^{II}]** (1.0 mL taken from the 20 mM

solution in THF), $Ir[dF(CF_3)ppy]_2(dtbbpy)PF_6$ (4.5 mg, 0.004 mmol), and THF (1.0 mL). The mixture was degassed via three freeze-pump-thaw cycles. The Schlenk tube was positioned approximately 5 cm away from a 24 W blue LEDs lamp. After being stirred at -40 °C for 20 h, the reaction mixture was concentrated and then purified by flash chromatography on silica gel (eluted with PE:EtOAc = 2:1) to afford product **3b** as a white solid (62.9 mg, 0.162 mmol, yield: 81%).

Condition C: A dried 10 mL Schlenk tube was charged with **1b** (38.0 mg, 0.20 mmol), **2a** (163.4 mg, 0.60 mmol), $Ir[dF(CF_3)ppy]_2(dtbbpy)PF_6$ (4.5 mg, 0.004 mmol), and THF (1.0 mL). The mixture was degassed via three freeze-pump-thaw cycles. The Schlenk tube was positioned approximately 5 cm away from a 24 W blue LEDs lamp. After being stirred at -40 °C for 20 h, traces of product **3b** were afforded (detected by TLC and ^1H NMR analysis).

Condition D: A dried 10 mL Schlenk tube was charged with **1b** (38.0 mg, 0.20 mmol), **2a** (163.4 mg, 0.60 mmol), chiral nickel catalyst **[L7-Ni^{II}]** (1.0 mL taken from the 20 mM solution in THF), $Ir[dF(CF_3)ppy]_2(dtbbpy)PF_6$ (4.5 mg, 0.004 mmol), and THF (1.0 mL). The mixture was degassed via three freeze-pump-thaw cycles. The Schlenk tube was positioned approximately 5 cm away from a 24 W blue LEDs lamp. After being stirred at -40 °C for 20 h, the reaction mixture was concentrated and then purified by flash chromatography on silica gel (eluted with PE:EtOAc = 2:1) to afford product **3b** as a white solid (24.1 mg, 0.062 mmol, yield: 31%).

Condition E: A dried 10 mL Schlenk tube was charged with **1b** (38.0 mg, 0.20 mmol), **2a** (163.4 mg, 0.60 mmol), chiral zinc catalyst **[L7-Zn^{II}]** (1.0 mL taken from the 20 mM solution in THF), $Ir[dF(CF_3)ppy]_2(dtbbpy)PF_6$ (4.5 mg, 0.004 mmol), and THF (1.0 mL). The mixture was degassed via three freeze-pump-thaw cycles. The Schlenk tube was positioned approximately 5 cm away from a 24 W blue LEDs lamp. After being stirred at -40 °C for 20 h, the reaction mixture was concentrated and then purified by flash chromatography on silica gel (eluted with PE:EtOAc = 2:1) to afford product **3b** as a white solid (11.7 mg, 0.030 mmol, yield: 15%).

5.3.4 Oxidation of 2a by [L7-Cu^{II}] in the Dark

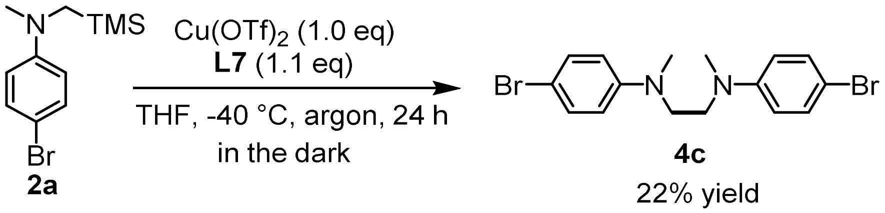

A dried 10 mL Schlenk tube was charged with **2a** (54.5 mg, 0.20 mmol) and chiral copper catalyst **[L7-Cu^{II}]** (2 mL taken from the 0.1 M solution in THF). The mixture was degassed via three freeze-pump-thaw cycles. After being stirred at -40 °C for 24 h in the dark, the reaction mixture was concentrated and then purified by flash chromatography on silica gel (eluted with PE:CH_2Cl_2 = 10:1) to afford product **4c** as a white solid (8.7 mg, 0.022 mmol, yield: 22%).

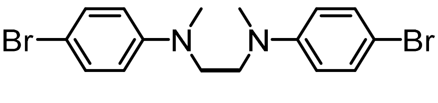

4c

^{1}H NMR (500 MHz, $CDCl_3$) δ 7.29-7.24 (m, 4 H), 6.53-6.50 (m, 4 H), 3.49 (s, 4 H), 2.87 (s, 6 H).

Other analytic data of **4c** are consistent with the literature.[14]

5.3.5 Oxidation of 2a by [L7-Cu^{II}] under Irradiation with Blue LEDs

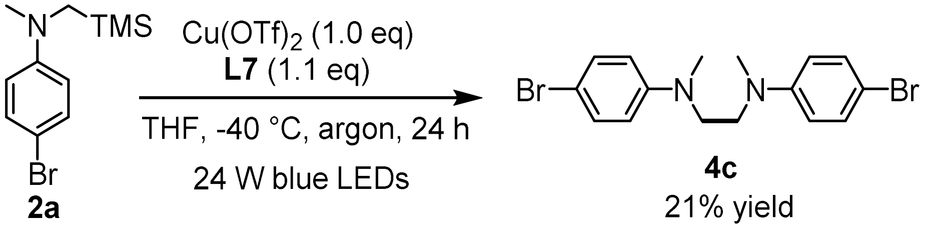

A dried 10 mL Schlenk tube was charged with **2a** (54.5 mg, 0.20 mmol) and chiral copper catalyst **[L7-Cu^{II}]** (2 mL taken from the 0.1 M solution in THF). The mixture was degassed via three freeze-pump-thaw cycles. The Schlenk tube was positioned approximately 5 cm away from a 24 W blue LEDs lamp. After being stirred at -40 °C for 24 h, the reaction mixture was concentrated and then purified by flash chromatography on silica gel (eluted

with PE:CH_2Cl_2 = 10:1) to afford product **4c** as a white solid (8.3 mg, 0.021 mmol, yield: 21%).

5.4 An Alternative Radical Coupling Mechanism for Photocatalytic Enantioselective α-aminoalkylation of Acyclic Imine Derivatives by a Chiral Copper Complex

A radical coupling pathway could not be completely excluded: **[L7-Cu^{II}]** single-electronically oxidized α-silylamine **2**, and generate **[L7-Cu^{I}]** and silylamine cation radical **II**. Accordingly, substrate **1** underwent fast ligand exchange with **[L7-Cu^{II}]** and afforded the intermediate complex **I**. Under irradiation of visible light, excited state of **[L7-Cu^{I}]** single-electronically reduced complex **I** to **IV**. Subsequent desilylation of **II** led to formation of α-amino radical **III**, followed by a radical couping with **IV**. Finally, further hydrolysis afforded the final product **3** and release intermediate **I** for the next catalytic cycle.

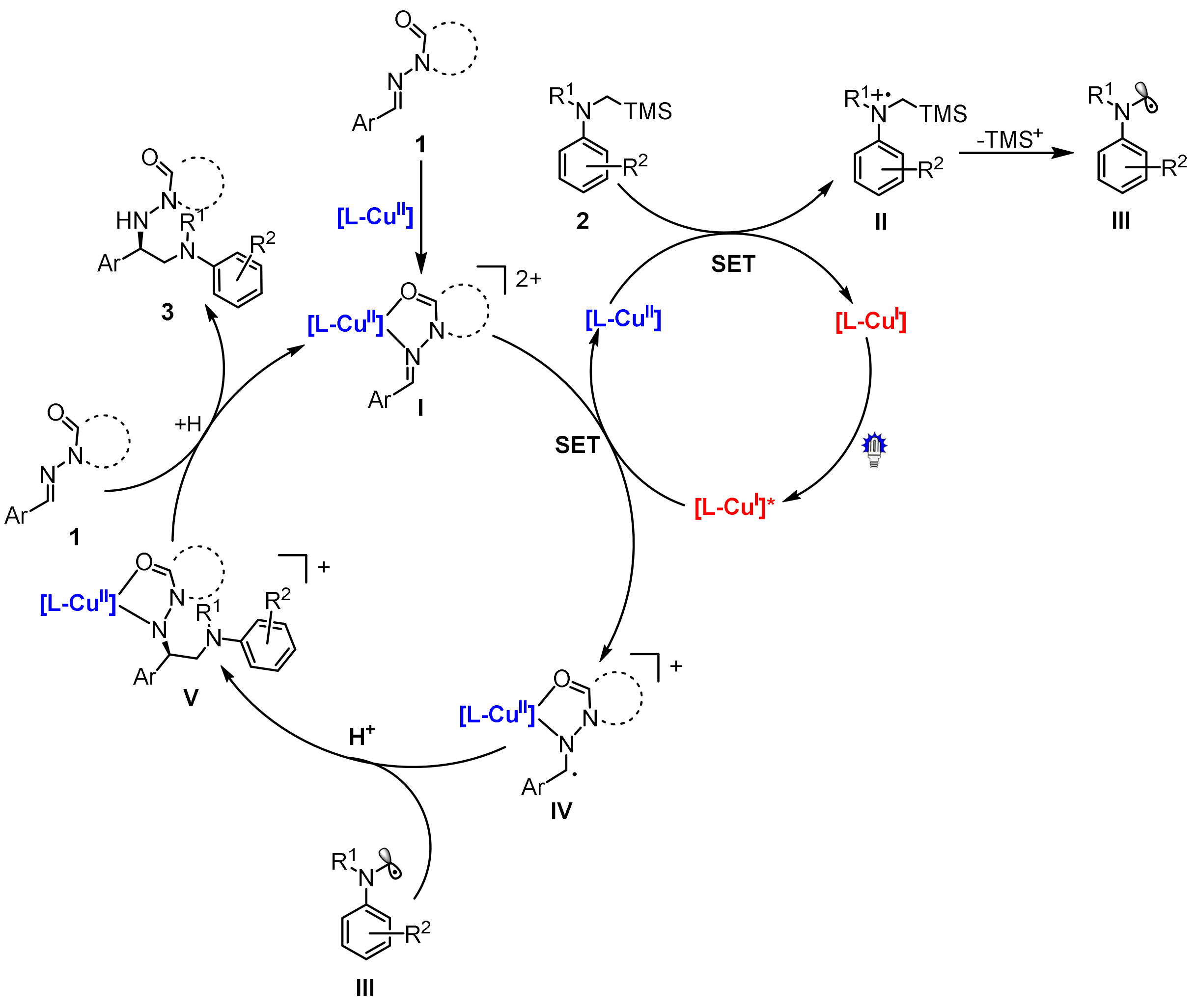

Supplementary Figure 3. An alternative radical coupling mechanism.

5.5 Luminescence Quenching Experiments

Emission intensities were recorded on a Spectra Max M5 microplate reader in a 10.0 mm quartz cuvette. Ir[dF(CF$_3$)ppy]$_2$(dtbbpy)PF$_6$ were excited at 400 nm. The concentration of Ir[dF(CF$_3$)ppy]$_2$(dtbbpy)PF$_6$ was 3.30 mM in CH_2Cl_2.

The concentration of Ir[dF(CF$_3$)ppy]$_2$(dtbbpy)PF$_6$ was 3.30 mM in CH_2Cl_2. The concentration of the quencher (**1a** and **5a**) was 3.30 mM in CH_2Cl_2. For each quenching experiment, **1a** or **5a** were titrated to a solution (5 mL) of the Ir[dF(CF$_3$)ppy]$_2$(dtbbpy)PF$_6$ solvent in a quartz glass bottle. The addition of **1a** or **5a** refers to an increase of the quencher

concentration of 16.50 mM. After degassing with an argon stream for 5 minutes, the emission intensity was collected.

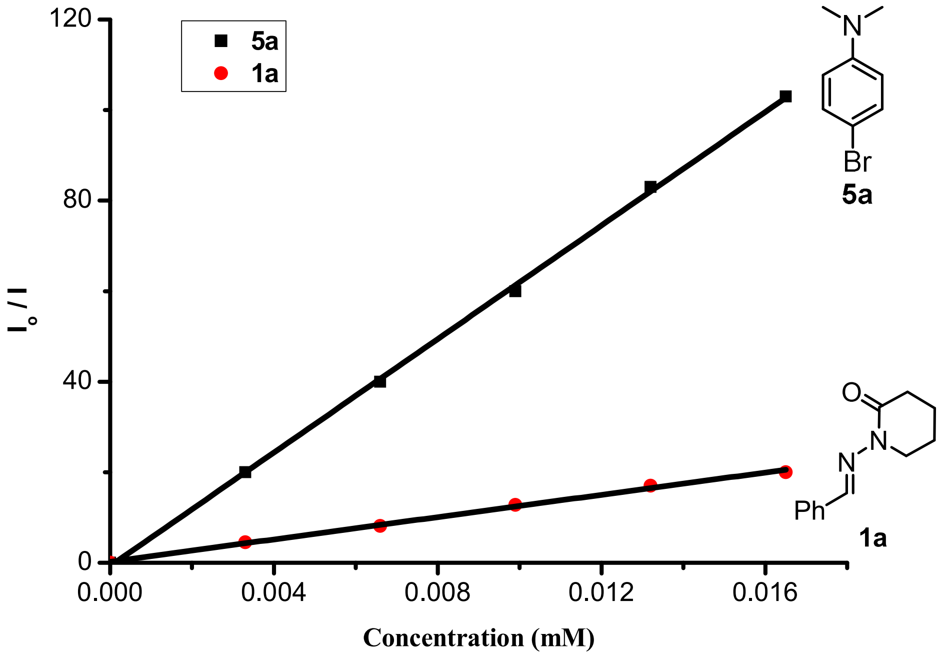

Supplementary Figure 4. Stern-Volmer plots. I_0 and I are respective luminescence intensities in the absence and presence of the indicated concentrations of the corresponding quencher.

5.6 A Radical Addition Mechanism for Asymmetric α-Aminoalkylation of Acyclic Imines Starting from Tertiary Amines

A proposed reaction mechanism is shown in Supplementary Figure 5, which proceeds through the similar enantioselective radical addition process as that of the bifunctional copper catalytic system. The difference is that the iridium-based photosensitizer replaces the copper(II) complex as the redox catalyst.

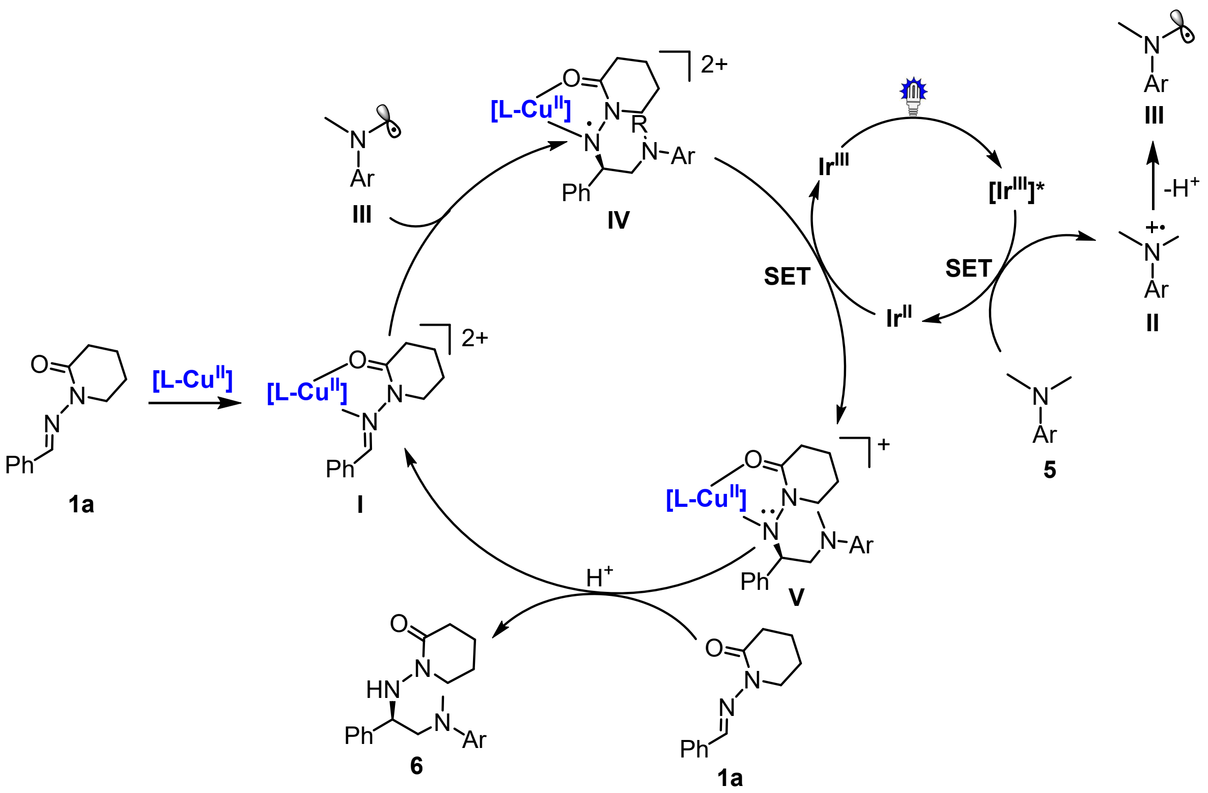

Supplementary Figure 5. A proposed reaction mechanism for the enantioselective α-aminoalkylation enabled by the synergistic combination of an Ir-based photocatalyst and a chiral copper catalyst.

Supplementary Figures

6. Chiral Chromatography

Optical purities of the compounds **3b**, **3d**–**3z** and **6a**–**6h** were determined with a Daicel Chiralpak AD-H, OD-H or IC HPLC column on an Agilent 1260 Series HPLC System. The column temperature was 30 °C and UV-absorption was measured at 220 nm.

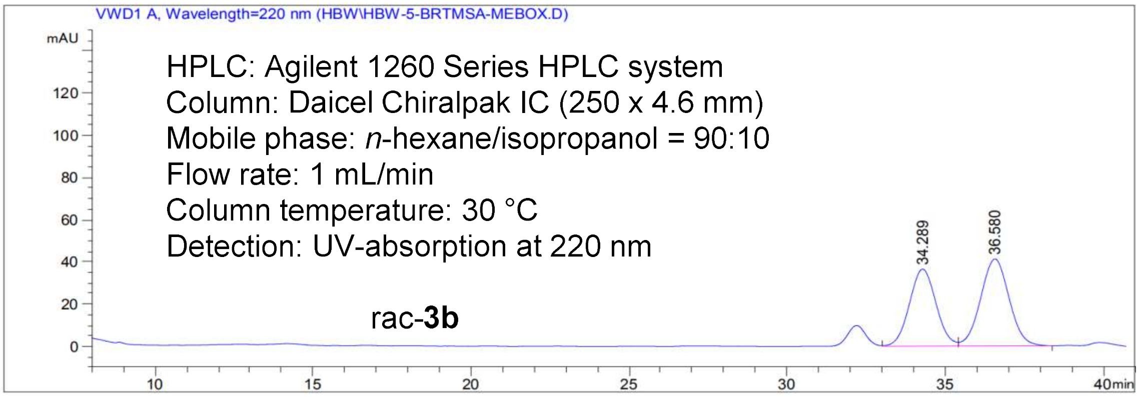

#	[min]		[min]	[mAU*s]	[mAU]	%
1	34.289	VV	0.9171	2130.54443	36.49239	45.2030
2	36.580	VB	0.9703	2582.73608	41.25832	54.7970

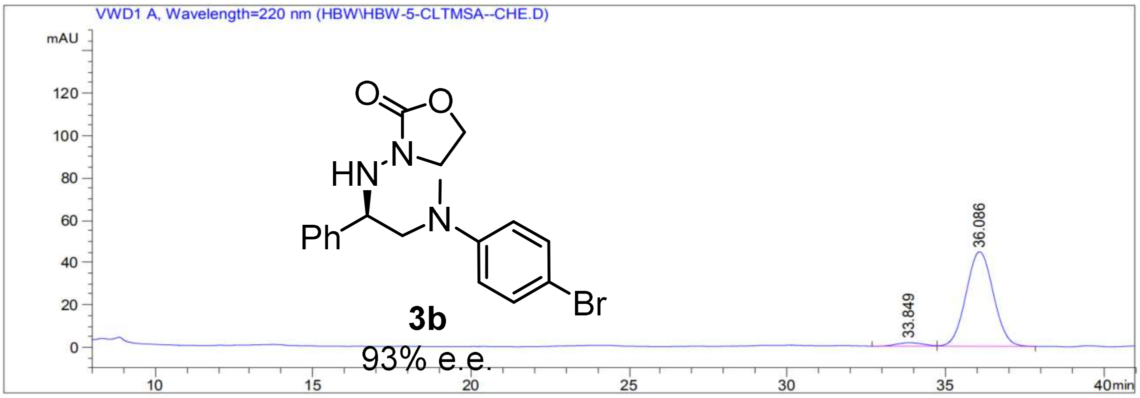

#	[min]		[min]	[mAU*s]	[mAU]	%
1	33.849	BV	0.7594	102.31799	1.76741	3.6569
2	36.086	VB	0.9448	2695.61304	44.74475	96.3431

Supplementary Figure 6. HPLC trace for the racemic reference rac-**3b**, and non-racemic product **3b**.

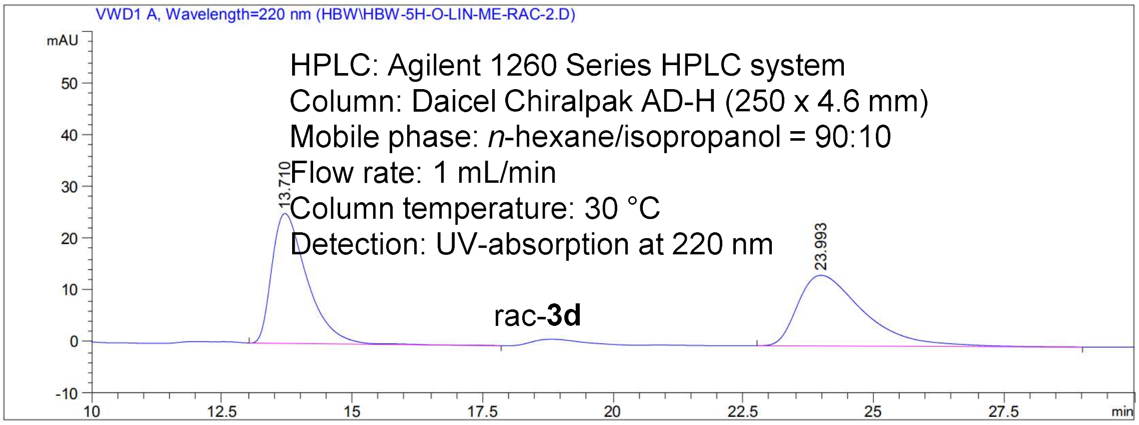

#	[min]		[min]	[mAU*s]	[mAU]	%
1	13.710	BB	0.7128	1181.51477	25.11508	49.7531
2	23.993	BB	1.3394	1193.23975	13.65654	50.2469

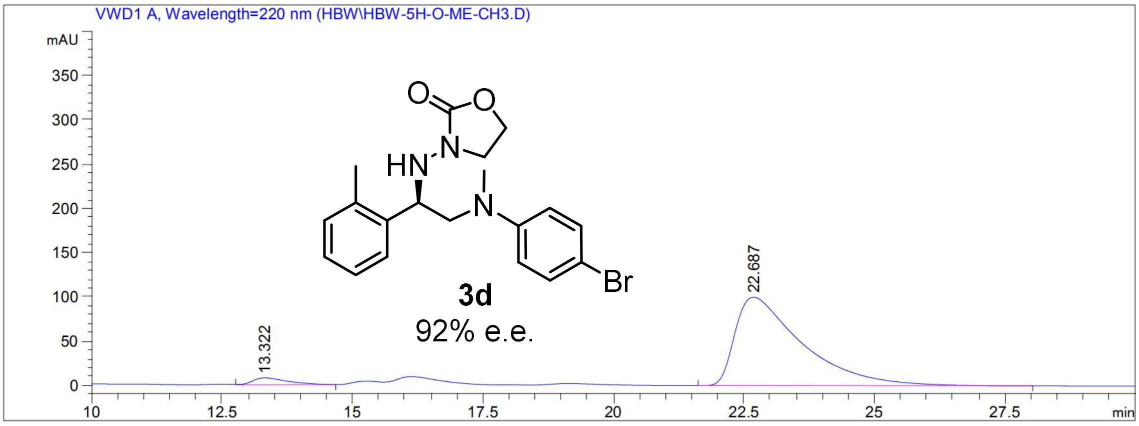

#	[min]		[min]	[mAU*s]	[mAU]	%
1	13.321	BB	0.6987	375.06900	7.72215	4.0337
2	22.686	BB	1.2987	8923.39453	100.03279	95.9663

Supplementary Figure 7. HPLC trace for the racemic reference rac-**3d**, and non-racemic product **3d**.

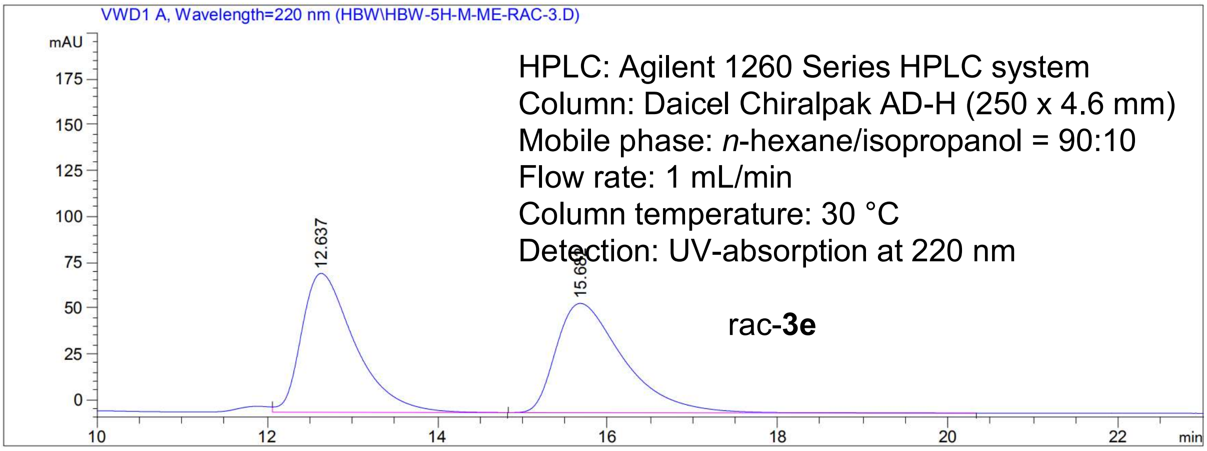

#	[min]		[min]	[mAU*s]	[mAU]	%
1	12.637	VB	0.6596	3295.97754	75.76087	50.0798
2	15.682	BB	0.8346	3285.47583	59.65369	49.9202

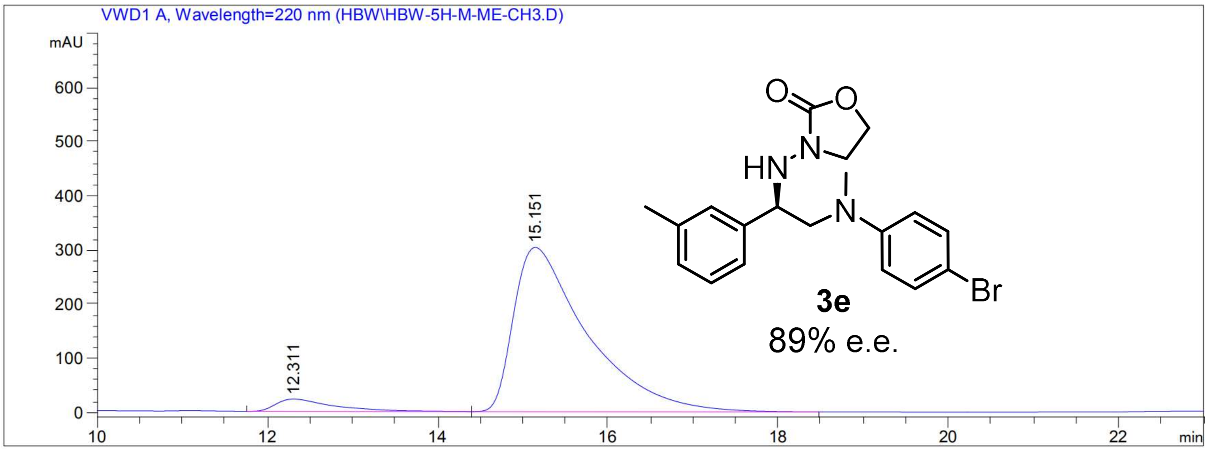

#	[min]		[min]	[mAU*s]	[mAU]	%
1	12.309	BV	0.6884	1081.44238	22.84127	5.6022
2	15.150	VB	0.8723	1.82226e4	303.12854	94.3978

Supplementary Figure 8. HPLC trace for the racemic reference rac-**3e**, and non-racemic product **3e**.

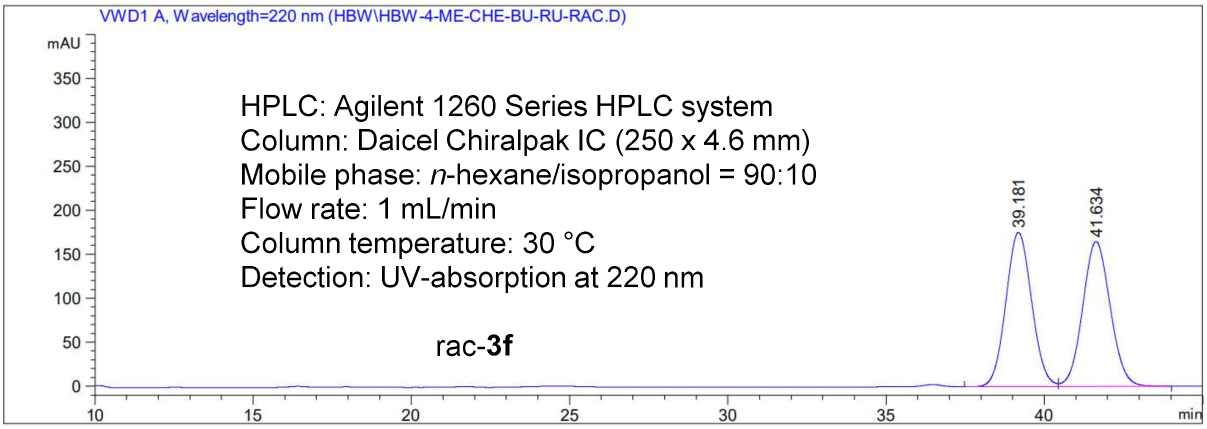

#	[min]		[min]	[mAU*s]	[mAU]	%
1	39.181	BV	0.8848	9949.73340	175.39093	49.9327
2	41.634	VB	0.9432	9976.54492	164.80795	50.0673

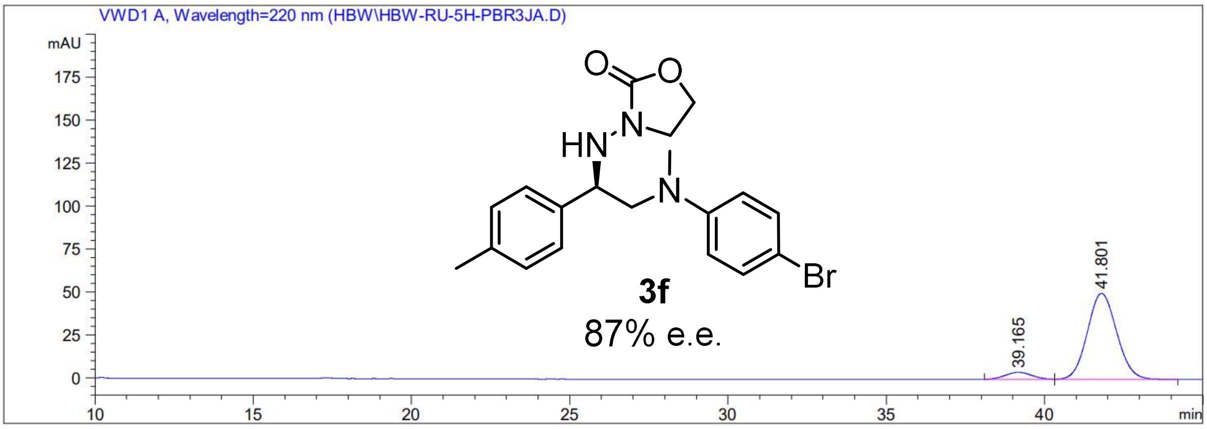

#	[min]		[min]	[mAU*s]	[mAU]	%
1	39.165	PM R	0.9505	231.49629	4.05924	6.7048
2	41.801	BB	0.9947	3221.17944	49.99678	93.2952

Supplementary Figure 9. HPLC trace for the racemic reference rac-**3f**, and non-racemic product **3f**.

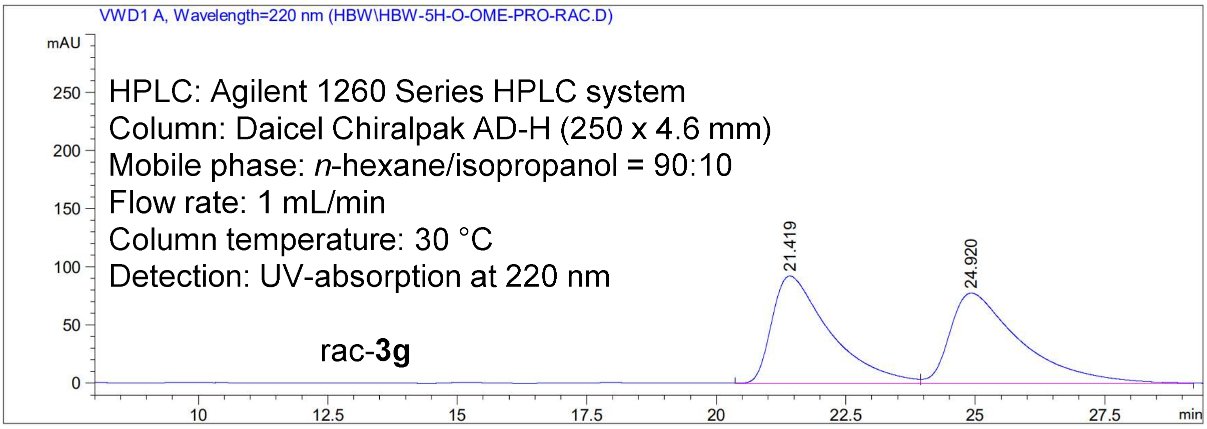

#	[min]		[min]	[mAU*s]	[mAU]	%
1	21.419	VV	1.1428	7165.05664	92.18572	49.3046
2	24.920	MF R	1.5803	7367.18311	77.69814	50.6954

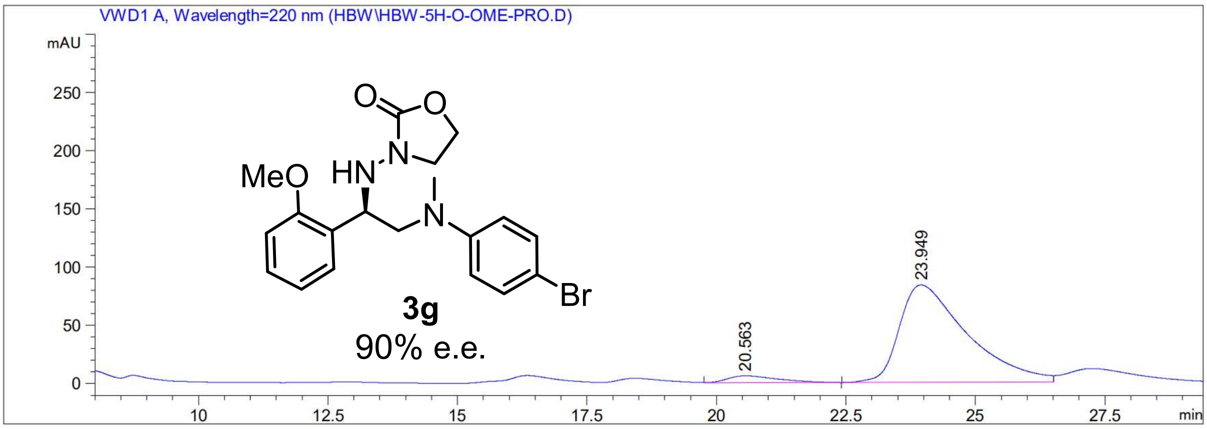

#	[min]		[min]	[mAU*s]	[mAU]	%
1	20.563	BB	0.9605	385.95557	5.76198	5.2520
2	23.948	BB	1.2590	6962.82568	81.63978	94.7480

Supplementary Figure 10. HPLC trace for the racemic reference rac-**3g**, and non-racemic product **3g**.

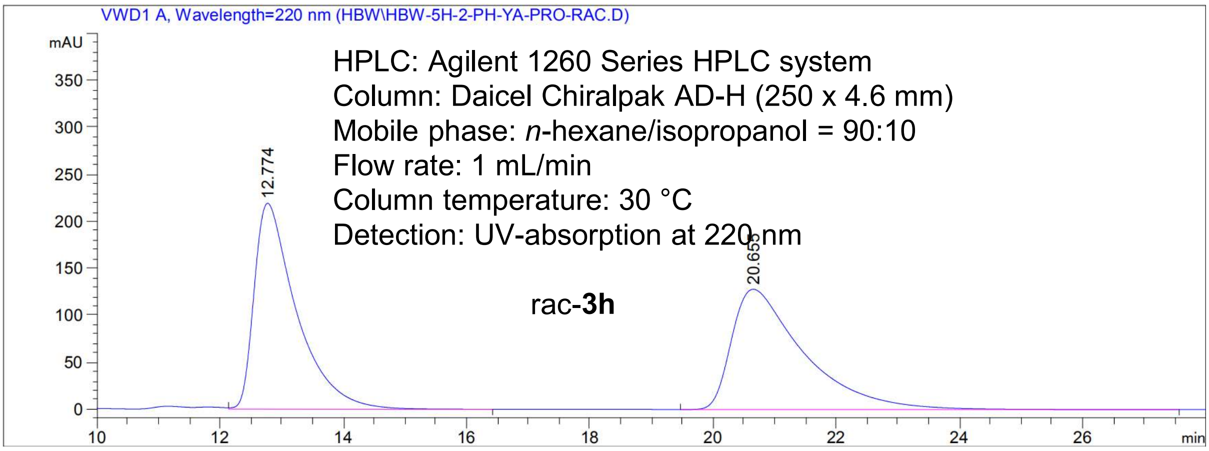

#	[min]		[min]	[mAU*s]	[mAU]	%
1	12.774	VB	0.6964	1.04503e4	219.11697	50.1771
2	20.655	BB	1.1698	1.03765e4	128.00101	49.8229

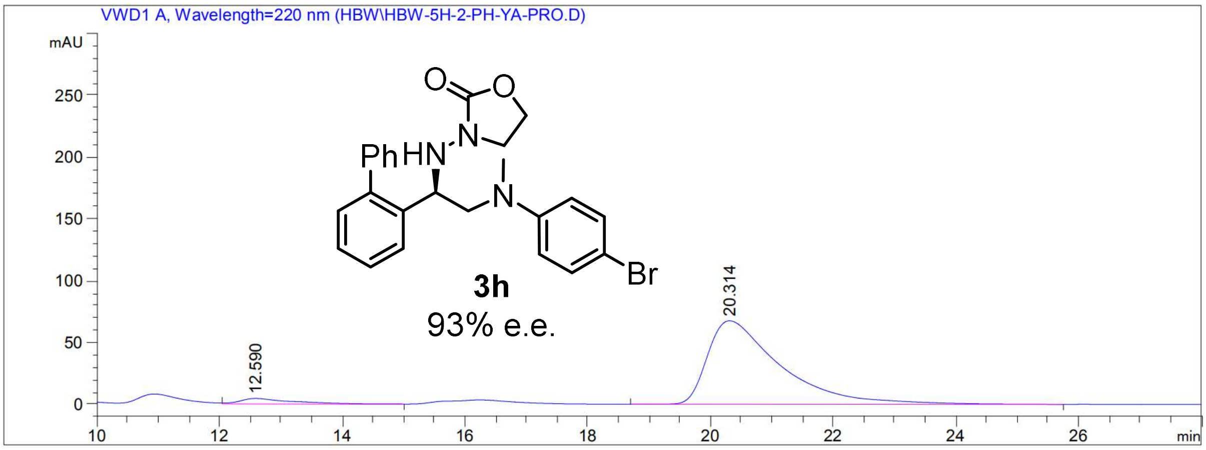

#	[min]		[min]	[mAU*s]	[mAU]	%
1	12.590	BB	0.7175	200.02701	3.86020	3.4851
2	20.313	BB	1.1817	5539.42725	67.41024	96.5149

Supplementary Figure 11. HPLC trace for the racemic reference rac-**3h**, and non-racemic product **3h**.

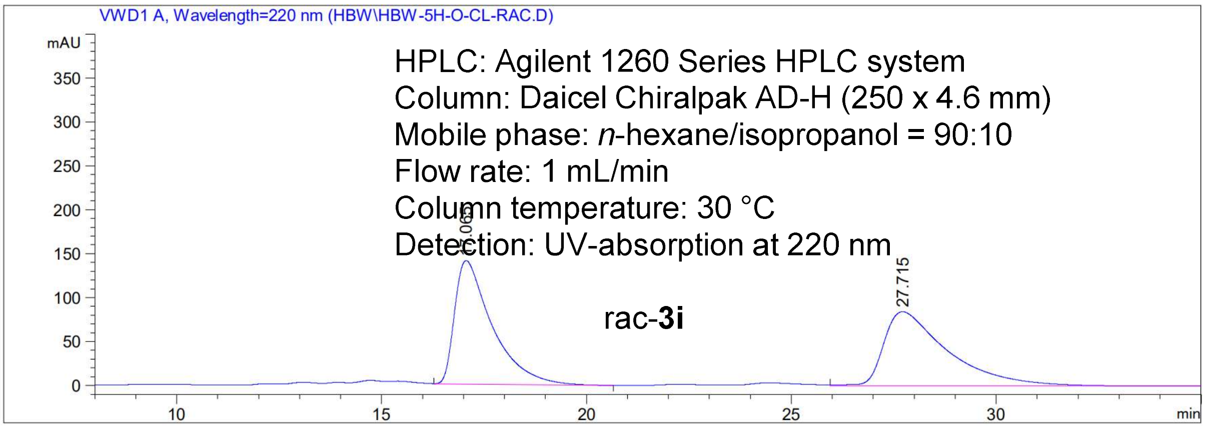

#	[min]		[min]	[mAU*s]	[mAU]	%
1	17.065	BB	0.9268	8943.36328	140.46680	49.4183
2	27.715	VB	1.5622	9153.90820	84.34515	50.5817

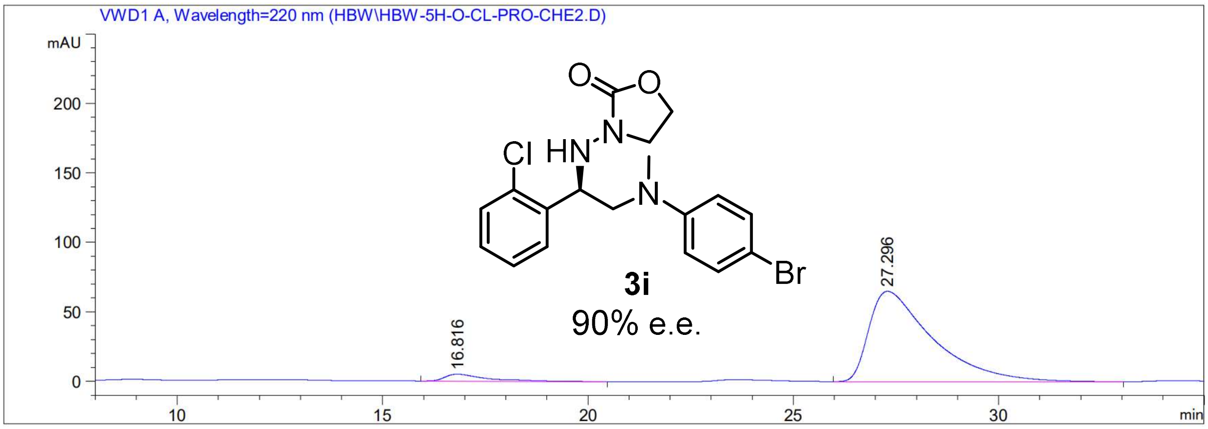

#	[min]		[min]	[mAU*s]	[mAU]	%
1	16.815	BB	0.9505	348.08536	5.00424	4.7330
2	27.295	BB	1.5450	7006.41309	65.02728	95.2670

Supplementary Figure 12. HPLC trace for the racemic reference rac-**3i**, and non-racemic product **3i**.

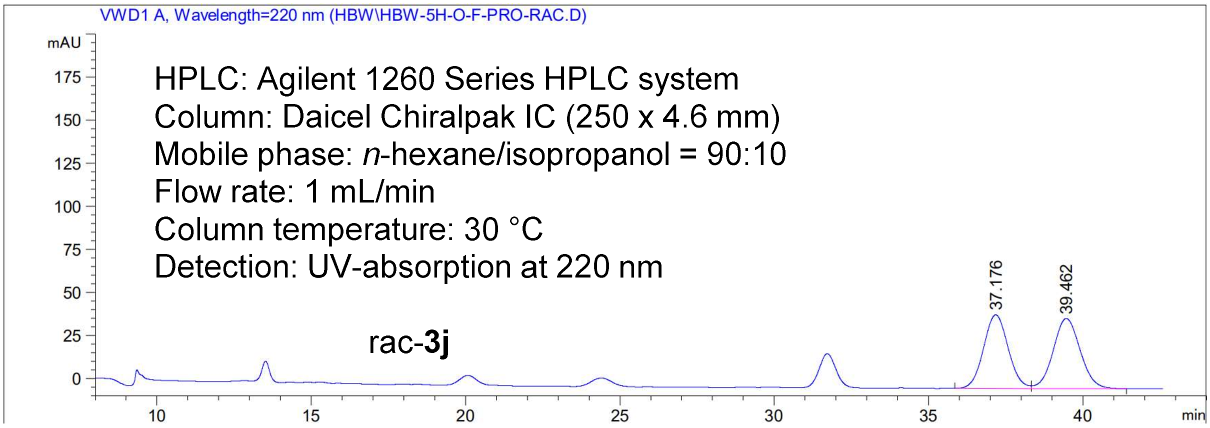

#	[min]		[min]	[mAU*s]	[mAU]	%
1	37.176	BV	0.8625	2369.03662	42.75216	48.8978
2	39.462	VV	0.9423	2475.83813	40.66677	51.1022

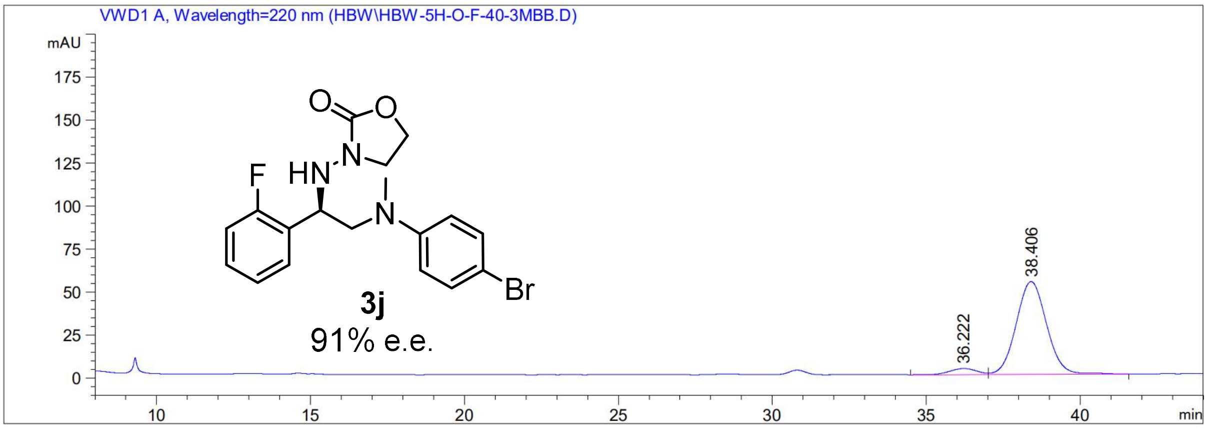

#	[min]		[min]	[mAU*s]	[mAU]	%
1	36.221	BB	0.7490	166.56099	3.05528	4.4238
2	38.406	BB	1.0482	3598.53882	53.40085	95.5762

Supplementary Figure 13. HPLC trace for the racemic reference rac-**3j**, and non-racemic product **3j**.

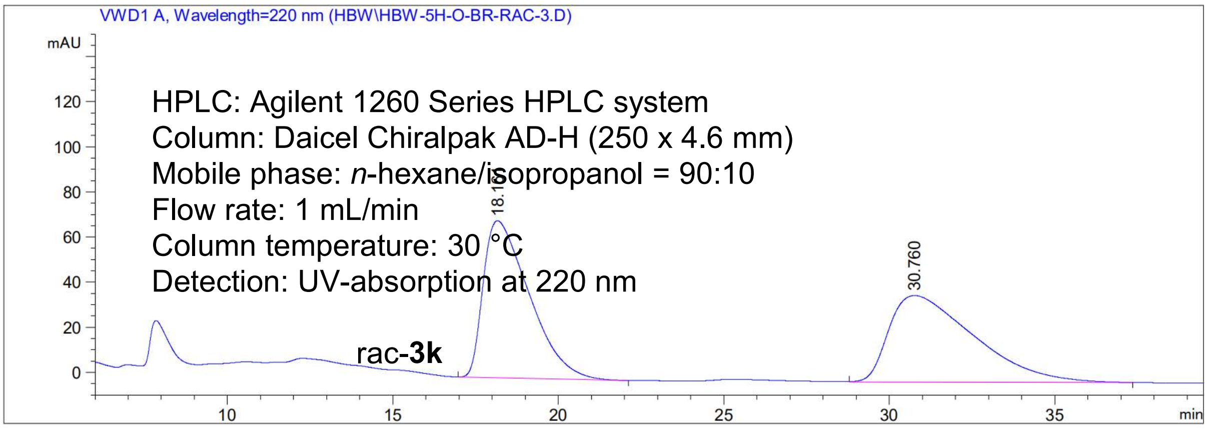

#	[min]		[min]	[mAU*s]	[mAU]	%
1	18.161	BB	1.4574	6714.42432	69.60573	49.4149
2	30.760	MM R	2.9834	6873.42725	38.39848	50.5851

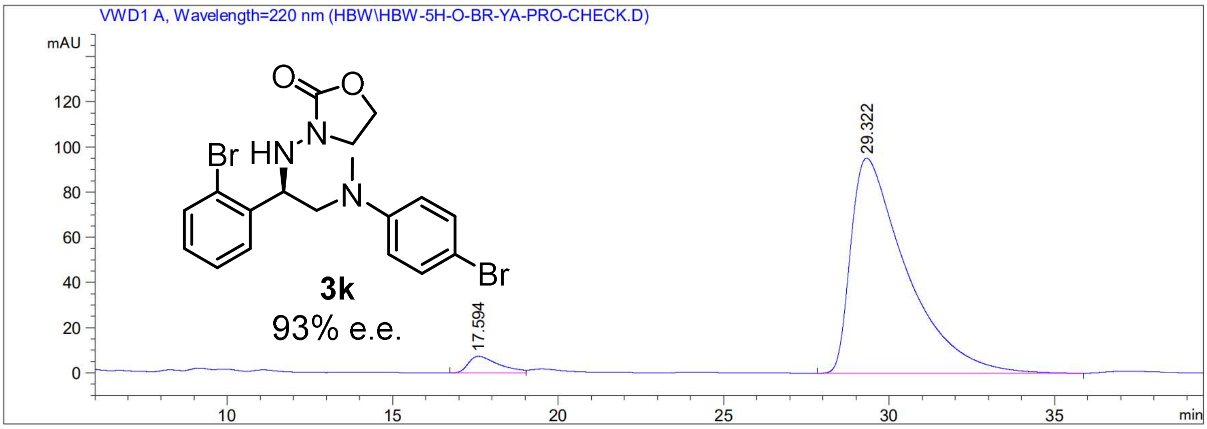

#	[min]		[min]	[mAU*s]	[mAU]	%
1	17.593	BB	0.8346	386.84921	6.88424	3.3652
2	29.322	BB	1.6739	1.11089e4	95.23055	96.6348

Supplementary Figure 14. HPLC trace for the racemic reference rac-**3k**, and non-racemic product **3k**.

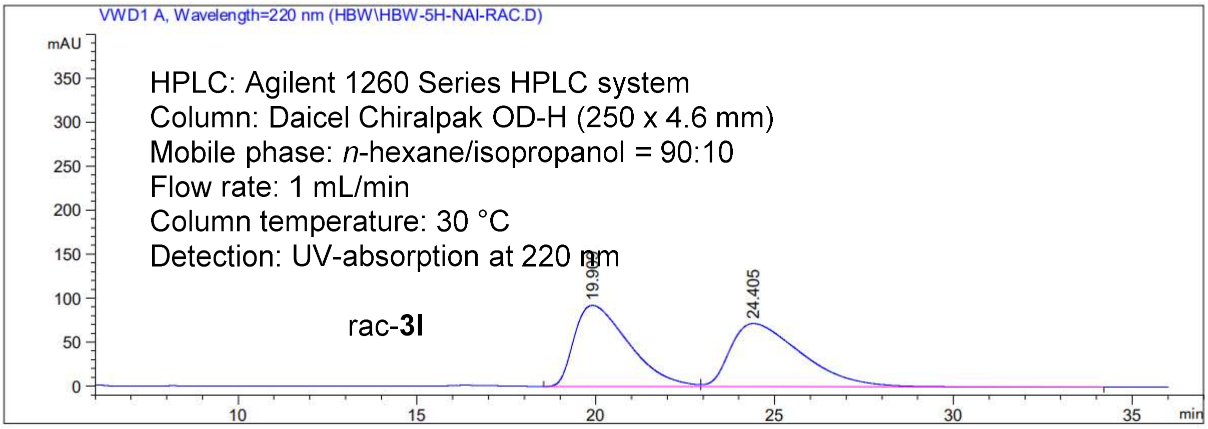

#	[min]		[min]	[mAU*s]	[mAU]	%
1	19.909	BV	1.6409	9738.76758	92.15518	49.5902
2	24.405	VB	2.0893	9899.73828	71.76868	50.4098

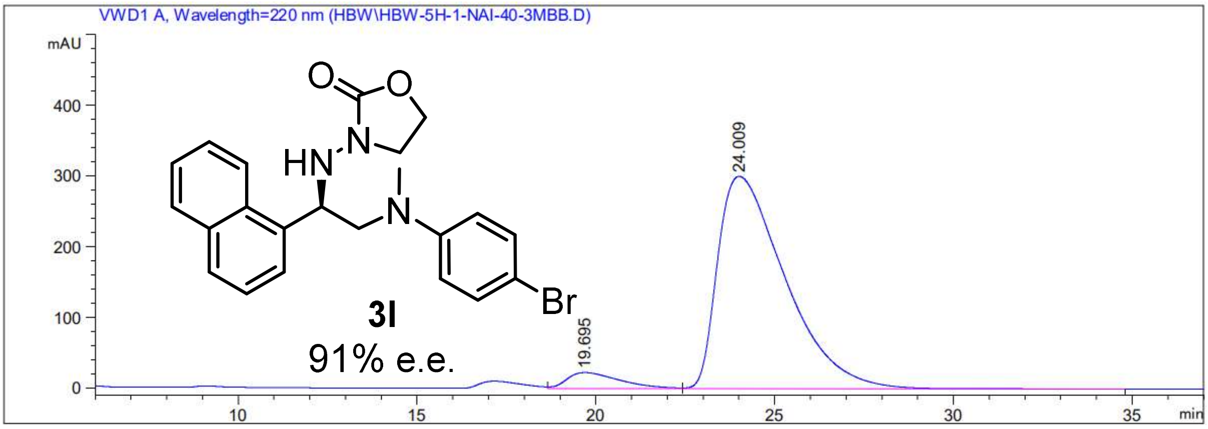

#	[min]		[min]	[mAU*s]	[mAU]	%
1	19.694	BB	1.4070	1973.16199	20.73962	4.7948
2	24.008	BB	1.9402	3.91794e4	299.30252	95.2052

Supplementary Figure 15. HPLC trace for the racemic reference rac-**3l**, and non-racemic product **3l**.

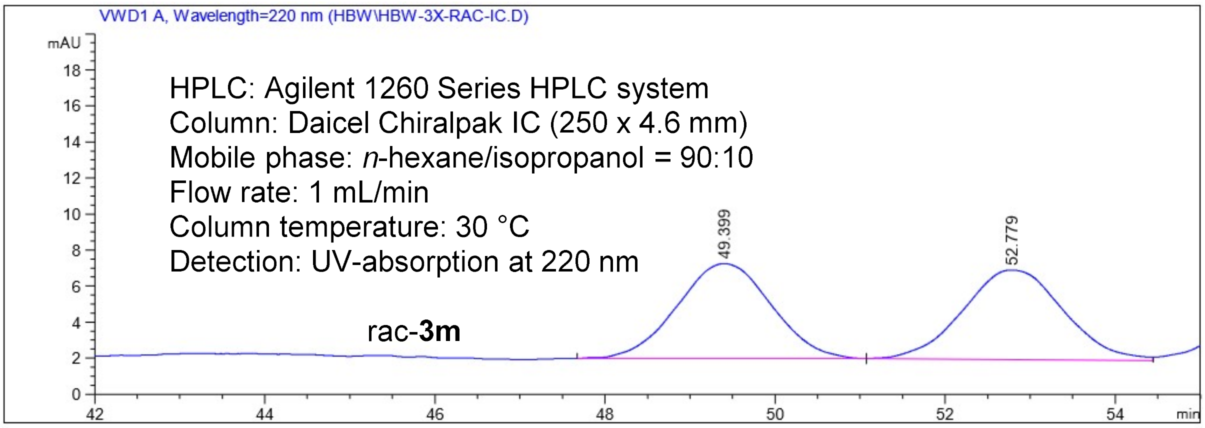

#	[min]		[min]	[mAU*s]	[mAU]	%
1	49.399	BB	1.1536	406.13345	5.27857	49.8988
2	52.779	BV	1.2256	407.78003	4.96813	50.1012

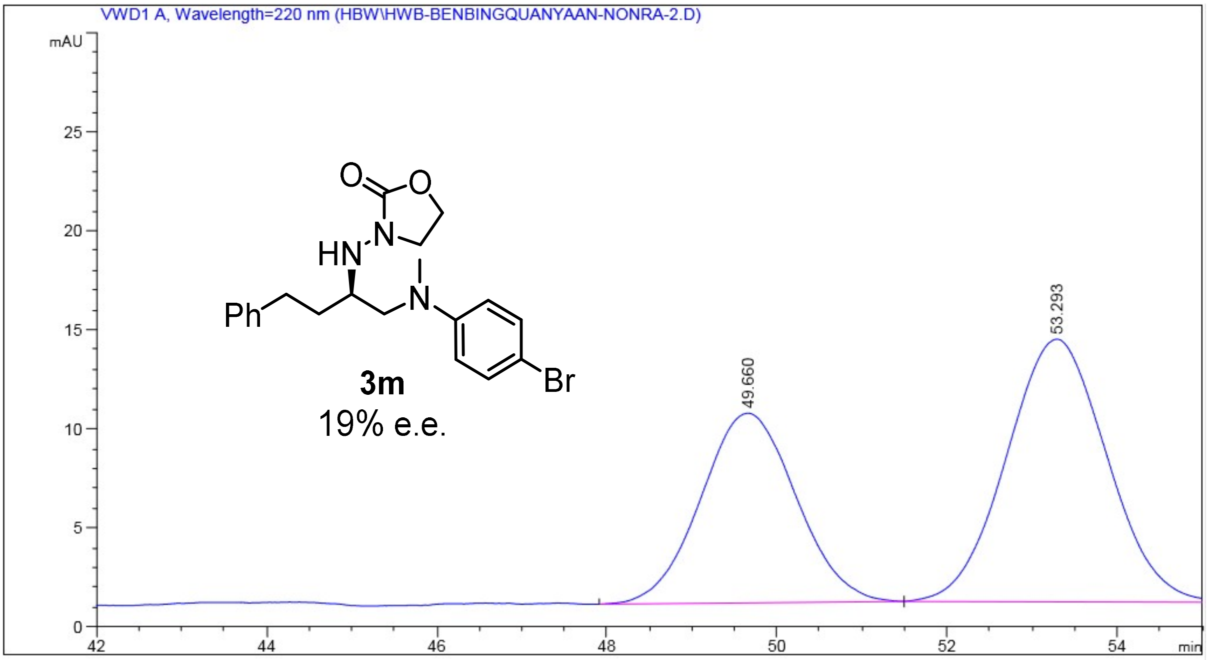

#	[min]		[min]	[mAU*s]	[mAU]	%
1	49.661	BV	1.1949	758.44031	9.62928	40.7088
2	53.293	VB	1.3303	1104.64490	13.33716	59.2912

Supplementary Figure 16. HPLC trace for the racemic reference rac-**3m**, and non-racemic product **3m**.

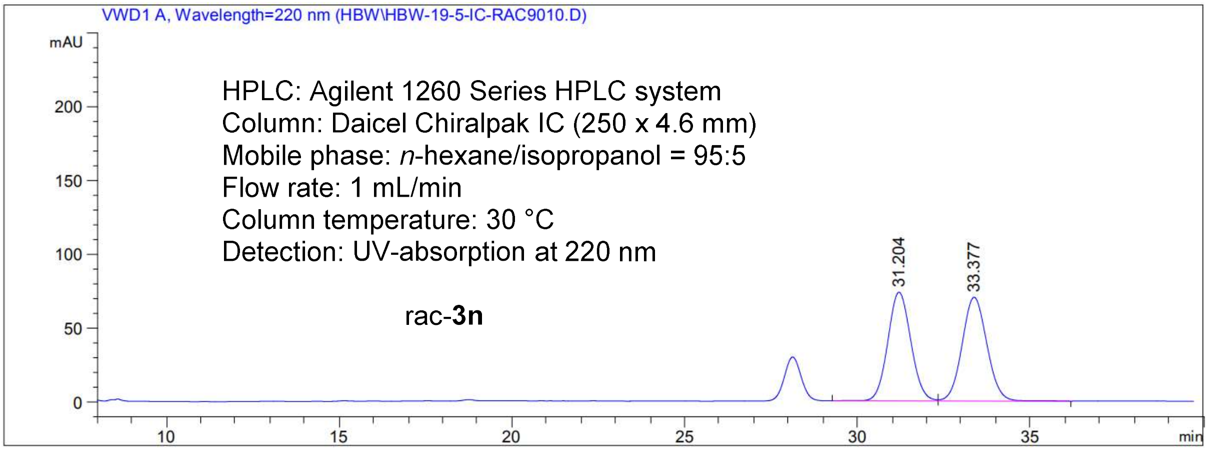

#	[min]		[min]	[mAU*s]	[mAU]	%
1	31.204	BV	0.7248	3406.62964	73.49509	49.3128
2	33.377	VB	0.7743	3501.58081	70.15302	50.6872

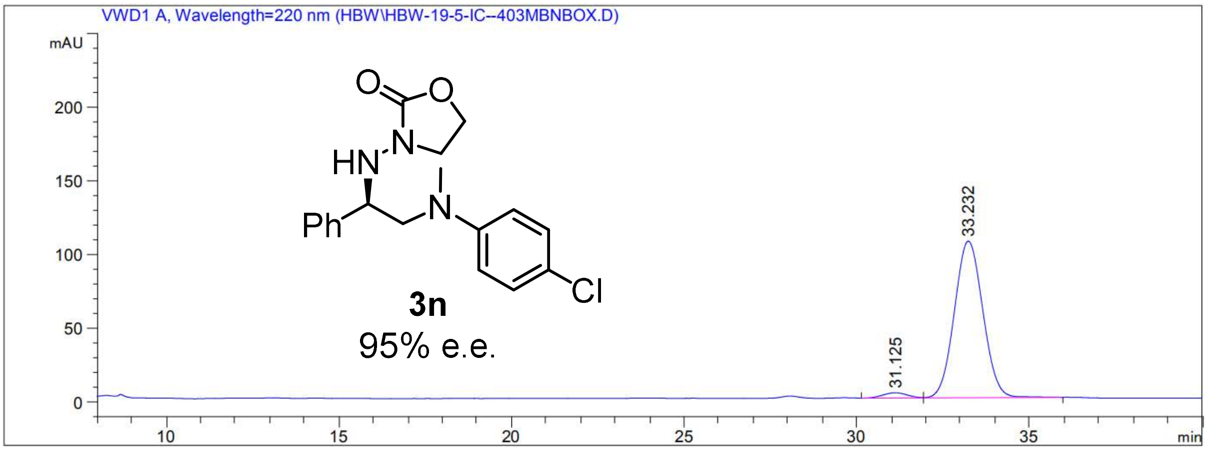

#	[min]		[min]	[mAU*s]	[mAU]	%
1	31.127	BB	0.6818	163.75175	3.43131	2.6380
2	33.232	BB	0.8949	6043.72461	105.88246	97.3620

Supplementary Figure 17. HPLC trace for the racemic reference rac-**3n**, and non-racemic product **3n**.

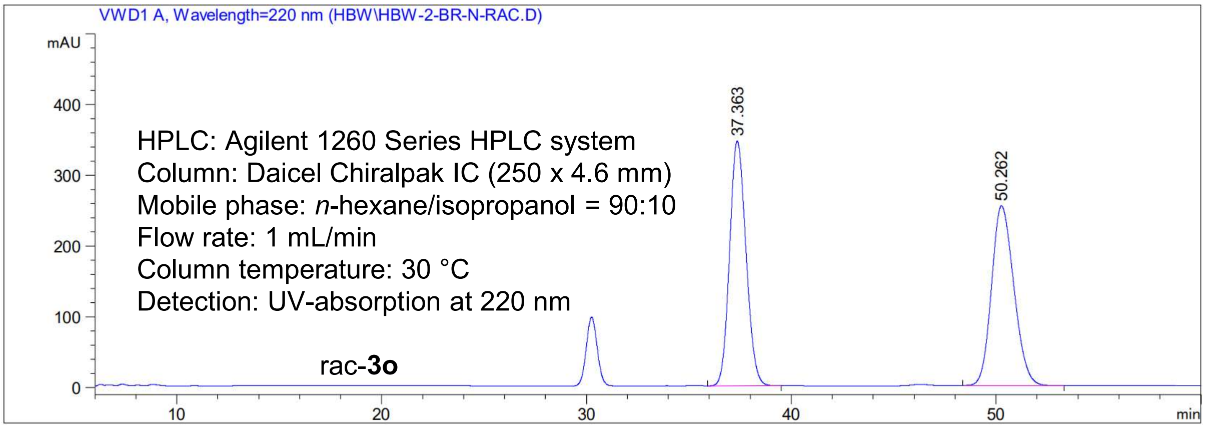

#	[min]		[min]	[mAU*s]	[mAU]	%
1	37.363	BB	0.8711	1.92525e4	346.06049	49.9692
2	50.262	BB	1.1958	1.92762e4	253.88684	50.0308

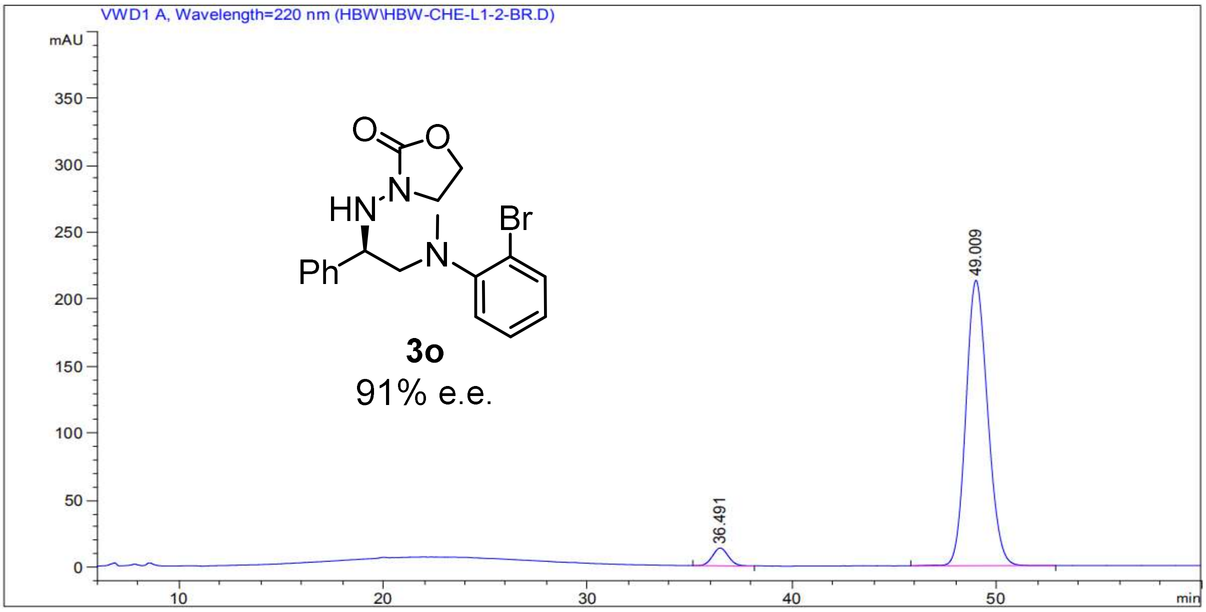

#	[min]		[min]	[mAU*s]	[mAU]	%
1	36.491	BV	0.8378	714.75299	13.23968	4.3517
2	49.009	BB	1.1484	1.57098e4	213.01544	95.6483

Supplementary Figure 18. HPLC trace for the racemic reference rac-**3o**, and non-racemic product **3o**

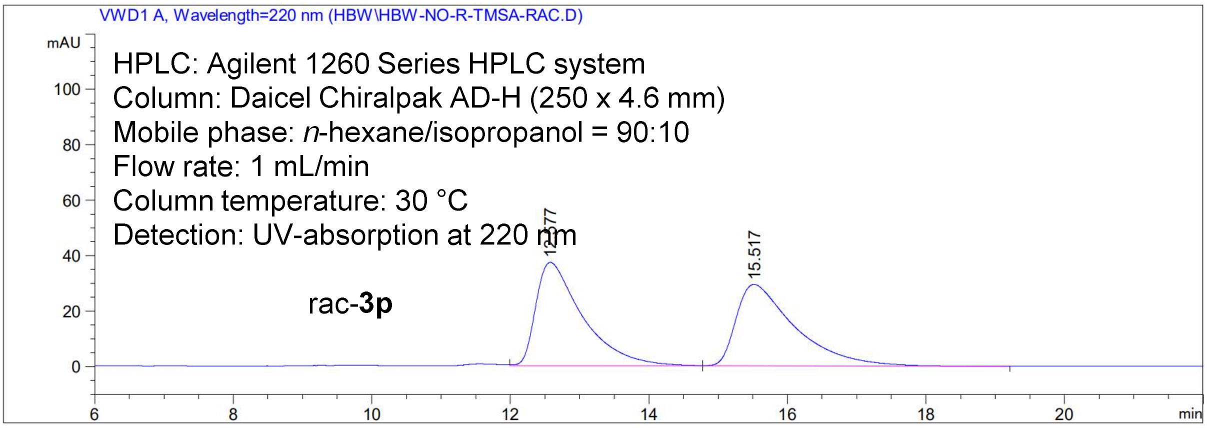

#	[min]		[min]	[mAU*s]	[mAU]	%
1	12.577	VB	0.6856	1743.80420	37.29095	50.2943
2	15.517	BB	0.8614	1723.39575	29.33713	49.7057

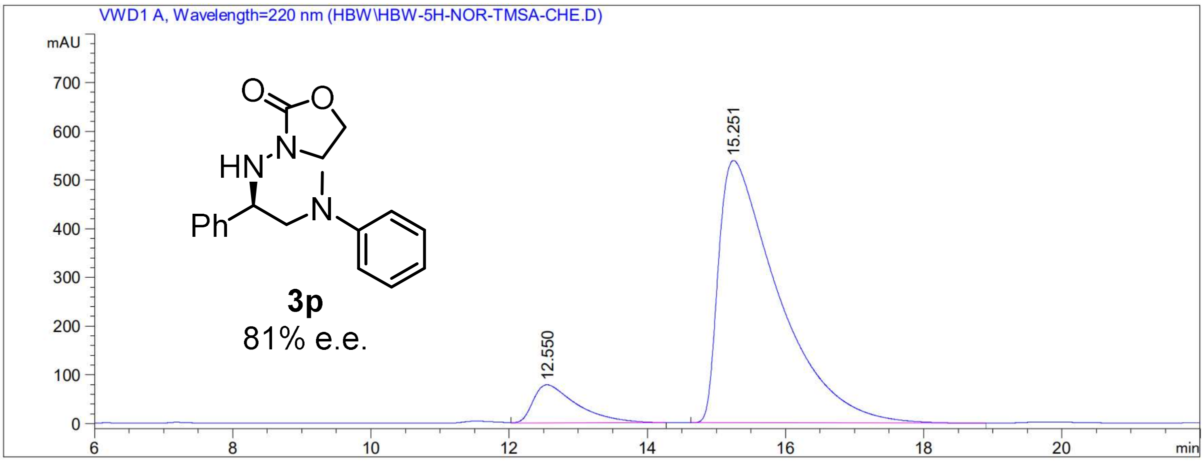

#	[min]		[min]	[mAU*s]	[mAU]	%
1	12.550	VV	0.6458	3448.94751	78.32016	9.6089
2	15.251	BB	0.8790	3.24443e4	538.41760	90.3911

Supplementary Figure 19. HPLC trace for the racemic reference rac-**3p**, and non-racemic product **3p**.

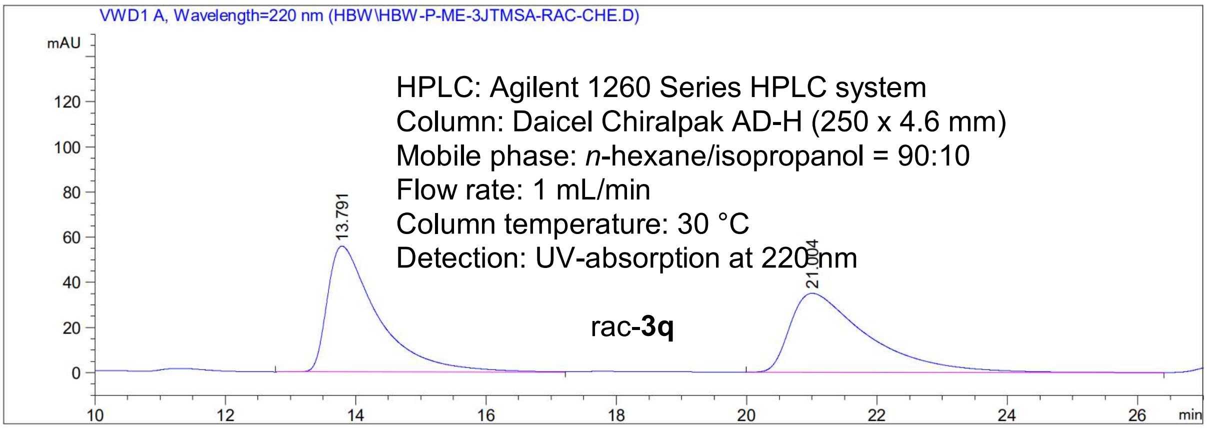

#	[min]		[min]	[mAU*s]	[mAU]	%
1	13.791	BV	0.7764	2984.23926	55.75867	51.0989
2	21.004	BB	1.1936	2855.89014	34.94110	48.9011

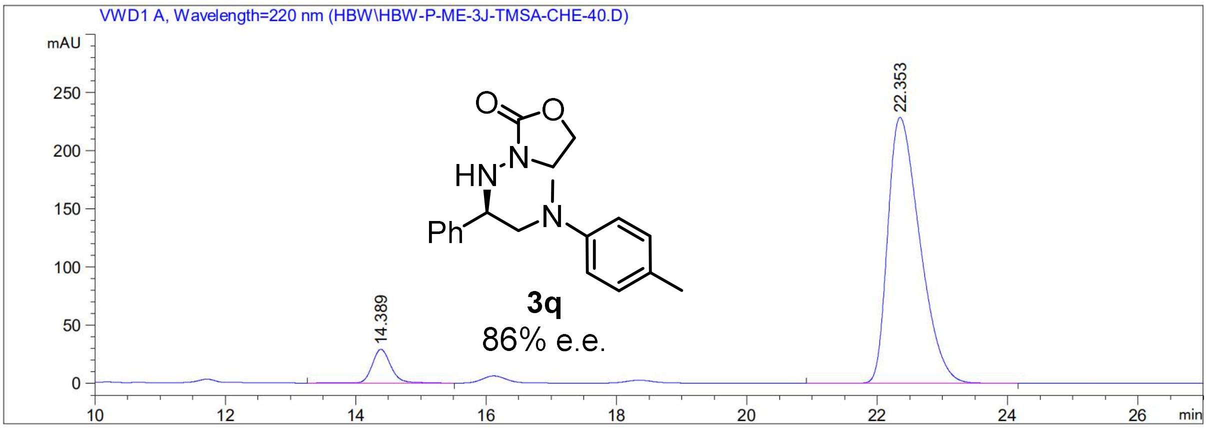

#	[min]		[min]	[mAU*s]	[mAU]	%
1	14.389	BB	0.3029	569.65540	28.90503	6.8126
2	22.352	BB	0.5282	7792.16797	228.27707	93.1874

Supplementary Figure 20. HPLC trace for the racemic reference rac-**3q**, and non-racemic product **3q**.

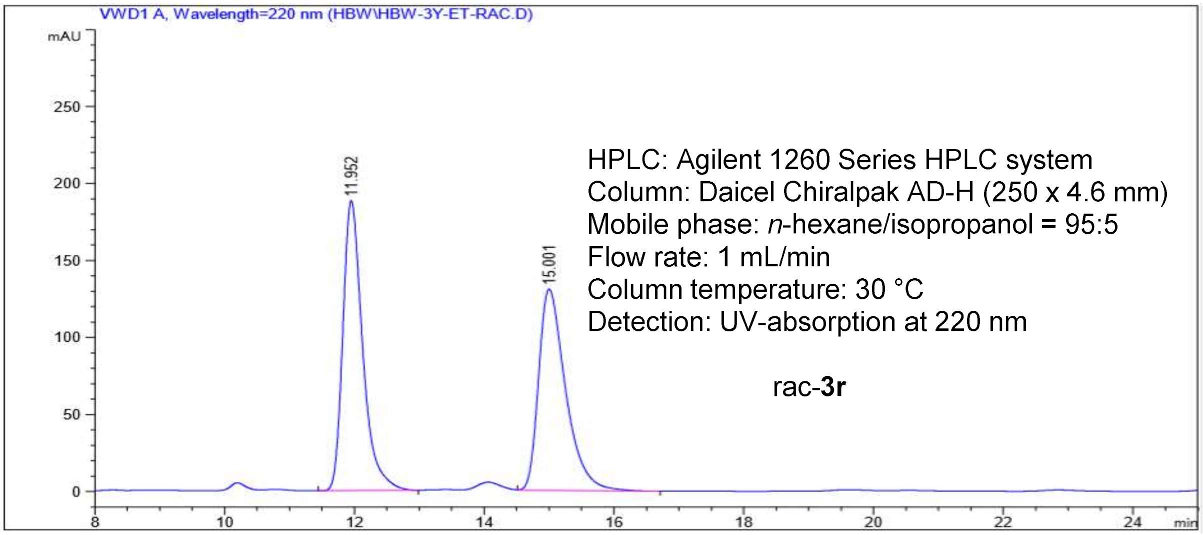

#	[min]		[min]	[mAU*s]	[mAU]	%
1	11.952	BB	0.3223	3988.67041	188.19398	51.3467
2	15.001	VB	0.4381	3779.44189	130.81346	48.6533

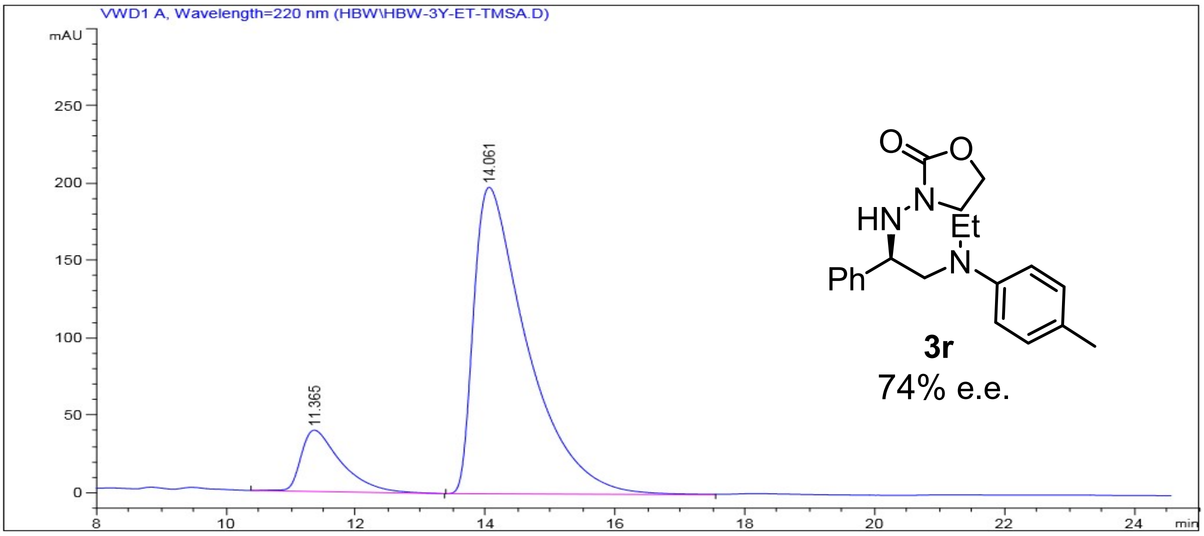

#	[min]		[min]	[mAU*s]	[mAU]	%
1	11.365	BB	0.6332	1703.28235	39.64595	13.0464
2	14.061	BV	0.8448	1.13522e4	198.04500	86.9536

Supplementary Figure 21. HPLC trace for the racemic reference rac-**3r**, and non-racemic product **3r**.

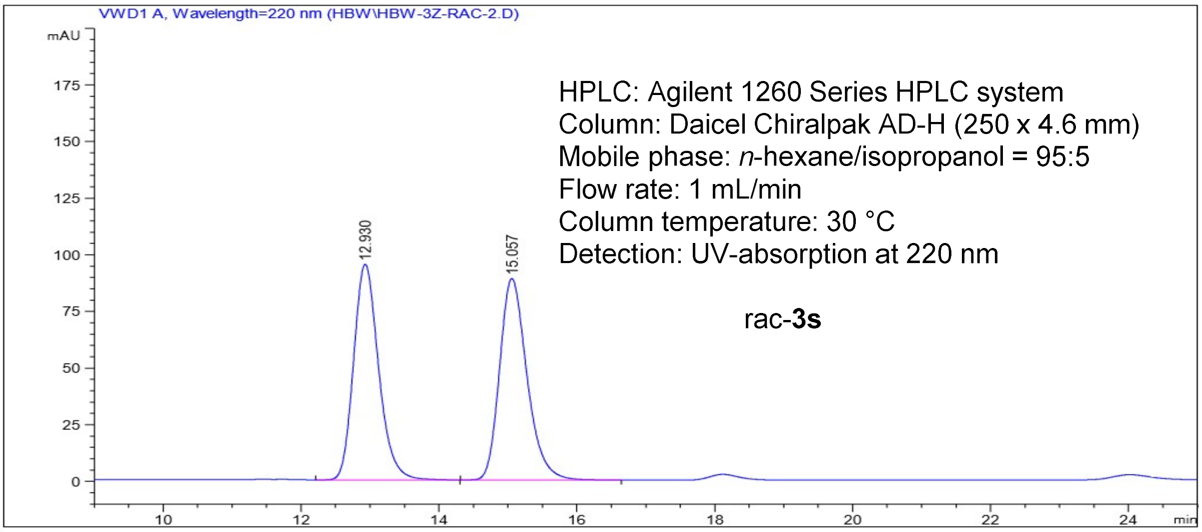

#	[min]		[min]	[mAU*s]	[mAU]	%
1	12.930	BB	0.3896	2395.69751	95.18163	49.4117
2	15.057	BB	0.4260	2452.74780	88.86864	50.5883

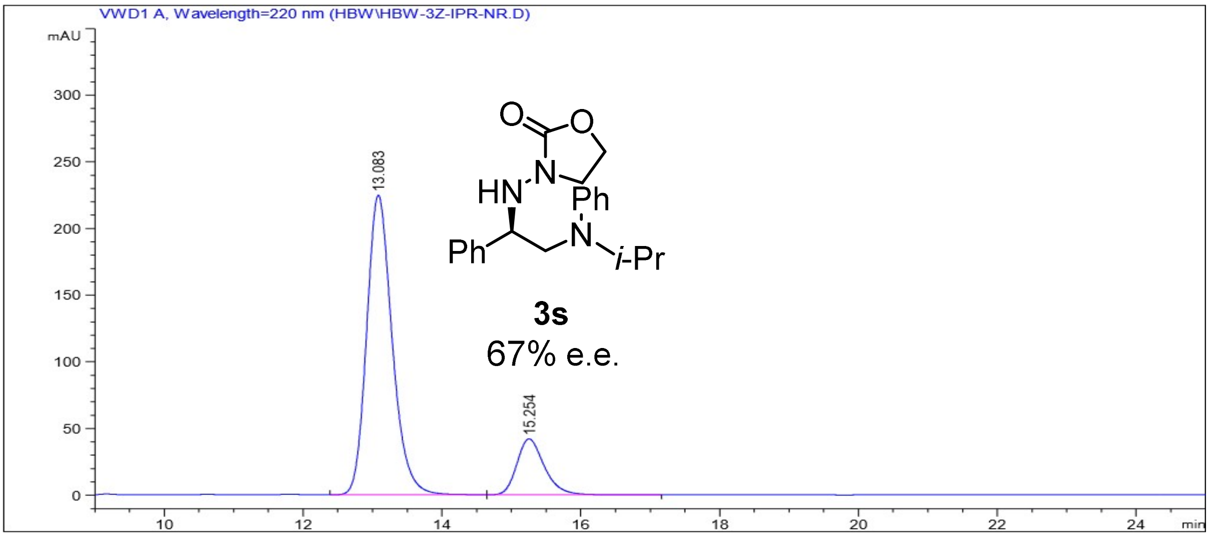

#	[min]		[min]	[mAU*s]	[mAU]	%
1	13.083	VB	0.3937	5731.22607	224.58195	83.2907
2	15.254	BB	0.4202	1149.76611	41.88677	16.7093

Supplementary Figure 22. HPLC trace for the racemic reference rac-**3s**, and non-racemic product **3s**.

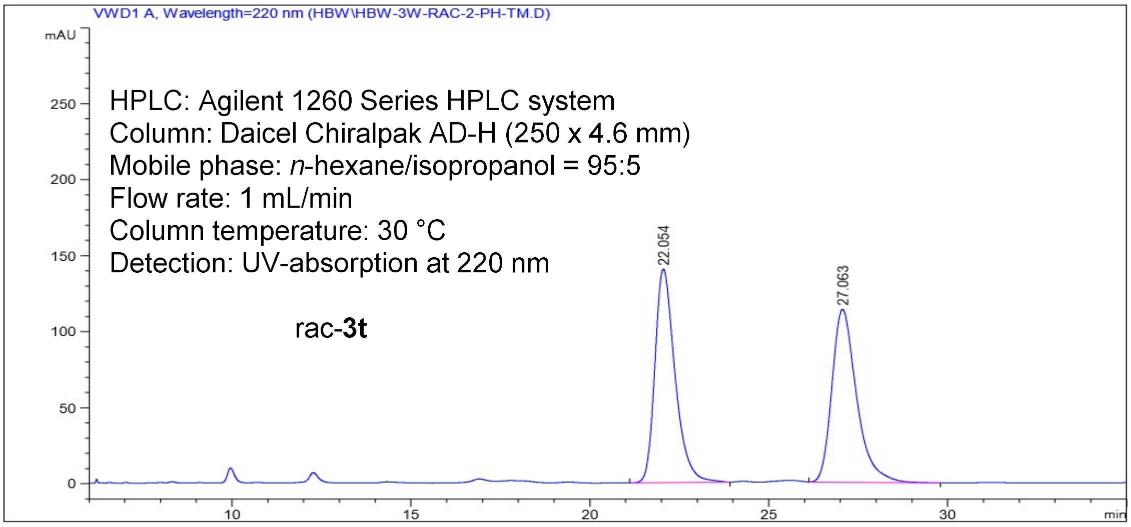

#	[min]		[min]	[mAU*s]	[mAU]	%
1	22.054	BB	0.5949	5503.56445	140.52364	50.1923
2	27.063	BB	0.7303	5461.39355	113.71694	49.8077

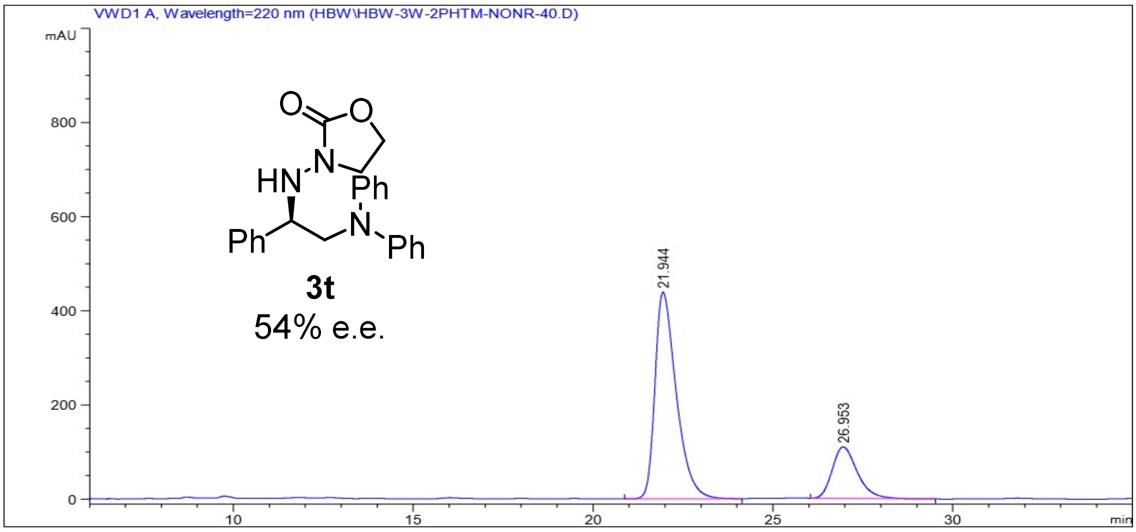

#	[min]		[min]	[mAU*s]	[mAU]	%
1	21.944	BB	0.6244	1.80621e4	438.67062	77.0198
2	26.953	VB	0.7485	5389.14502	110.00878	22.9802

Supplementary Figure 23. HPLC trace for the racemic reference rac-**3t**, and non-racemic product **3t**.

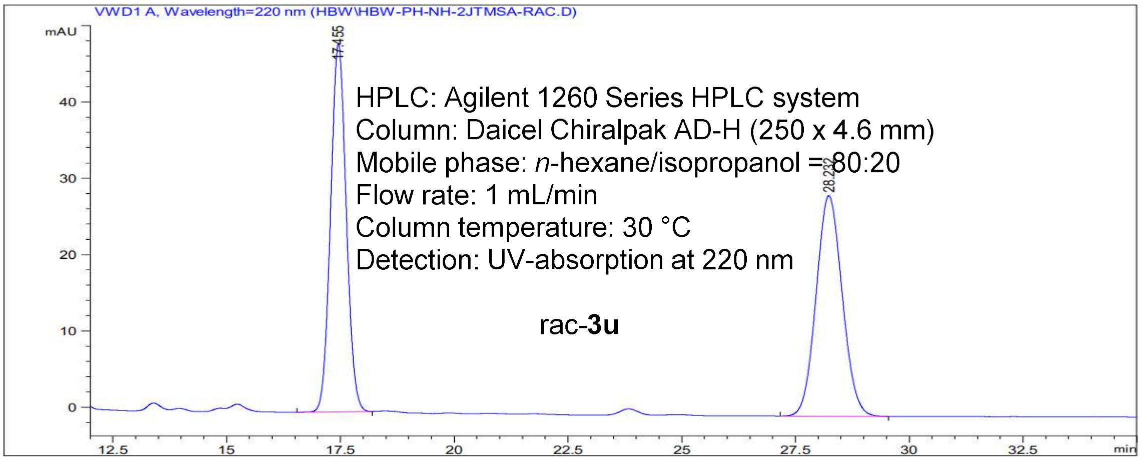

#	[min]		[min]	[mAU*s]	[mAU]	%
1	17.455	BV	0.3772	1164.88037	48.32748	50.2265
2	28.232	BB	0.6222	1154.37585	28.89029	49.7735

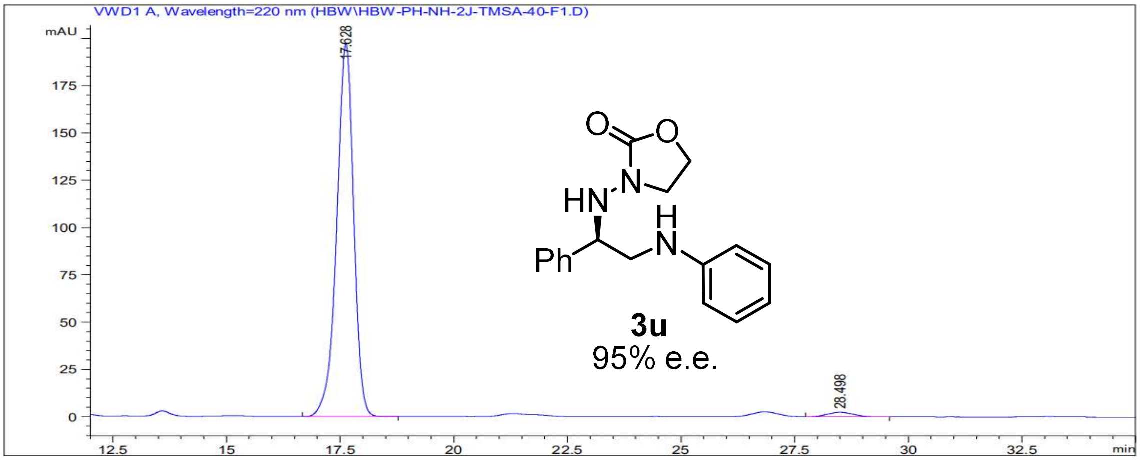

#	[min]		[min]	[mAU*s]	[mAU]	%
1	17.628	MF	0.3516	4161.34326	197.26407	97.7389
2	28.498	BB	0.5897	96.26823	2.40676	2.2611

Supplementary Figure 24. HPLC trace for the racemic reference rac-**3u**, and non-racemic product **3u**.

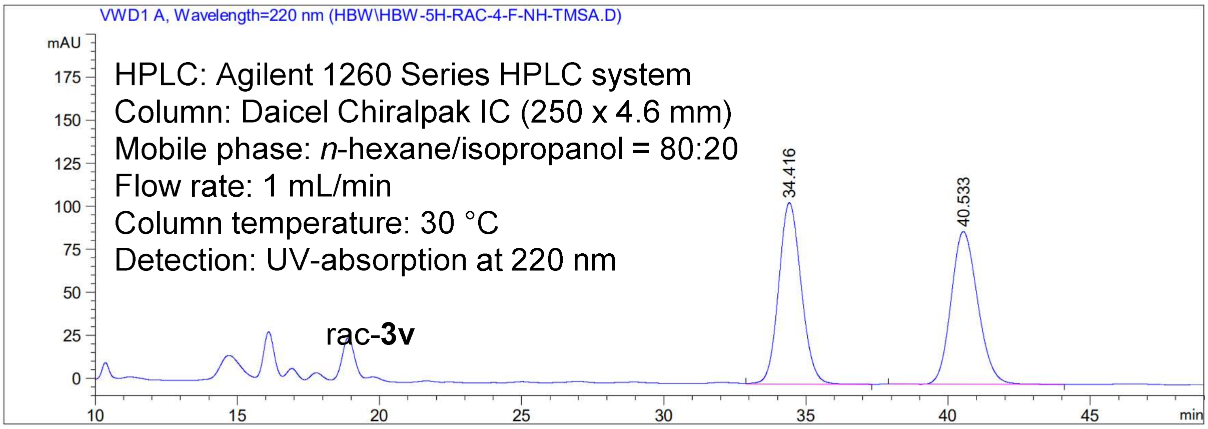

#	[min]		[min]	[mAU*s]	[mAU]	%
1	34.416	VB	0.8590	5799.68066	105.22308	49.9600
2	40.533	BB	1.0189	5808.96191	88.82997	50.0400

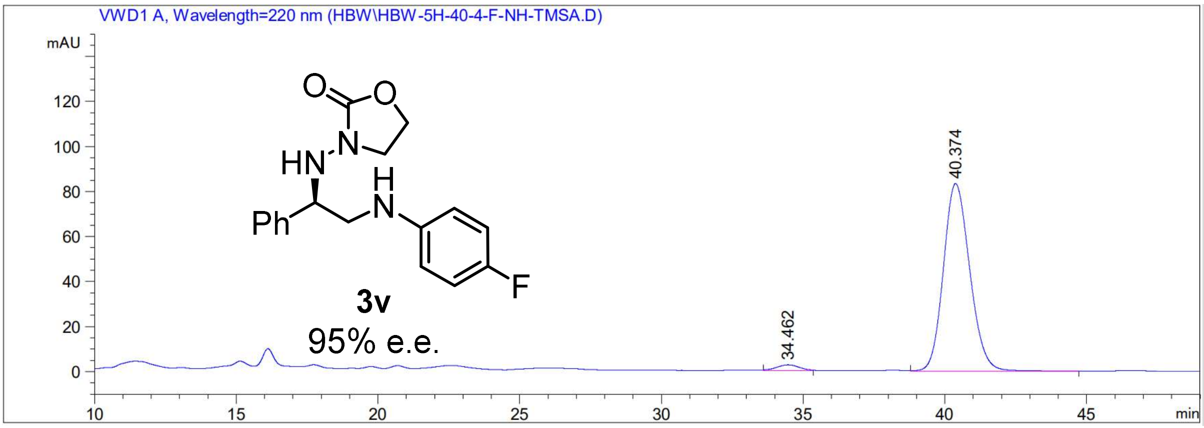

#	[min]		[min]	[mAU*s]	[mAU]	%
1	34.464	BB	0.8760	140.23640	2.50952	2.5504
2	40.374	BB	0.9998	5358.31006	83.15240	97.4496

Supplementary Figure 25. HPLC trace for the racemic reference rac-**3v**, and non-racemic product **3v**.

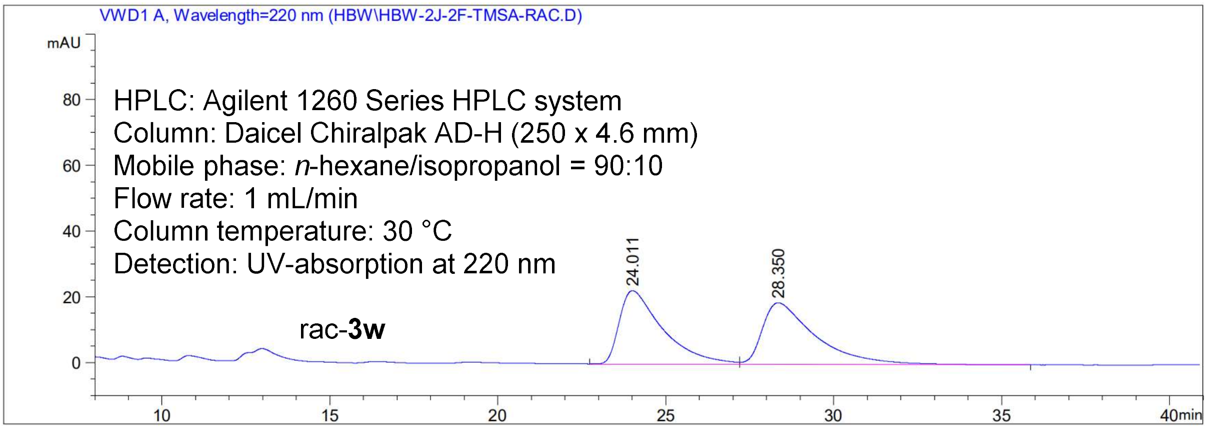

#	[min]		[min]	[mAU*s]	[mAU]	%
1	24.011	BV	1.2763	2006.13599	22.32656	49.3774
2	28.350	VB	1.5765	2056.72339	18.73869	50.6226

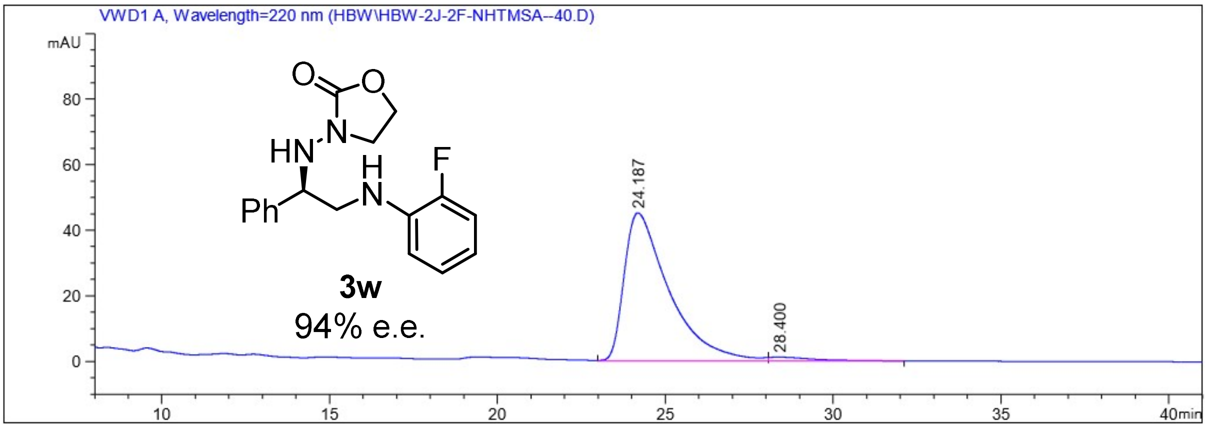

#	[min]		[min]	[mAU*s]	[mAU]	%
1	24.187	MF R	1.5559	4221.60303	45.22081	97.1702
2	28.400	FM R	1.6277	122.94416	1.25887	2.8298

Supplementary Figure 26. HPLC trace for the racemic reference rac-**3w**, and non-racemic product **3w**.

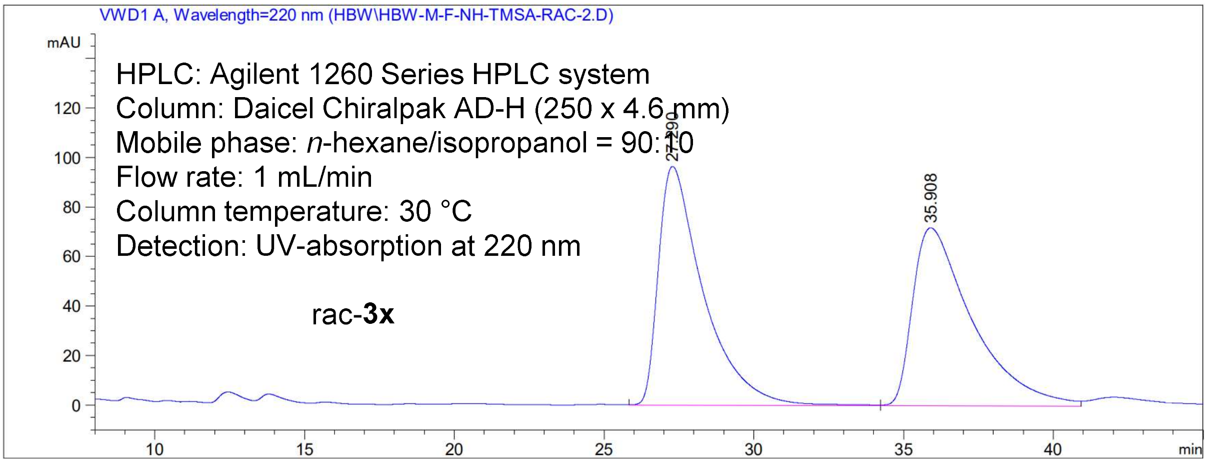

#	[min]		[min]	[mAU*s]	[mAU]	%
1	27.290	VV	1.5100	1.01090e4	96.51479	50.3371
2	35.908	VV	2.0639	9973.61133	72.00781	49.6629

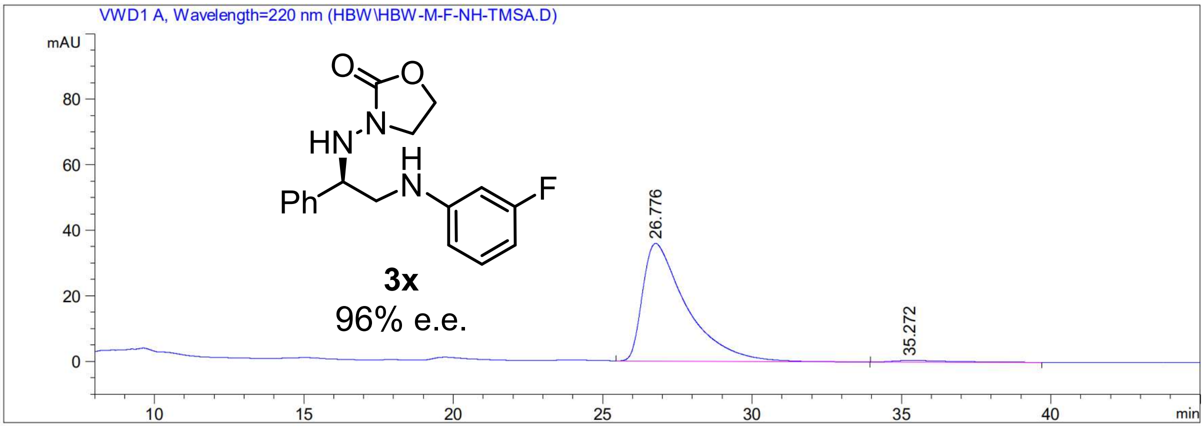

#	[min]		[min]	[mAU*s]	[mAU]	%
1	26.775	BB	1.5103	3663.16919	35.90407	97.8227
2	35.272	MM	2.4867	81.53211	5.46456e-1	2.1773

Supplementary Figure 27. HPLC trace for the racemic reference rac-**3x**, and non-racemic product **3x**.

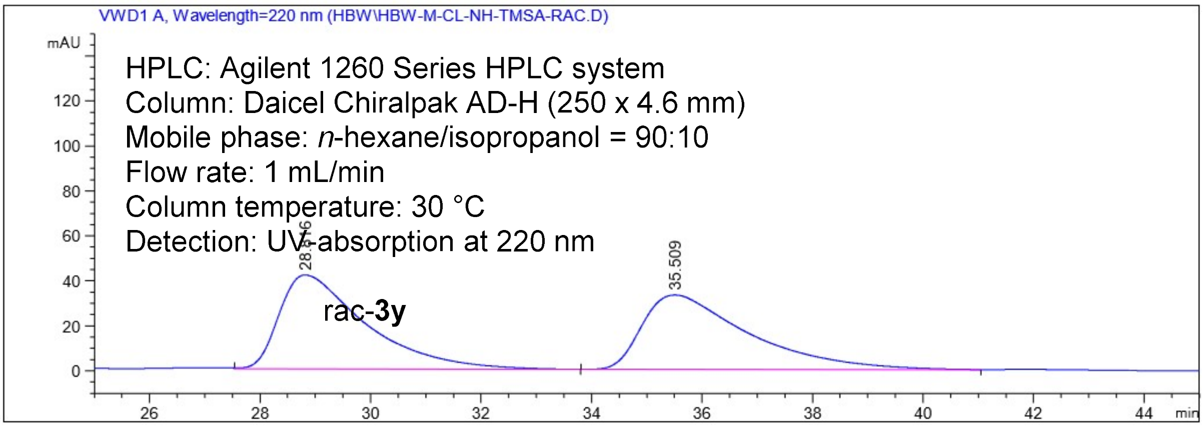

#	[min]		[min]	[mAU*s]	[mAU]	%
1	28.818	VB	1.6032	4539.45264	41.79209	50.2660
2	35.510	BV	2.0016	4491.41602	33.21389	49.7340

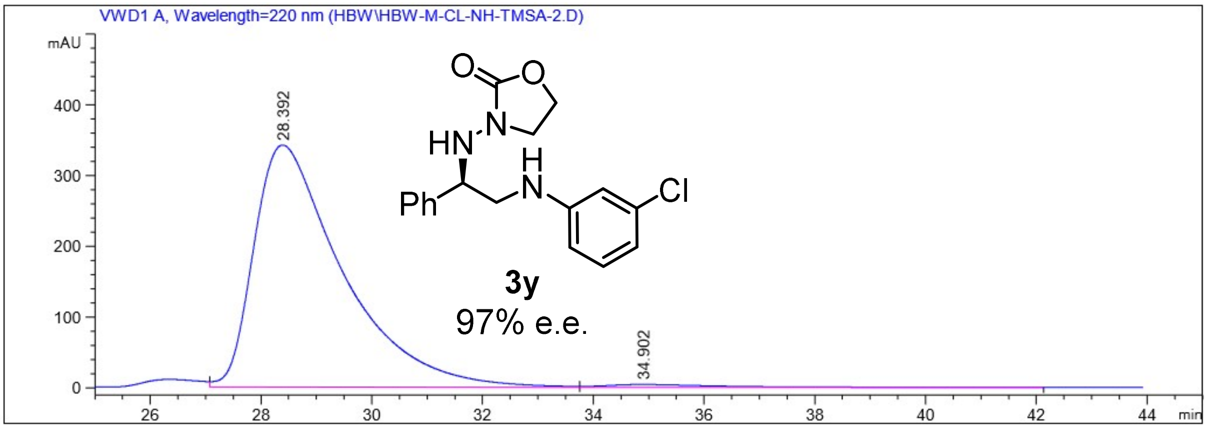

#	[min]		[min]	[mAU*s]	[mAU]	%
1	28.392	VV	1.6420	3.80505e4	342.01102	98.5260
2	34.901	VB	1.9983	569.25323	3.95714	1.4740

Supplementary Figure 28. HPLC trace for the racemic reference rac-**3y**, and non-racemic product **3y**.

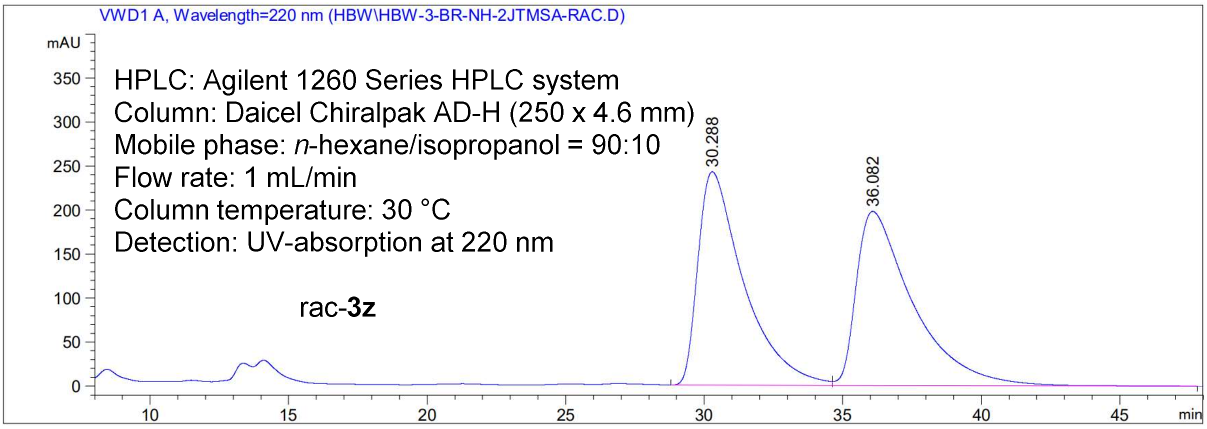

#	[min]		[min]	[mAU*s]	[mAU]	%
1	30.288	BV	1.6707	2.78777e4	242.23253	49.8954
2	36.082	VB	2.0359	2.79946e4	197.71445	50.1046

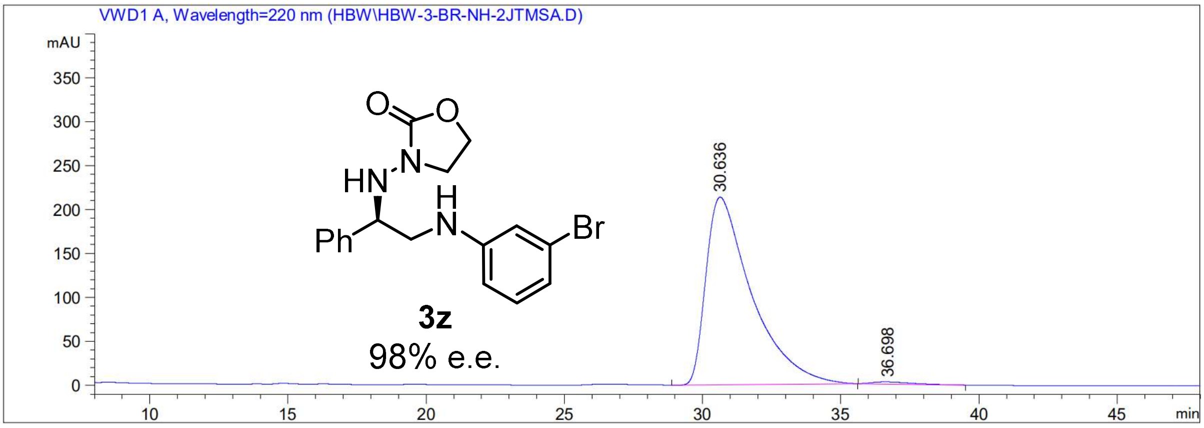

#	[min]		[min]	[mAU*s]	[mAU]	%
1	30.636	MM	1.9229	2.46470e4	213.62297	98.7605
2	36.698	MM	1.9316	309.32391	2.66897	1.2395

Supplementary Figure 29. HPLC trace for the racemic reference rac-**3z**, and non-racemic product **3z**.

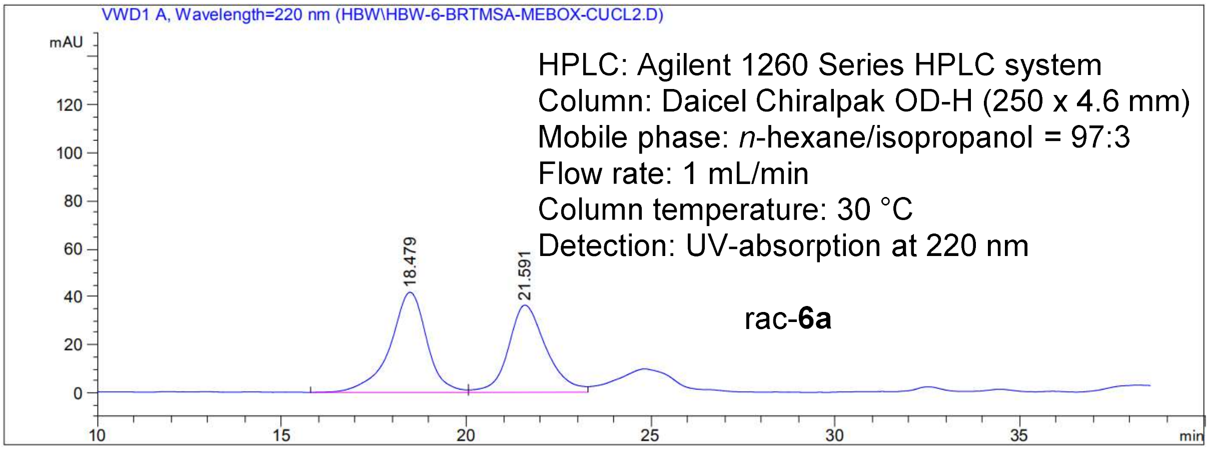

#	[min]		[min]	[mAU*s]	[mAU]	%
1	18.479	BV	1.0298	2881.22461	41.71746	52.7151
2	21.591	VV	1.0618	2584.42554	36.33759	47.2849

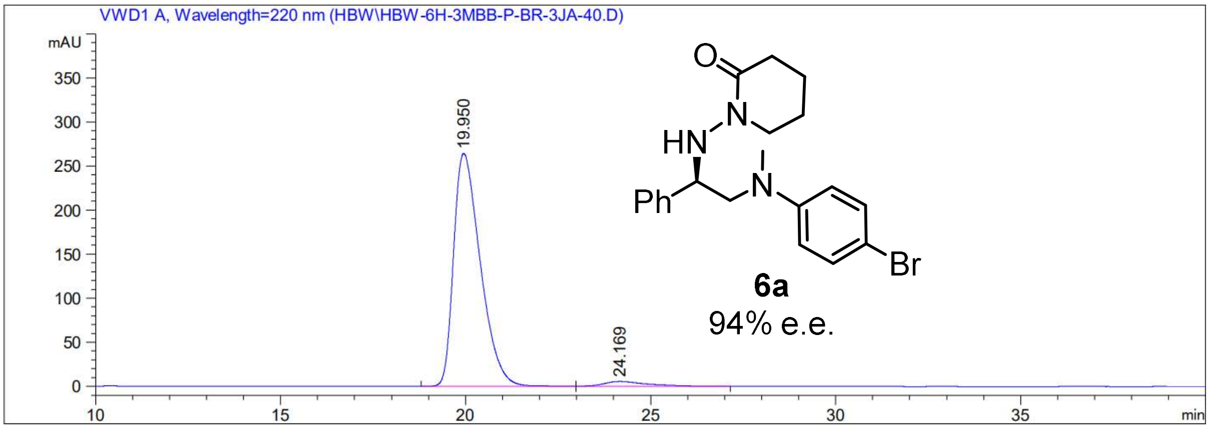

#	[min]		[min]	[mAU*s]	[mAU]	%
1	19.950	BB	0.7716	1.32393e4	264.20740	96.8784
2	24.169	BB	1.0680	426.58948	5.41528	3.1216

Supplementary Figure 30. HPLC trace for the racemic reference rac-**6a**, and non-racemic product **6a**.

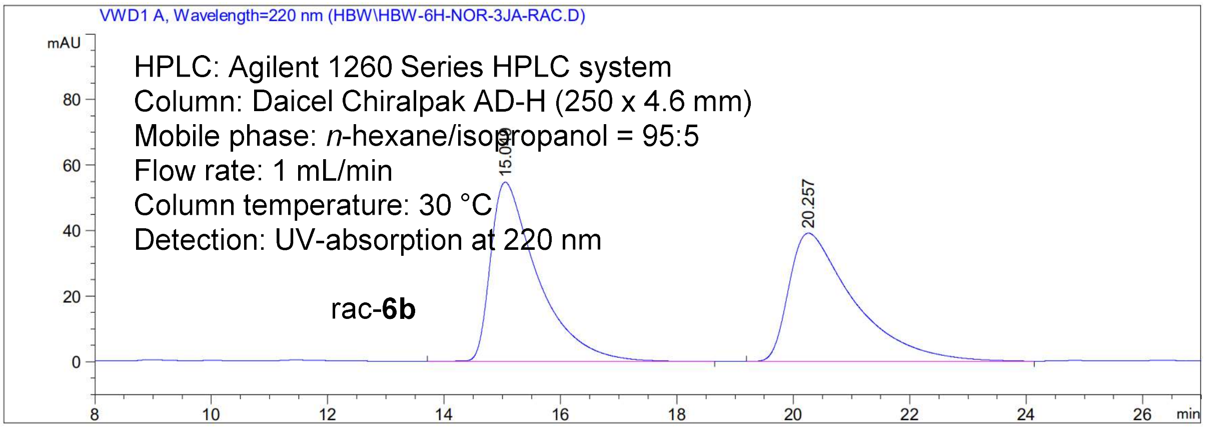

#	[min]		[min]	[mAU*s]	[mAU]	%
1	15.049	BB	0.8213	3082.90796	54.72111	50.3799
2	20.257	BV	1.1291	3036.41187	39.13802	49.6201

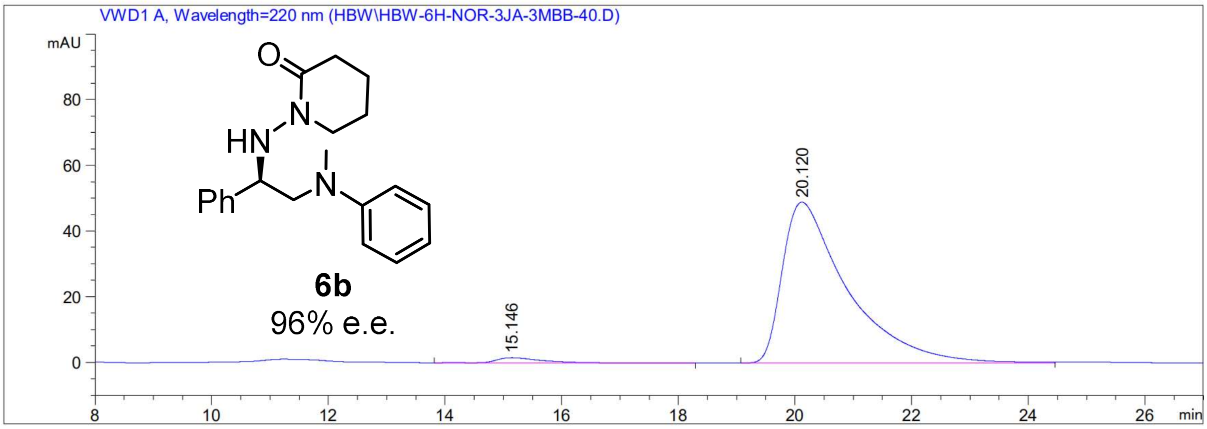

#	[min]		[min]	[mAU*s]	[mAU]	%
1	15.147	BB	0.7835	84.45888	1.57021	2.1977
2	20.120	BB	1.1080	3758.67017	48.93871	97.8023

Supplementary Figure 31. HPLC trace for the racemic reference rac-**6b**, and non-racemic product **6b**.

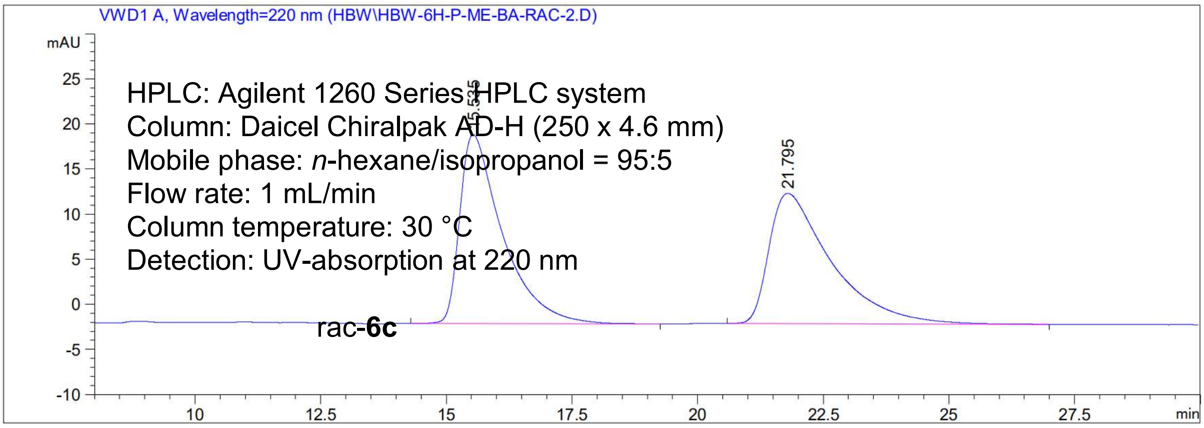

#	[min]		[min]	[mAU*s]	[mAU]	%
1	15.535	BB	0.8653	1246.72754	20.97874	50.0973
2	21.795	BB	1.2438	1241.88513	14.43357	49.9027

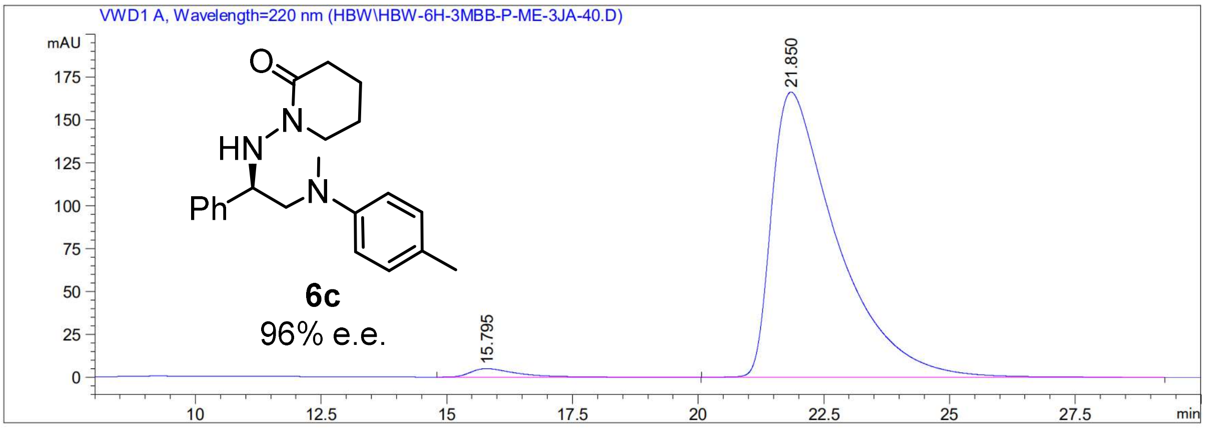

#	[min]		[min]	[mAU*s]	[mAU]	%
1	15.796	BB	0.9271	312.13373	4.92673	2.0297
2	21.850	BB	1.3334	1.50658e4	166.21985	97.9703

Supplementary Figure 32. HPLC trace for the racemic reference rac-**6c**, and non-racemic product **6c**.

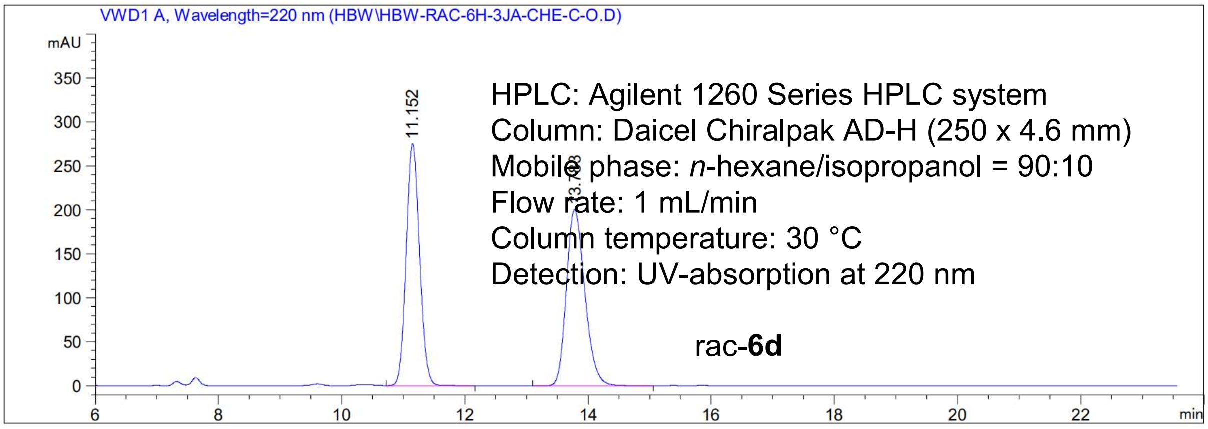

#	[min]		[min]	[mAU*s]	[mAU]	%
1	11.152	VB	0.2366	4163.30615	274.96307	49.9284
2	13.783	BV	0.3232	4175.24463	200.33275	50.0716

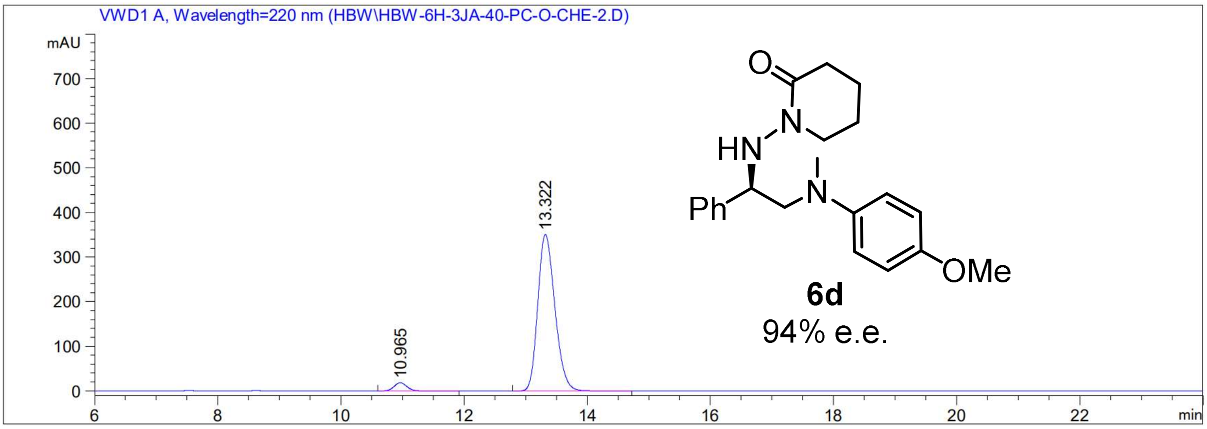

#	[min]		[min]	[mAU*s]	[mAU]	%
1	10.964	MF	0.1952	215.20651	18.37333	3.0823
2	13.321	BB	0.2992	6766.70801	350.50461	96.9177

Supplementary Figure 33. HPLC trace for the racemic reference rac-**6d**, and non-racemic product **6d**.

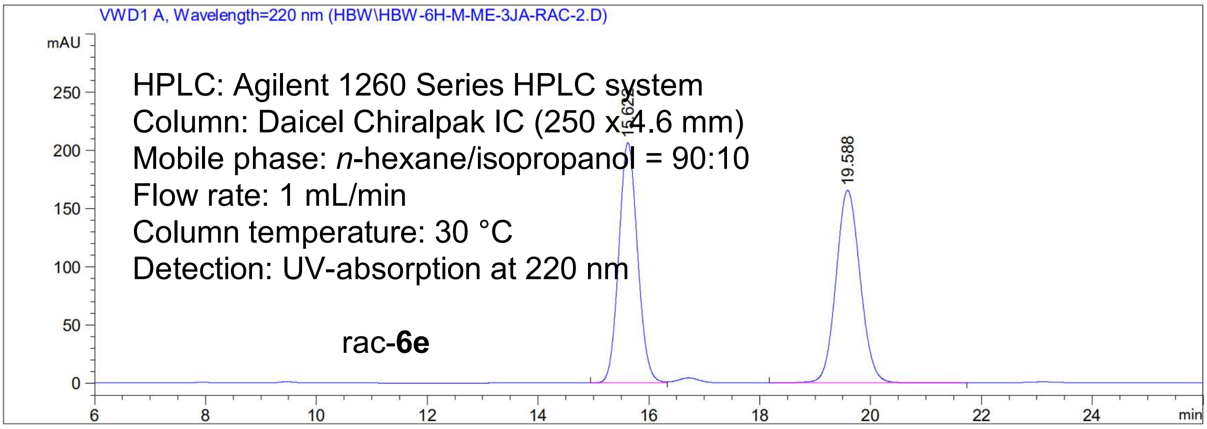

#	[min]		[min]	[mAU*s]	[mAU]	%
1	15.622	BV	0.3669	4856.14355	206.07265	49.1555
2	19.588	BV	0.4707	5023.00977	165.19196	50.8445

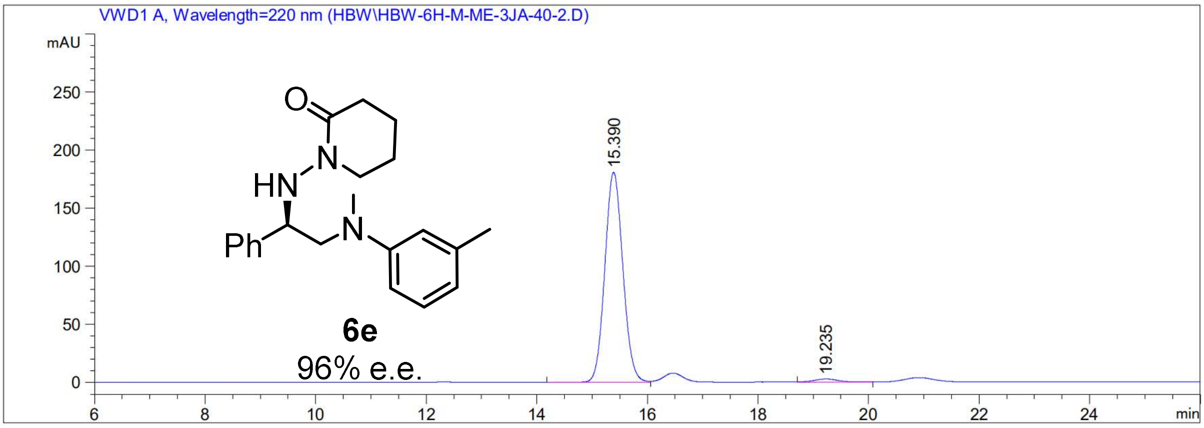

#	[min]		[min]	[mAU*s]	[mAU]	%
1	15.390	BV	0.3521	4082.12988	180.32460	97.8932
2	19.235	BV	0.4920	87.85173	2.69685	2.1068

Supplementary Figure 34. HPLC trace for the racemic reference rac-**6e**, and non-racemic product **6e**.

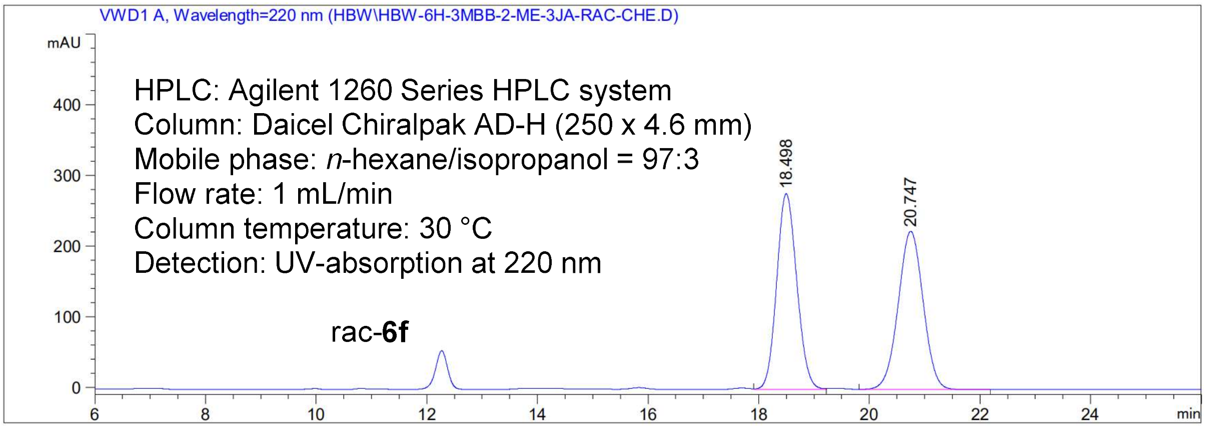

#	[min]		[min]	[mAU*s]	[mAU]	%
1	18.498	VV	0.3954	7010.75195	276.90213	50.0003
2	20.747	BB	0.4894	7010.67041	223.81477	49.9997

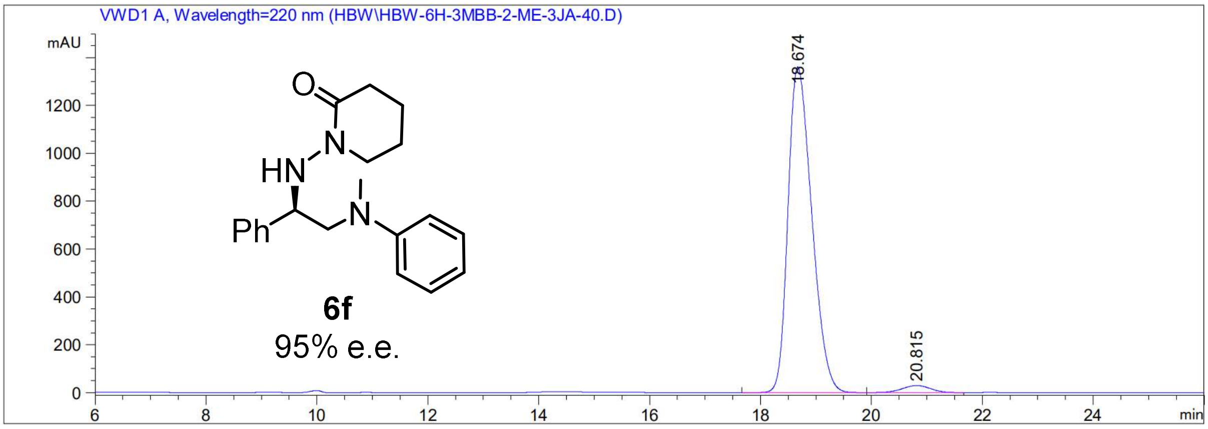

#	[min]		[min]	[mAU*s]	[mAU]	%
1	18.673	BV	0.4637	4.05424e4	1360.51758	97.3913
2	20.815	VV	0.5657	1085.94263	29.76110	2.6087

Supplementary Figure 35. HPLC trace for the racemic reference rac-**6f**, and non-racemic product **6f**.

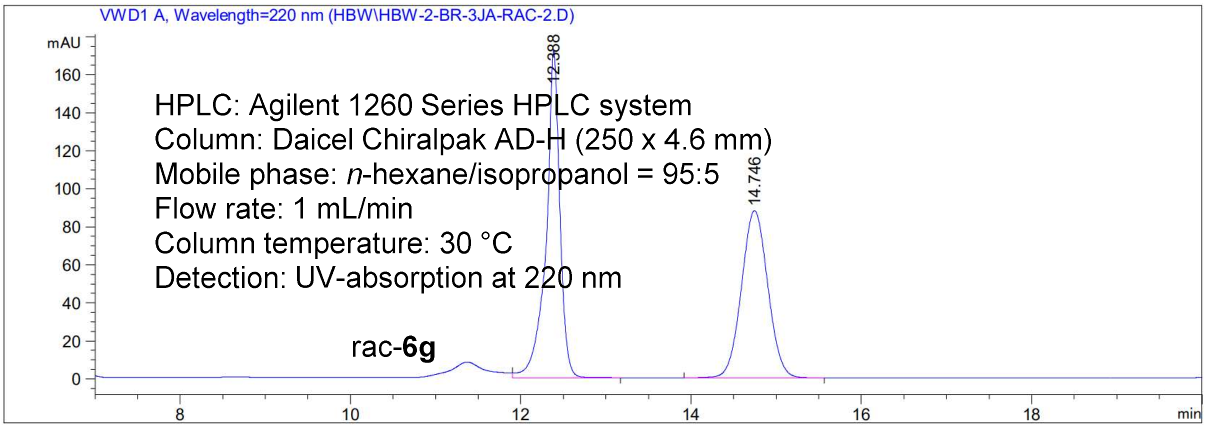

#	[min]		[min]	[mAU*s]	[mAU]	%
1	12.389	VB	0.1688	1952.54565	171.50009	50.7390
2	14.746	BB	0.3394	1895.67114	87.96947	49.2610

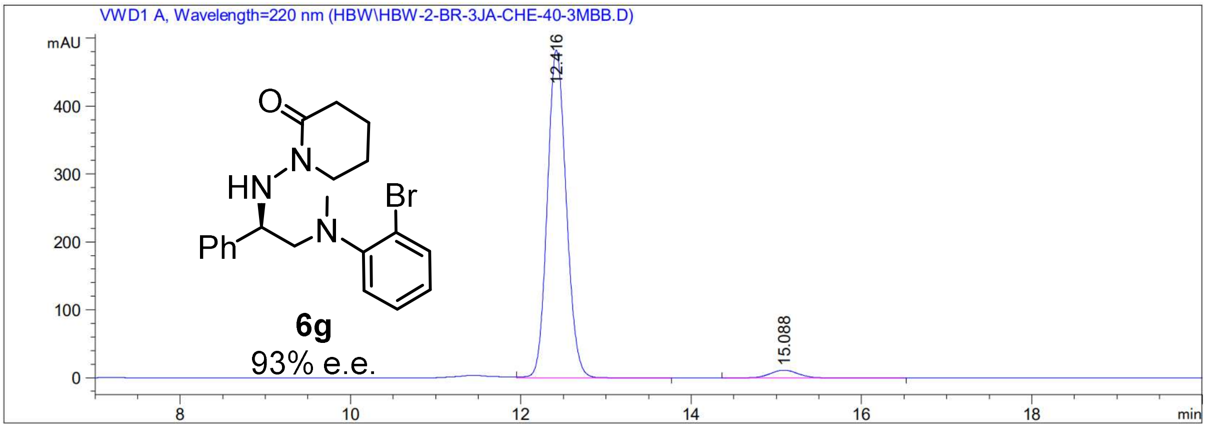

#	[min]		[min]	[mAU*s]	[mAU]	%
1	12.416	VB	0.2404	7451.40186	481.95438	96.6159
2	15.088	BB	0.3581	260.99536	11.44104	3.3841

Supplementary Figure 36. HPLC trace for the racemic reference rac-**6g**, and non-racemic product **6g**.

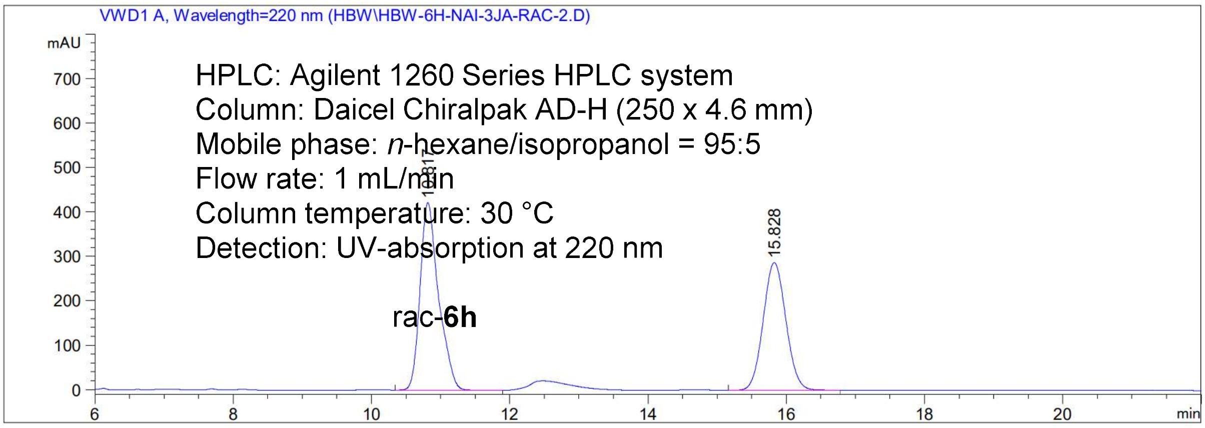

#	[min]		[min]	[mAU*s]	[mAU]	%
1	10.817	VV	0.2776	7861.86279	421.57956	54.4271
2	15.828	BV	0.3550	6582.89844	287.60114	45.5729

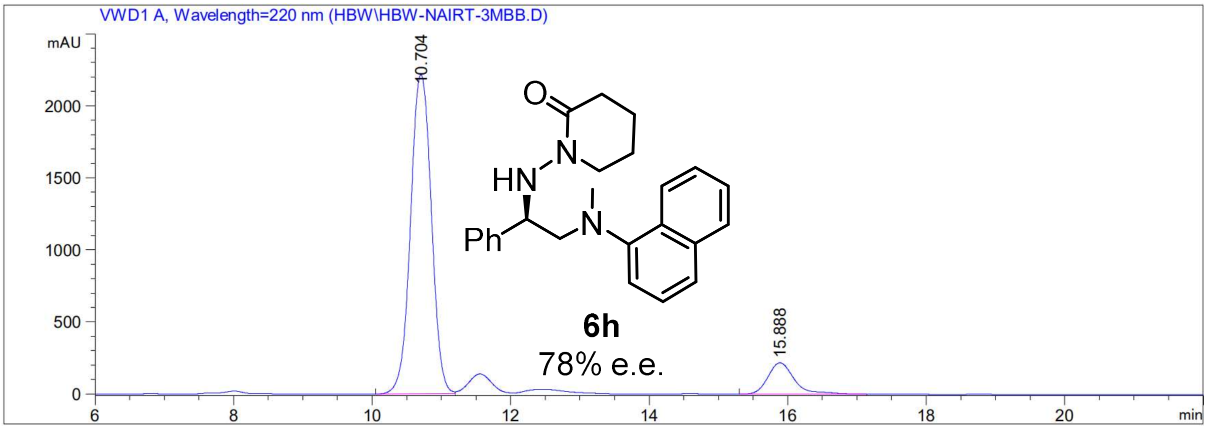

#	[min]		[min]	[mAU*s]	[mAU]	%
1	10.704	BV	0.3310	4.63556e4	2216.90405	88.9962
2	15.888	BB	0.4041	5731.55029	217.01244	11.0038

Supplementary Figure 37. HPLC trace for the racemic reference rac-**6h**, and non-racemic product **6h**.

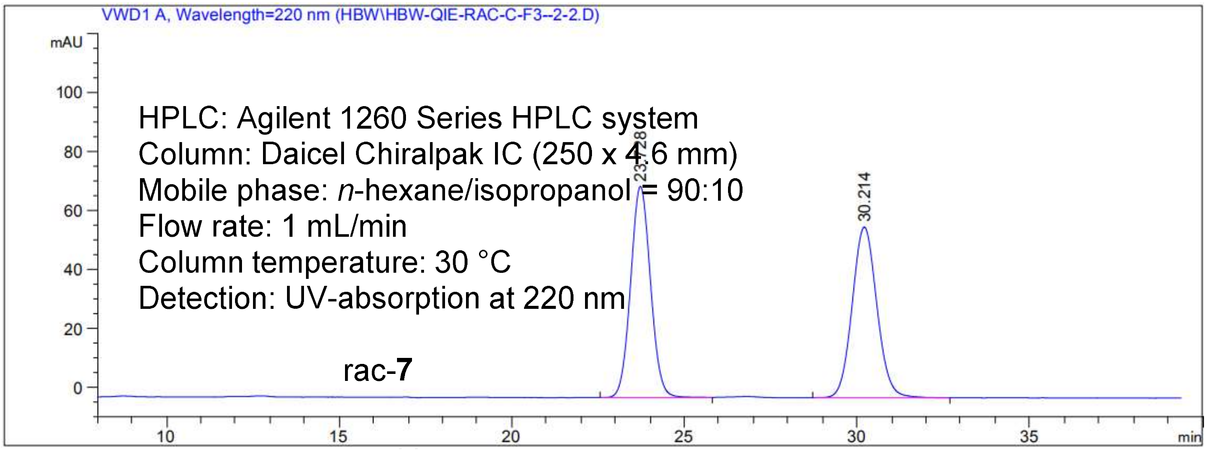

#	[min]		[min]	[mAU*s]	[mAU]	%
1	23.728	BB	0.6265	2858.03149	71.47385	49.8565
2	30.214	BB	0.7685	2874.48242	57.77239	50.1435

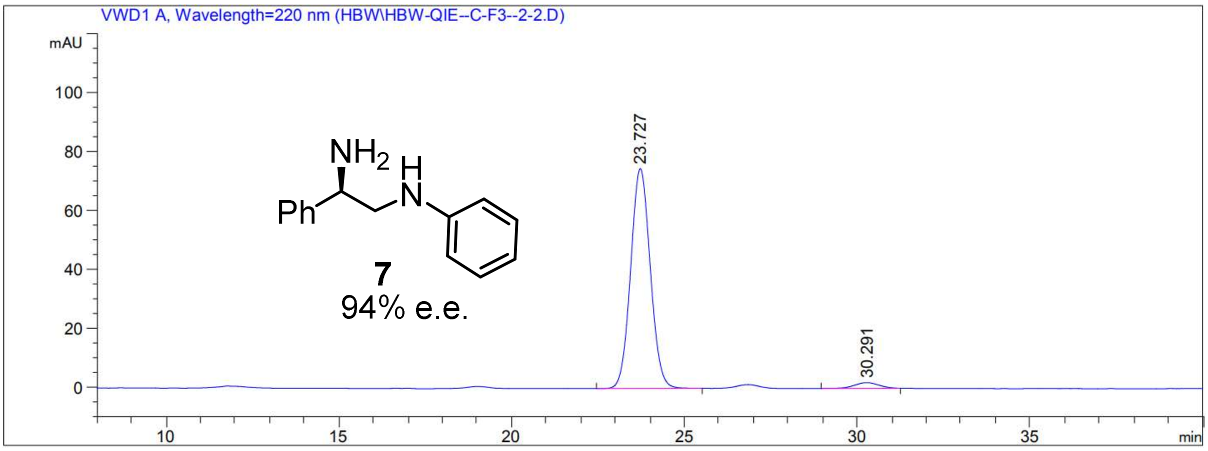

#	[min]		[min]	[mAU*s]	[mAU]	%
1	23.727	BB	0.6165	2937.17651	74.41936	96.7714
2	30.291	MM R	0.8379	97.99293	1.94921	3.2286

Supplementary Figure 38. HPLC trace for the racemic reference rac-**7**, and non-racemic product-**7**.

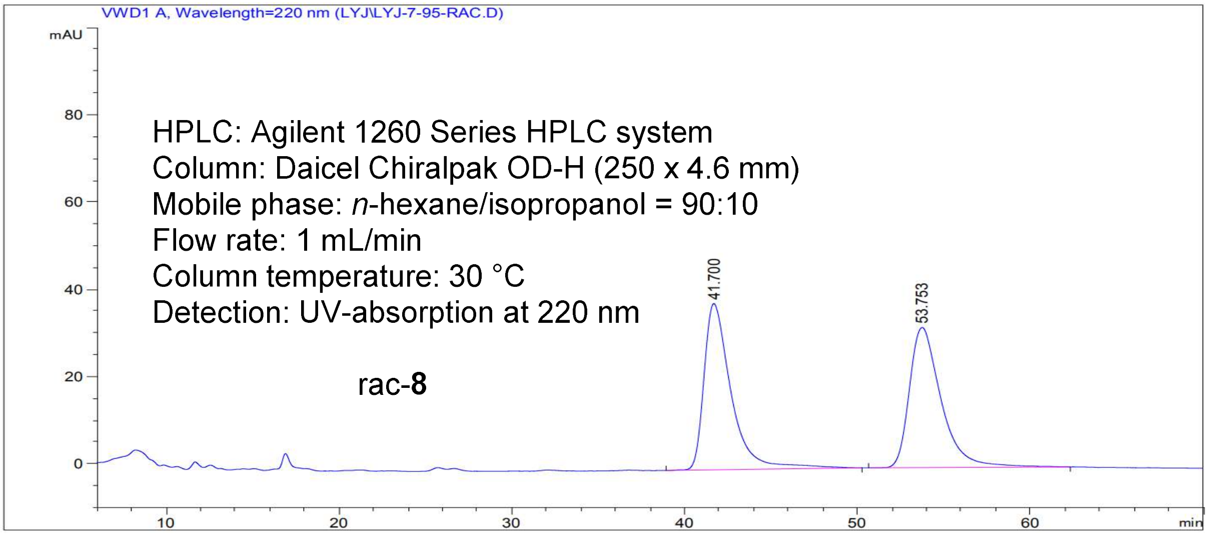

#	[min]		[min]	[mAU*s]	[mAU]	%
1	41.700	BB	1.6385	4176.75049	38.11336	50.4587
2	53.753	BB	1.9135	4100.80664	32.11994	49.5413

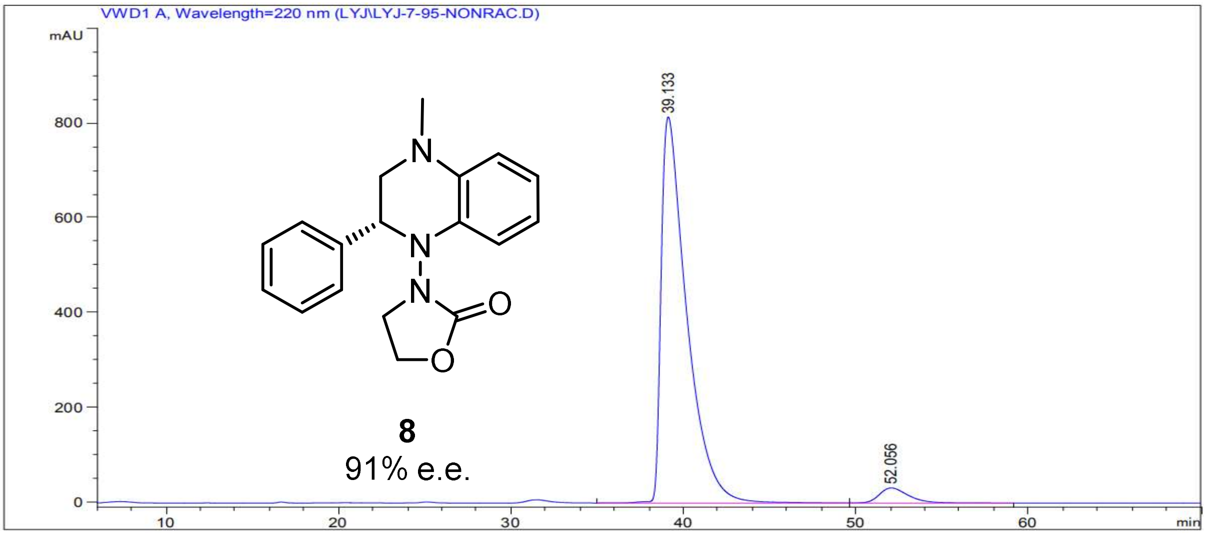

#	[min]		[min]	[mAU*s]	[mAU]	%
1	39.133	VV	1.5089	8.40343e4	813.68756	95.7088
2	52.056	VB	1.7789	3767.80005	31.87786	4.2912

Supplementary Figure 39. HPLC trace for the racemic reference rac-**8**, and non-racemic product-**8**.

7. ^{1}H and ^{13}C NMR Spectrum

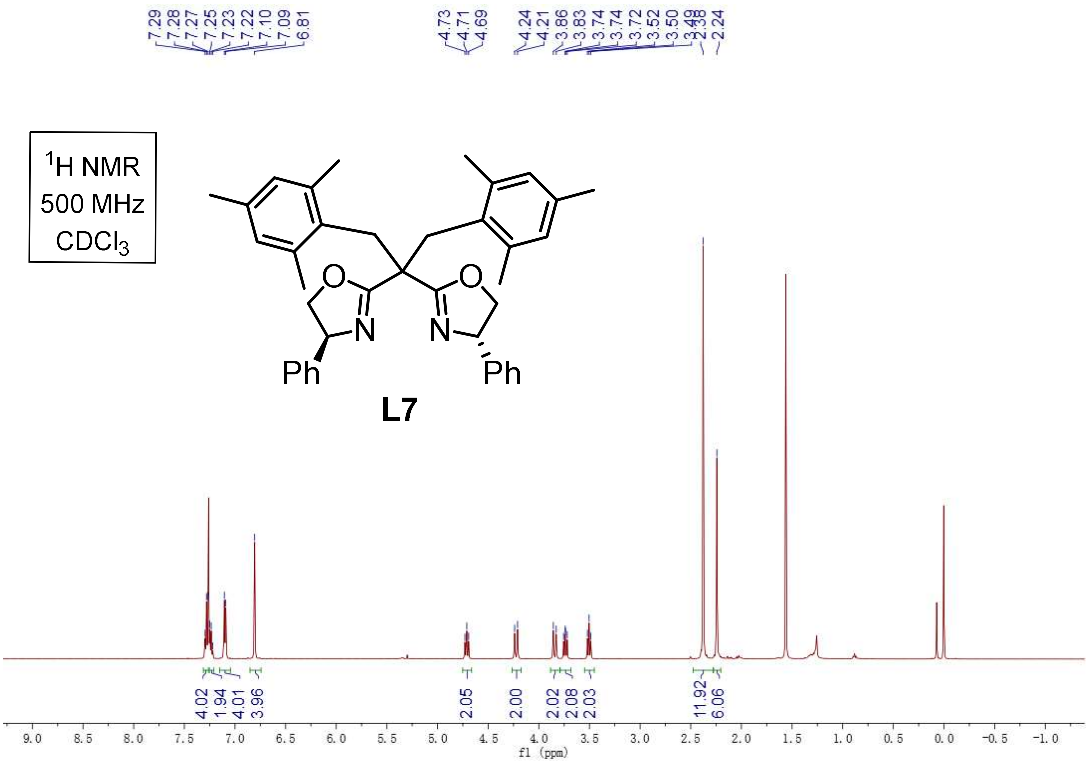

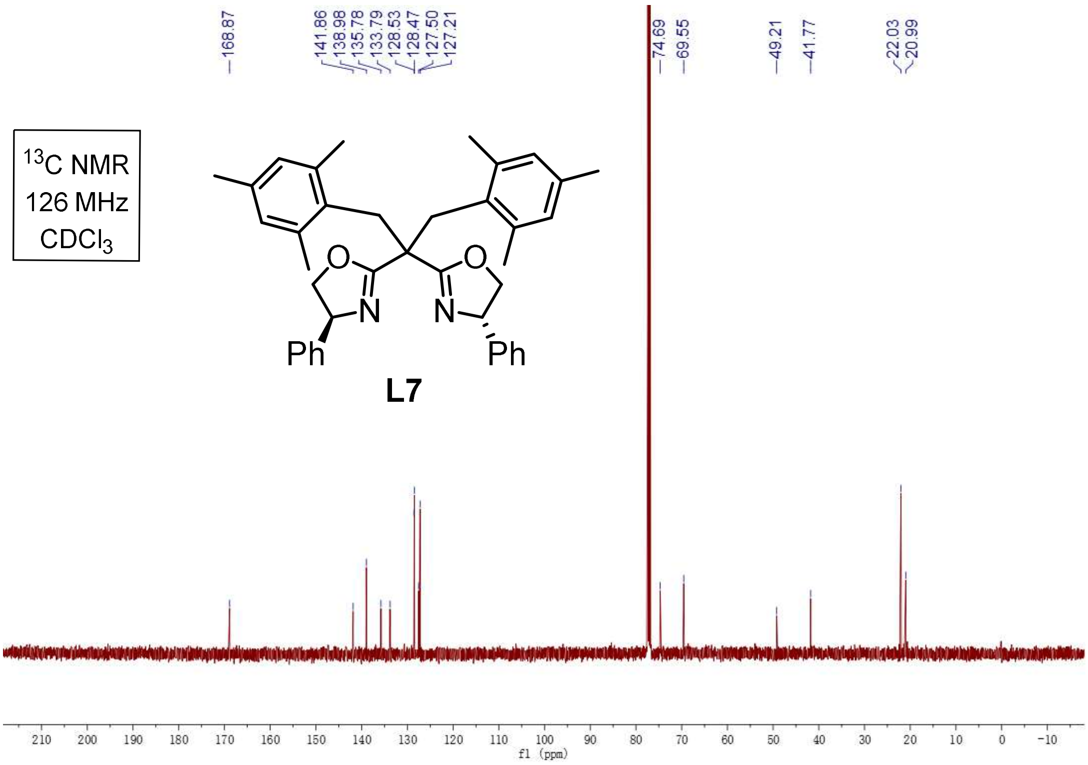

Supplementary Figure 40. ^{1}H and ^{13}C-NMR of **L7**.

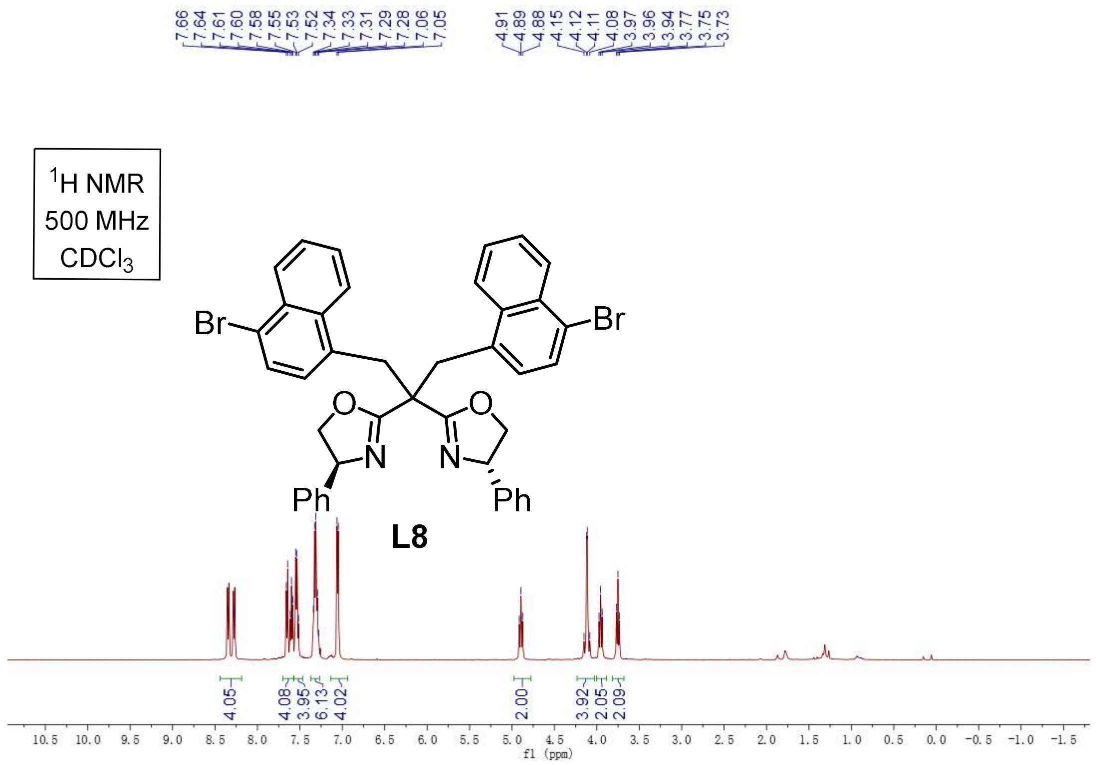

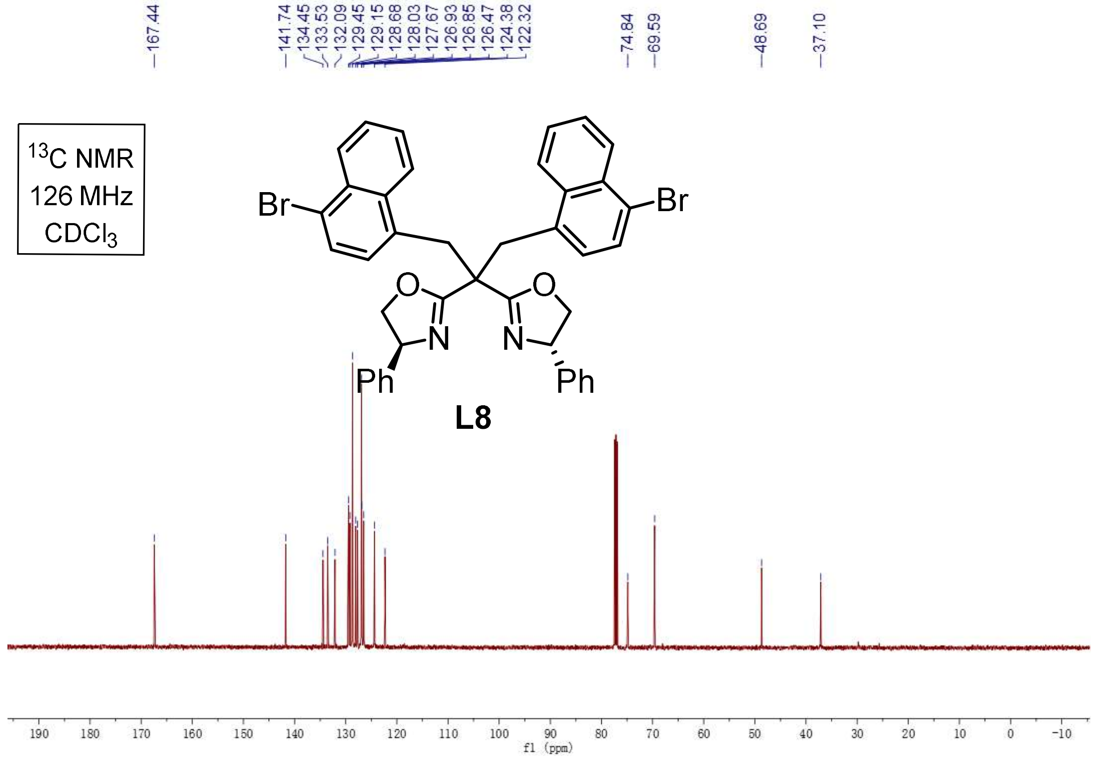

Supplementary Figure 41. ^{1}H and ^{13}C-NMR of **L8**.

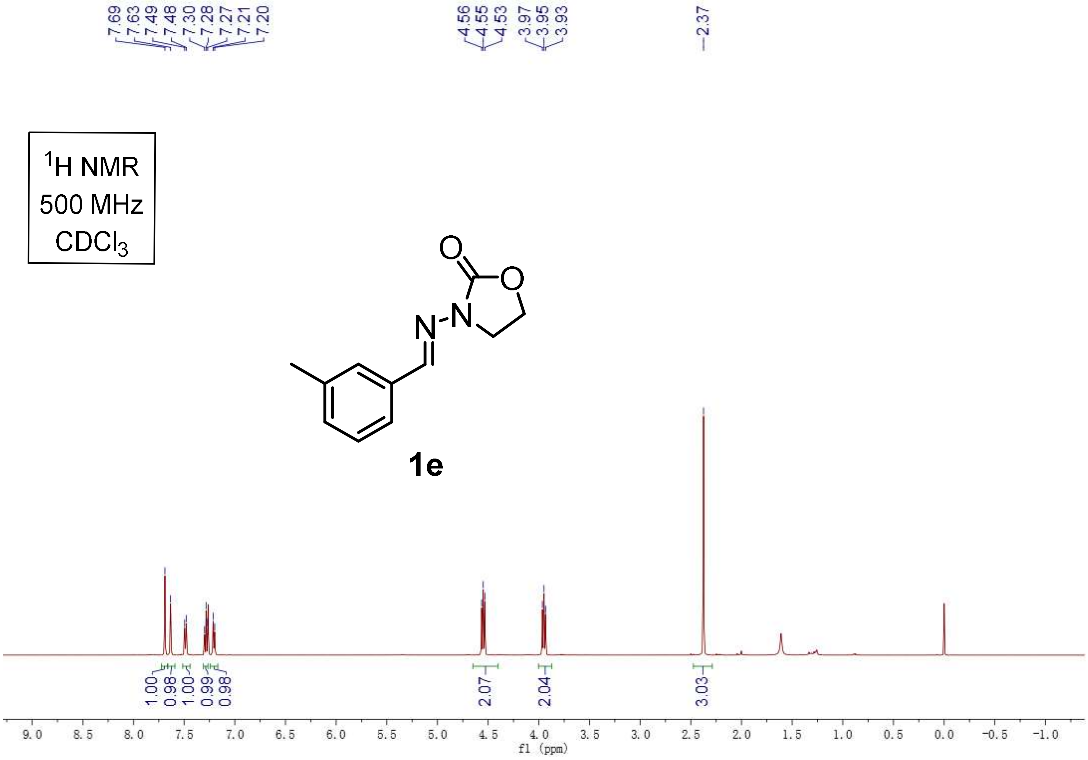

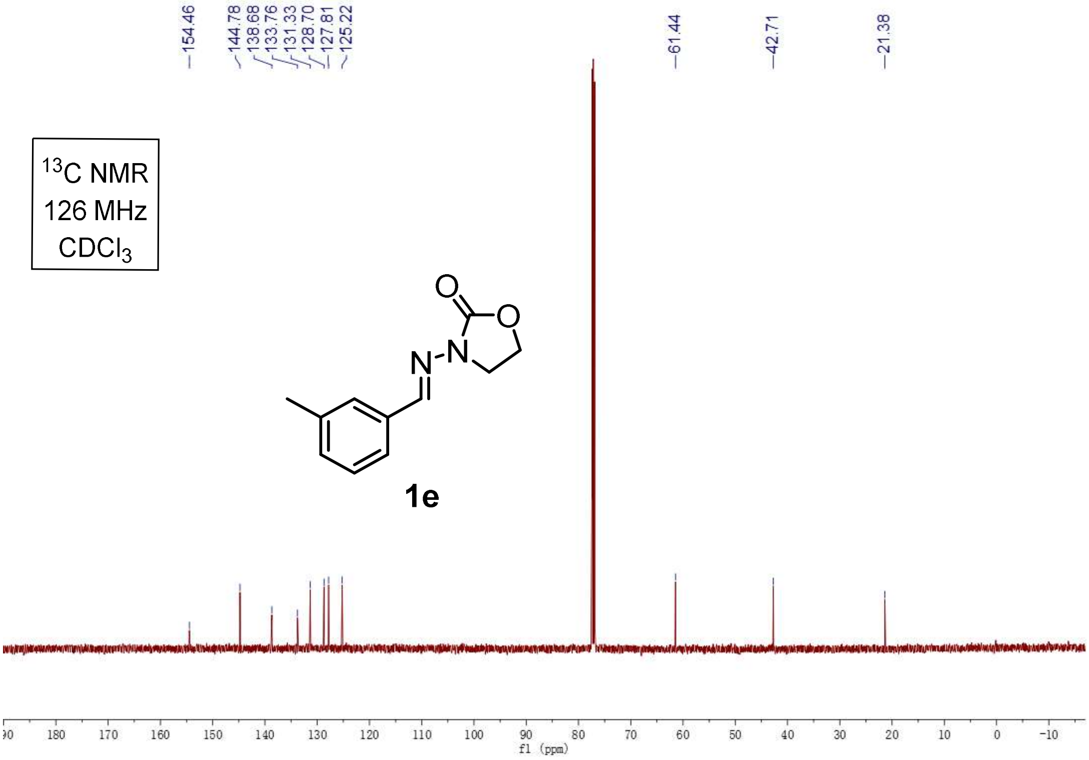

Supplementary Figure 42. ^{1}H and ^{13}C-NMR of **1e**.

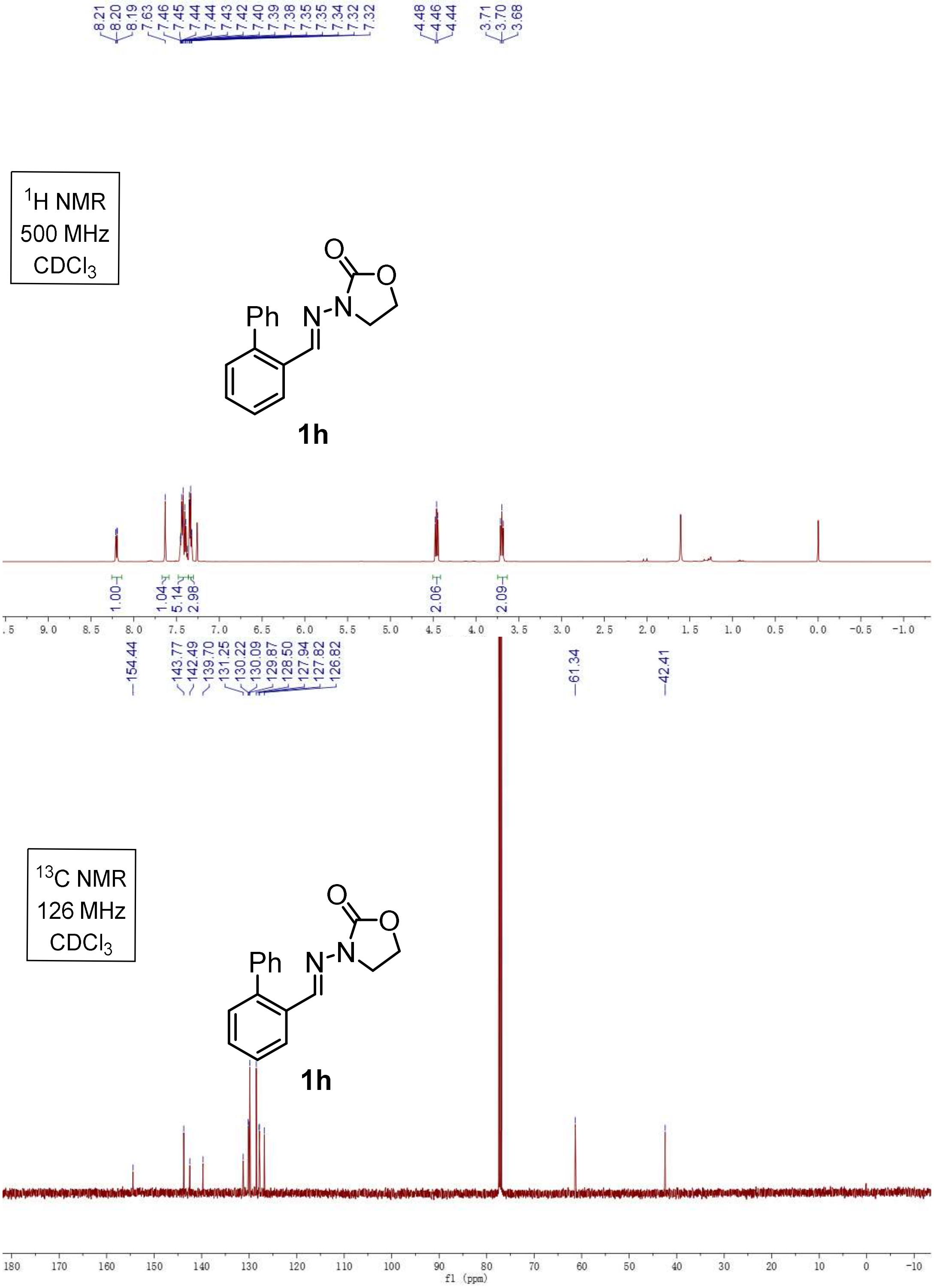

Supplementary Figure 43. ^{1}H and ^{13}C-NMR of **1h**.

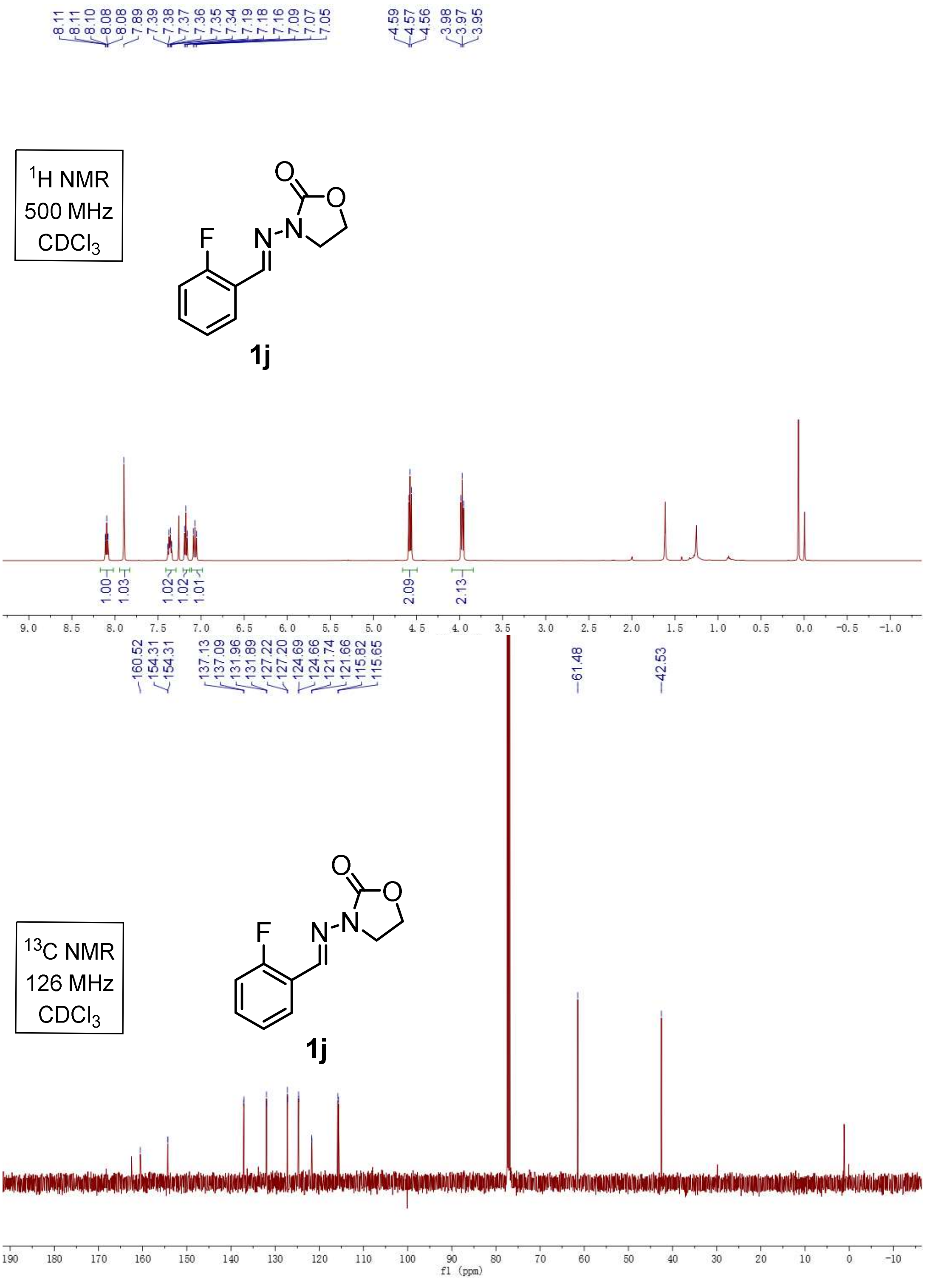

Supplementary Figure 44. ^{1}H and ^{13}C-NMR of **1j**.

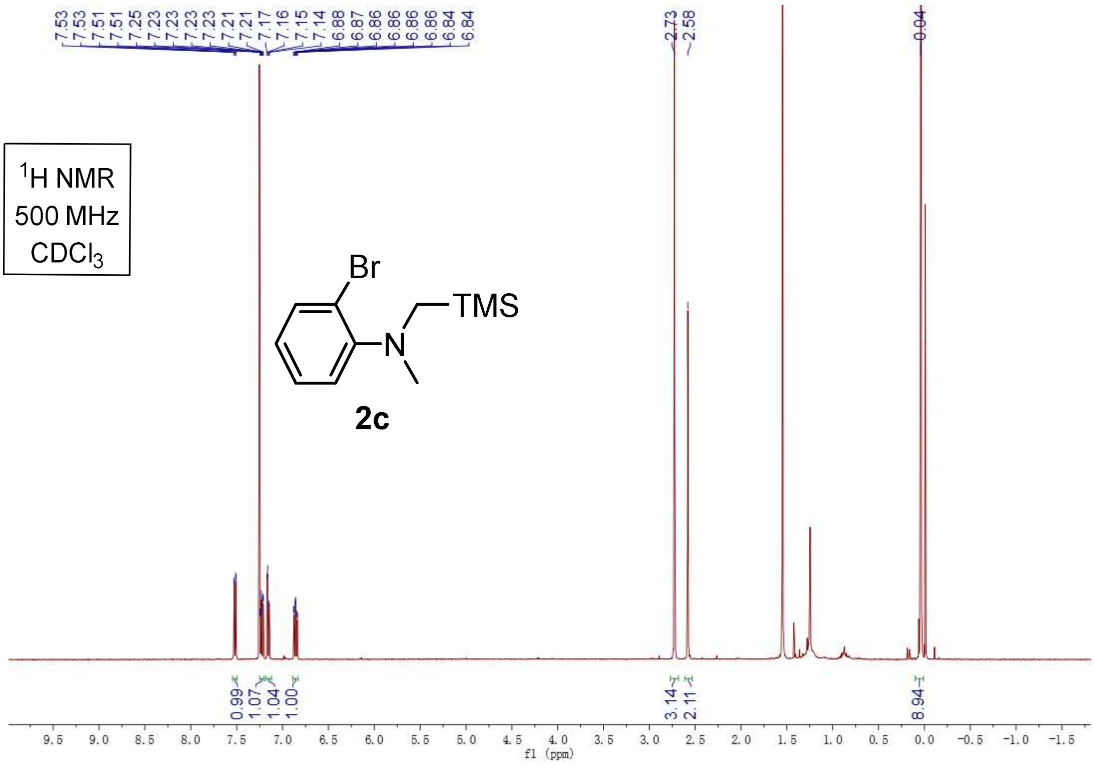

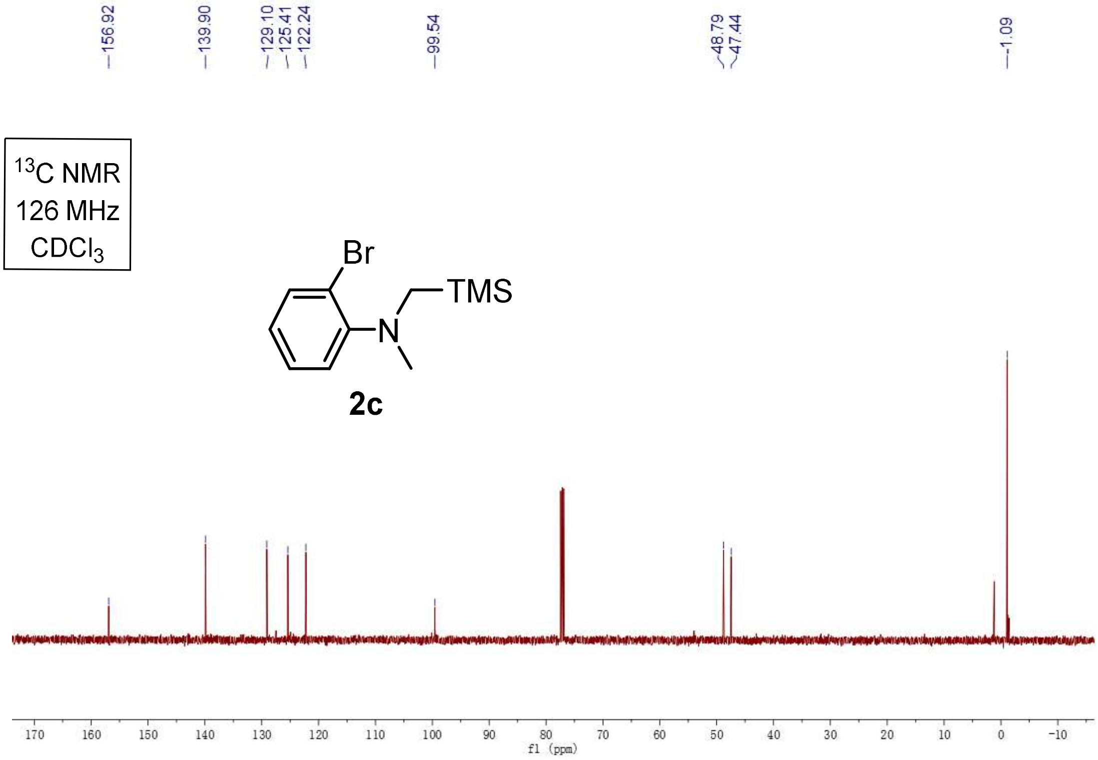

Supplementary Figure 45. ^{1}H and ^{13}C-NMR of **2c**.

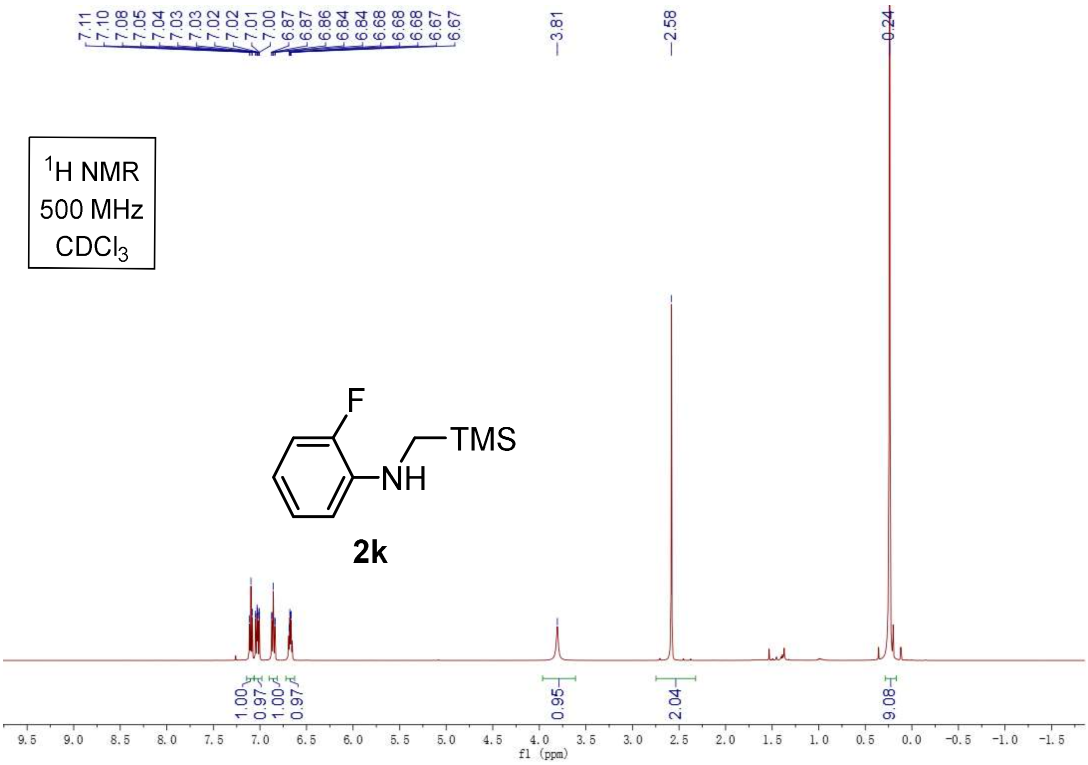

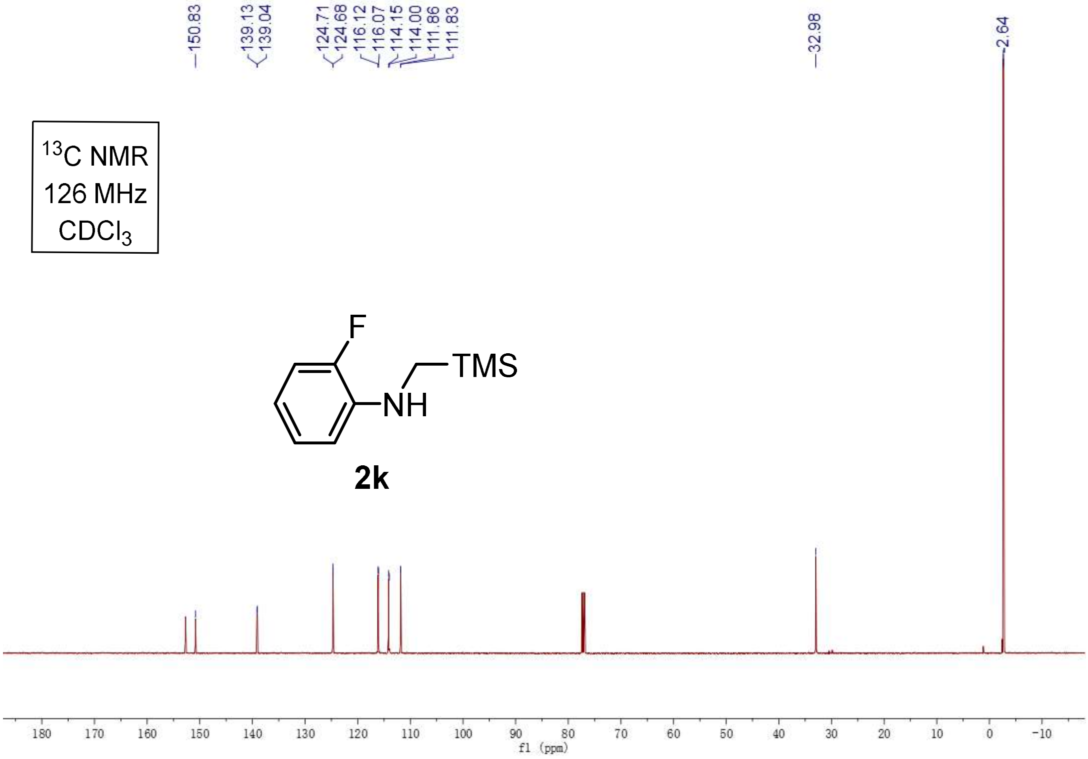

Supplementary Figure 46. ^{1}H and ^{13}C-NMR of **2k**.

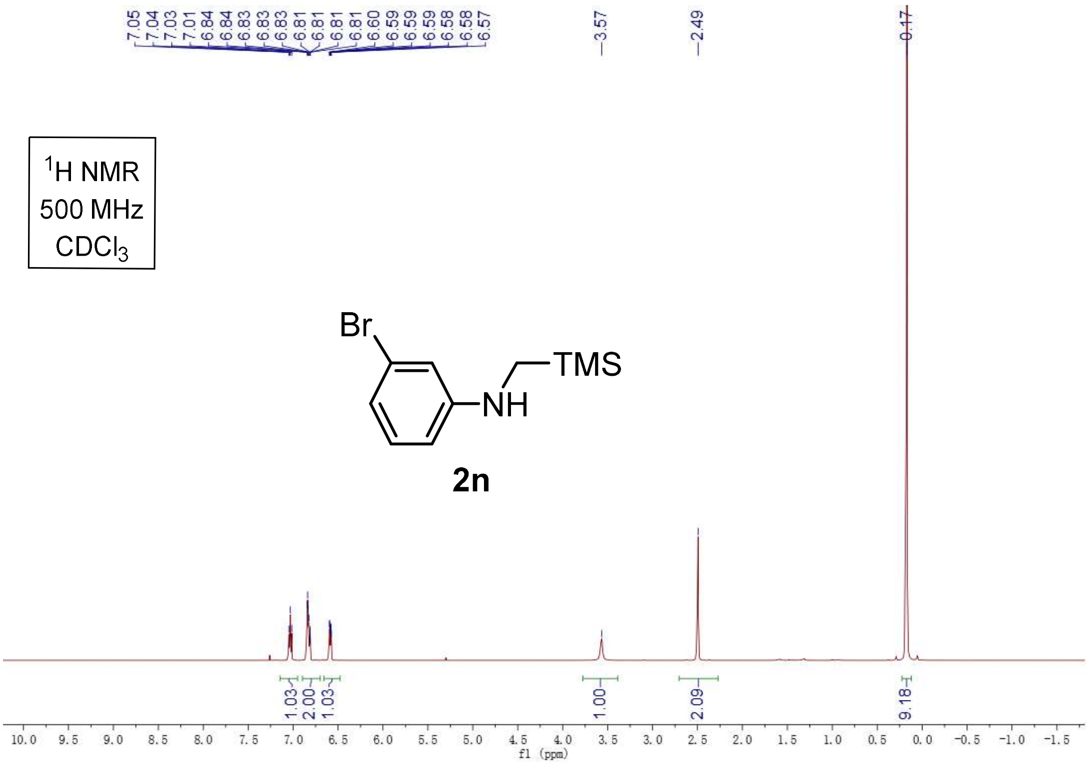

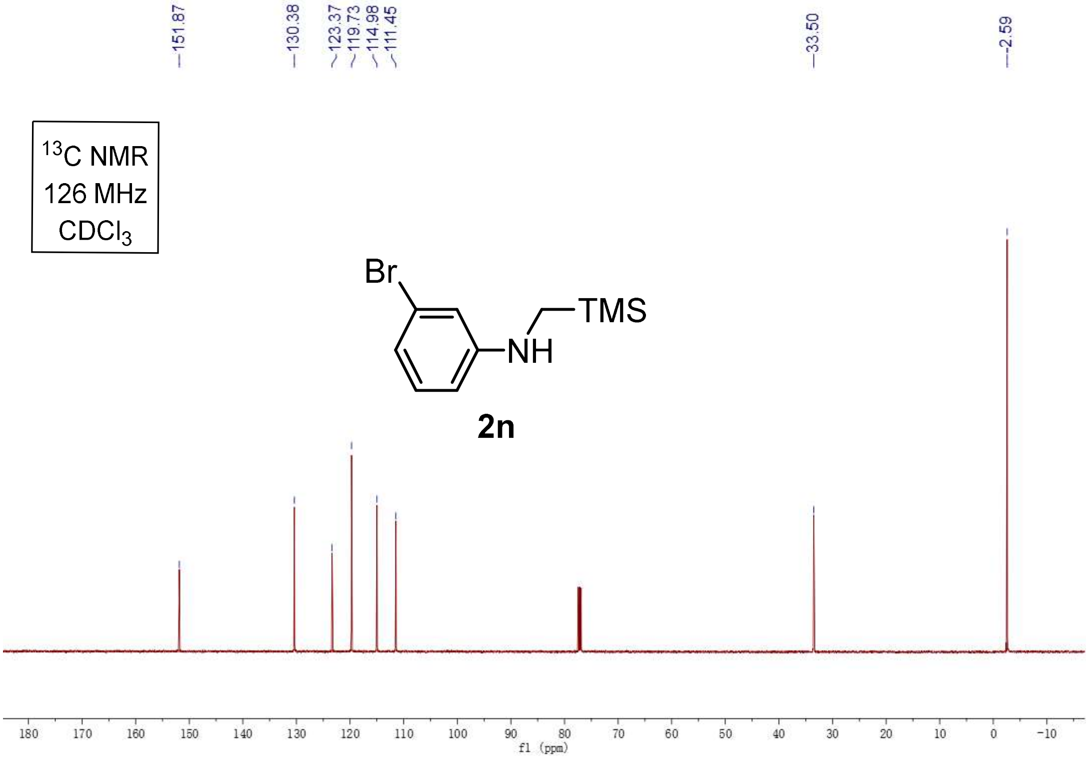

Supplementary Figure 47. ^{1}H and ^{13}C-NMR of **2n**.

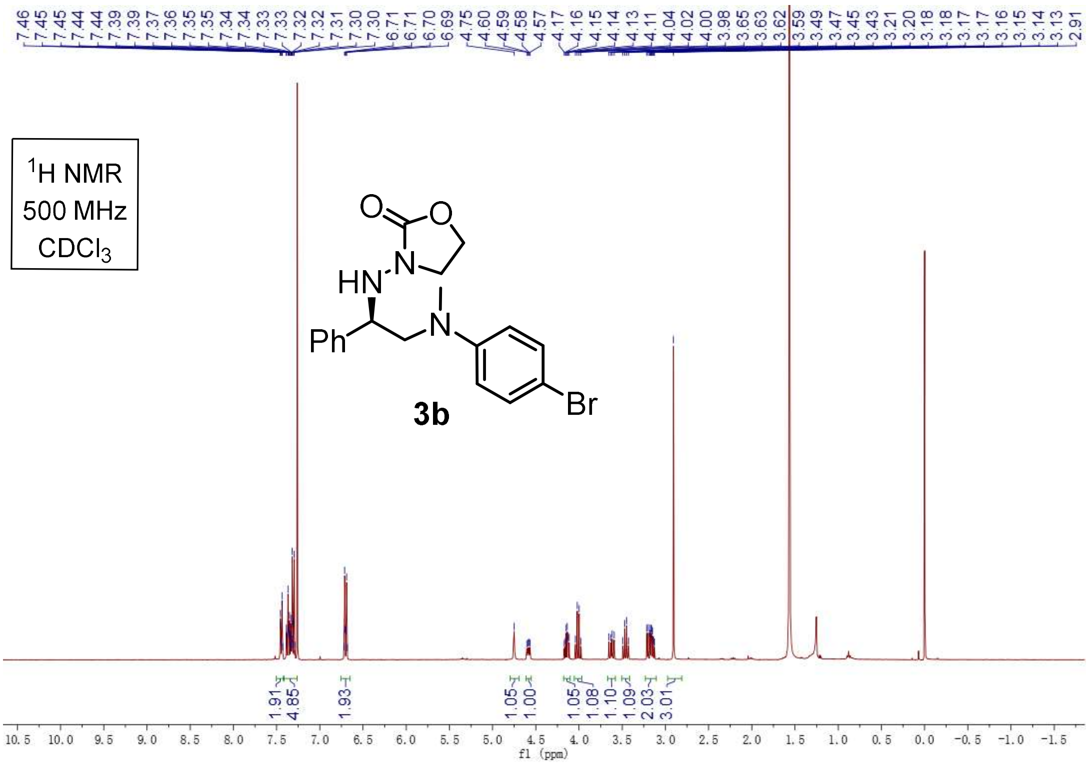

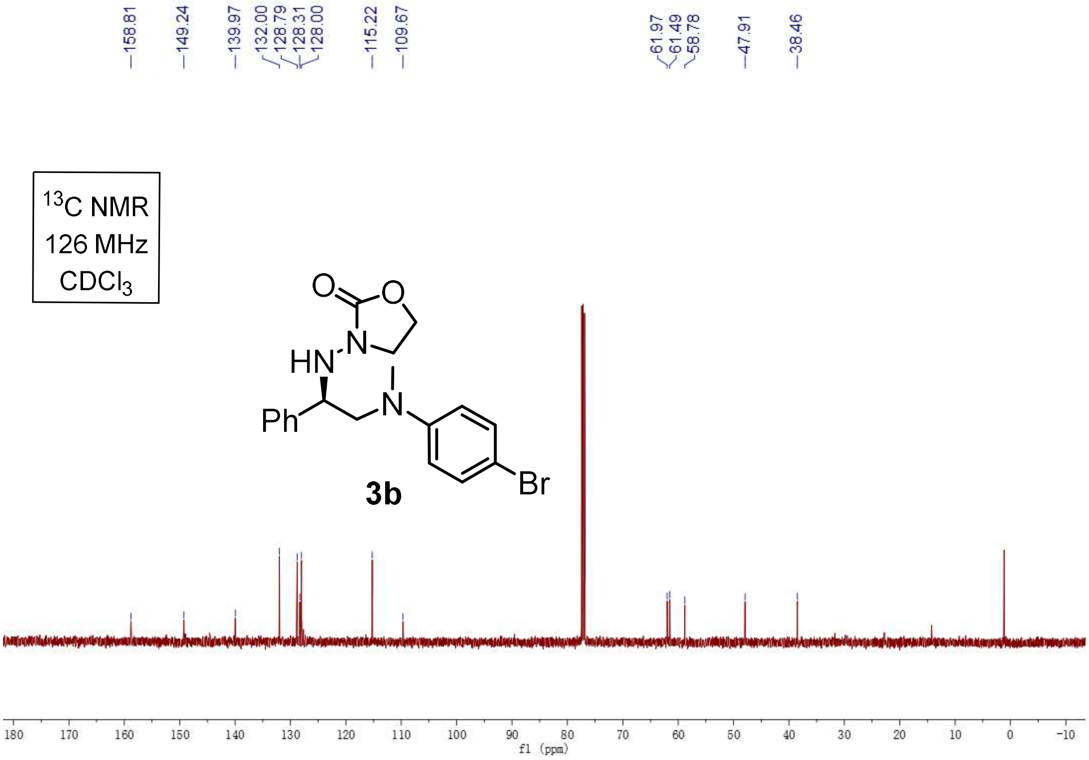

Supplementary Figure 48. ^{1}H and ^{13}C-NMR of **3b**.

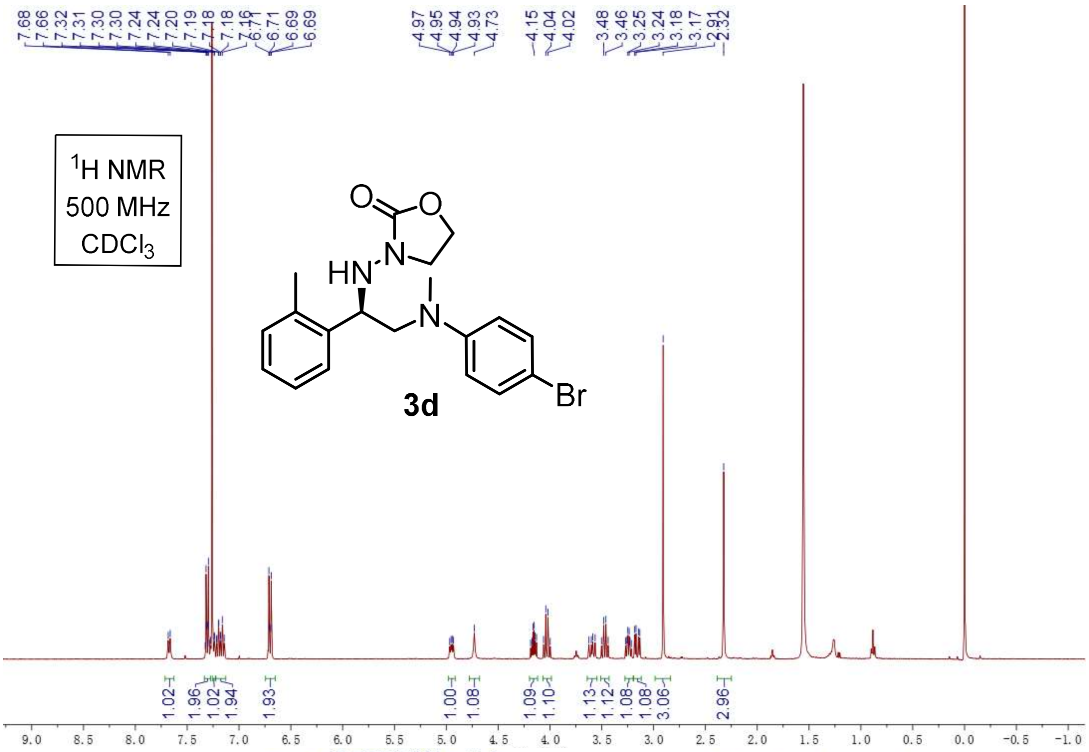

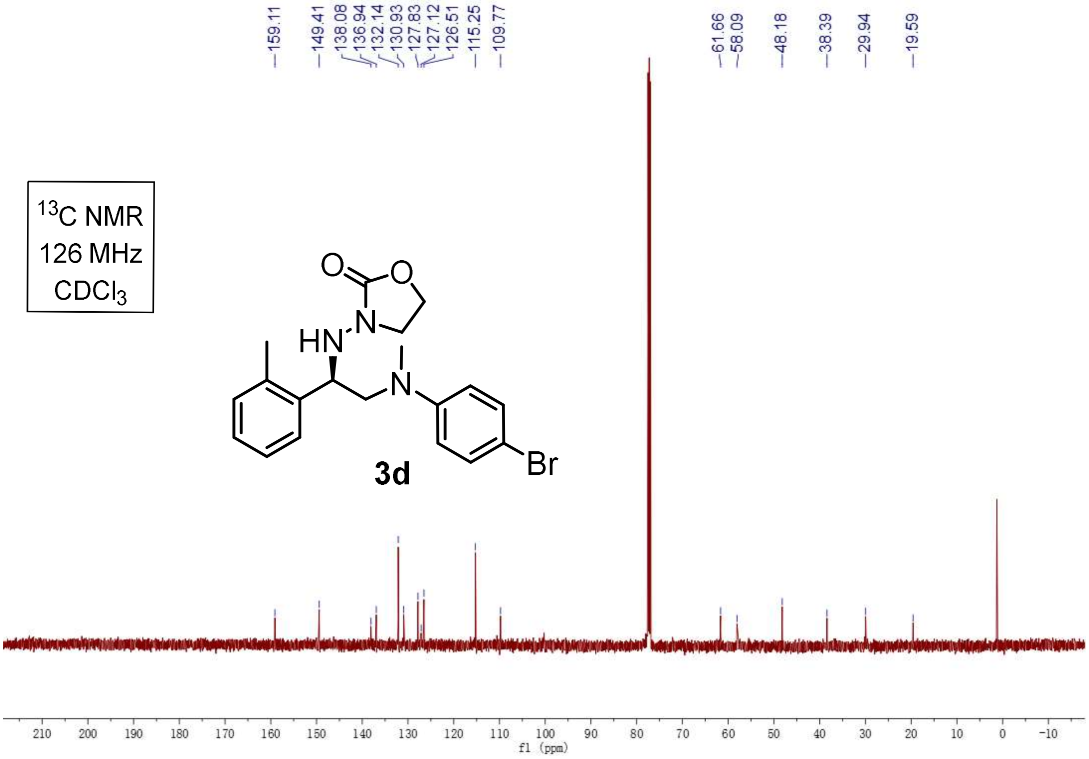

Supplementary Figure 49. ^{1}H and ^{13}C-NMR of **3d**.

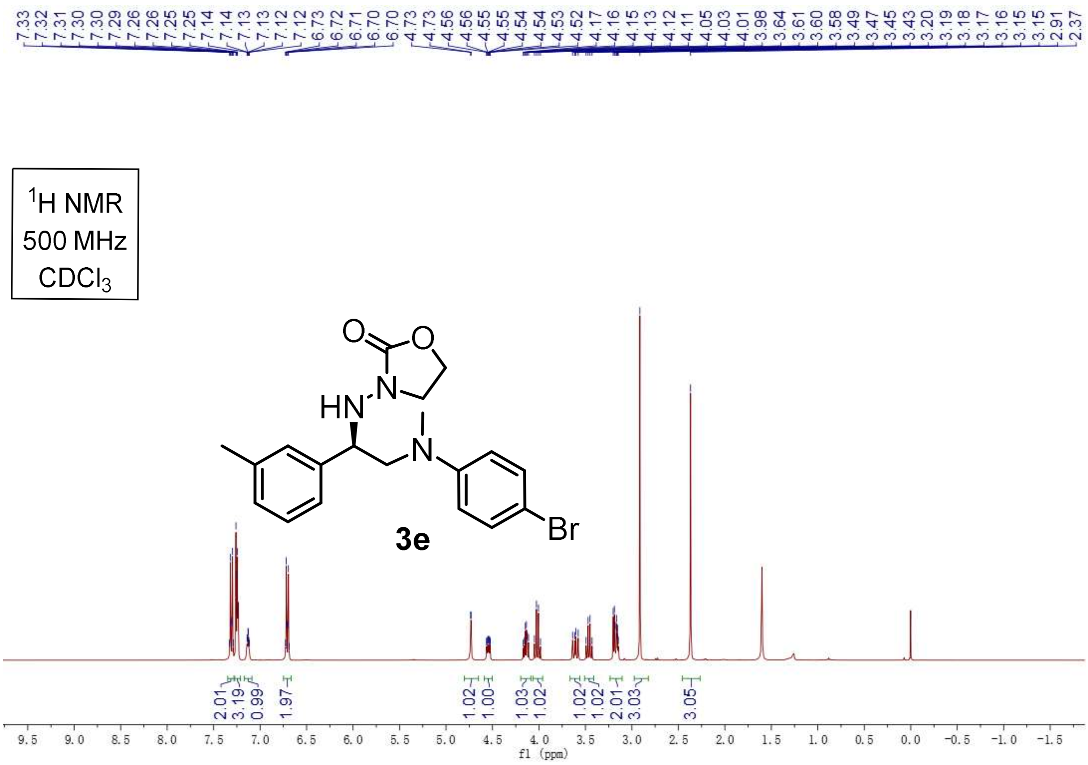

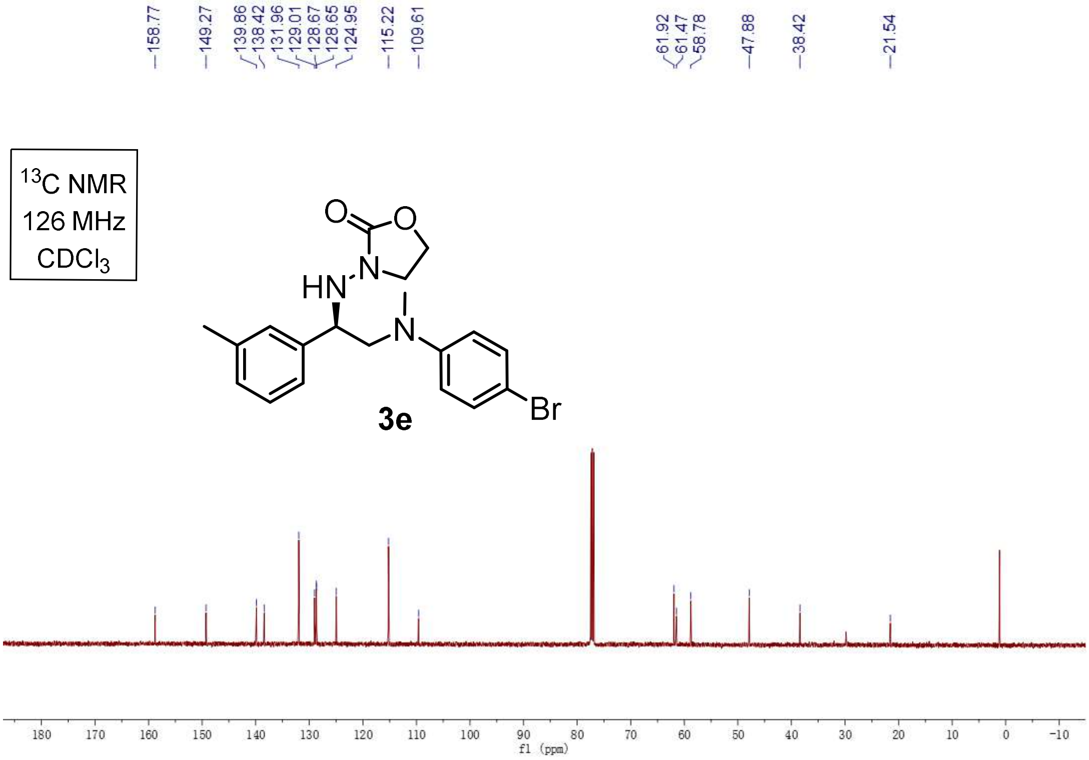

Supplementary Figure 50. ^{1}H and ^{13}C-NMR of **3e**.

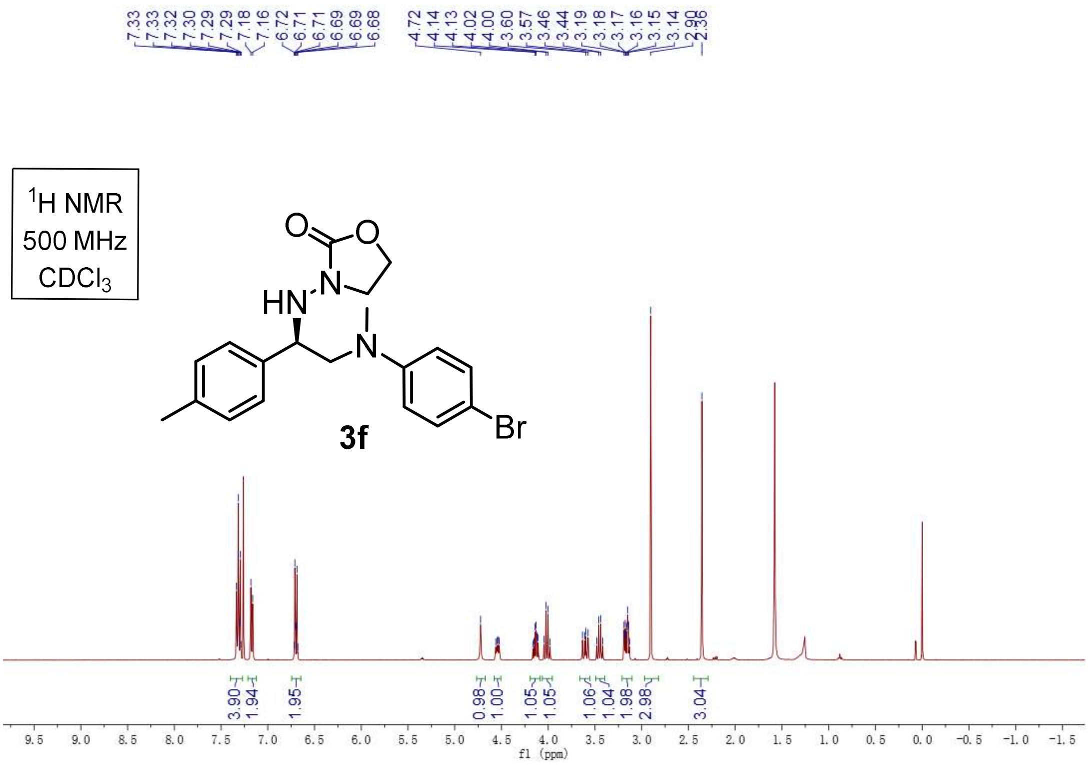

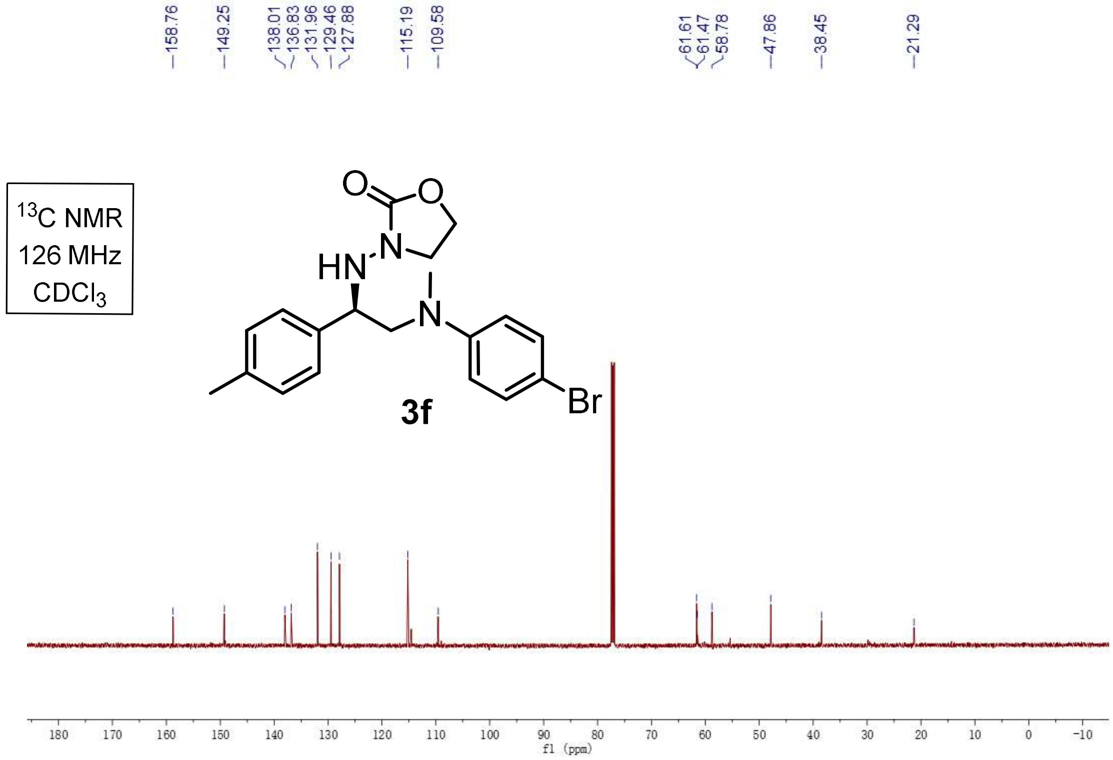

Supplementary Figure 51. ^{1}H and ^{13}C-NMR of **3f**.

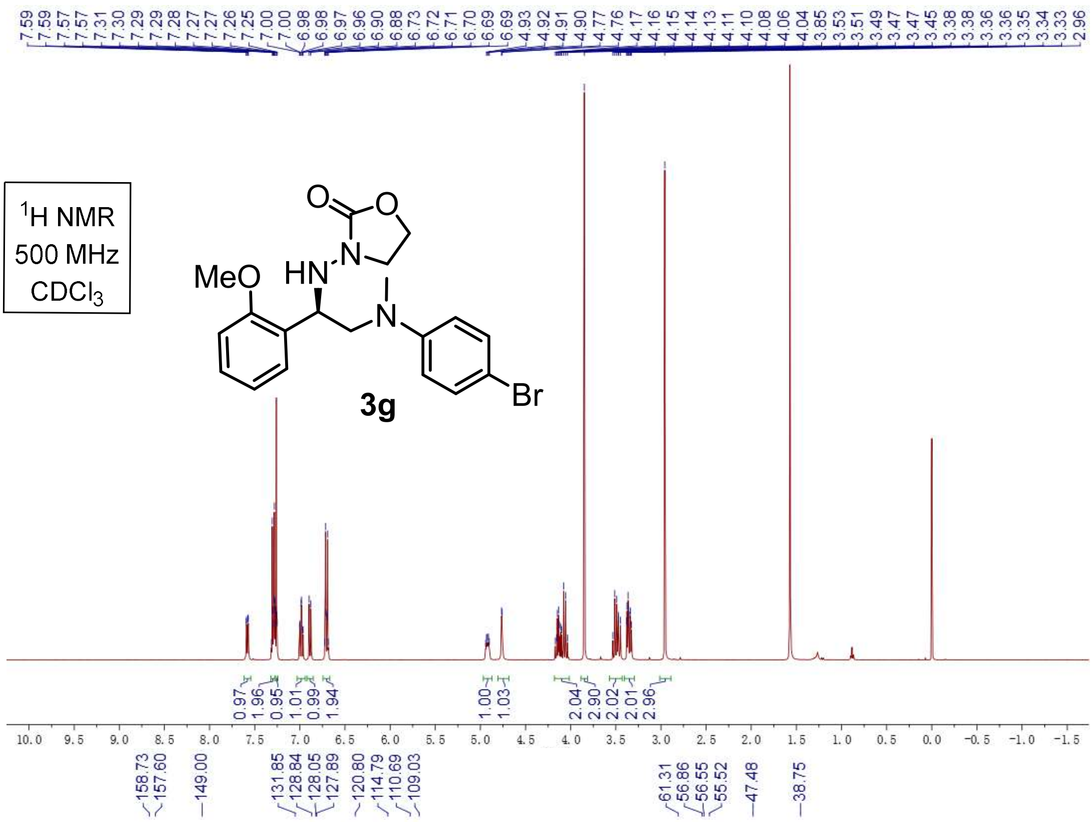

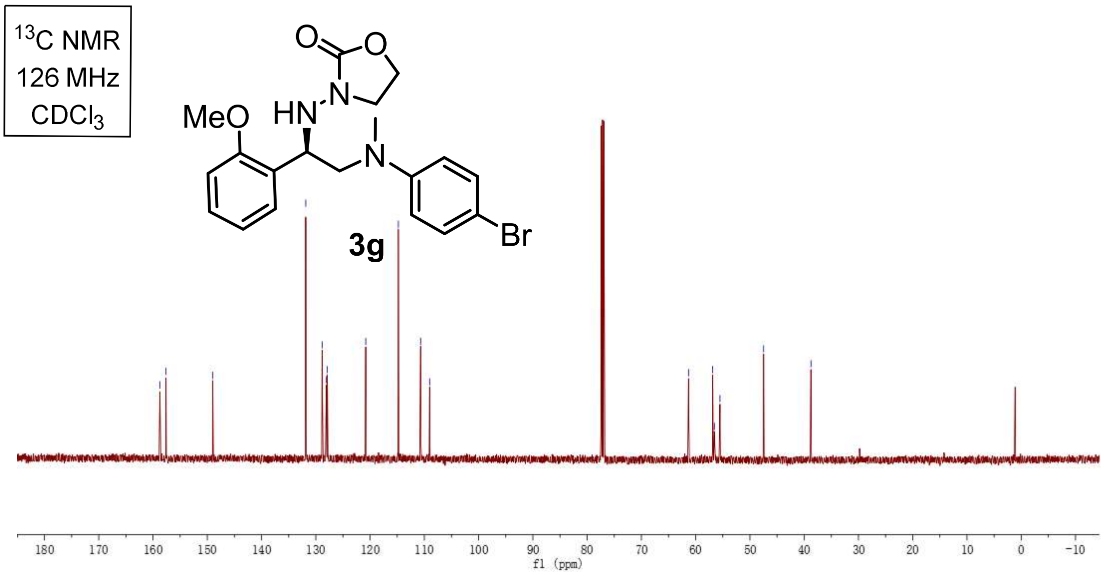

Supplementary Figure 52. ^{1}H and ^{13}C-NMR of **3g**.

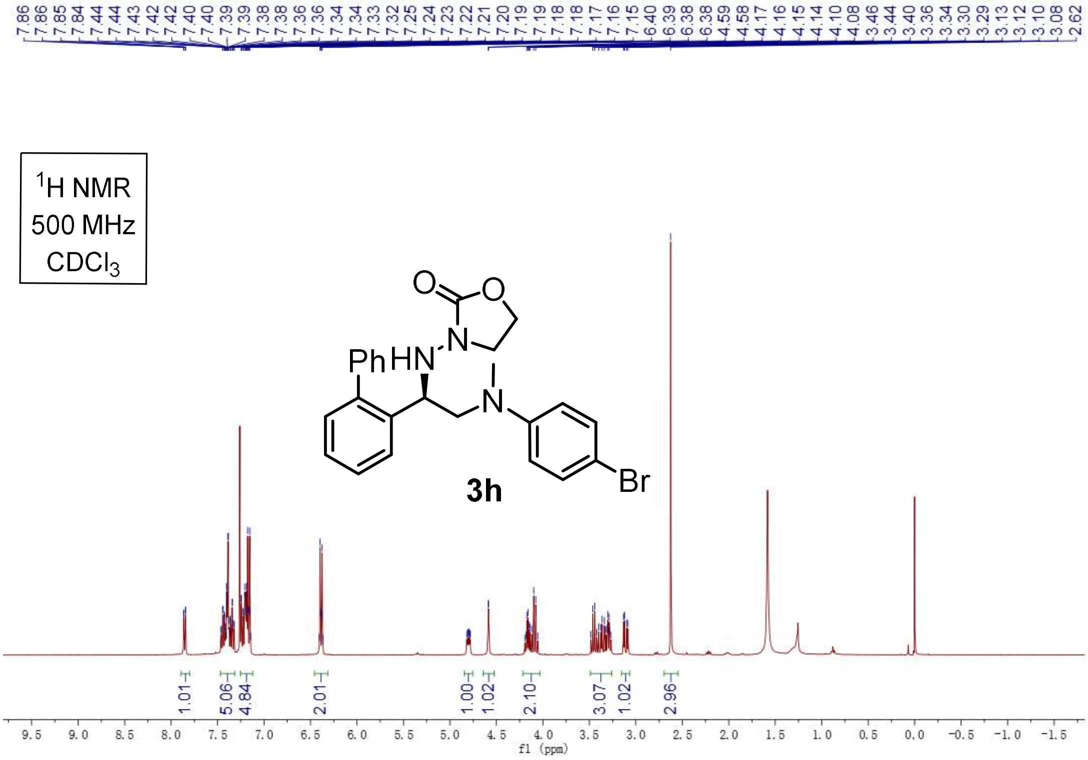

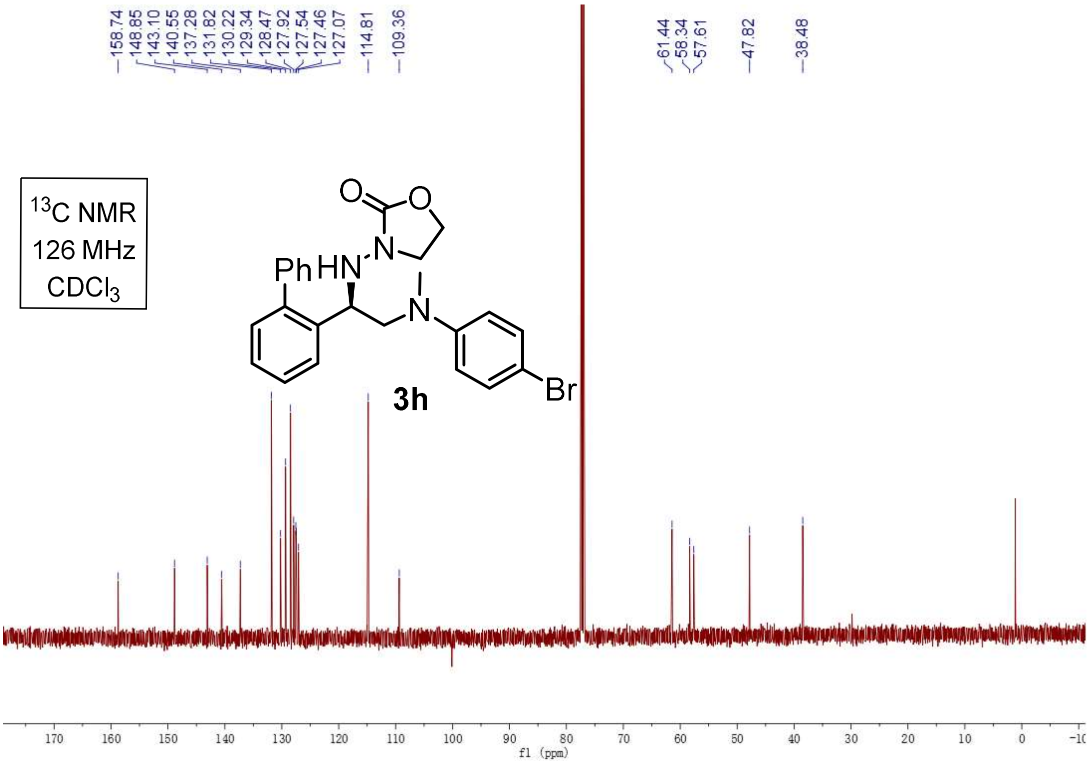

Supplementary Figure 53. ^{1}H and ^{13}C-NMR of **3h**.

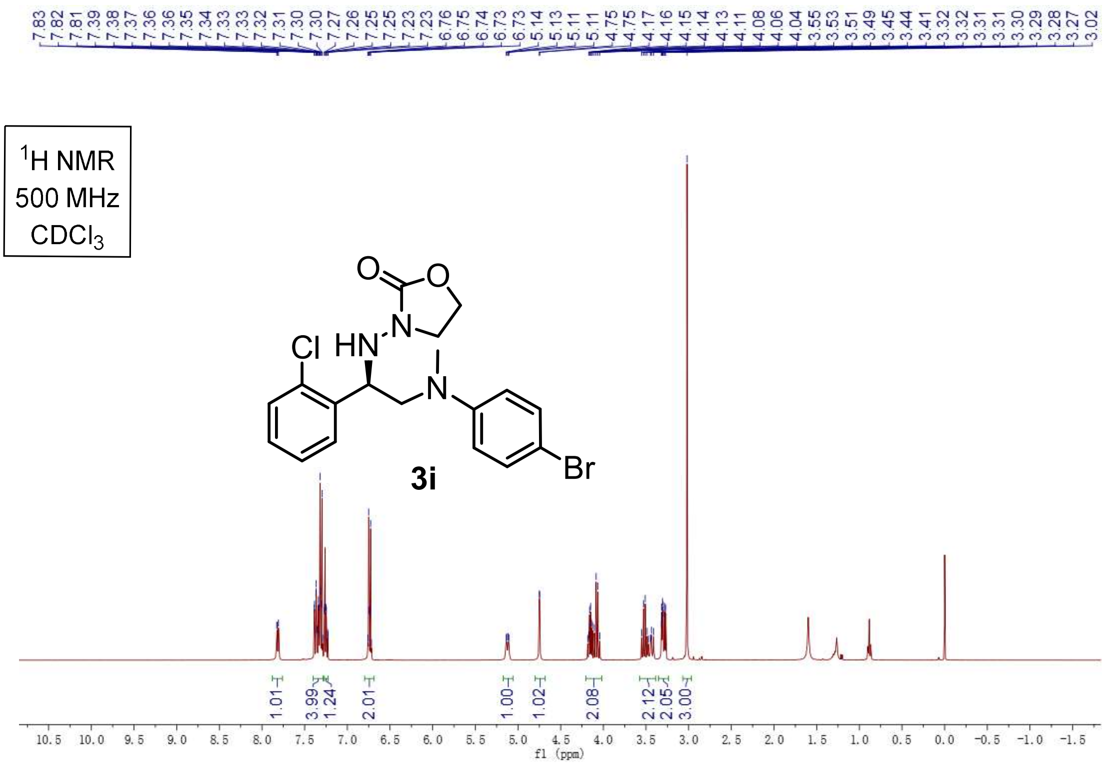

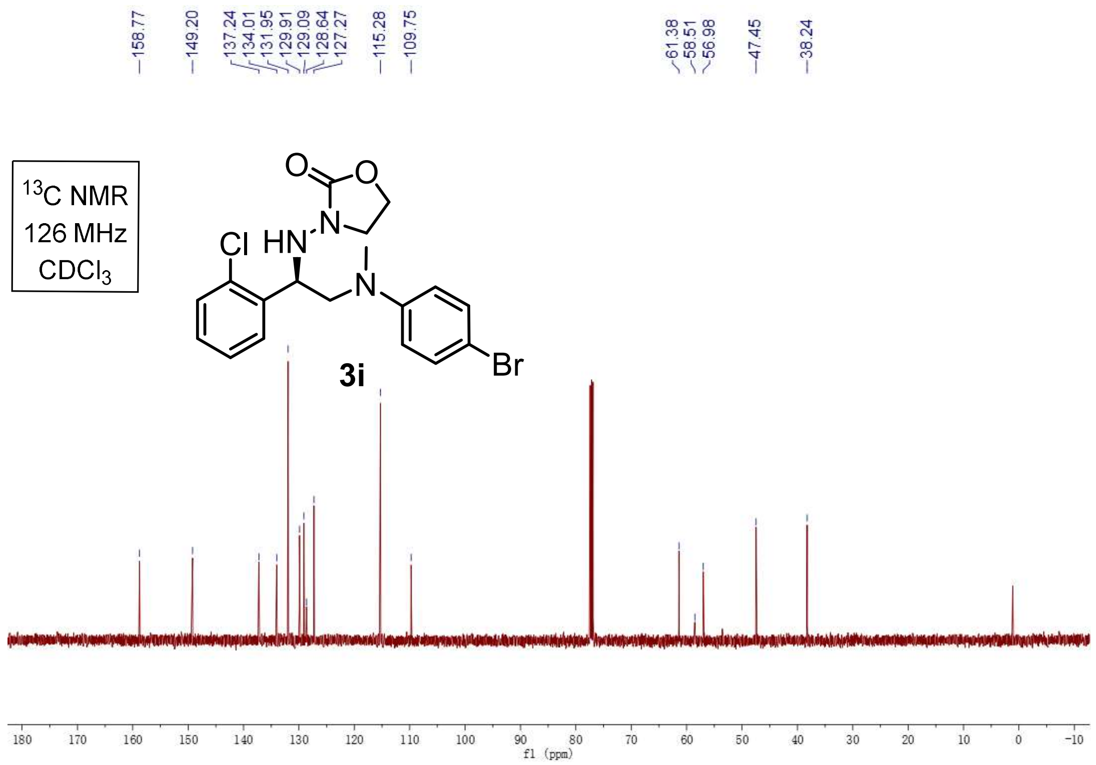

Supplementary Figure 54. ^{1}H and ^{13}C-NMR of **3i**.

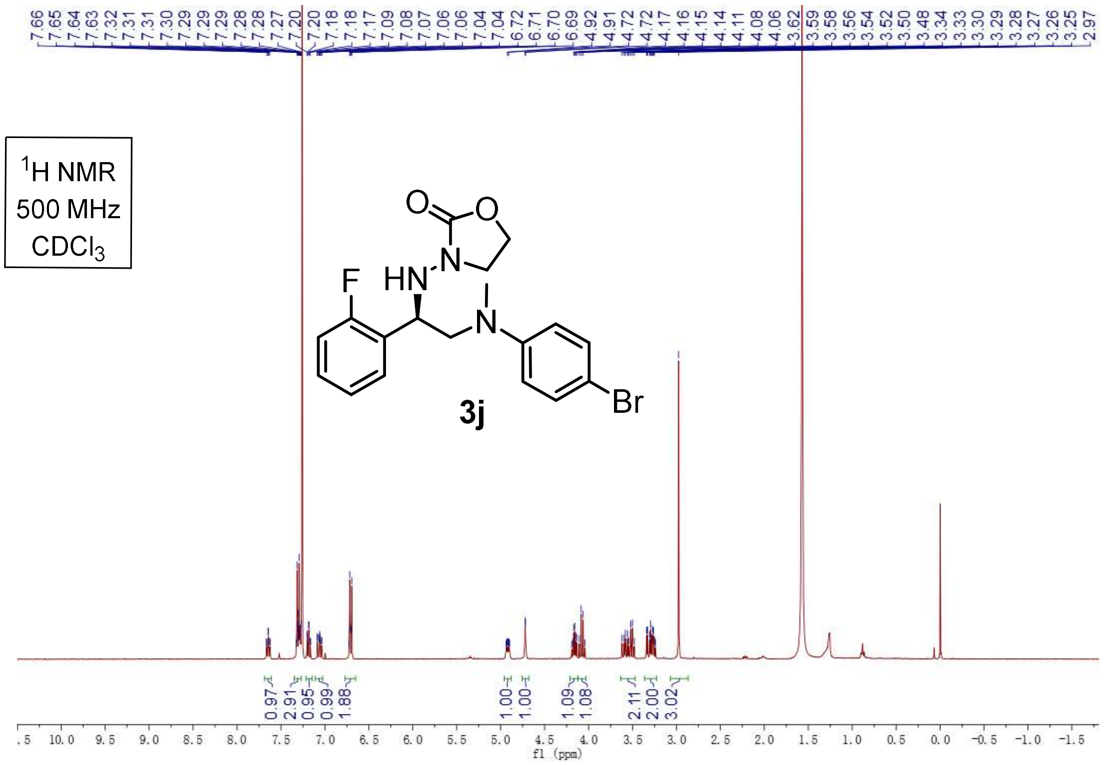

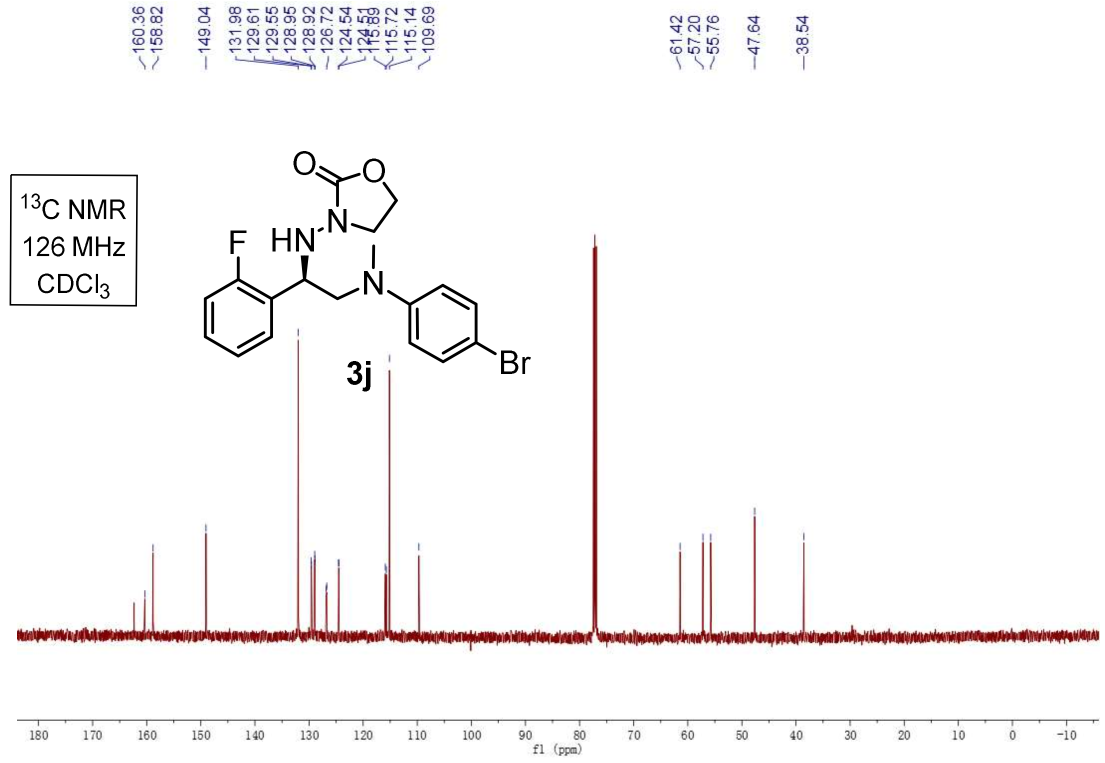

Supplementary Figure 55. ^{1}H and ^{13}C-NMR of **3j**.

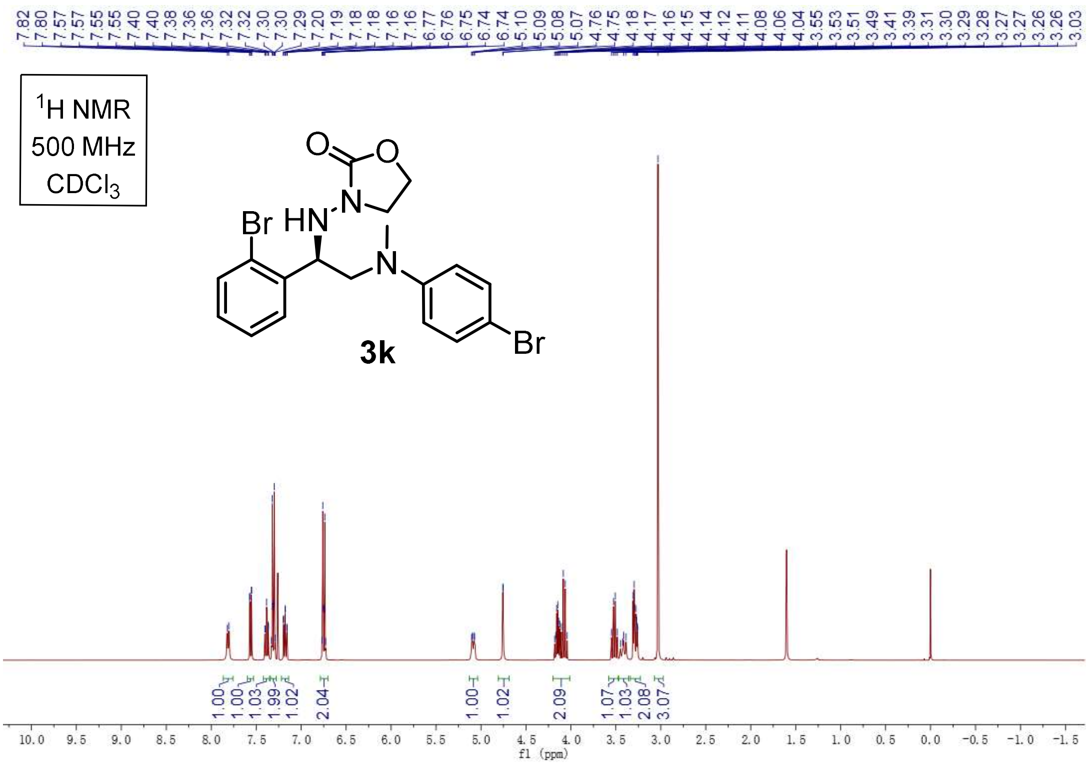

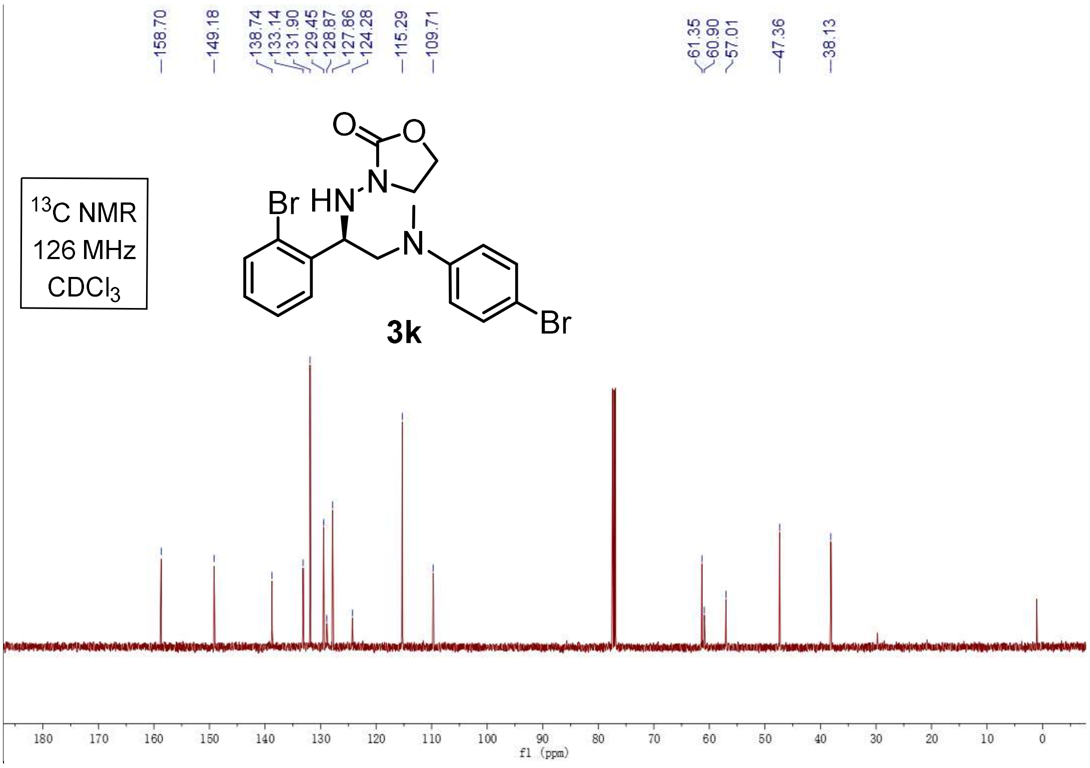

Supplementary Figure 56. ^{1}H and ^{13}C-NMR of **3k**.

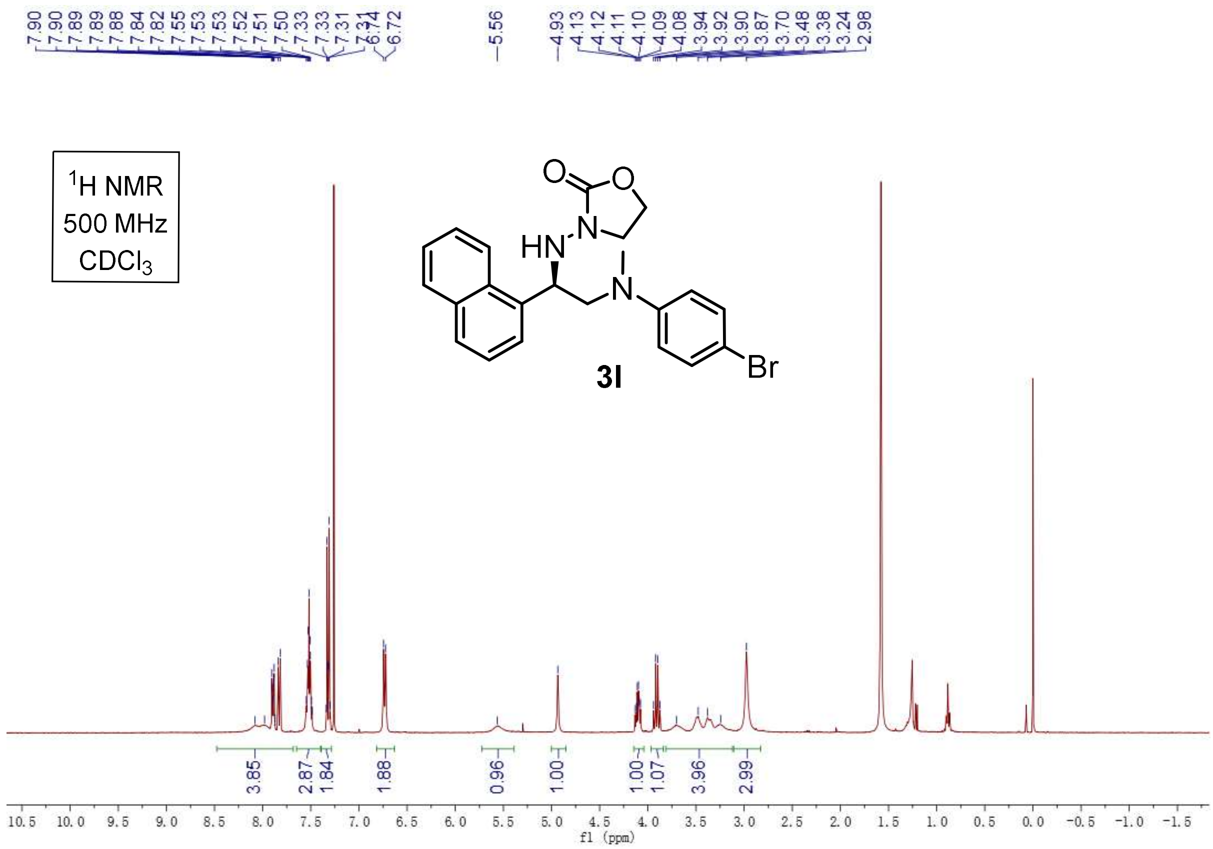

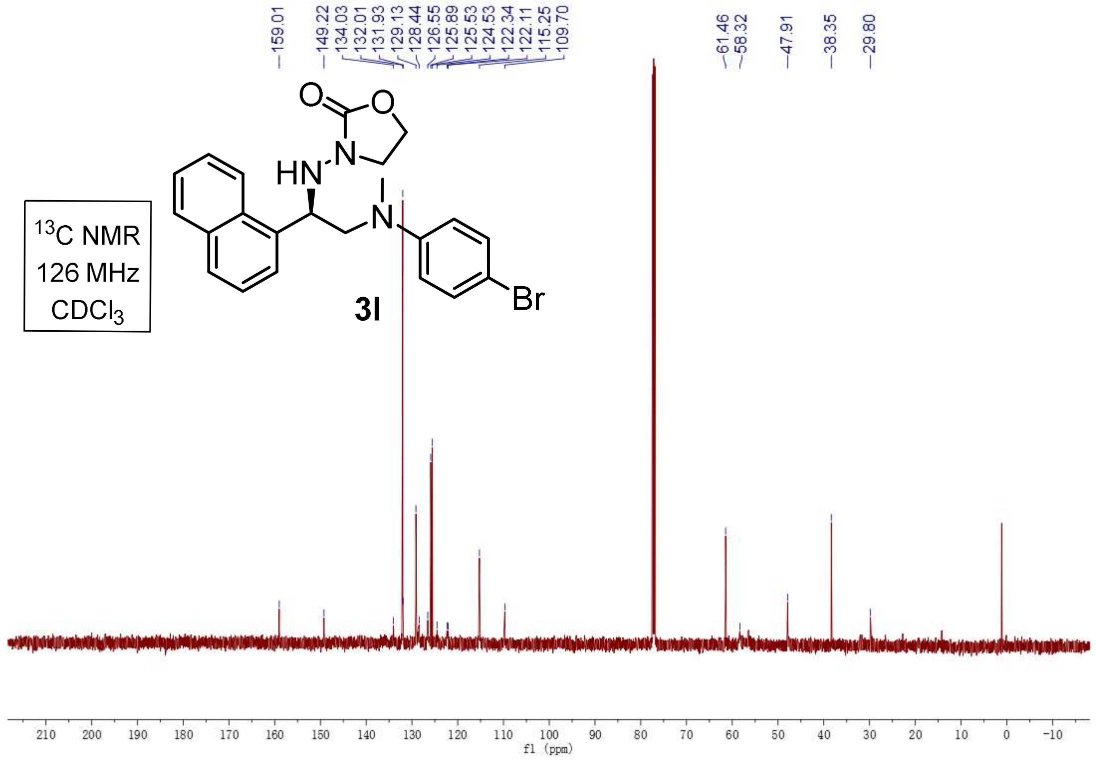

Supplementary Figure 57. ^{1}H and ^{13}C-NMR of **3l**.

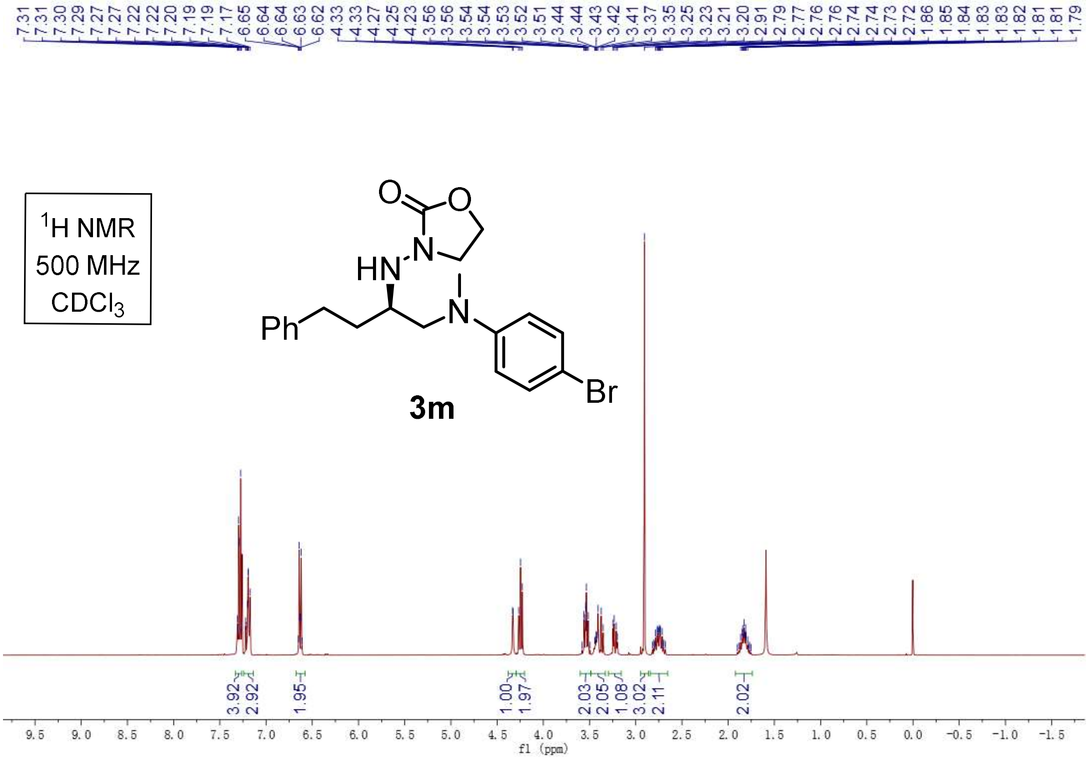

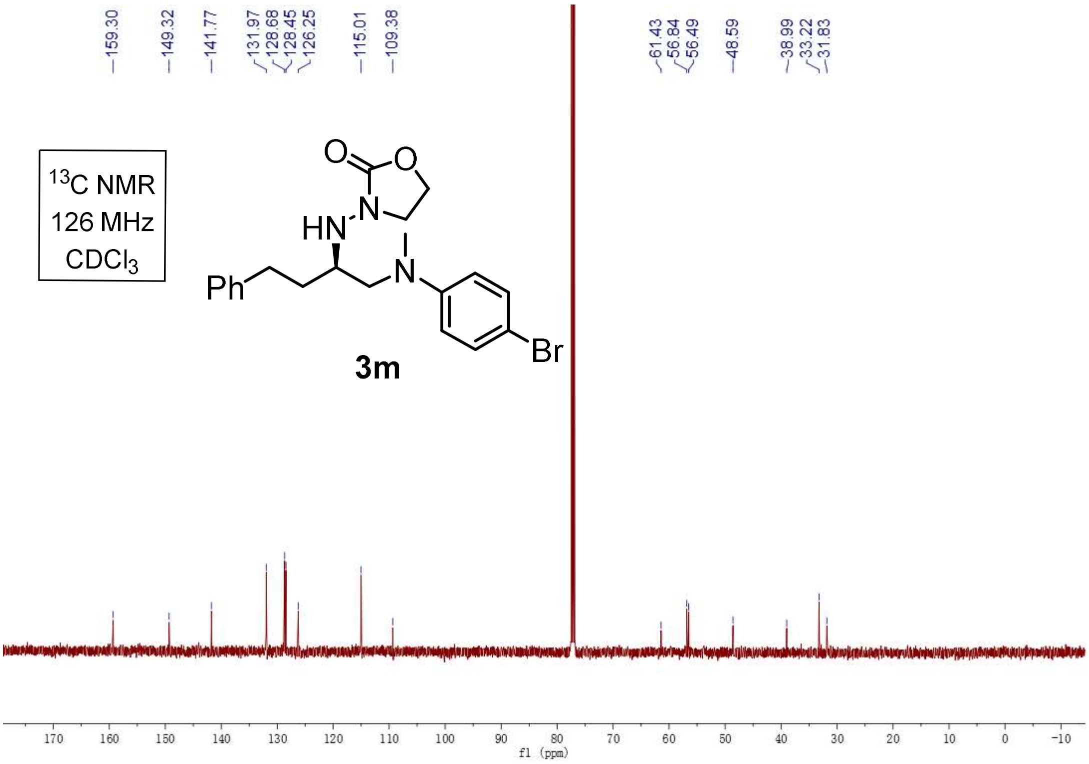

Supplementary Figure 58. ^{1}H and ^{13}C-NMR of **3m**.

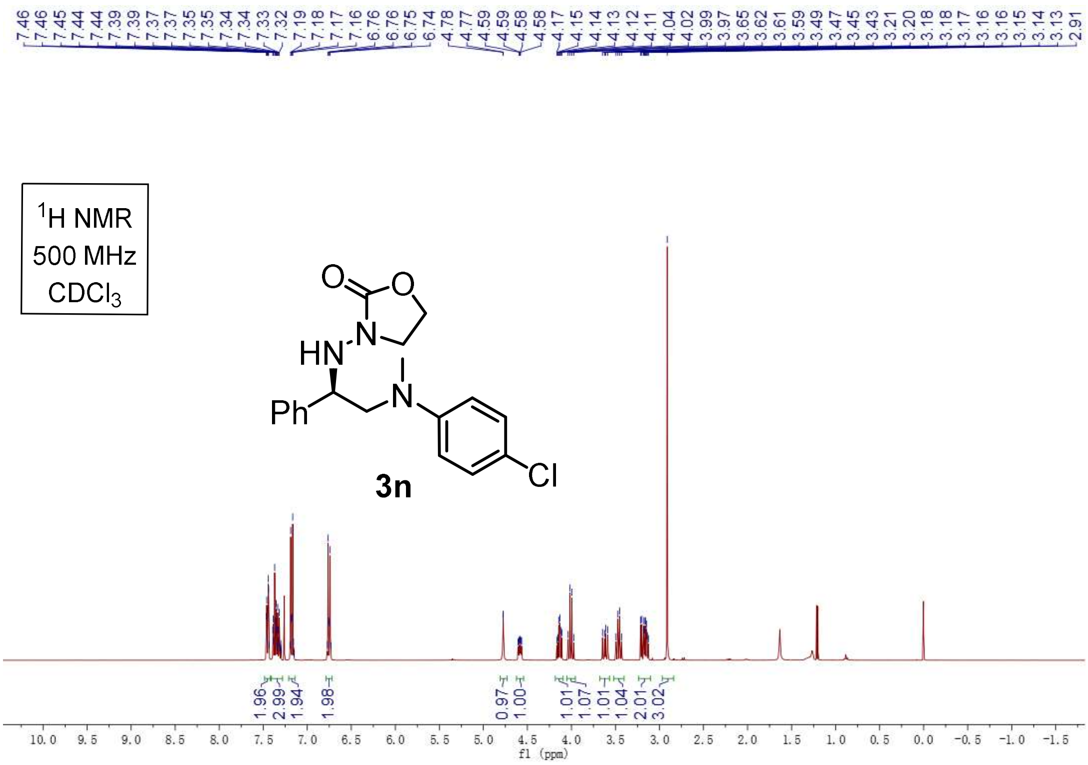

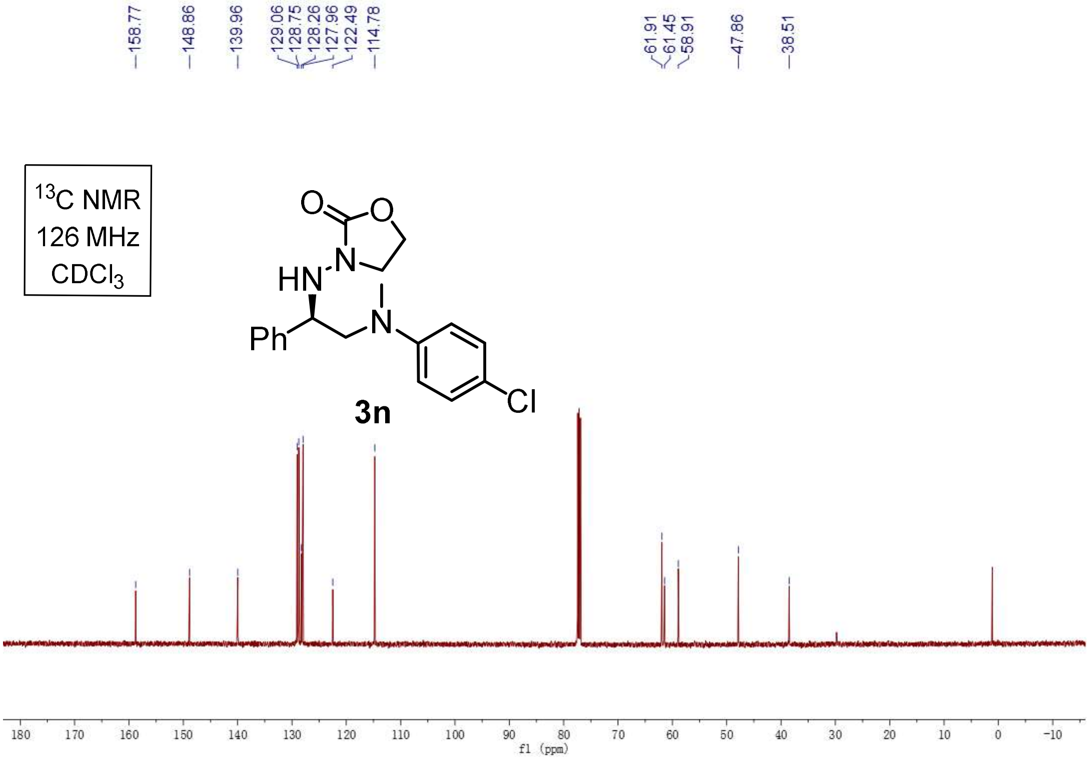

Supplementary Figure 59. ^{1}H and ^{13}C-NMR of **3n**.

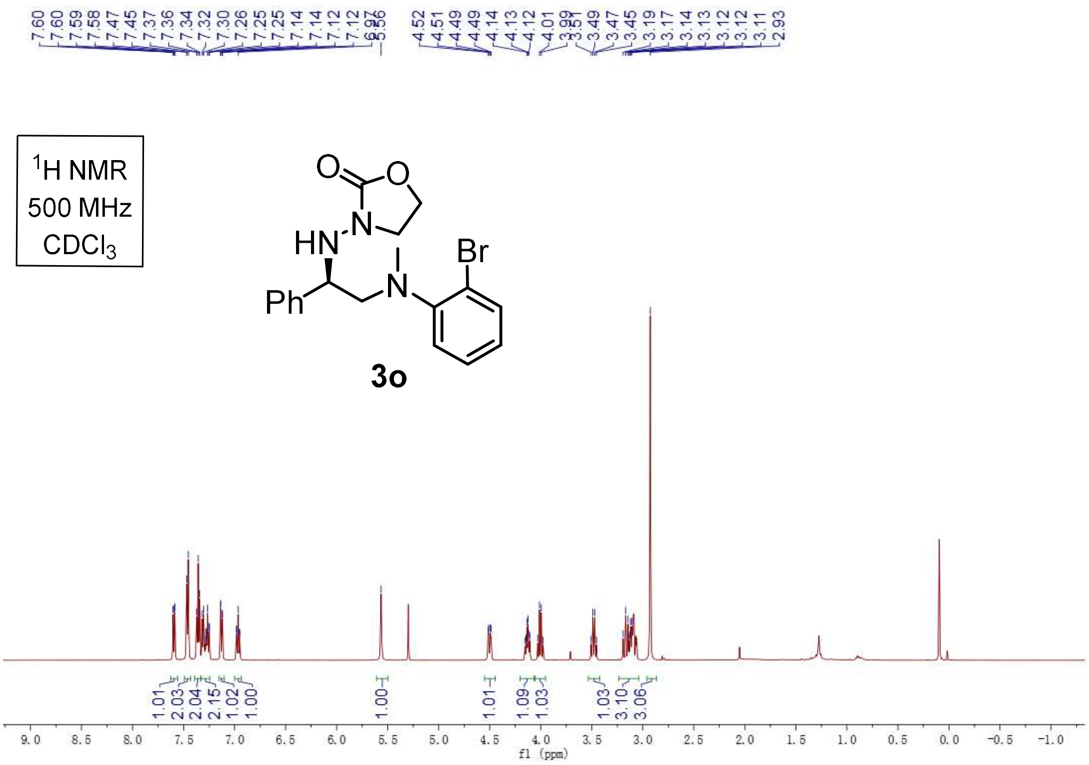

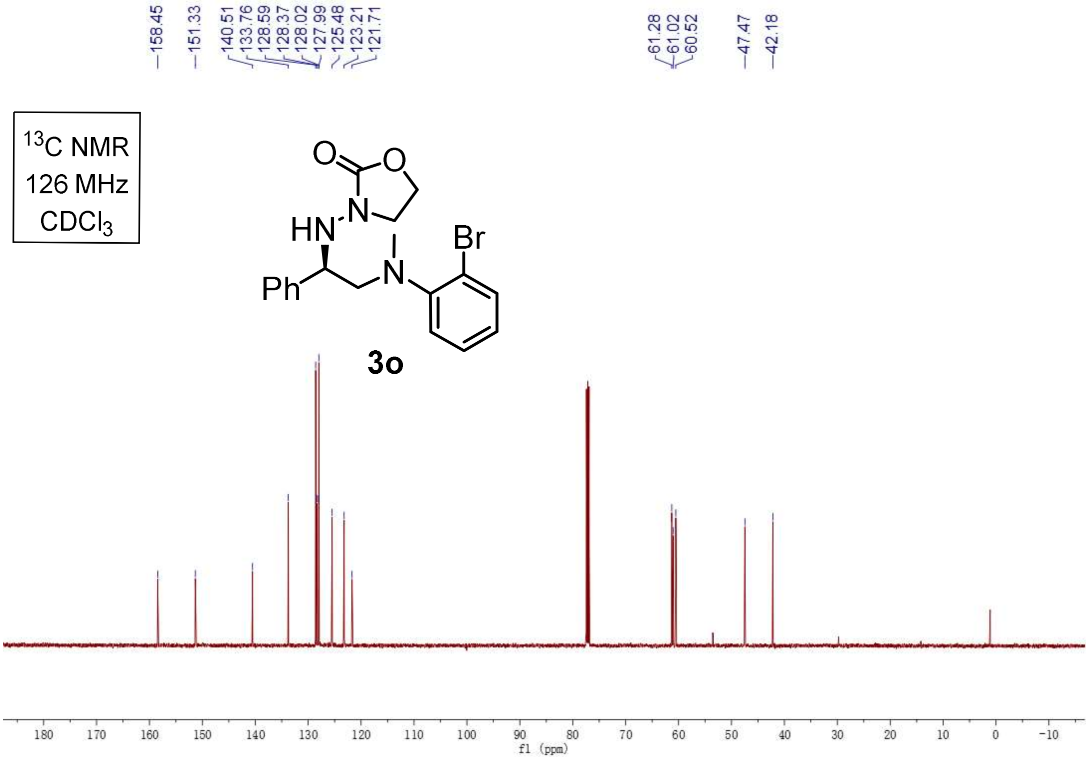

Supplementary Figure 60. ^{1}H and ^{13}C-NMR of **3o**.

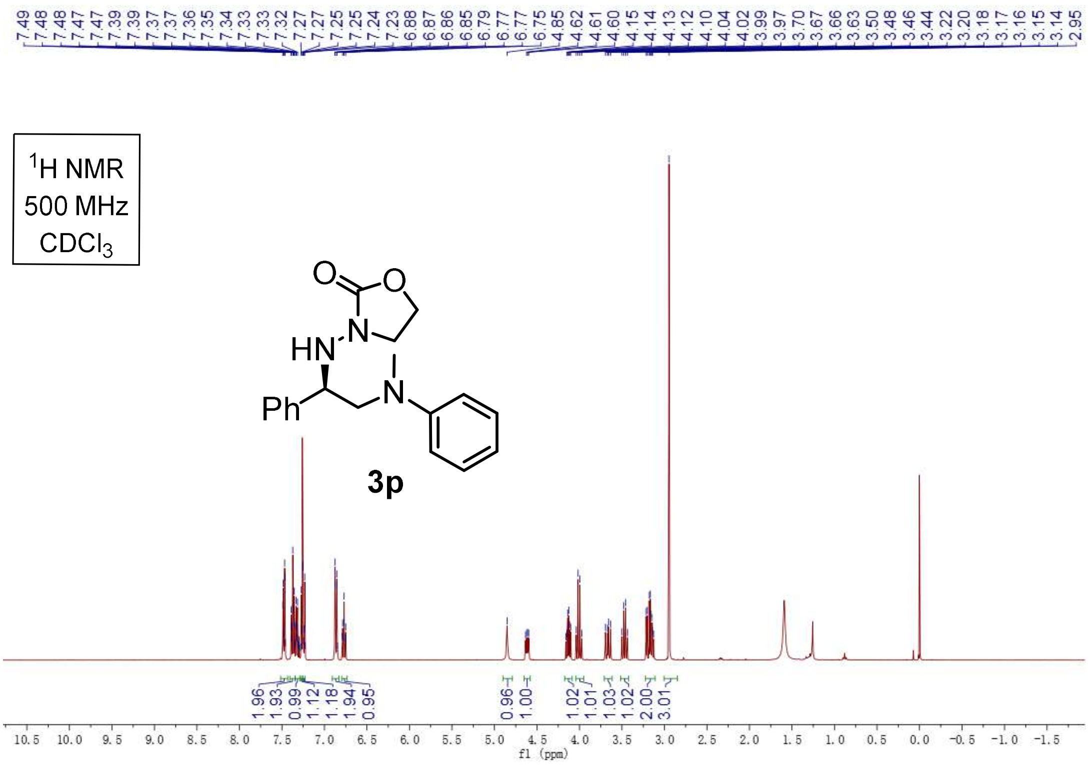

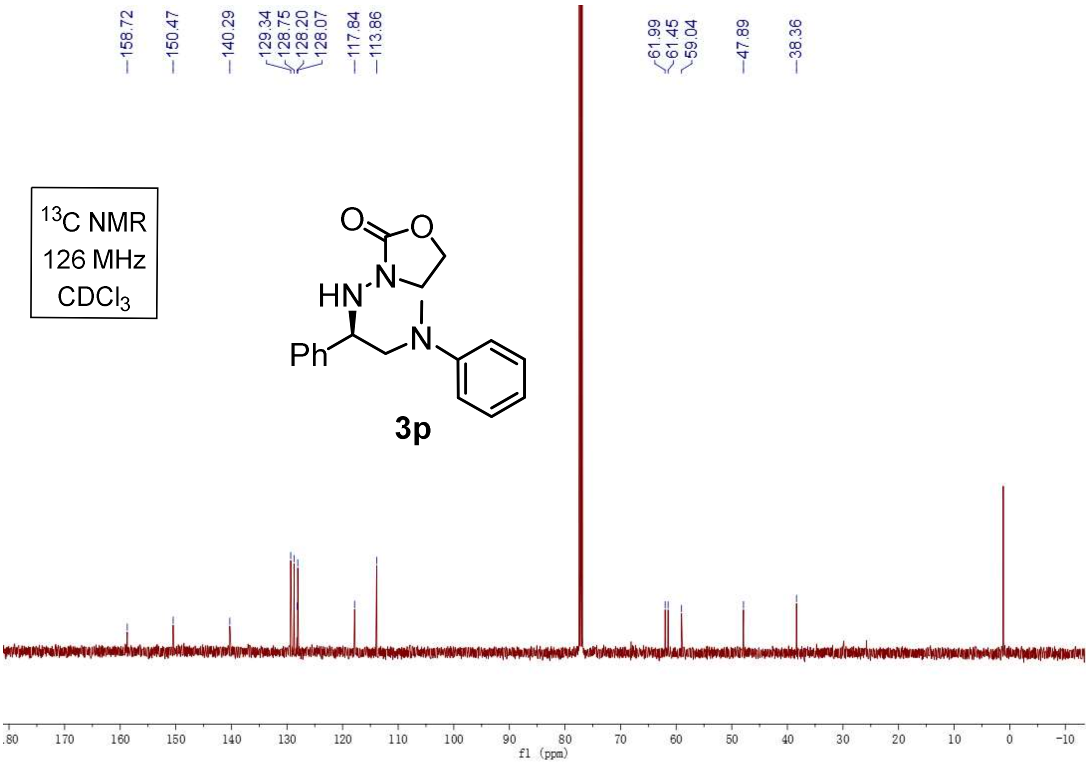

Supplementary Figure 61. ^{1}H and ^{13}C-NMR of **3p**.

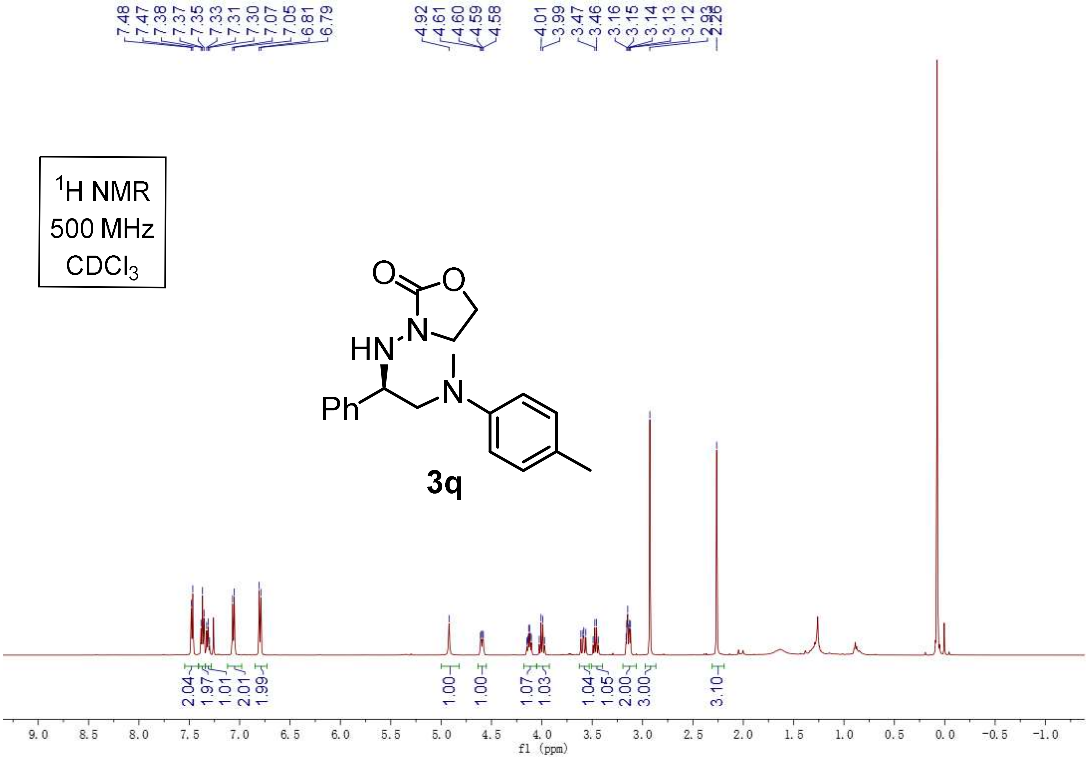

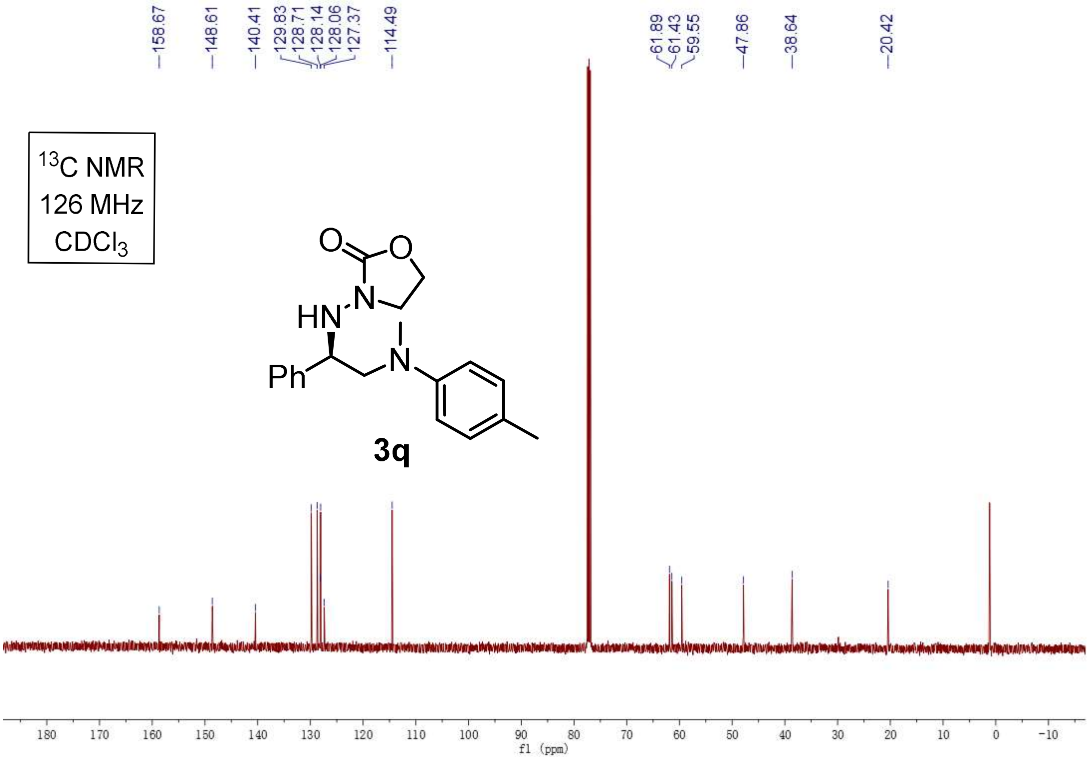

Supplementary Figure 62. ^{1}H and ^{13}C-NMR of **3q**

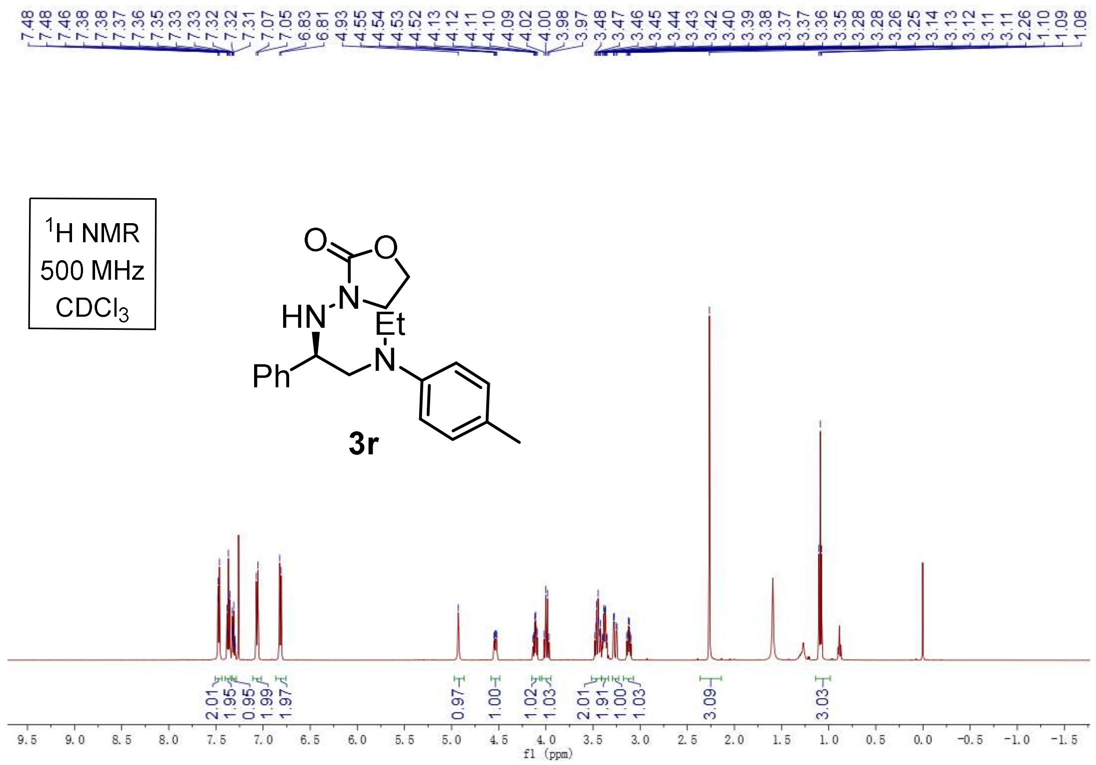

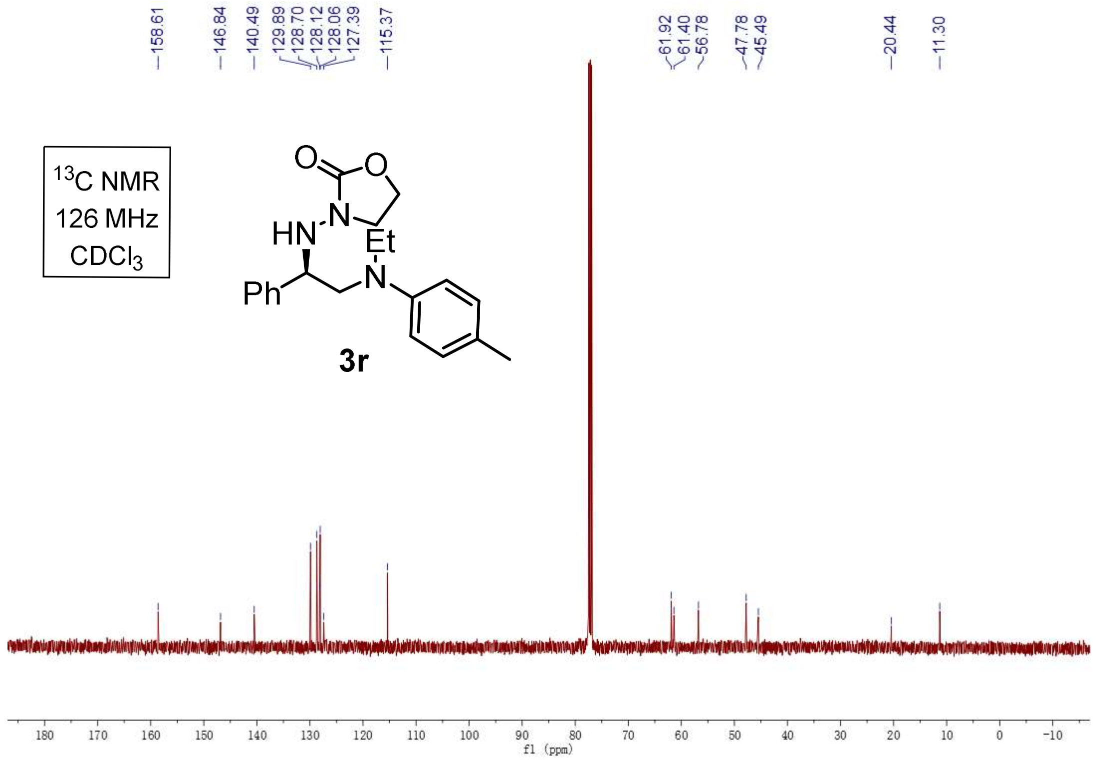

Supplementary Figure 63. ^{1}H and ^{13}C-NMR of **3r**.

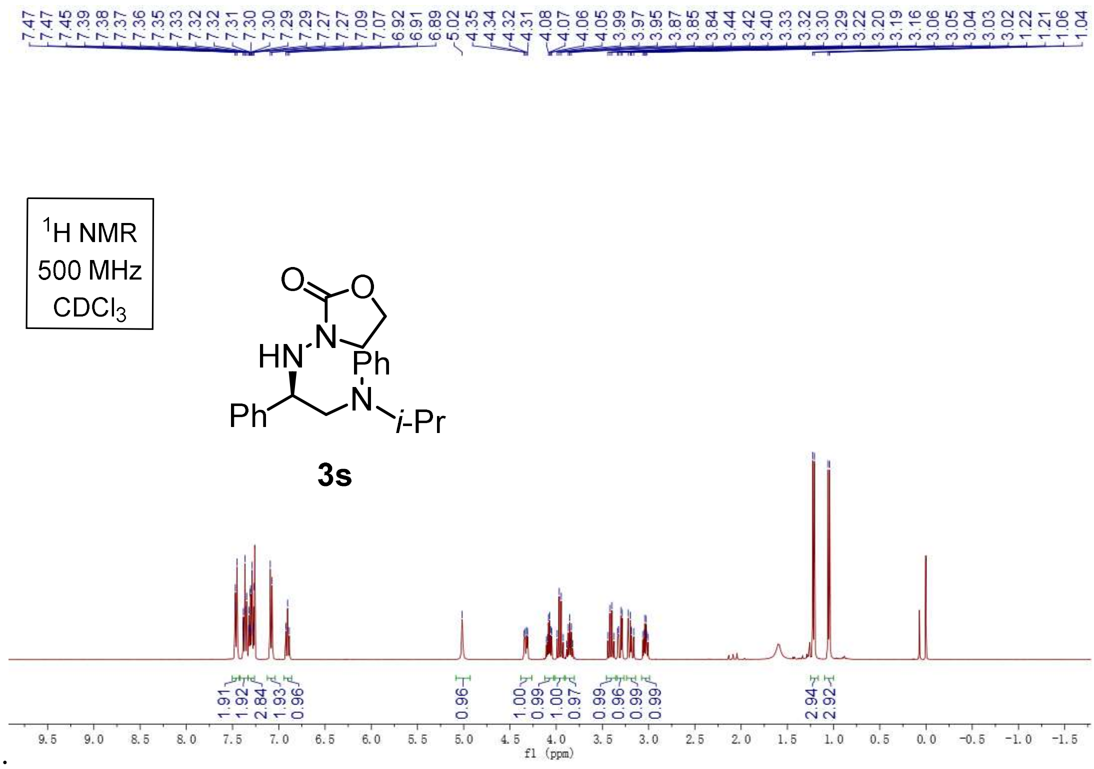

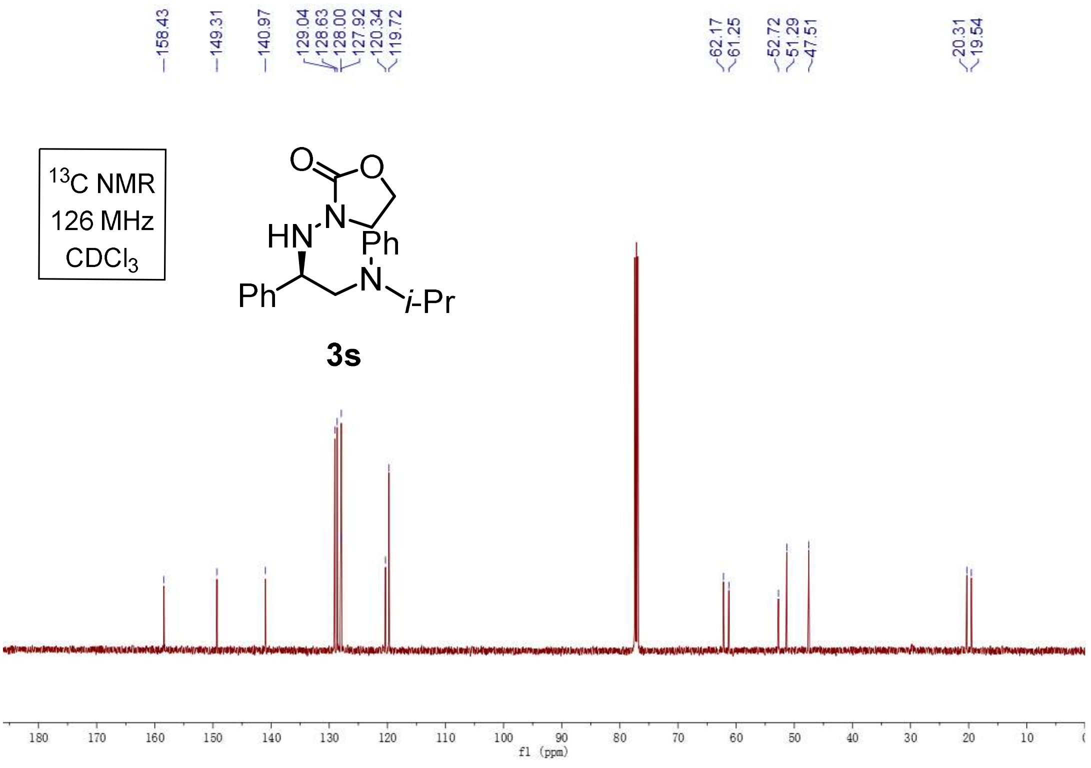

Supplementary Figure 64. ^{1}H and ^{13}C-NMR of **3s**.

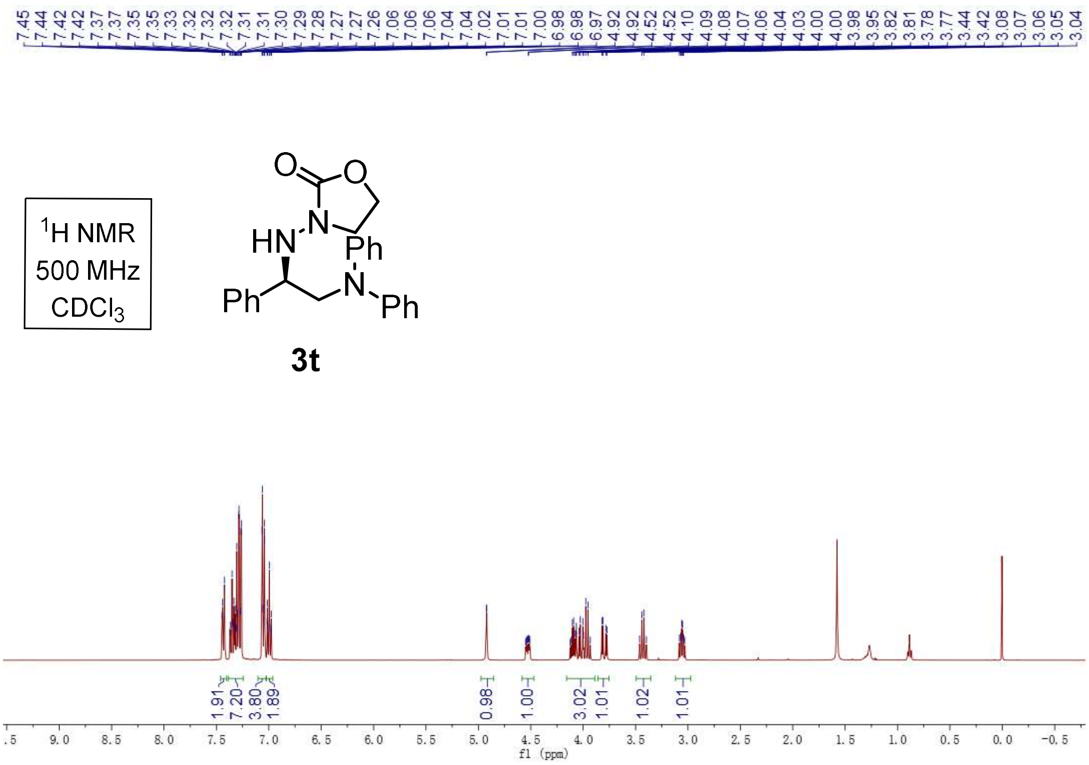

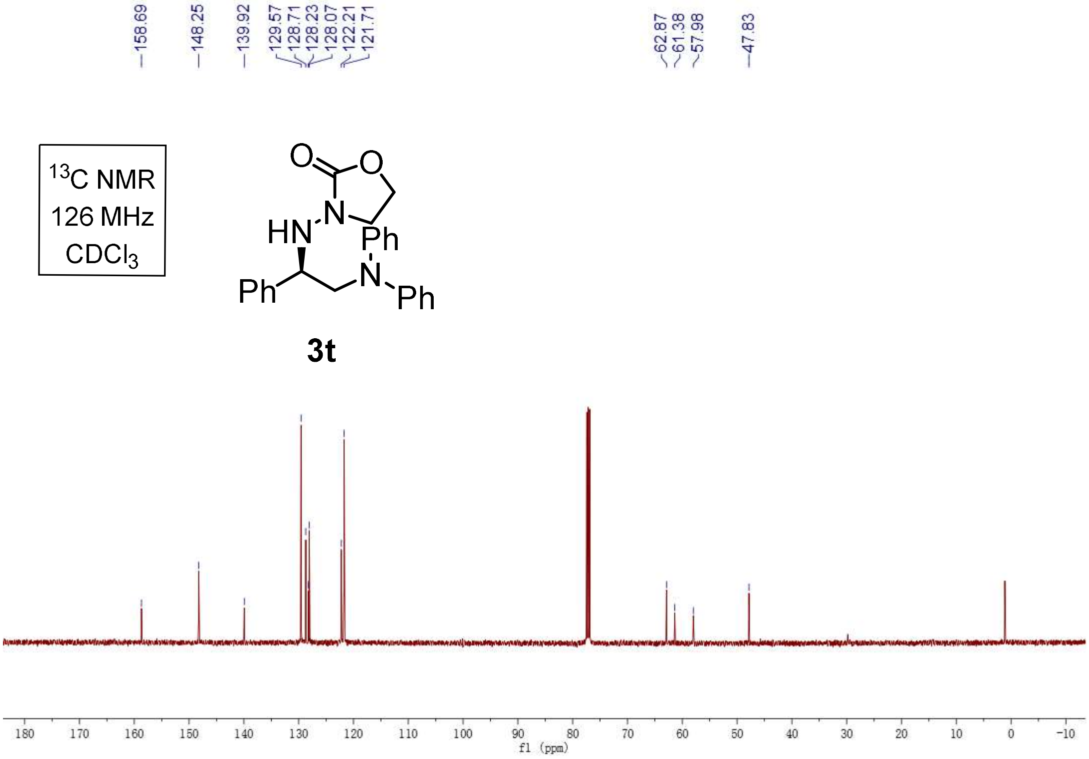

Supplementary Figure 65. ^{1}H and ^{13}C-NMR of **3t**.

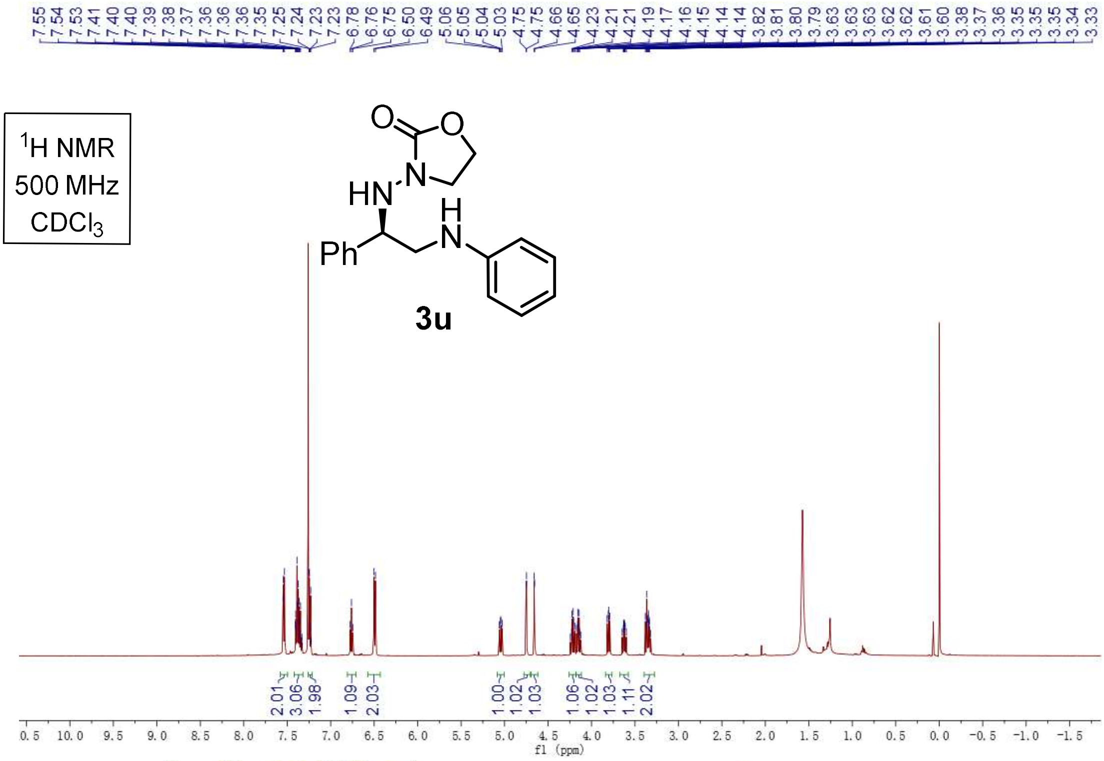

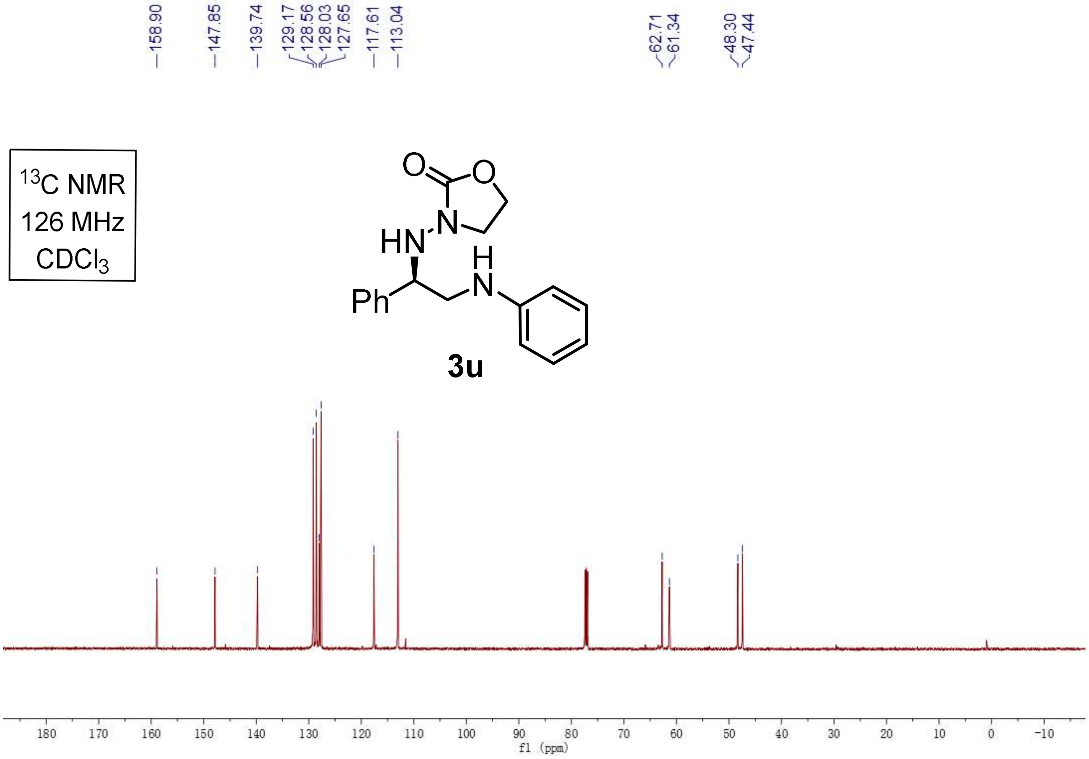

Supplementary Figure 66. ^{1}H and ^{13}C-NMR of **3u**.

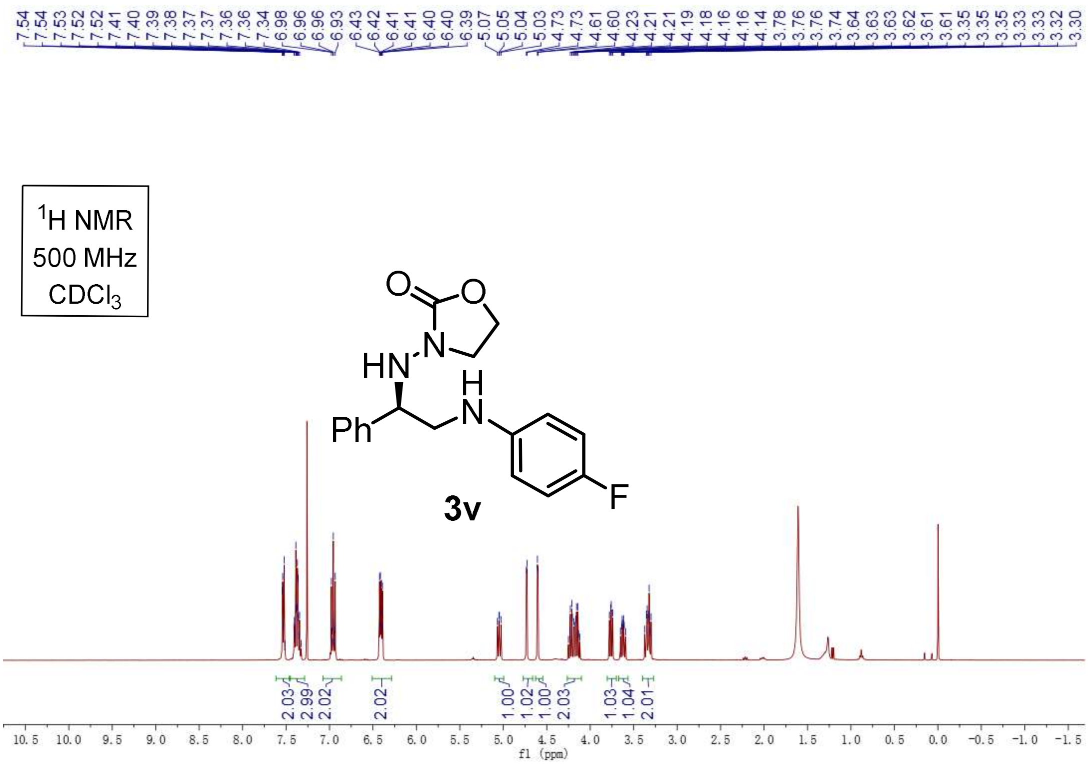

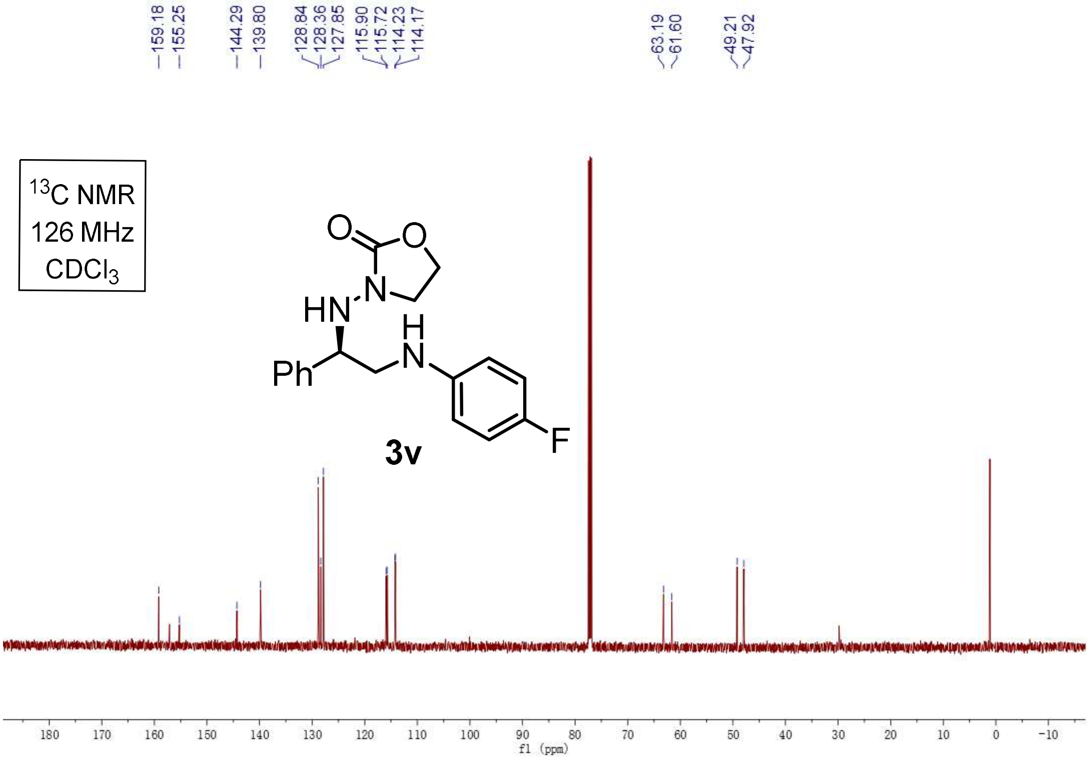

Supplementary Figure 67. ^{1}H and ^{13}C-NMR of **3v**.

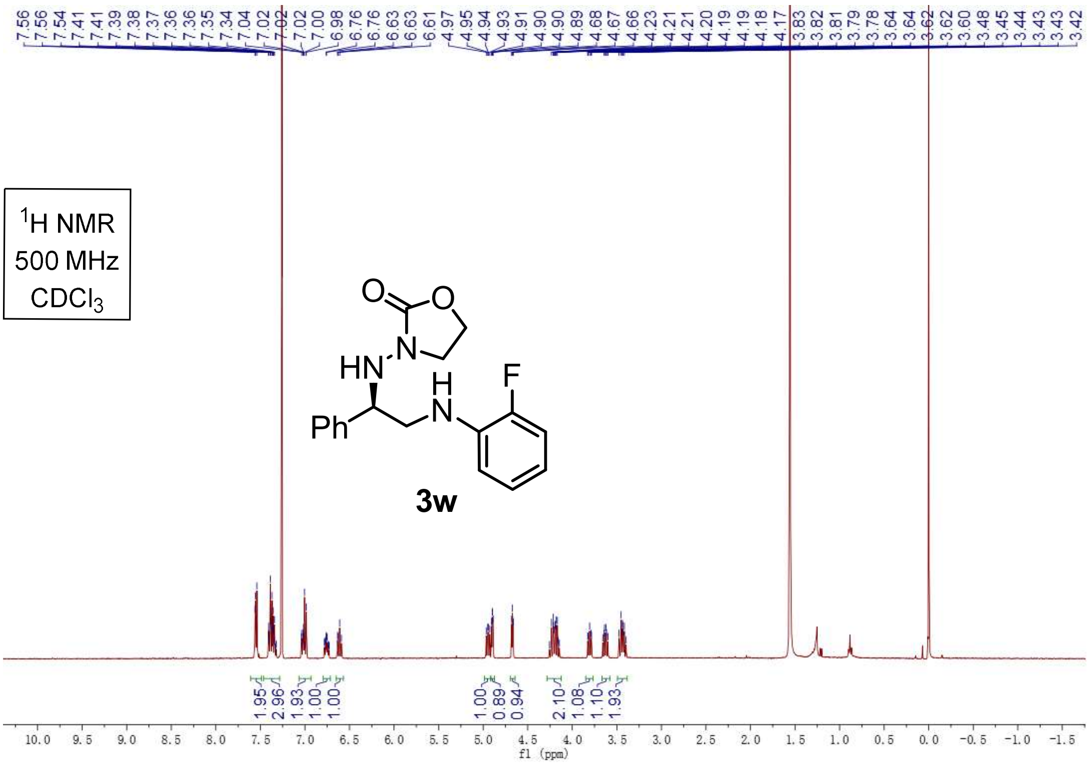

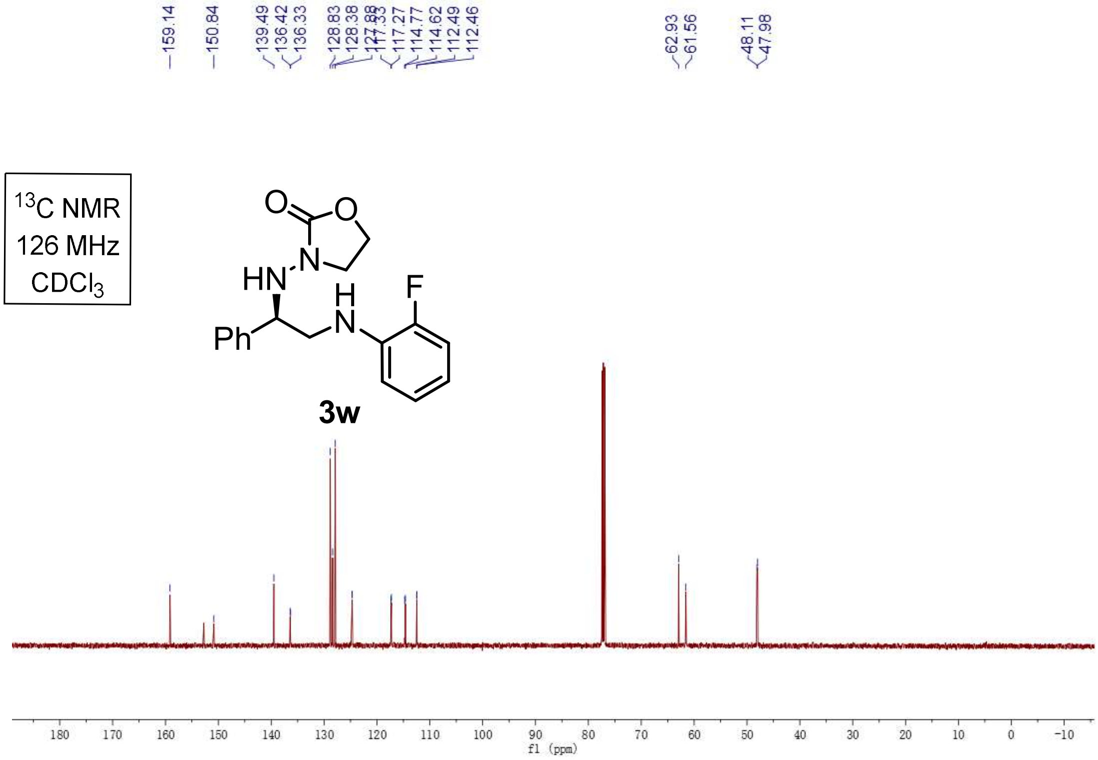

Supplementary Figure 68. ^{1}H and ^{13}C-NMR of **3w**.

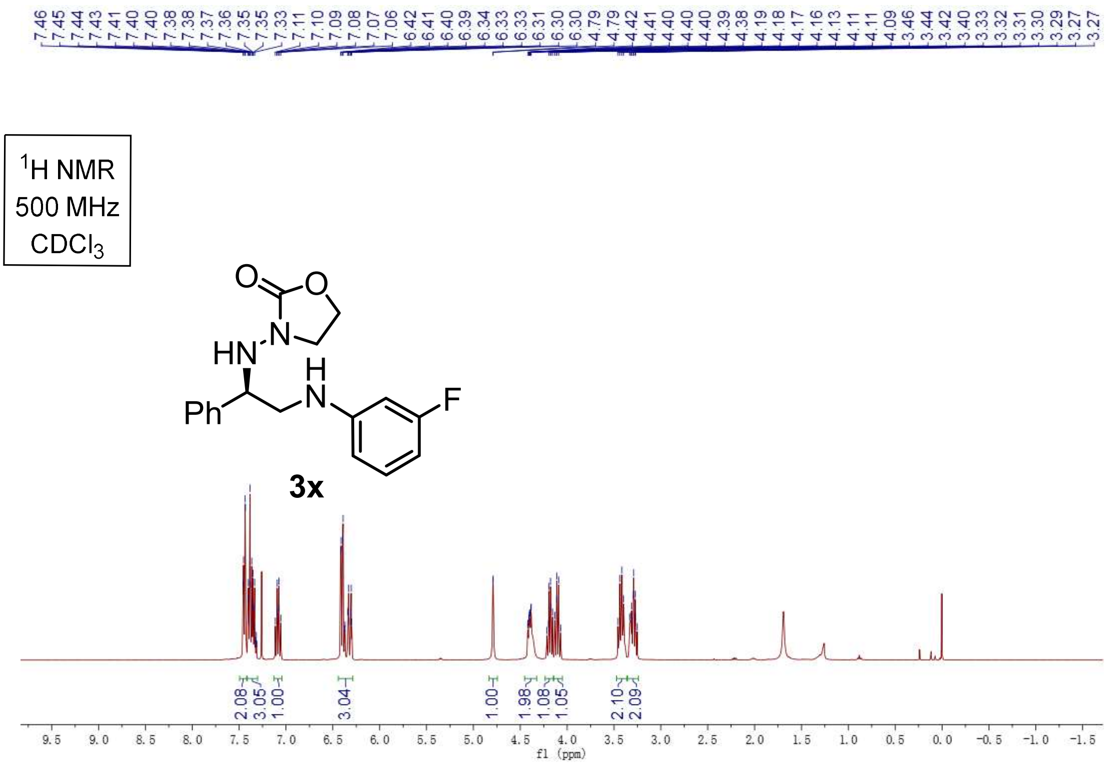

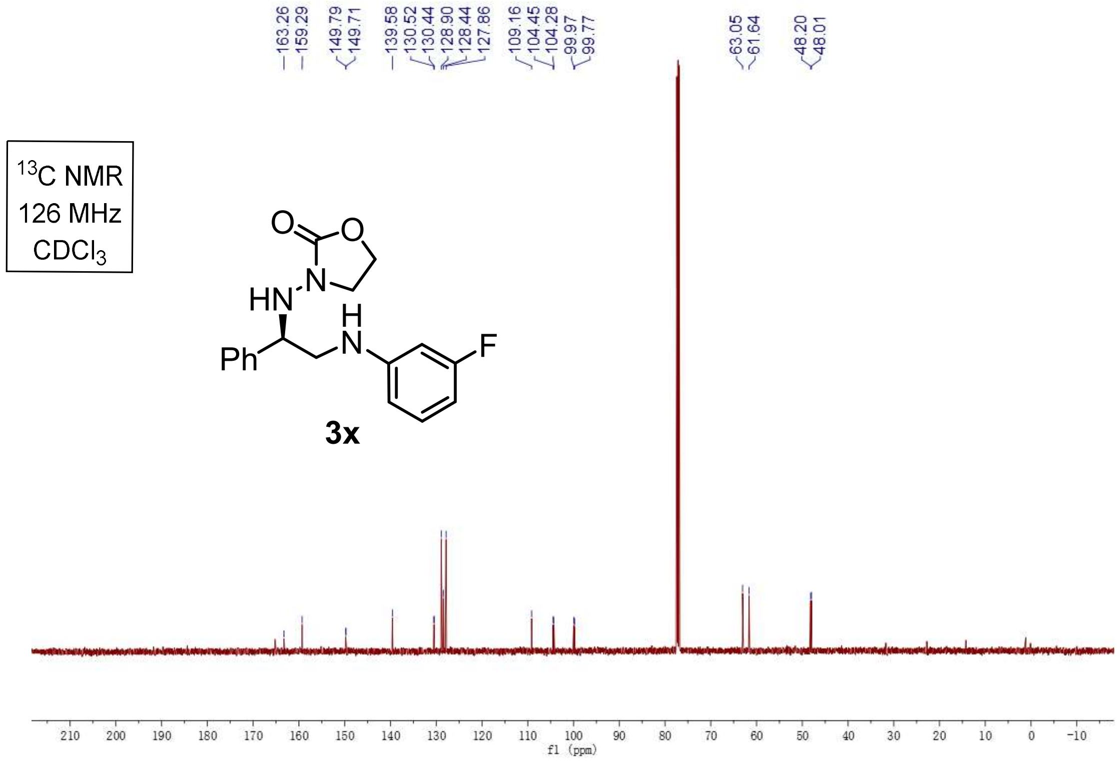

Supplementary Figure 69. ^{1}H and ^{13}C-NMR of **3x**.

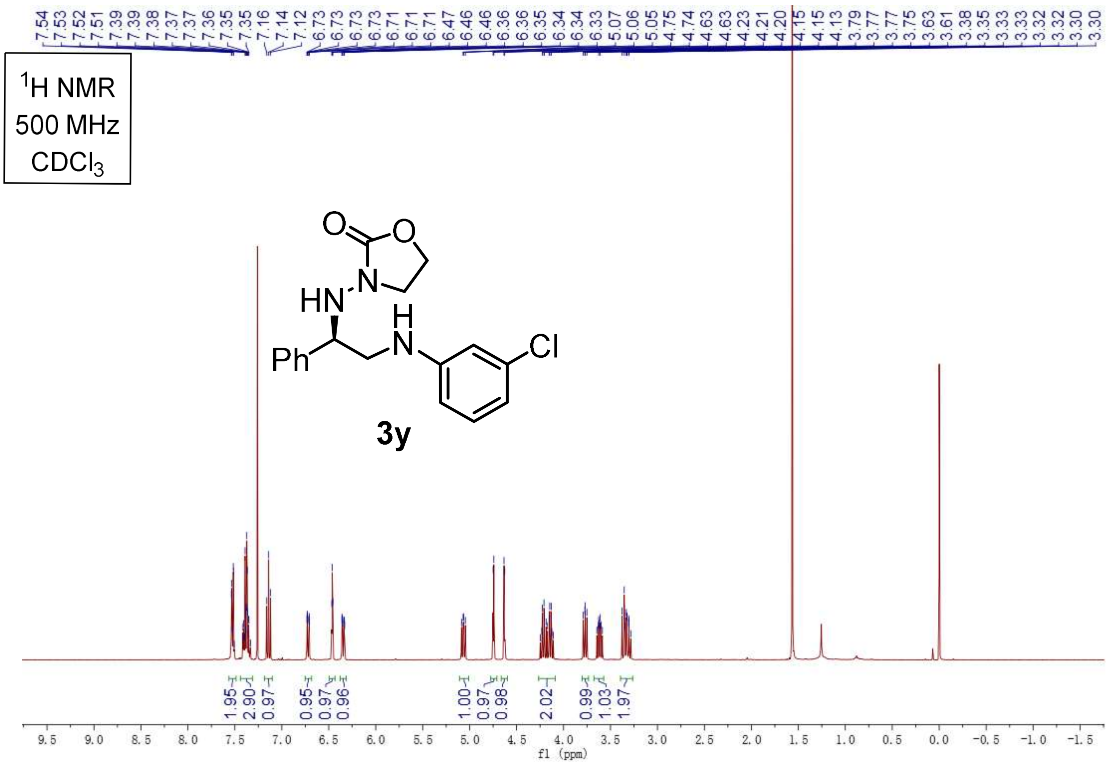

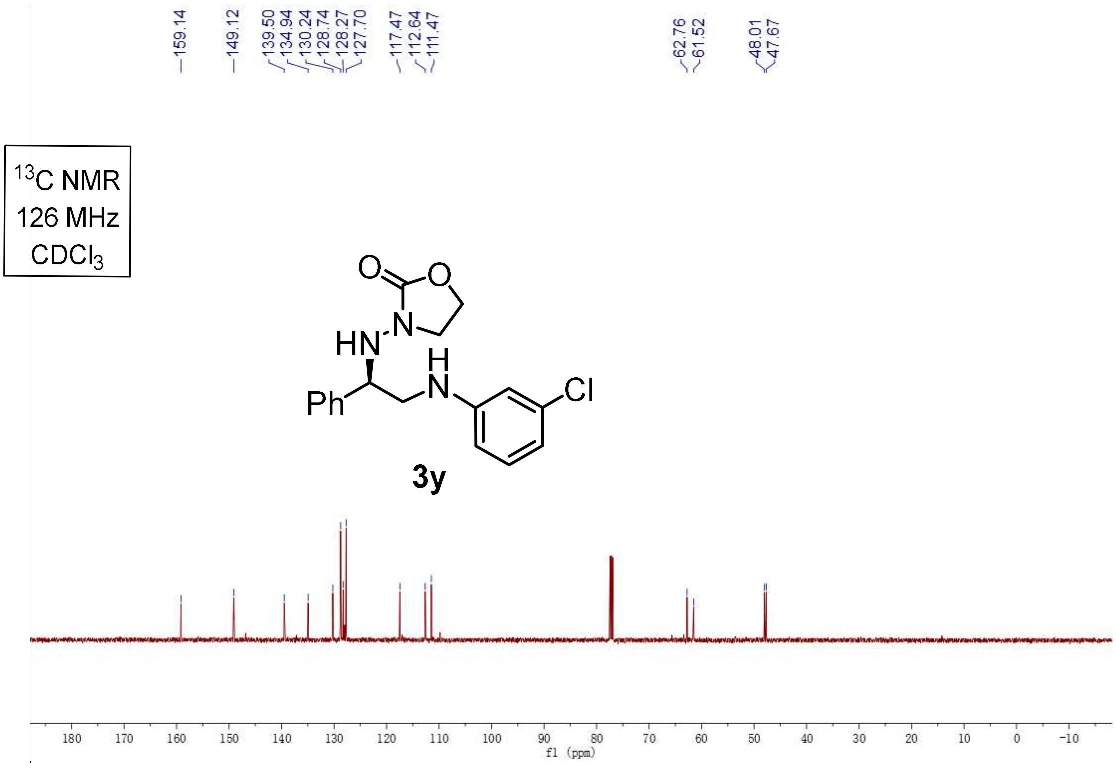

Supplementary Figure 70. ^{1}H and ^{13}C-NMR of **3y**.

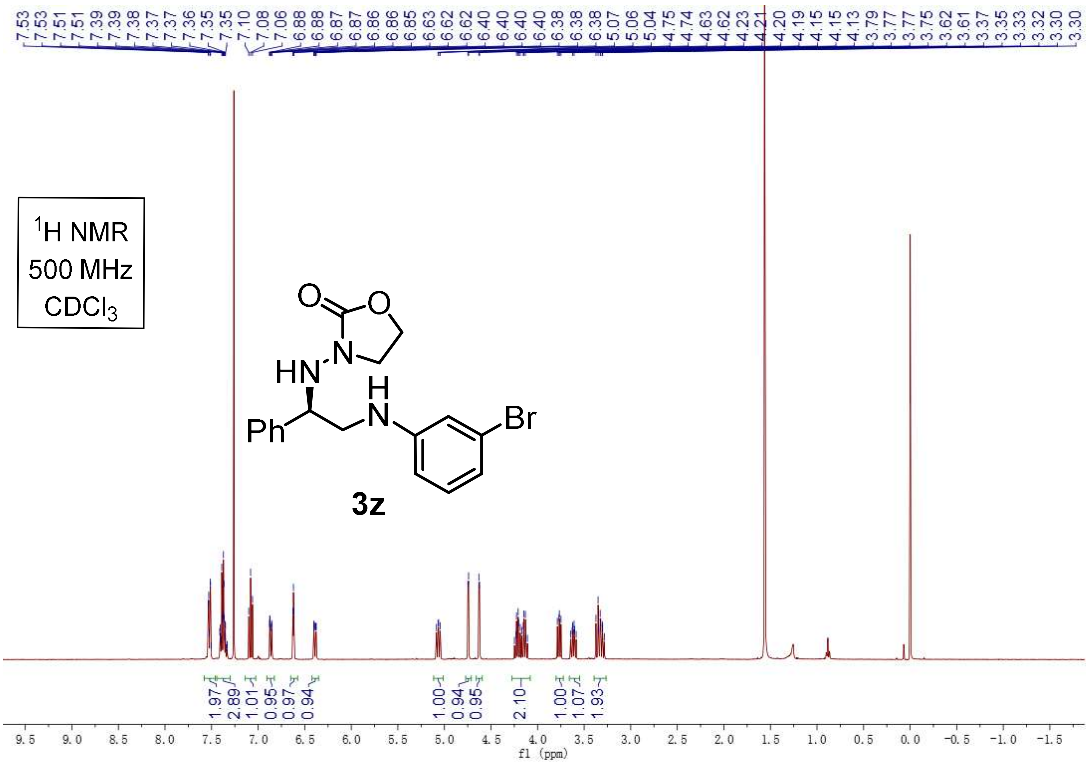

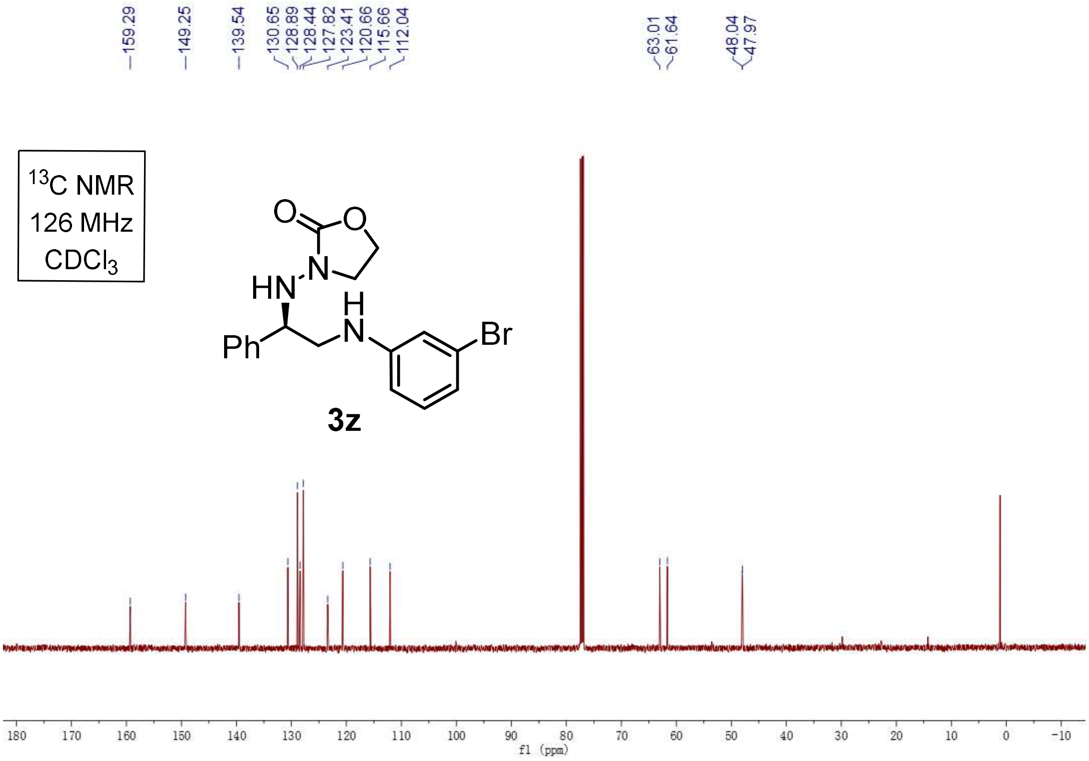

Supplementary Figure 71. ^{1}H and ^{13}C-NMR of **3z**.

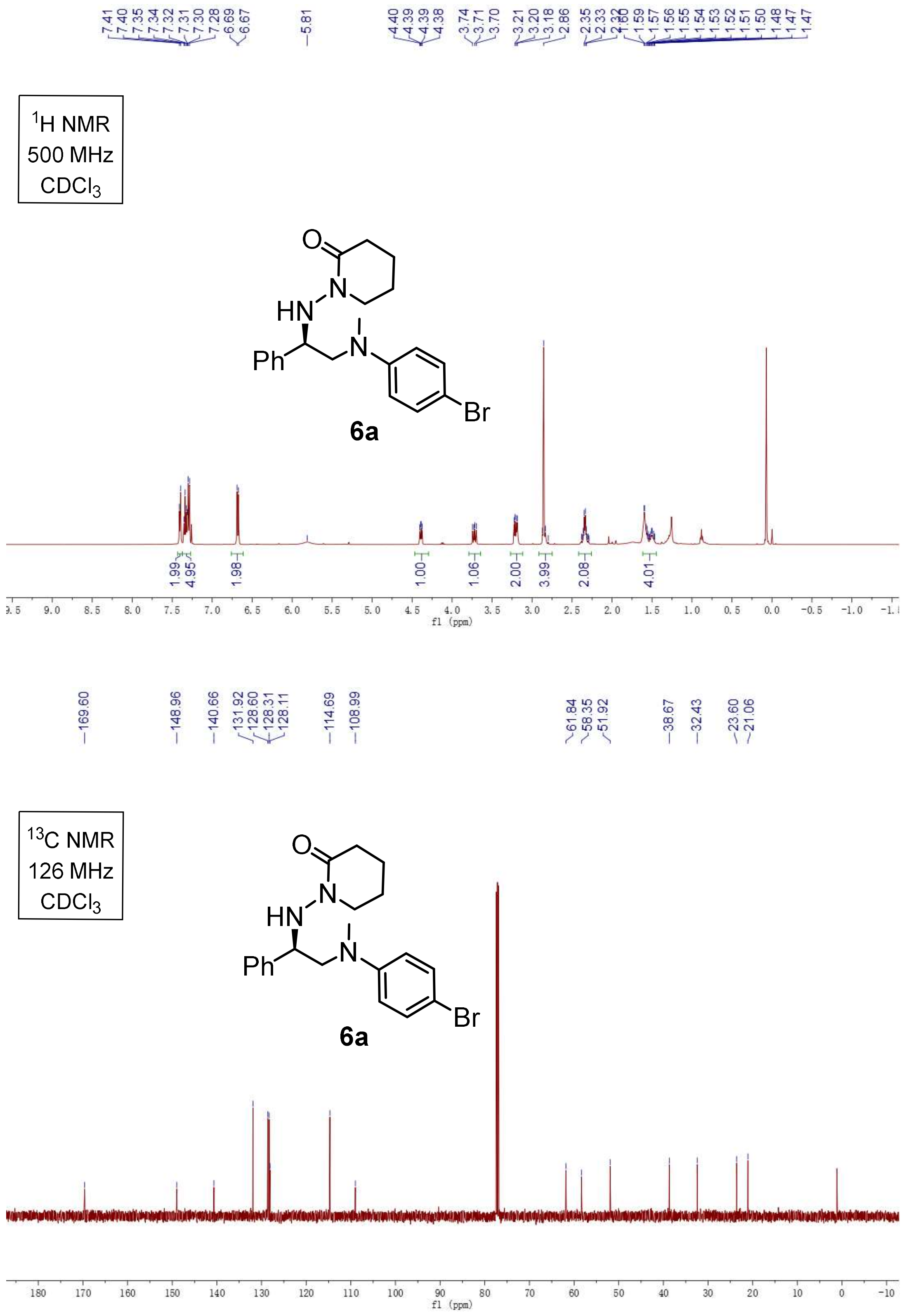

Supplementary Figure 72. ^{1}H and ^{13}C-NMR of **6a**.

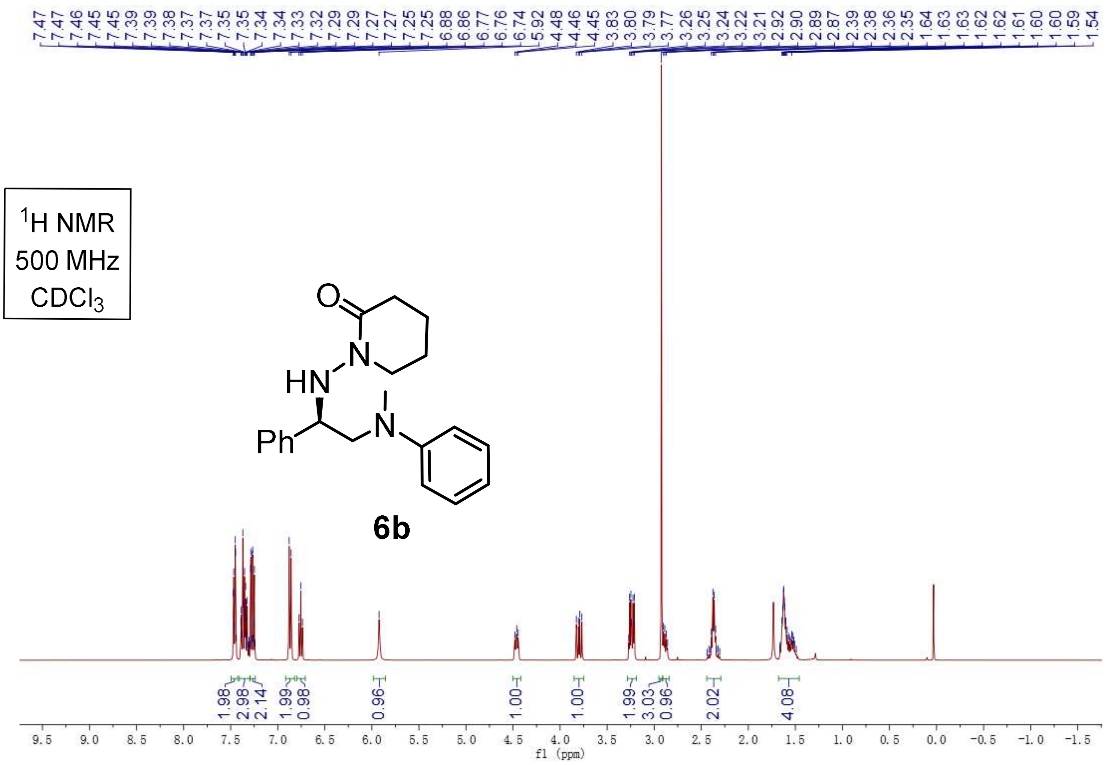

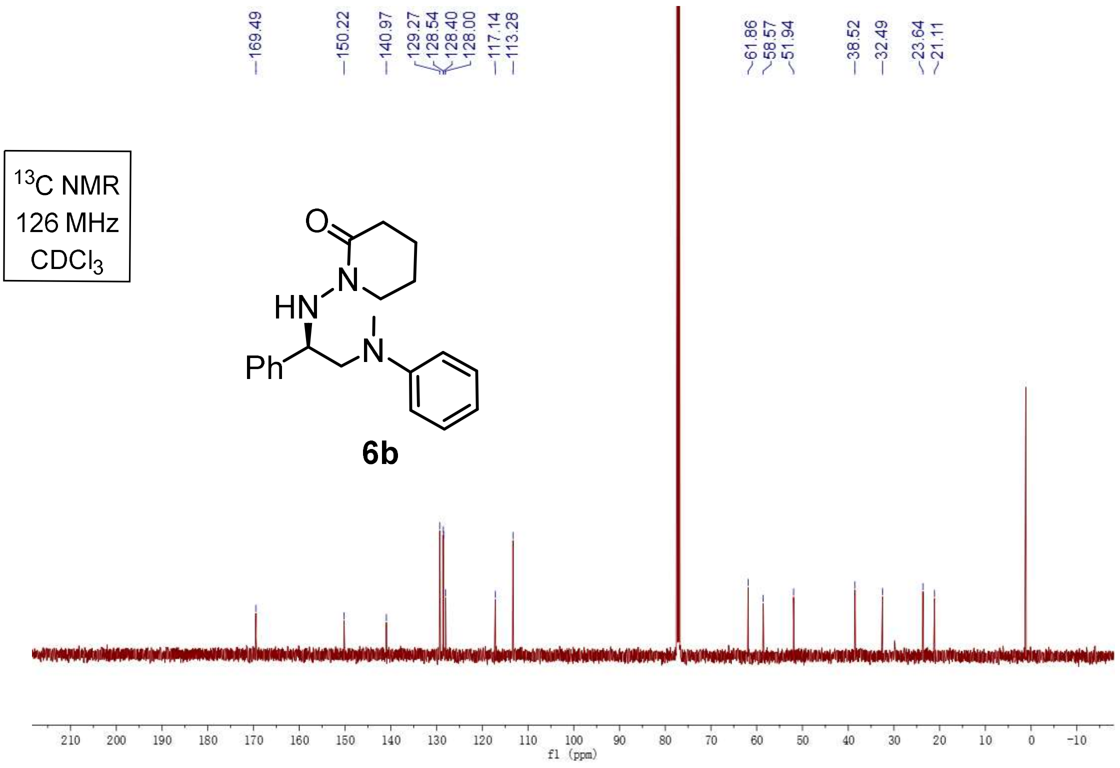

Supplementary Figure 73. ^{1}H and ^{13}C-NMR of **6b**.

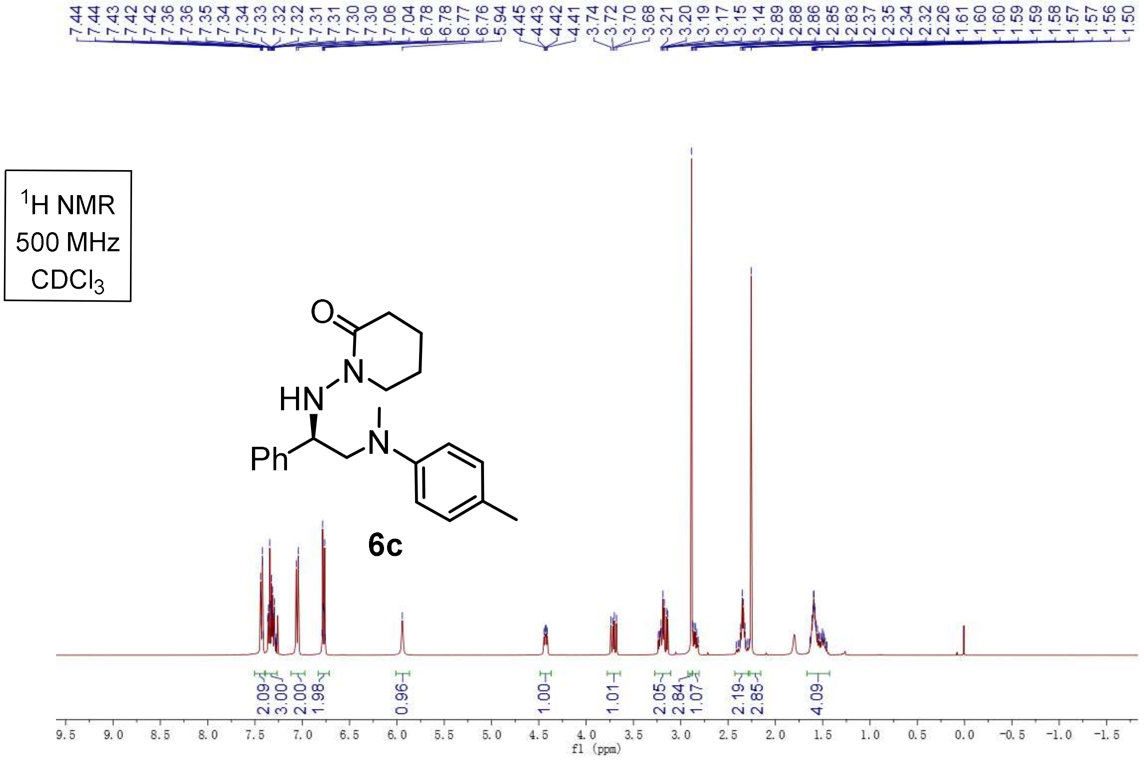

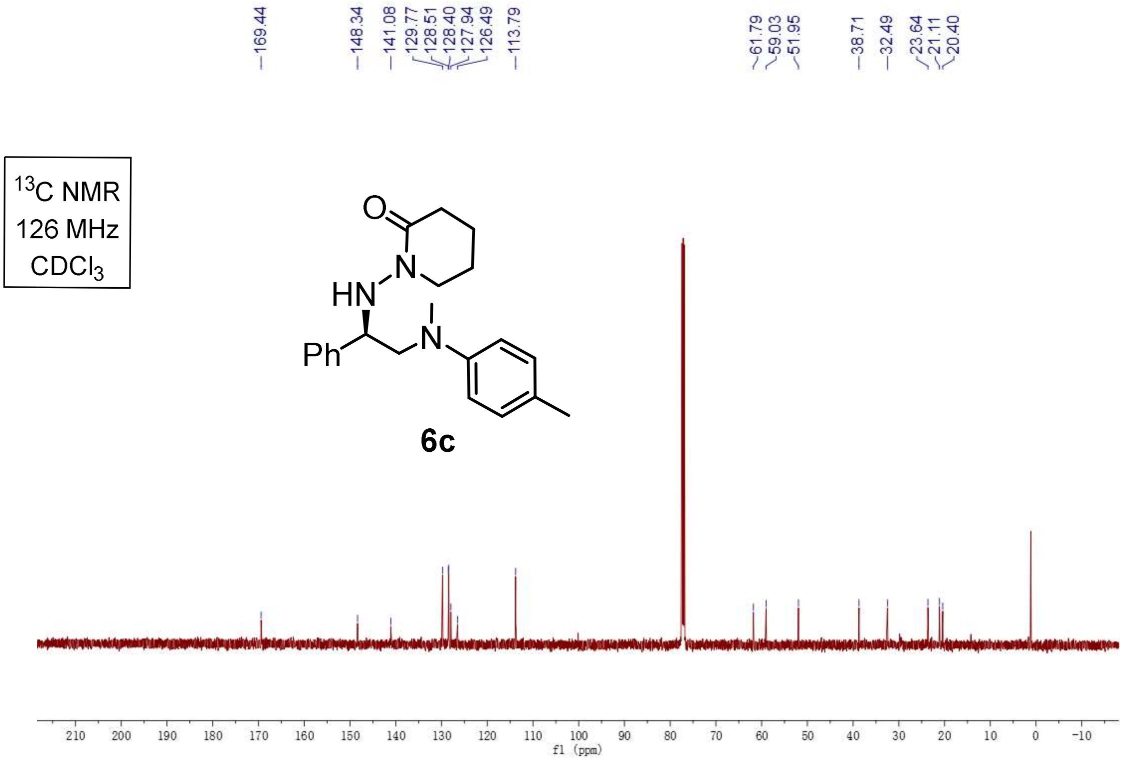

Supplementary Figure 74. ^{1}H and ^{13}C-NMR of **6c**.

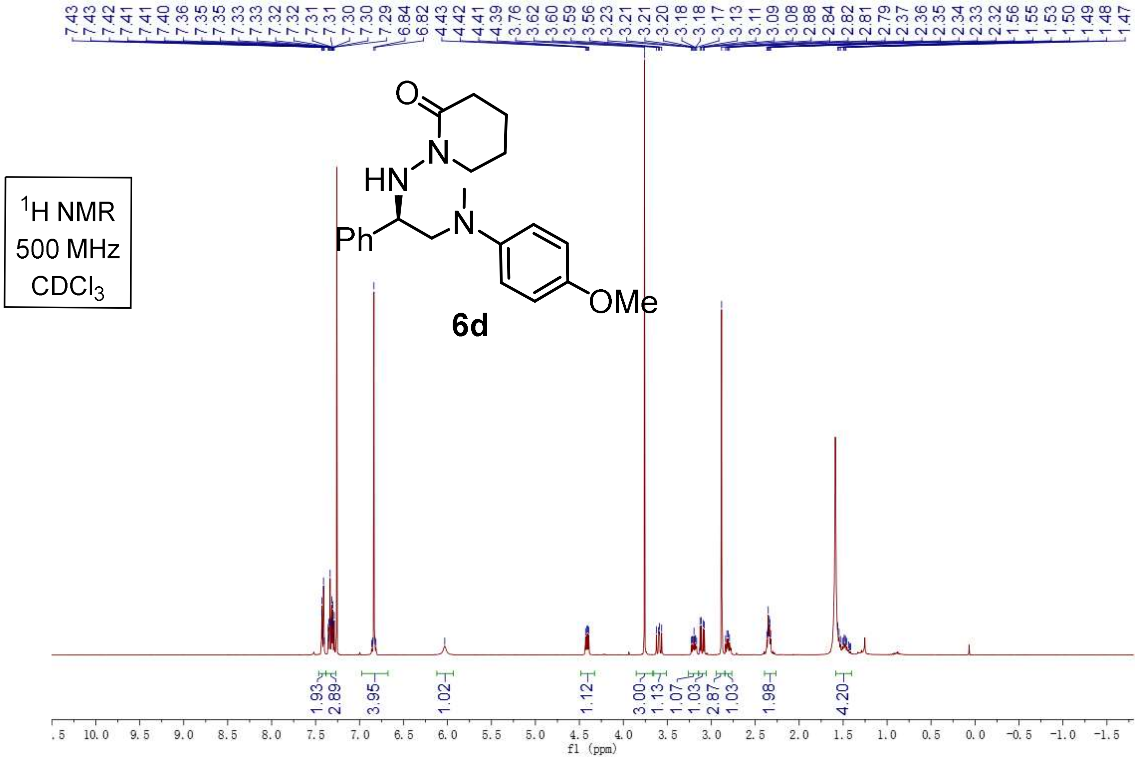

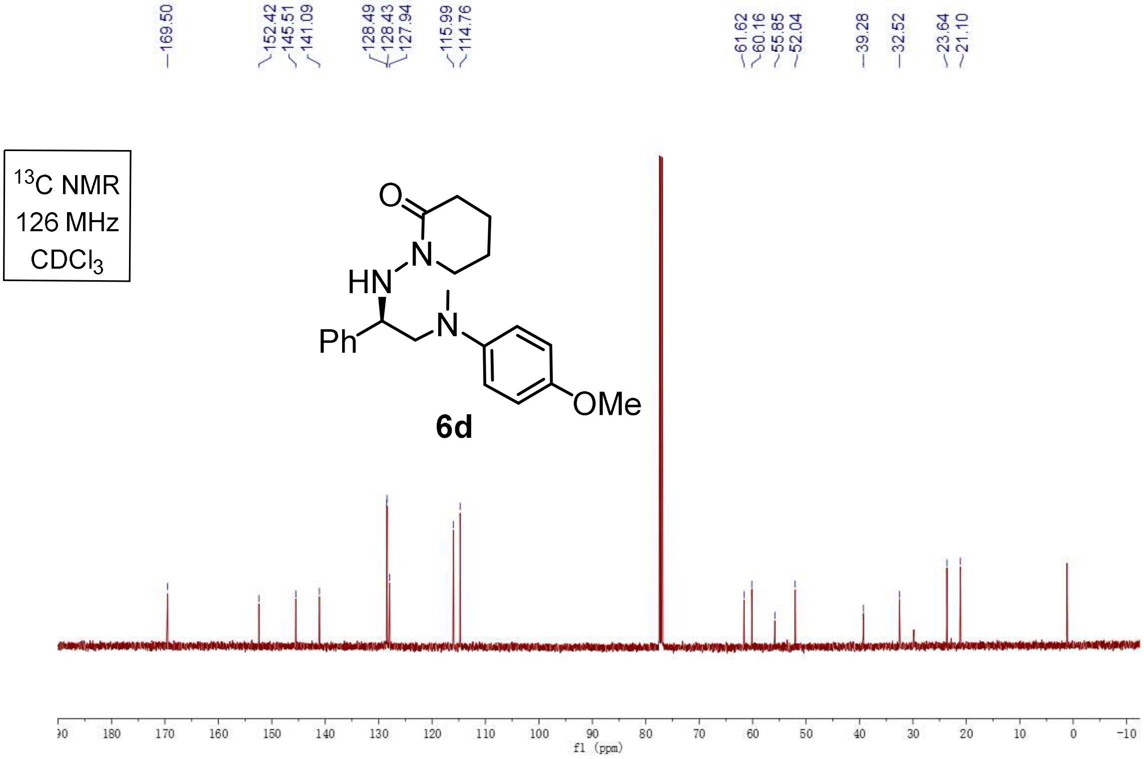

Supplementary Figure 75. ^{1}H and ^{13}C-NMR of **6d**.

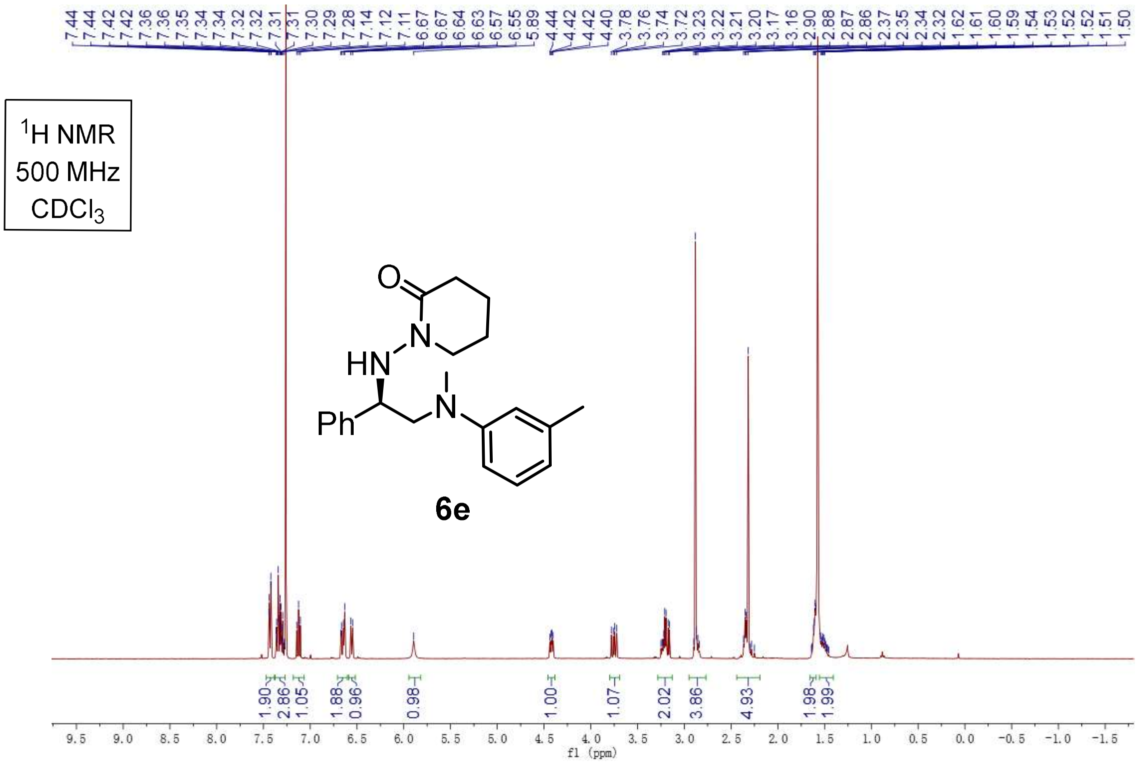

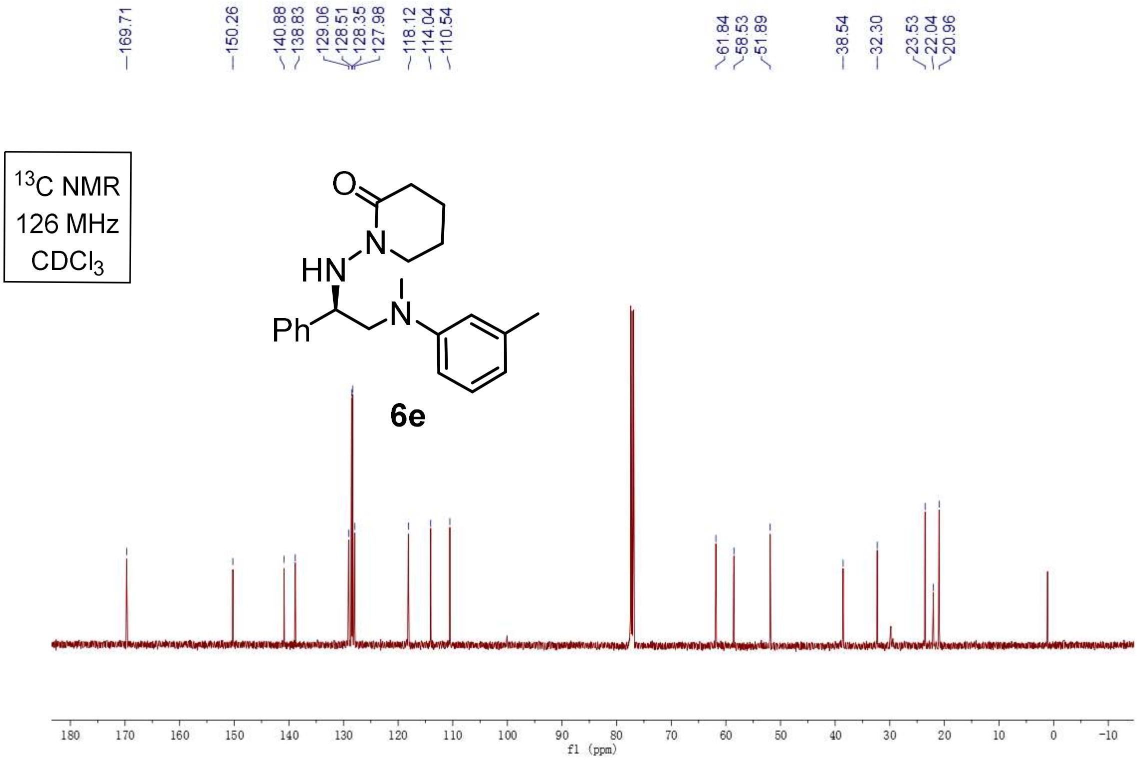

Supplementary Figure 76. ^{1}H and ^{13}C-NMR of **6e**.

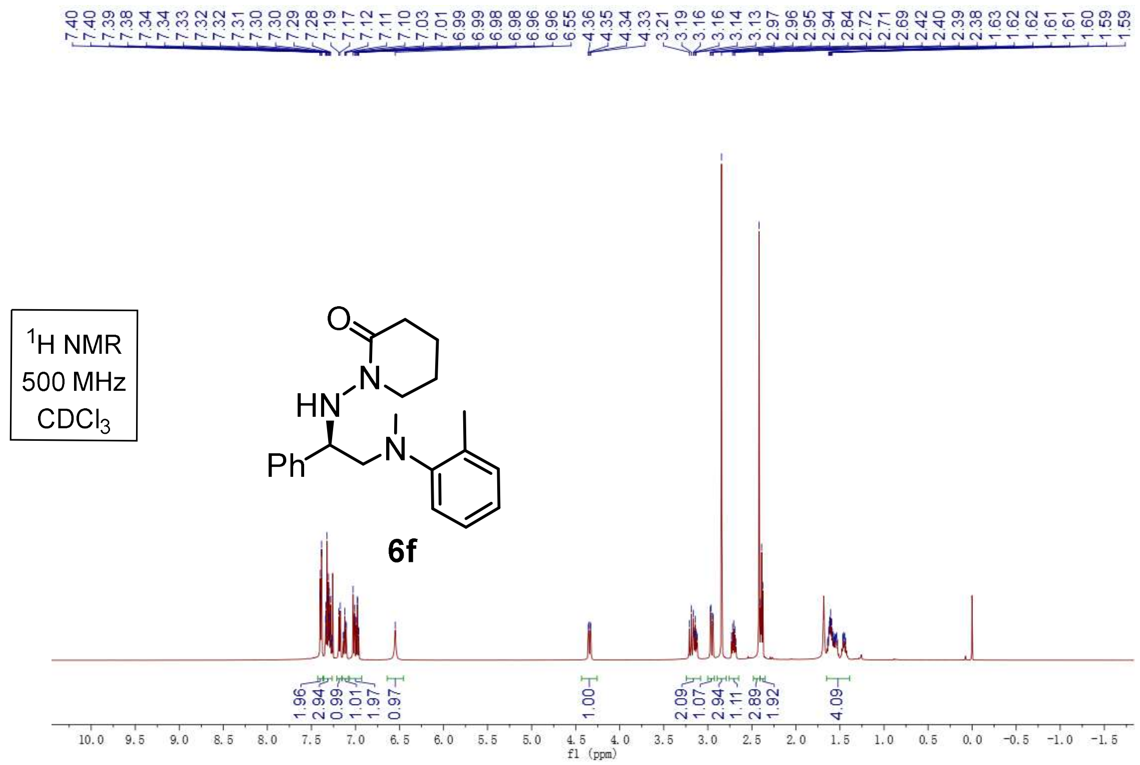

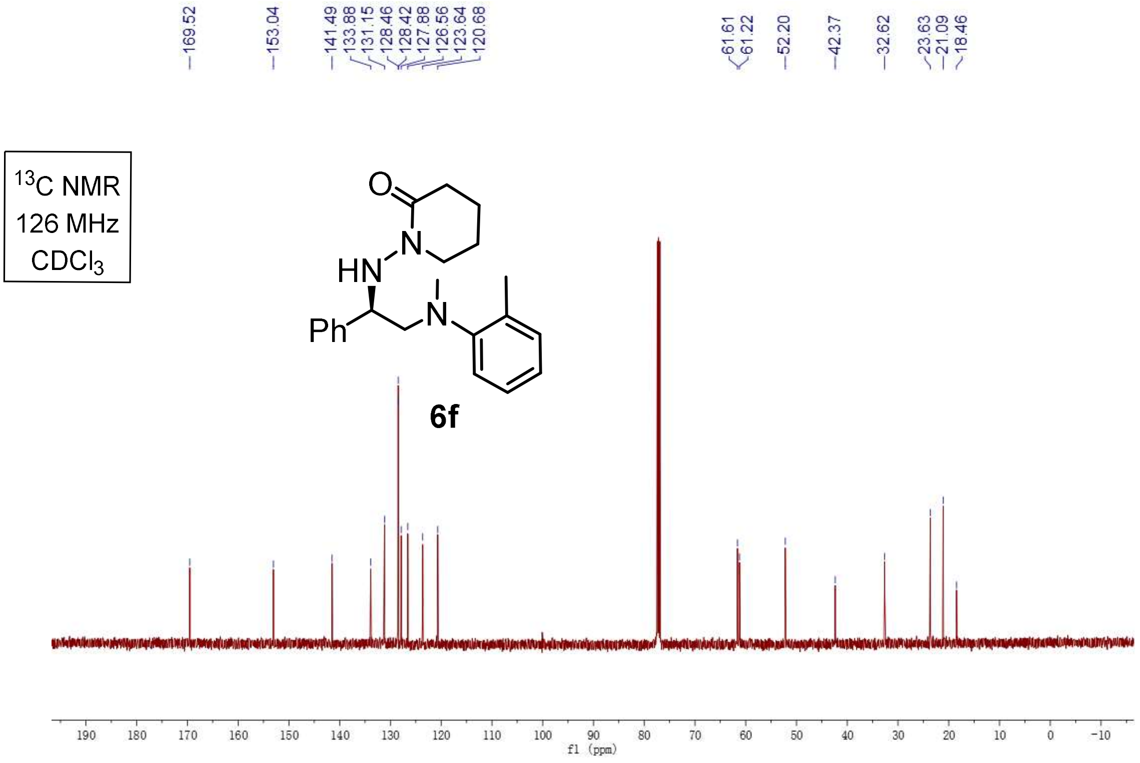

Supplementary Figure 77. ^{1}H and ^{13}C-NMR of **6f**.

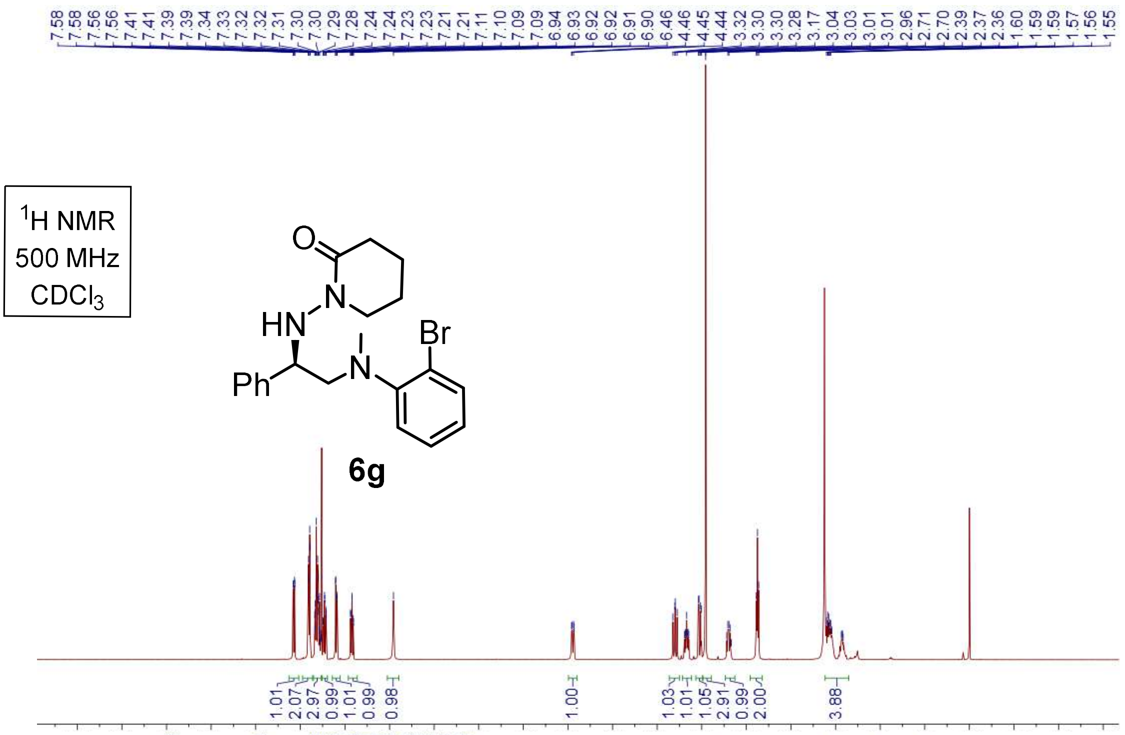

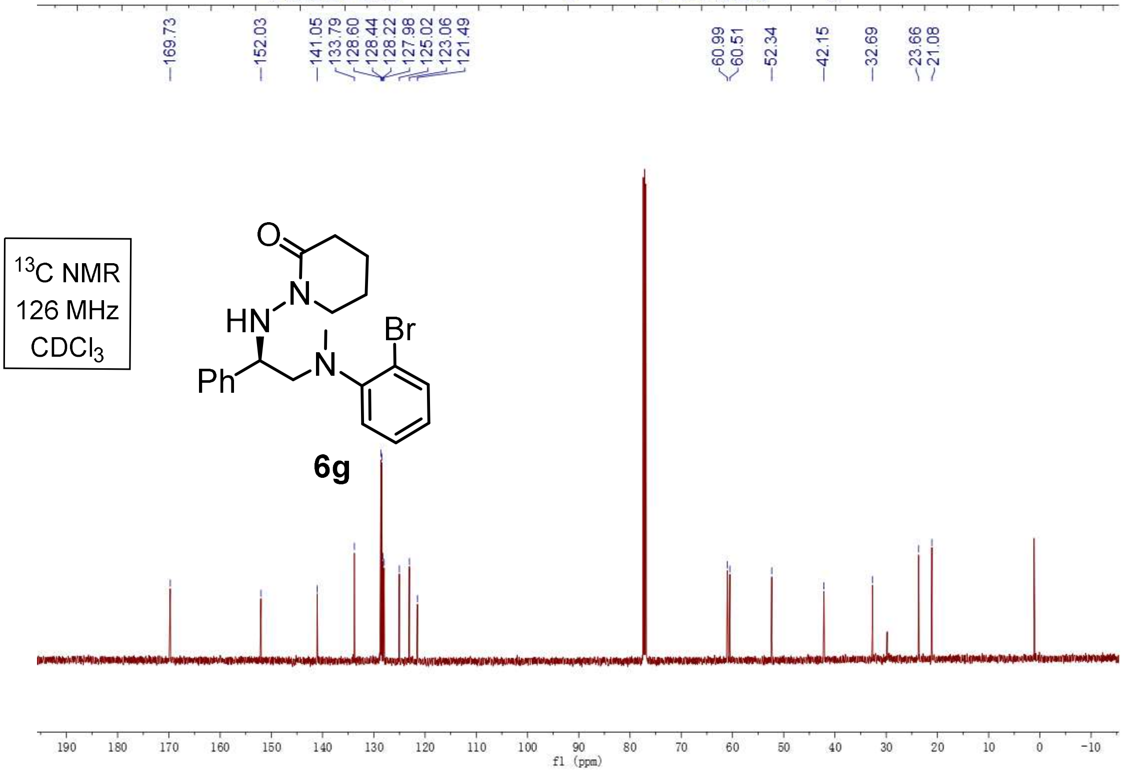

Supplementary Figure 78. ^{1}H and ^{13}C-NMR of **6g**.

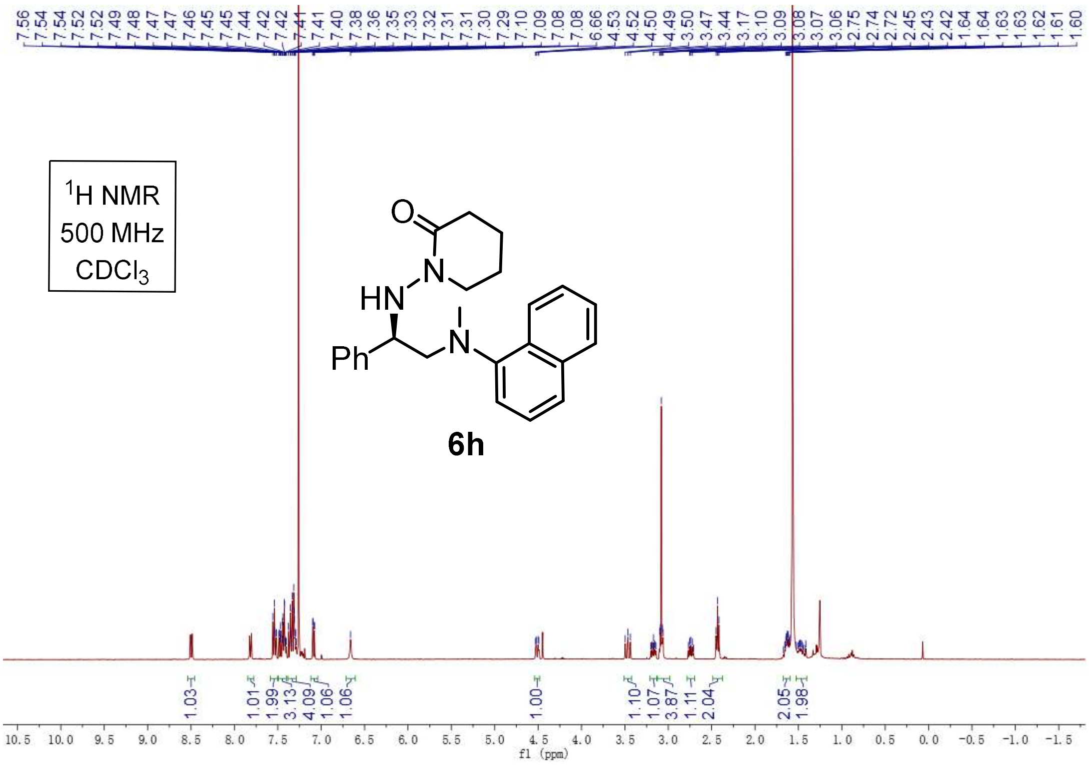

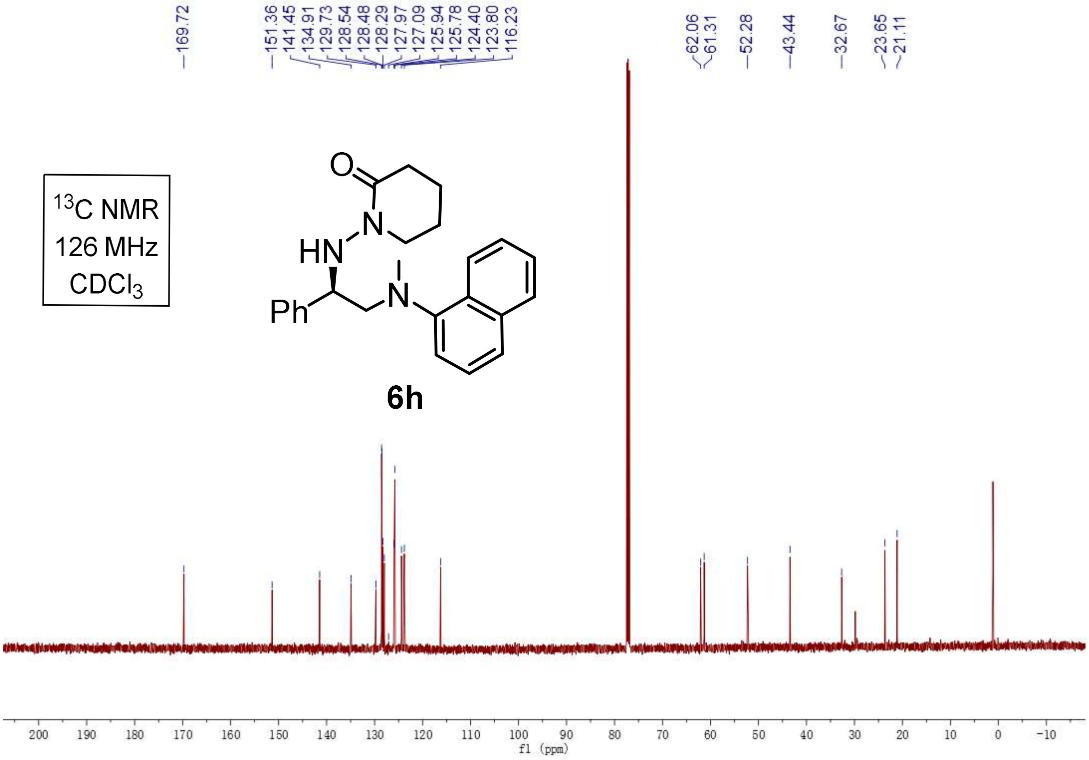

Supplementary Figure 79. ^{1}H and ^{13}C-NMR of **6h**.

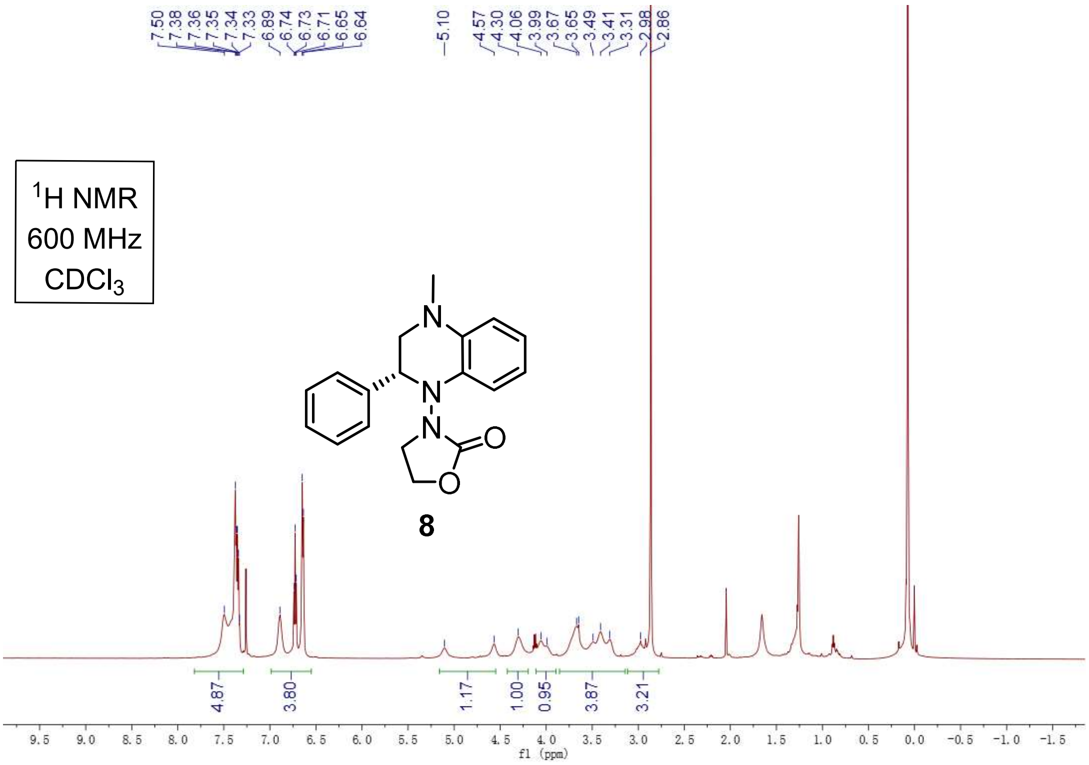

Supplementary Figure 80. ^{1}H-NMR of **8**.

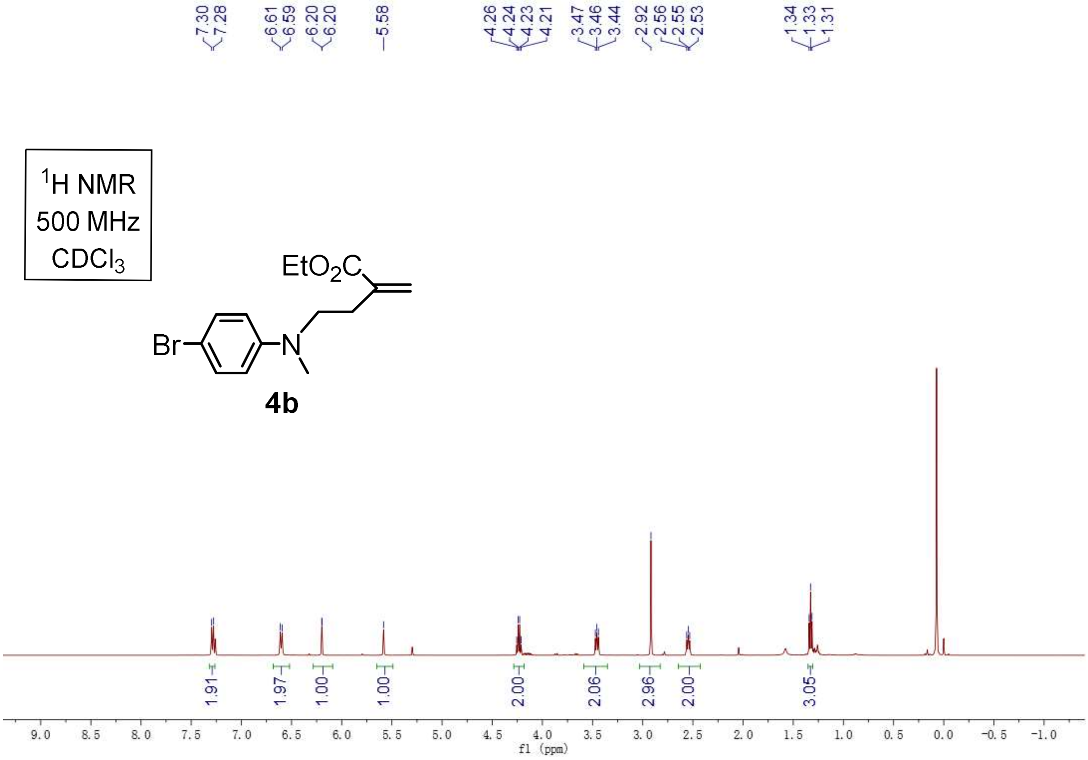

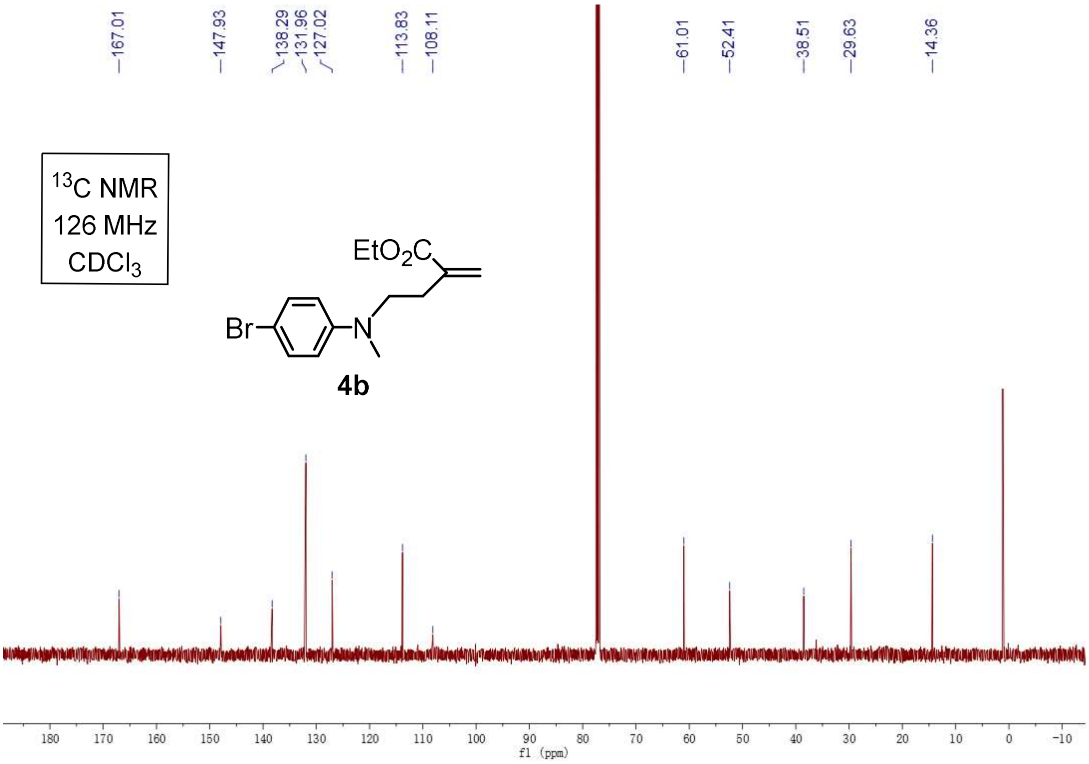

Supplementary Figure 81. ^{1}H and ^{13}C-NMR of **4b**.

Supplementary References

1 Friestad, G. K., Shen, Y. & Ruggles, E. L. Enantioselective radical addition to N-acyl hydrazones mediated by chiral Lewis acids hydrazones mediated by chiral Lewis acids. *Angew. Chem. Int. Ed.* **42**, 5061−5063 (2003).

2 Cook, G., Kargbo, R. & Maity, B. Catalytic Enantioselective indium-mediated allylation of hydrazones. *Org. Lett.* **7**, 2767−2770 (2005).

3 Kargbo, R., Takahashi, Y., Bhor, S., Cook, G., Lloyd-Jones, G. & Shepperson, I. Readily accessible, modular, and tuneable BINOL 3,3'-perfluoroalkylsulfones: highly efficient catalysts for enantioselective In-mediated imine allylation. *J. Am. Chem. Soc.* **129**, 3846−3847 (2007).

4 Ruiz Espelt, L., McPherson, I. S., Wiensch, E. M. & Yoon, T. P. Enantioselective conjugate additions of α-amino radicals via cooperative photoredox and Lewis acid catalysis. *J. Am. Chem. Soc.* **137**, 2452−2455 (2015).

5 Nakajima, K., Kitagawa, M., Ashida, Y., Miyake, Y. & Nishibayashi, Y. Synthesis of nitrogen heterocycles via α-aminoalkyl radicals generated from α-silyl secondary amines under visible light irradiation. *Chem. Commun.* **50**, 8900−8903 (2014).

6 Shen, X., Li, Y., Wen, Z., Cao, S., Hou, X. & Gong, L. A chiral nickel DBFOX complex as a bifunctional catalyst for visible-light-promoted asymmetric photoredox reactions. *Chem. Sci.* **9**, 4562−4568 (2018).

7 Chen, S., Ma, W., Yan, Z., Zhang, F., Wang, S., Tu, Y., Zhang, X. & Tian, J. Organo-cation catalyzed asymmetric homo/heterodialkylation of bisoxindoles: construction of vicinal all-carbon quaternary stereocenters and total synthesis of (−)-chimonanthidine. *J. Am. Chem. Soc.* **140**, 10099−10103 (2018).

8 Carreno, M., Hernandez-Sanchez, R., Mahugo, J. & Urbano, A. Enantioselective approach to both enantiomers of helical bisquinones. *J. Org. Chem.* **64**, 1387−1390 (1999).

9 Burguette, M. I., Fraile, J. M., Garcia, J. I., Garcia-Verdugo, E., Luis, S. V. & Mayoral, J. A. Bis(oxazoline)copper complexes covalently bonded to insoluble support as catalysts in cyclopropanation reactions. *J. Org. Chem.* **66**, 8893−8901 (2001).

10 Li, Y., Zhou, K., Wen, Z., Cao, S., Shen, X., Lei, M. & Gong, L. Copper(II)-catalyzed asymmetric photoredox reactions: enantioselective alkylation of imines driven by visible light. *J. Am. Chem. Soc.* **140**, 15850−15858 (2018).

11 Deng, C., Wang, L.-J., Zhu, J. & Tang, Y. A chiral cagelike copper(I) catalyst for the highly enantioselective synthesis of 1,1-cyclopropane diesters. *Angew. Chem. Int. Ed.* **51**, 11620−11623 (2012).

12 Sheshenev, A., Boltukhina, E., White, A. & Hii, K. Methylene-bridged bis(imidazoline)-derived 2-oxopyrimidinium salts as catalysts for asymmetric michael reactions. *Angew. Chem. Int. Ed.* **52**, 6988−6991 (2013).

13 Li, X., Zhang, X., Chen, F. & Zhang, X. Copper-catalyzed N-formylation of amines through tandem amination/hydrolysis/decarboxylation reaction of ethyl bromodifluoroacetate. *J. Org. Chem.* **83**, 12815−12821 (2018).

14 Liao, W., Chen, W., Chen, C., Lim, T. & Luh, T. Photoinduced electron transfer as a probe for the folding behavior of dimethylsilylene-spaced alternating donor−acceptor oligomers and polymers. *Macromolecules* **46**, 1305−1311 (2013).
